# Global, regional, and national time trends in ischaemic heart disease incidence over three decades (1990–2019): an age-period-cohort analysis of the global burden of disease study 2019

**DOI:** 10.3389/fcvm.2024.1396380

**Published:** 2024-11-01

**Authors:** Juan Tang, Shaobo Hu, Xiaozhu Liu, Huan Li, Lirong Kuang, Lei Zhang, Wenzhai Cao, Ting Zhang, Xiaoyan Guan, Lang Li, Yutao Zhang, Shengxian Peng, Qingwei Zhang, Xiaoqian Zhou

**Affiliations:** ^1^ Scientific Research Department, First People’s Hospital of Zigong City, Zigong, China; ^2^ Department of Neurosurgery, The Affiliated Li Huili Hospital, Ningbo University, Ningbo, China; ^3^ Emergency and Critical Care Medical Center, Beijing Shijitan Hospital, Capital Medical University, Beijing, China; ^4^ Chongqing College of Electronic Engineering, Chongqing, China; ^5^ Department of Ophthalmology, Wuhan Wuchang Hospital (Wuchang Hospital Affiliated to Wuhan University of Science and Technology), Wuhan, China; ^6^ Guangdong Provincial Institute of Biological Products and Materia Medica, Guangzhou, China; ^7^ Department of Cardiology, First People’s Hospital of Zigong City, Zigong, China; ^8^ Scientific Rresearch Center, Sichuan Vocational College of Health and Rehabilitation, Zigong, China; ^9^ First People’s Hospital of Zigong City, Zigong Academy of Medical Sciences, Zigong, China; ^10^ Department of Pathology, First People’s Hospital of Zigong City, Zigong, China; ^11^ Key Laboratory Gastroenterology and Hepatology, State Key Laboratory for Oncogenes and Related Genes, Division of Gastroenterology and Hepatology, Ministry of Health, School of Medicine, Renji Hospital, Shanghai Institute of Digestive Disease, Shanghai Jiao Tong University, Shanghai, China; ^12^ Department of Cardiovascular, The Third Affiliated Hospital of Wenzhou Medical University, Wenzhou, China

**Keywords:** incidence, ischemic heart disease, age-period-cohort, healthy, differences

## Abstract

**Introduction:**

To assess the prevailing trends in the incidence of ischemic heart disease (IHD) across 204 countries and territories from 1990 to 2019, and to elucidate their correlations with age, period, and birth cohort, a comprehensive analysis was conducted.

**Methods:**

From 1990 to 2019, we employed the Global Burden of Disease Study (GBD) Results Tool in conjunction with an age-period-cohort model. This approach facilitated the estimation of annual percentage changes in incidence, referred to as net drifts, encompassing the overall population. Additionally, we calculated annual percentage changes spanning ages 15 - 19 to 95 + years, denoted as local drifts. Furthermore, our analysis involved determining period and cohort relative risks, elucidating the effects associated with distinct periods and birth cohorts.

**Results:**

Globally, 21,203,479 [95% uncertainty interval (UI): 18,799,322 − 23,704,124] cases of IHD occurred in 2019. There were 33 countries with at least 100000 cases. Between 1990 and 2019, the net drift of IHD incidence exhibited a range from −1.7% per year [95% confidence interval (CI): −1.79, −1.61] in countries with a high socio-demographic index (SDI) to 0.08% per year (95% CI: 0.05, 0.11) in countries with a low SDI. Age effects across all countries and genders demonstrated an increasing trend over time, indicating age as a significant risk factor for IHD. Moreover, period and cohort effects in higher SDI countries exhibited a more rapid decline in both genders compared to lower SDI countries. The findings indicated that nations with a higher SDI manifested overall favorable trends in the relative risk of IHD incidence, both across time and in successive younger birth cohorts.

**Discussion:**

The incidence of IHD serves as a valuable and accessible indicator for assessing trends in IHD provision, spanning from early youth through later life. Enhancements in IHD prevention have the potential to mitigate risks for successively younger cohorts and, over time, redistribute the risk across all age groups. Despite global declines in IHD incidence over the last three decades, decreasing trends in incidence have slowed and, in some countries, flattened. Many countries have experienced unfavorable period and cohort effects.

## Introduction

The term “ischemic heart disease” (IHD) encompasses a group of chronic conditions characterized by coronary ischemia and consequential damage to the cardiac muscle. While incidence and mortality rates associated with IHD are decreasing in most developed and developing nations, the absolute numbers of cases continue to rise (1). Even in many European countries with high-income, IHD persists as a prominent contributor to mortality, morbidity, and economic burden (2, 3), and has become a global public health issue (4, 5). In the Arab States, China, Latin America,and India, according to epidemiological studies, the prevalence of IHD-related illnesses and deaths has increased quickly, in contrast to Western European nations (5–9).

Some studies indicate that the prevalence and severity of IHD can be significantly ameliorated if risk factors are appropriately managed (4, 6, 10). However, the escalating global burden of ischemic heart disease (IHD) is exacerbated by social-demographic disadvantages, inadequate access to healthcare, and the suboptimal performance of healthcare systems (10, 11). The incidence and prevalence of IHD exhibit variations across age groups, genders, and regions; nevertheless, there is a scarcity of specific studies addressing the worldwide burden of IHD and its associated risk factors (4, 12, 13).

The existing literature from high-income countries indicates a global decrease in the incidence of ischemic heart disease (IHD) (14, 15). However, these analyses did not differentiate the respective contributions of age, period, and cohort impacts on incidence. Many countries worldwide, particularly those with low- and middle-income status, lack comprehensive data on trends in IHD incidence or their associations with age, temporal changes, and birth cohorts. This study aimed to leverage GBD 2019 data and age-period-cohort models to evaluate the influence of age, gender, region, and the socio-demographic index (SDI) on global trends in IHD incidence from 1990 to 2019. Additionally, the study sought to investigate whether there was a diminishing decline in the incidence of IHD during this period.

## Methods

### Data collection

GBD provides comprehensive and standardized estimates of descriptive epidemiological data for 359 diseases and injuries starting in 1990, using methods that are consistent across countries to enable comparisons between countries (3). Details on the detailed definition, data inputs, processing, synthesis, and final modeling of IHD and its incidence can be found in the companion publication to GBD (3, 12, 16). ICD-10 codes I20–I25, as well as ICD-9 and ICD-8 codes 4,100–4,149, define ischemic heart disease as coronary artery disease resulting in myocardial infarction or ischemia (17). The estimation of the global burden of IHD in all countries relies on the GBD network's utilization of standardized tools within a Bayesian framework. This approach facilitates the integration of all available data spanning time, age, geography, and various health domains to generate comprehensive disease estimates. In this study, incidence estimates and their 95% uncertainty intervals (UI) were derived from GBD 2019 using the 25th and 975th sequential values of 1,000 draws from the posterior distribution (3). The GBD utilized diverse data sources, including national cause of death records, hospital registries, national or regional surveys, and surveillance data, to ascertain the incidence of IHD (defined as the number of new cases of the disease per 100,000 population). Age-standardized incidence rates (ASIRs) were reported per 100,000 individuals after adjusting for age using the GBD standard.

In this study, the SDI serves to categorize countries along the development spectrum, classifying them into five SDI quintiles. This classification is based on the composite factors of per capita income distribution, average educational attainment above the age of 15, and total fertility rate below the age of 25 (11). Notably, secondary analysis of the data in this study did not require approval or consent from an institutional review board or ethics committee.

### Analysis of overall temporal trends in IHD incidence

To assess temporal trends in incidence during the study period, metrics such as all-age (crude) incidence rate, ASIR, and the percentage change in incidence between 1990 and 2019 were employed. The ASIR calculations were based on the global age-standardized population data from GBD 2019.

### Age-period-cohort modelling analysis of incidence data

This study employs an age-period-cohort (APC) model to analyze incidence trends based on age, time, and birth cohort dynamics (18). Going beyond traditional epidemiological analysis, the APC model aims to dissect the influences of biological factors associated with age, as well as technological and social factors, on disease trends (19). This methodology has found application in the descriptive epidemiology of certain chronic diseases, including cardiovascular diseases (19). The APC model fits a log-linear Poisson model across a Lexis diagram of observed rates, capturing the combined effects of age, period, and birth cohort. However, the linear relationship between age, period, and cohort (birth cohort = period—age) presents an identification problem, making it statistically impossible to estimate their individual effects (18, 19). This study addresses this issue by developing estimable APC parameters and functions without constraining model parameters (18). The APC model is implemented using freely accessible R tools, and the methodology is detailed in previous publications (20).

The APC model uses IHD incidence estimates from GBD 2019 in addition to population data from each country or area. All age groups and time intervals in a standard APC model should have the same length, therefore using age groups of five years and time intervals of five years would be correct. Given that the GBD estimates are produced in an unevenly spaced data format with five-year age groups and annual data, we standardized the dataset into a unified framework. This involved selecting incidence and population counts from the midpoint of six consecutive five-year periods to represent the respective periods. There were 17 age categories present in the input data (from 15 to 19 to 95 plus in five year age group intervals). The annual percentage change in incidence (that is, the net drift of incidence, % per year) was calculated using the fitted APC model to estimate the overarching temporal trend in incidence. Both the trend due to the passage of time and the trend due to the subsequent cohorts contribute to the overall drift. The APC model also computed the temporal trend of incidence within each age group, quantified as the annual percentage change in age-specific incidence (referred to as the local drift of incidence,% per year). This measure reflects trends in birth cohort effects (20). There is a consensus that a ±10%, ±18%, or ±26% change in the fitted rate over a 10, 20, or 30-year period constitutes a significant shift in mortality, with a drift of ±1% per year or more meeting this threshold. The significance of changes in annual percentage change was examined using the Wald chi-squared test (20). In addition to period (cohort) relative risks of incidence for each period (cohort), the APC model outputs fitted longitudinal age-specific rates in the referent cohort adjusted for period deviations, representing age-associated natural history (i.e., age effects) (20). The determination of relative risk involves the division of age-specific rates for each cohort during a specific period by the rates for a reference cohort during a reference period. The complete extent of the net drift is captured in the rate ratio curves for both period and cohort. The interpretability of the results remains consistent irrespective of the chosen reference time period or cohort. Two-tailed tests were executed, considering a *p*-value less than 0.05 as statistically significant. The analysis was carried out using R version 3.6.3.

## Results

### Overall Status of ischemic heart disease burden, 1990–2019

**Figure 1:**
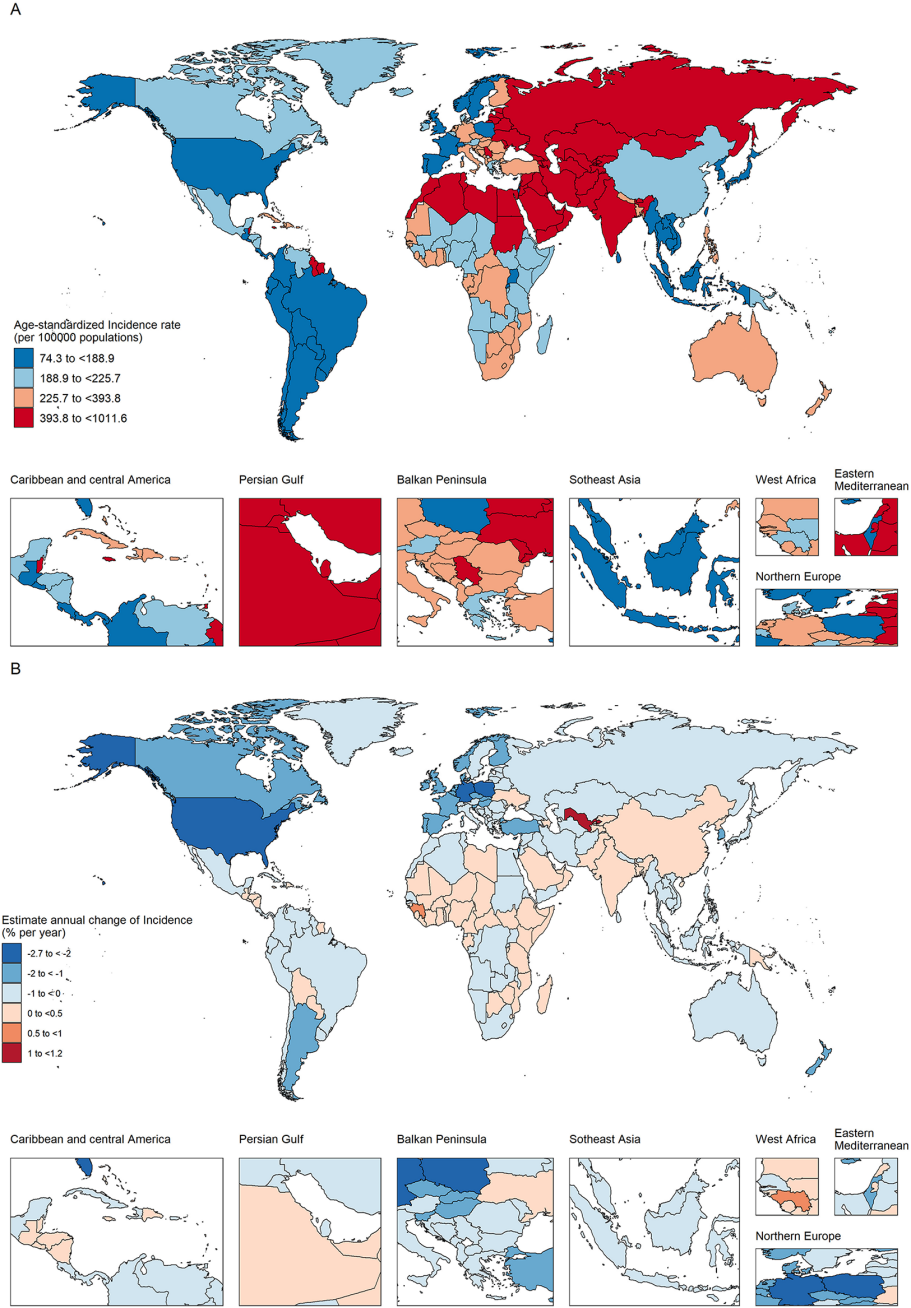
The age-standardized incidence in 2019 **(A)** and net drift of incidence during 1990–2019 **(B)** for ischemic heart disease in 204 countries and territories **(A)**. **(A)** World map of age-standardized ischemic heart disease incidence in 2019; **(B)** world map of net ischemic heart disease incidence drifts, i.e., estimated annual percentage change in mortality from the age-period-cohort model. Net drift captures components of the trends attributable to calendar time and successive birth cohorts.

The overall number of cases, ASIR, and the net drift of incidences are shown in Table 1; Figure 1, and Supplementary Table S1; Figure S1, respectively [estimated using the APC model, it is comparable to the annual percentage change in incidences but accounts for the impact of calendar time and successive birth cohorts (21)]. Globally, the incident cases of IHD have risen to 2.1 million [95% (UI): 1.9–2.4] with an ASIR of 262.4 (233.3–293.3) per 100,000 persons in 2019 (Table 1). The number of IHD incident cases witnessed an increase between 1990 and 2019, with a notable rise in middle and low SDI countries (2.57 and 2.28 times), and a less pronounced increase in high SDI countries. The APC model estimated a net drift of IHD incidence globally, ranging from −1.7% (−1.79 to −1.67) in high-SDI regions to 0.08% (0.05 to 0.11) in low-SDI regions, with an overall estimate of −0.54% (95% CI −0.59 to −0.49) per year. The upper bound of the 95% CI for the net drifts for 25 countries was <−1.0%. Between 1990 and 2019, the proportion of global IHD cases in low and low-middle SDI regions increased.

**Table 1:** Trends in ischemic heart disease incidence across socio-demographic index quintiles and 21 regions, 1990–2019.

Location	Case_1990	Case_2019	Case_percent_change	ASR_1990	ASR_2019	ASR_percent_change	AAPC	Netdrift
Global	11,752,028 (10,376,504, 13,161,208)	21,203,479 (18,799,322, 23,704,124)	80.42 (78.52, 82.3)	316.4 (282.16, 352.31)	262.39 (233.25, 293.26)	−17.07 (−17.88, −16.15)	−0.71 (−0.96, −0.46)	−0.54 (−0.58, −0.49)
High SDI	3,394,012 (3,030,051, 3,770,664)	3,517,780 (3,162,403, 3,900,932)	3.65 (1.15, 6.1)	327.28 (293.49, 363.61)	190.25 (170.65, 210.92)	−41.87 (−43.16, −40.46)	−1.89 (−1.97, −1.81)	−1.7 (−1.79, −1.61)
High-middle SDI	3,308,750 (2,935,809, 3,702,399)	5,276,430 (4,677,607, 5,892,903)	59.38 (56.7, 62.29)	337.27 (300.38, 375.8)	262.97 (233.69, 292.92)	−22.07 (−22.97, −21.25)	−0.93 (−1.07, −0.79)	−0.82 (−0.89, −0.75)
Middle SDI	2,442,338 (2,158,661, 2,736,120)	6,285,634 (5,560,731, 7,029,327)	148.33 (144.14, 152.68)	258.59 (229.8, 288.64)	265.85 (237.3, 296.27)	−0.83 (−1.68, 0.1)	0.03 (−0.13, 0.2)	0.11 (0.08, 0.15)
Low-middle SDI	1,912,056 (1,669,721, 2,159,093)	4,543,167 (3,996,952, 5,099,263)	136.14 (131.99, 140.58)	340.36 (298.6, 383.2)	343.56 (303.89, 384.63)	0.34 (−1, 1.79)	−0.03 (−0.24, 0.18)	0.08 (0.03, 0.13)
Low SDI	688,265 (599,420, 782,461)	1,567,876 (1,375,007, 1,767,494)	114.62 (110.7, 118.36)	310.3 (271.43, 350.92)	315.23 (276.37, 355.47)	−4.15 (−5.82, −2.58)	0.01 (−0.07, 0.1)	0.08 (0.05, 0.11)
High-income Asia Pacific	300,287 (261,360, 340,105)	540,931 (466,370, 621,714)	80.14 (71.31, 89.32)	155.32 (135.77, 175.68)	121.99 (106.39, 138.37)	−21.46 (−22.94, −20)	−0.84 (−1.04, −0.64)	−0.92 (−1.02, −0.82)
High-income North America	1,340,416 (1,162,450, 1,522,945)	1,089,691 (990,873, 1,202,617)	−18.7 (−23.82, −13.19)	381.57 (332.99, 435.49)	177.66 (162.19, 195.21)	−53.44 (−56.33, −50.2)	−2.69 (−2.84, −2.53)	−2.55 (−2.73, −2.38)
Western Europe	1,867,490 (1,695,306, 2,044,803)	1,800,608 (1,619,127, 2,000,180)	−3.58 (−6.54, −0.43)	325.3 (296.45, 356.28)	204.99 (182.89, 227.18)	−36.99 (−38.84, −35.13)	−1.59 (−1.68, −1.51)	−1.39 (−1.47, −1.3)
Australasia	110,662 (99,437, 122,460)	164,211 (145,122, 185,526)	48.39 (39.91, 57.2)	479.15 (431.18, 530.38)	345.24 (304.09, 388.83)	−27.95 (−31.67, −23.88)	−1.16 (−1.26, −1.05)	−0.93 (−1, −0.86)
Andean Latin America	16,540 (14,485, 18,713)	43,311 (37,925, 48,754)	161.86 (153.85, 171.4)	85.19 (74.48, 96.52)	78.57 (68.7, 88.61)	−7.78 (−10.56, −4.62)	−0.29 (−0.73, 0.15)	−0.24 (−0.31, −0.16)
Tropical Latin America	111,578 (98,385, 125,583)	266,234 (235,028, 299,902)	138.61 (131.94, 145.4)	124.57 (109.59, 139.94)	109.7 (96.8, 123.31)	−11.94 (−14.47, −9.39)	−0.49 (−0.59, −0.39)	−0.45 (−0.53, −0.38)
Central Latin America	169,412 (148,920, 190,786)	438,233 (386,397, 494,737)	158.68 (153.28, 164.29)	206.88 (181.69, 233.8)	187.3 (165.07, 211.56)	−9.46 (−10.83, −8.17)	−0.37 (−0.46, −0.28)	−0.4 (−0.44, −0.35)
Southern Latin America	103,206 (93,379, 113,853)	136,342 (121,658, 152,979)	32.11 (26.14, 38.79)	236.3 (214.64, 259.69)	162.65 (145.17, 182.51)	−31.17 (−34.12, −27.58)	−1.46 (−1.74, −1.17)	−1.1 (−1.24, −0.96)
Caribbean	101,532 (89,953, 113,864)	189,668 (166,876, 213,864)	86.81 (82.48, 91.63)	391.45 (345.7, 438.7)	366.45 (322.99, 412.58)	−6.39 (−8.53, −4.1)	−0.19 (−0.31, −0.07)	−0.16 (−0.19, −0.12)
Central Europe	535,772 (483,181, 592,264)	537,054 (485,750, 590,992)	0.24 (−2.18, 2.83)	384.3 (348.44, 422.5)	253.73 (228.75, 277.88)	−33.98 (−35.76, −32.31)	−1.68 (−2.41, −0.95)	−1.33 (−1.43, −1.24)
Eastern Europe	1,383,430 (1,212,414, 1,564,799)	1,774,308 (1,559,226, 2,003,076)	28.25 (25.14, 31.72)	535.16 (473.43, 600.66)	514.43 (455.64, 579.41)	−3.87 (−5.4, −2.36)	−0.2 (−0.3, −0.1)	−0.23 (−0.3, −0.17)
Central Asia	262,559 (234,485, 290,450)	424,451 (383,911, 466,922)	61.66 (57.11, 66.63)	598.99 (542.29, 659.96)	652.39 (600.19, 709.39)	8.91 (6.13, 12.08)	0.25 (0.15, 0.35)	−0.02 (−0.11, 0.08)
North Africa and Middle East	1,084,677 (972,520, 1,201,363)	2,550,432 (2,287,730, 2,826,390)	135.13 (131.3, 139.34)	674.52 (612.2, 740.46)	613.87 (555.84, 675.16)	−8.99 (−10.26, −7.5)	−0.35 (−0.41, −0.28)	−0.32 (−0.37, −0.27)
South Asia	2,250,012 (1,942,977, 2,562,189)	5,841,779 (5,123,255, 6,589,464)	159.63 (154.76, 165.06)	425.76 (371.42, 481.88)	427.59 (374.87, 480.92)	0.43 (−0.82, 1.76)	−0.09 (−0.44, 0.26)	0.08 (0.01, 0.16)
Southeast Asia	364,741 (319,048, 413,311)	803,047 (706,798, 898,992)	120.17 (116.53, 124.08)	147.54 (130.25, 166.31)	135.91 (121.02, 151.44)	−7.88 (−9.51, −6.13)	−0.33 (−0.86, 0.21)	−0.29 (−0.32, −0.26)
East Asia	1,309,173 (1,151,725, 1,473,947)	3,616,375 (3,189,267, 4,067,850)	176.23 (166.01, 187.23)	176.97 (156.66, 199.01)	195.88 (174.64, 220.69)	10.69 (8.78, 12.66)	0.36 (0.3, 0.41)	0.27 (0.2, 0.35)
Oceania	5,688 (4,948, 6,488)	14,069 (12,272, 16,051)	147.35 (139.04, 155.22)	203.26 (176.48, 231.91)	209.06 (183.44, 237.25)	2.85 (−0.31, 5.79)	0.09 (−0.05, 0.24)	0.1 (−0.02, 0.23)
Western Sub-Saharan Africa	173,522 (150,640, 198,000)	397,292 (349,366, 449,940)	128.96 (124.99, 133.76)	208.45 (180.4, 238.39)	219.35 (191.01, 250.24)	5.23 (4.05, 6.4)	0.19 (0.14, 0.24)	0.19 (0.16, 0.21)
Eastern Sub-Saharan Africa	142,115 (123,923, 162,271)	325,498 (285,653, 367,845)	129.04 (125.09, 133.33)	199.13 (173.32, 227.2)	203.17 (177.75, 231.37)	2.03 (0.51, 3.43)	0.06 (0.03, 0.08)	0.08 (0.06, 0.11)
Central Sub-Saharan Africa	50,069 (43,496, 56,588)	115,886 (102,050, 130,655)	131.45 (123.79, 139.83)	236.78 (206.23, 267.54)	227.68 (200.47, 256.11)	−3.84 (−7.03, −0.43)	−0.16 (−0.19, −0.13)	−0.17 (−0.22, −0.13)
Southern Sub-Saharan Africa	69,147 (60,332, 78,510)	134,059 (116,828, 152,763)	93.87 (90.63, 97.41)	255.71 (222.1, 292.05)	244.12 (213.03, 278.46)	−4.53 (−6.18, −2.87)	−0.14 (−0.17, −0.1)	−0.2 (−0.23, −0.16)

Age-standardized incidence rate is computed by direct standardization with global standard population in GBD 2019. Net drifts are estimates derived from the age-period-cohort model and denotes overall annual percentage change in incidence, which captures the contribution of the effects from calendar time and successive birth cohorts. SDI, socio-demographic index; APC, age-period-cohort; AAPCs, average annual percentage changes.

### Regional and national trends in IHD incidences, 1990–2019

From 1990 to 2019, progress in reducing this incidence rate has likewise varied greatly. Although ASIR climbed in some countries, they fell in the majority of countries. The largest decreases in ASIR from 1990 to 2019 were in high- and high-middle SDI countries, with an overall AAPC of −1.89% (95% UI: −1.97 to −1.81) and −0.93% (95% UI: −1.07 to −0.79). Among the 21 GBD regions, Central Asia had the highest ASIR for IHD in 2019 (652.4 cases/100,000 people, 95% UI: 600.2–709.4), followed by North Africa and the Middle East, and then Eastern Europe (Table 1; Figure 1). The incidence rates of IHD is relatively high in young people in the Caribbean, Australasia. The lower the level of SDI, the higher the rate in young people. In addition, the incidence of IHD in the elderly was shown to be greater in Western and Central Europe. North Africa and the Middle East have the highest all-age incidence. Out of a total of 204 countries and territories, 33 had at least 100,000 cases. India [number of cases = 4.7 million, (95% UI 4.1 to 5.4 million)], China [3.5 million, (3.1 to 3.9 million)] and the Russian Federation [1.1 million, (0.9–1.2 million)] had the highest cases, accounting for 44.1% of global cases. Twenty of the 33 countries exhibited a rising incidence trend (net drifts ≥0.0% per year) or a slight decline (−0.5% to 0.0%). Uzbekistan’s all-age incidence increased by the most (from 0.6 [0.54–0.66] to 1.4 [1.3–1.6] per 100,000), with a yearly net drift of 1.18% [1.02–1.34].In 2019, 29 countries had ASIR that were greater than two times the global average, while only eight of these countries had lower SDIs. Despite favorable incidence reductions commonly being observed in higher-SDI countries in Western and Central Europe and North America, the United Arab Emirates had a particularly high incidence in 2019 [654per100,000 (565.64–754.13)] with a moderately flat net drift in the incidence of 0.2% (−0.1 to 0.51)per year.

In summary, these findings indicate that the trends in IHD incidence exhibit disparities among countries, and the increases in incidence do not necessarily align with expectations based on the country-level SDI status. This is exemplified by instances such as the United Arab Emirates and Saudi Arabia. In addition, the direction of change in incidence shown by traditional indicators (age-standardized incidence rates) may not exactly match the change shown by the net drift derived from the APC model. This suggests that it is important to distinguish between period and cohort trends in IHD incidence.

### Trends in IHD incidence across age groups over time

**Figure 2:**
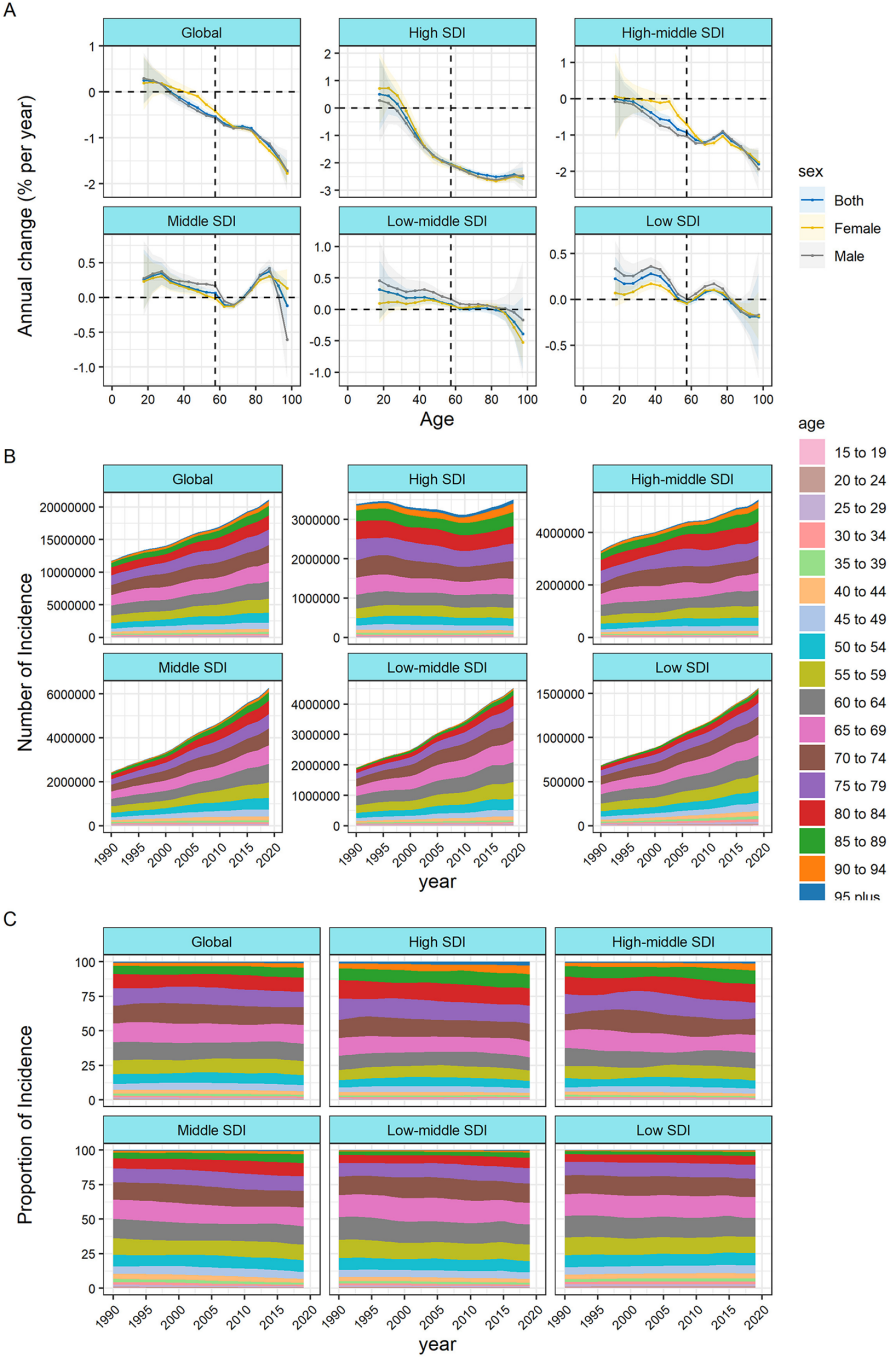
Local drifts of ischemic heart disease incidence and age distribution of ischemic heart disease incidence by SDI quintiles, 1990–2019. **(A)** Local drifts of ischemic heart disease incidence (estimates from age-period-cohort models) for 19 age groups (5–9 to 95+ years), 1990–2019 The dots and shaded areas indicate the annual percentage change of incidence (% per year) and the corresponding 95% CIs. **(B)** Temporal change in the absolute cases of ischemic heart disease incidence across age groups, 1990–2019 **(C)** temporal change in the relative proportion of ischemic heart disease incidence across age groups, 1990–2019 SDI, socio-demographic Index.

Figure 2A presents the annual percentage change in the incidence rate of IHD for each age group (i.e., the local drift of mortality estimated from the APC model, capturing trends in birth cohort effects) from 15 to 19 to 95+ years. On a global scale, there is a tendency for the incidence of IHD to decrease across all age groups (*p* < 0.001). However, the extent of the decline does not appear to be notably significant. The steepest decline in incidence occurred among those over 50 years oldin high-SDI countries, and this downward trend was maintained at a certain level among the elderly. In countries characterized by middle SDI, low-middle SDI, and low SDI, the incidence is showing an increasing trend, albeit with fluctuations, in individuals under 80 years of age. In age groups where the 95% CI bounds did not overlap, there was no statistically significant difference in the rate of change in incidence between men and women. In middle-SDI, low-SDI, and low-SDI countries, there was little improvement or even a slight increase in prevalence among adults aged 20 years and older. Supplementary Figures S1–S6 show the local drift of incidence rates in each country.

Figures 2A,B illustrate the temporal shifts in the age distribution of incidence. Globally, there was an incidence transition from individuals under 80 years of age to those over 80 years, with a more pronounced trend observed in countries characterized by middle SDI, high-middle SDI, or high SDI. In countries with low SDI, more than 80% of the incidence was concentrated among individuals under the age of 80. Detailed age distribution of incidence for each country is provided in Supplementary Figures S7–S18 in the Supplementary.

### The effect of age, period, and cohort on the incidence of IHD

**Figure 3:**
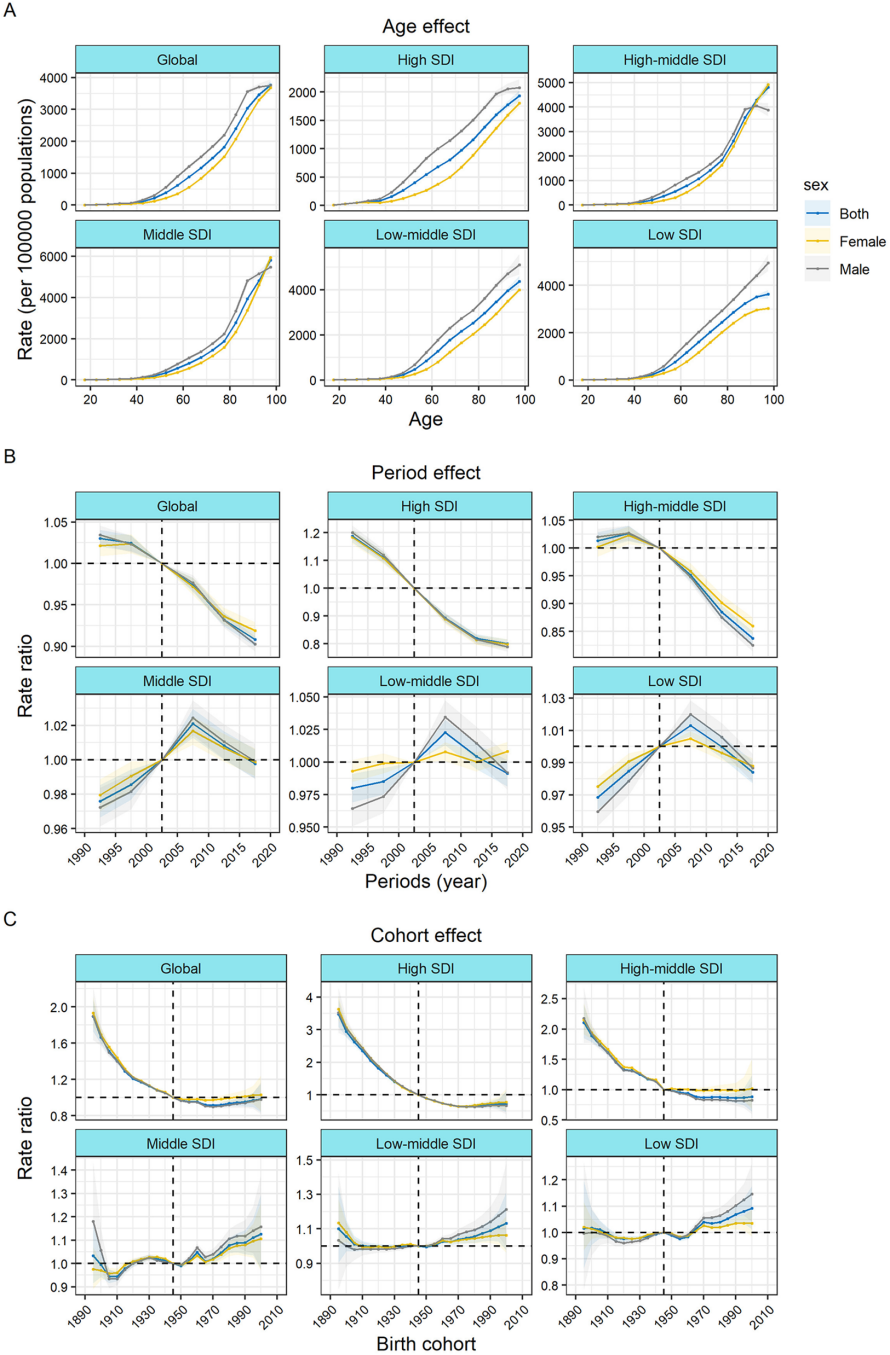
Age, period, and cohort effects on ischemic heart disease incidence by SDI quintiles **(A)** Age effects are shown by the fitted longitudinal age curves of incidence (per 100,000 person-years) adjusted for period deviations. **(B)** Period effects are shown by the relative risk of incidence (incidence rate ratio) and computed as the ratio of age-specific rates from 1990–1994 to 2015–2019 (with 2000–2005 as the referent period). **(C)** Cohort effects are shown by the relative risk of incidence and computed as the ratio of age-specific rates from the 1895 cohort to the 2000 cohort, with the referent cohort set at 1945. The dots and shaded areas denote incidence rates or rate ratios and their corresponding 95% CIs. SDI, socio-demographic Index.

**Figure 4:**
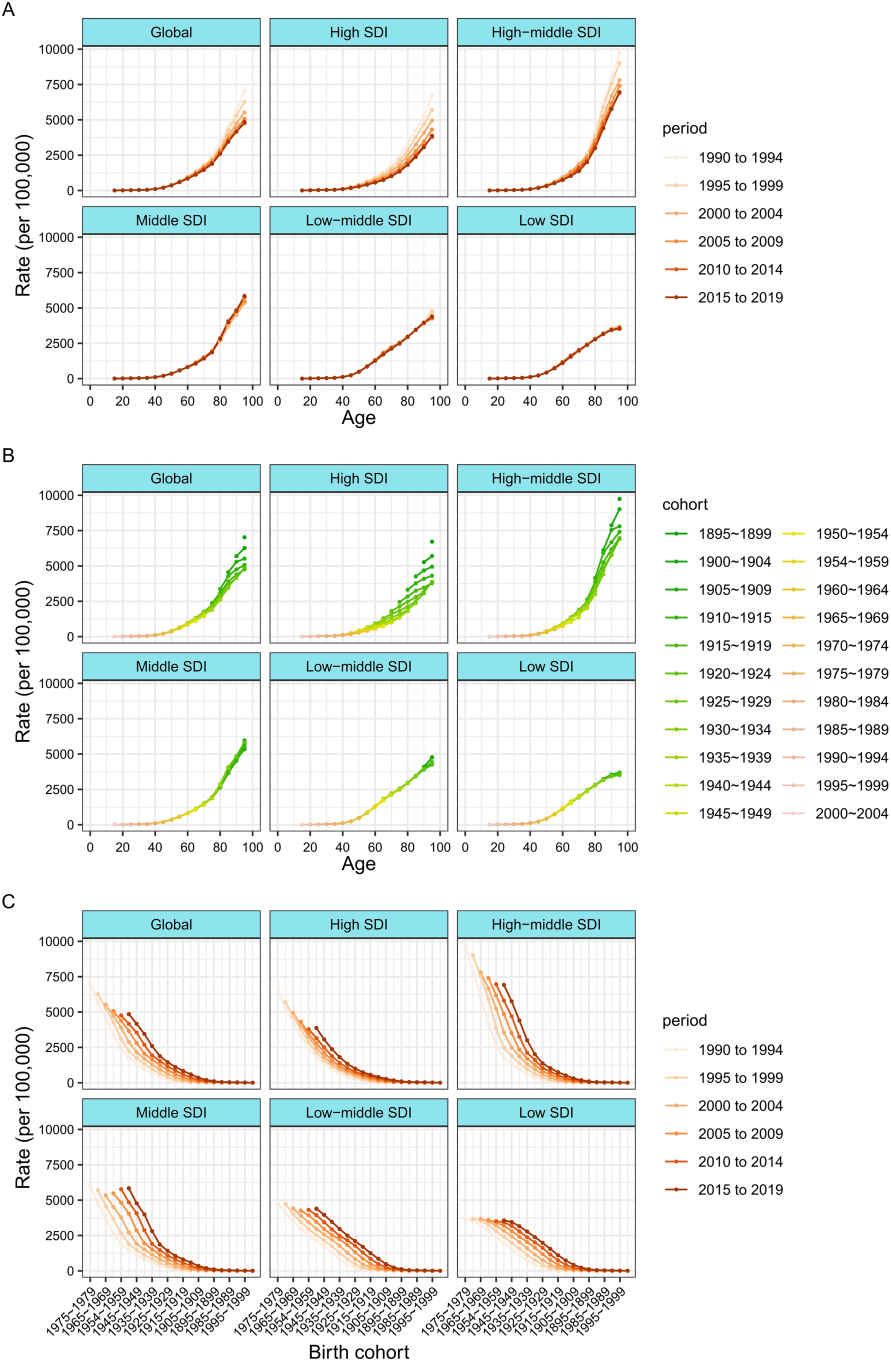
Incidence rates of ischemic heart disease across different age groups, periods, and birth cohorts during 1990–2019. **(A)** Incidence rates of ischemic heart disease across age groups by period. **(B)** Incidence rates of ischemic heart disease across age groups by birth cohorts **(C)** Incidence rates of ischemic heart disease across birth cohorts by periods.

The age-period-cohort effects, derived from the APC model, are depicted in Figure 3 according to SDI quintiles. Figure 4 shows the incidence rates of ischemic heart disease for different age groups, periods, and birth cohorts from 1990 to 2019. Longitudinal age curves represent the age effects, illustrating the typical progression of IHD incidence with age. Period effects are portrayed as the relative risk of incidence by period, serving as a tool to monitor changes in incidence over time. Cohort effects, denoted as the relative risk of incidence based on birth cohorts, serve as a metric to monitor changes in incidence for distinct birth cohorts. Generally, consistent age effect patterns were evident across diverse SDI quintiles. Specifically, the lowest risk was observed in individuals under 40 years of age, with a virtually linear increase in risk after the age of 60. Additionally, the risk tended to be relatively higher in men compared to women.

The period effects generally indicates that during the study period, the incidence rate of global IHD decreased. The incidence risk of IHD in the high and high-middle SDI quintiles was also declining. However, the incidence risk of IHD in middle SDI, low-middle SDI, and low SDI countries showed an upward trend before 2007 and only began to decline thereafter, with relatively significant gender differences.

Globally, the risk is generally decreasing. The declining cohort impacts were, like the period effects, more noticeable in high-SDI countries. The incidence rate in people born after 1895 had gradually improved in high SDI countries, while the risk in low SDI countries had hardly improved before the 1945 cohort and even continued to increase thereafter. In comparison to individuals born in the reference 1945 cohort, the relative cohort risk for those born in the 2000 cohort varied, ranging from 1.09 (95% CI 1.01–1.17) in low SDI countries to 0.72 (0.47–1.10) in high SDI countries. The influences of age, period, and cohort on the incidence of IHD in each country are visually represented in Supplementary Figures S19–S36.

## Discussion

By conducting a post-hoc analysis of 2019 GBD study data, this study examines the global landscape, long-term trends, and regional disparities in the incidence of IHD. In this study, the incidence of IHD was depicted with age-standardized rates, and APC model-derived incidence estimates were employed to assist an in-depth analysis of incidence patterns. This study found that although ASIR showed a downward pattern in all countries (with wide variation across countries and regions) and the declining trend was fastest in countries with high SDI, the burden of IHD remained high, with an increasing trend in the absolute number of global incident cases during this period. The rise in IHD incidence, influenced in part by population growth and aging (given the age-related increase in the incidence and prevalence of IHD), is better captured by age-standardized rates, which offer a more accurate reflection of the underlying trends (21, 22). The observed improvements in IHD incidence do not align with the anticipated outcomes based on a country's socioeconomic status, as evidenced by instances in Guam, China, or Uzbekistan. This suggests that disparities in the effectiveness of healthcare for this demographic may not correspond accurately with SDI. Furthermore, this increase in the burden of cardiovascular diseases (CVD) has significant consequences for the capacity and planning of health systems. Specifically, a policy centered on delivering tertiary cardiac care may not be sustainable if prevention and primary care are not also emphasized (23). In response to the unfavorable trend in ASIR of IHD, innovative challenges have been presented for controlling or eradicating this condition.

Age was shown to be associated with an elevated risk of IHD, as shown by the longitudinal age curves of the incidence rate, which showed a sharp increase with age, especially in those aged 70 or older, and were significantly greater than those under 49 years of age. Similarly, the effect of age on IHD mortality was found to grow logarithmically in another APC investigation (24). Our results add more evidence to the idea that death from IHD is strongly related to age. This phenomenon can be attributed to both population growth and rapid aging. The aging process is accompanied by a reduction in physiological reserve function and exhibits a positive correlation with heart disease mortality (25). Aging, being a non-modifiable risk factor for ischemic heart disease (IHD), is also positively linked to modifiable risk variables (24). These findings underscore the importance of prioritizing middle-aged and elderly individuals when addressing the global burden of IHD.

Over the past three decades, a comprehensive and substantial influence on potential risk factors for IHD has been exerted by demographic shifts, alterations in economic systems, modifications in social structures, environmental factors, changes in lifestyles, and advancements in medical treatments and healthcare services (26). Differences in these factors may also contribute to regional variations in ASIRs. Those with a lower SDI, like those in Africa and other LMICs, should have a greater ASIR due to their lower health literacy, lower access to medical care, more unhealthy lifestyle choices,and less education (8, 27). Although IHD was once thought of as a rather uncommon disease, it has recently risen to the number eight spot on the list of leading causes of mortality for both men and women in SSA (28). Some studies have demonstrated that low SDI regions, such as Sub-Saharan Africa (SSA), facing the dual burden of infectious and non-communicable diseases, are under significant strain and are in the early stages of an epidemiological transformation (29). Other research has indicated that in these low-SDI areas, the incidence of IHD is lower than expected. This is attributed to a scarcity of well-known risk factors for the disease, including diabetes, obesity, metabolic disorders, and ethnic susceptibility. Additionally, the artificially low case-diagnosis rates and misclassification of potential causes of death are influenced by inadequate healthcare systems and a shortage of cardiac professionals (30).

High-income North America and Western Europe of the 21 regions, had the fastest declines in the incidence of IHD. However, in the Western European countries studied, the declining trend in age-standardized IHD incidence has slowed considerably over the past 30 years and has even flattened in some countries (14). One plausible explanation is the demographic shifts observed in most Western European countries, driven by a rise in immigration from non-European low-income countries in recent years. Numerous studies have indicated that, in comparison to native-born individuals, immigrants exhibit higher risk factors and incidence of ischemic heart disease (IHD), primarily associated with low socioeconomic status. It can be asserted that these demographic changes in Western European countries might have contributed to impeding the decline in IHD incidence (31, 32). The United States, a high-income North American country, has seen a significant decline in incidence due to IHD between 1999 and 2019, although the rate of decline has slowed since 2010.The effect of the cohort effect on the incidence of IHD showed a significant downward trend before 1970, after which it showed a relatively low and stable state. The age-standardized incidence and overall mortality levels of IHD showed a rapid downward trend. This may be attributable to the United States’ adoption of effective cardiovascular disease (CVD) preventative strategies, the strengthening of the healthcare system, and the use of innovative drugs, etc. (11, 21).

The rising demand for IHD care in low-income nations is not being met by current worldwide initiatives. Despite substantial advancements in the prevention and treatment of IHD, particularly in regions characterized by relatively high Socio-demographic Index (SDI), a significant proportion of the global disease burden is still attributable to IHD. Given the predominantly preventable and treatable nature of IHD, there exists a pressing need for innovative strategies to implement cost-effective therapies and target modifiable risk factors, particularly in regions experiencing a high or increasing burden (33).

India, with a population of 1.39 billion, stands as the second-most populous country globally. Despite an 85% decline in the ASIR for IHD between 1990 and 2019, it continues to contribute the highest absolute number of IHD event cases worldwide. The increased incidence appears to have been driven by unfavorable cohort effects in recent years, with a clear upward trend in incidence for those born after 1950. In India, over half of the cardiovascular disease deaths in 2016 occurred in individuals under the age of 70. The rise in age-standardized prevalence of ischemic heart disease (IHD) since 1990 has been more pronounced in states with low epidemiological transition level (ETL) and in the lower-middle ETL state groups. These groups encompass many less developed states, constituting 55% of India's population. Simultaneously, there exists a paradoxical and uneven distribution of cardiac facilities, with a concentration in states characterized by a low burden of coronary heart disease (CHD) (7). The significant gap in healthcare quality between the public and private sectors, coupled with financial constraints, emerges as a crucial factor contributing to this scenario (7). This underscores the imperative for prompt policy and health system responses tailored to the specific circumstances in each state. A particular challenge in many parts of India is that population-based data on the incidence of CVD and its sequelae are often not available, although some ongoing studies are attempting to address this issue (7). In India, there is a requirement for robust population-level disease registration and surveillance systems to effectively monitor trends in the incidence of major cardiovascular diseases (CVD) and their associated risk factors. While there have been great strides made in the ability to prevent and treat CVD on a global scale, there is still much work to be done in India as a whole, especially in the less developed states where the rate of increase in CVD burden is among the greatest in the country (7).

Our research revealed that period effects on IHD incidence in China exhibited an upward trajectory over time, followed by a decline after 2012. This shift can be attributed to advancements in medical treatment, extended life expectancy, and an increase in lifestyle and metabolic risk factors among the younger population. There has been rapid economic development in China since the 1990s, and with it has come widespread adoption of Western dietary habits, tobacco use, alcohol consumption, and a decline in physical activity (34). Before 1950, there was an upward trend in cohort effects on ischemic heart disease (IHD) incidence. This could be attributed to the potential impacts of World War II (1939–1945) and the Great Chinese Famine, which might have contributed to a temporary surge in the cohort effect during the period of 1959–1961. Premature exposure to environmental stressors could elevate the incidence of post-traumatic stress disorder. Furthermore, individuals who have undergone severe wartime trauma may exhibit an excess of cardiovascular disease risk factors, potentially leading to profound fatigue (35, 36). The “open door” policy adopted by China in 1978 marked the beginning of a fast economic boom. The quality of life has risen dramatically since then. Nevertheless, an increasing number of individuals are experiencing psychological stress, encompassing factors such as work-related stress, educational stress, housing-related stress, and similar concerns. Since 1970, the cohort effects on the incidence of IHD in women have been found to be somewhat greater than those in men. Possible contributing factors include the association of family conflicts, depression, anxiety, and obligations with an elevated risk of cardiovascular disease. Moreover, these issues tend to be more prevalent in women than in men (37, 38). Despite the implementation of crucial strategies, effective prevention and control programs in cardiovascular rehabilitation and tobacco control, and extensive promotion of balanced diets and healthy lifestyles by national leadership, achieving the goals outlined in “Healthy China 2030” and the “13th Five-Year Public Health and Health Care Plan” is expected to be challenging. This difficulty is attributed to the persistent threat of IHD growth (39).

### Strengths and limitations

This research compared the rates of decreasing IHD incidence over a thirty-year period. Meanwhile, our research demonstrates how GBD data may be analyzed in depth to reveal trends in disease. Employing APC models to evaluate disease trends concerning age, time, and cohorts can provide insights into the effectiveness of health system responses beyond traditional epidemiological measures. This approach facilitates a more accessible monitoring of country-specific advancements toward achieving the Sustainable Development Goals (SDG) targets related to various non-communicable diseases (NCDs).

Our research has a number of limitations. First, socio-cultural and ethnic variations were not taken into consideration in the GBD Research models. These disparities were frequently associated with health behaviors and risk factors that impact the overall burden of IHD. Second, the quality of the data is another constraint of the current study, as it was not always possible to collect participants between 1990 and 2019 in a thorough and comparable manner. Even if a number of modeling tools are employed to standardize the data, it is still possible that there is missing information for some countries. Patients are more likely to be misclassified as having IHD if they have other cardiovascular (CV) problems at the same time. Collection of surveillance data on ischemic heart disease (IHD) across all Global Burden of Disease (GBD) regions is recommended, utilizing standardized case definitions and measurement methods. It is advisable to avoid relying on methodologies designed for estimating missing data and adjusting for measurement variations between source studies. Notably, the estimates considered data from certain countries, the majority of which lacked raw data and involved the incorporation of parameters and models. Despite these considerations, the GBD provided a reasonable global comparative foundation.

## Conclusions and implications

IHD is an important contributor to the remaining worldwide mortality due to NCDs. Our age-period-cohort analysis of global IHD incidence revealed that incidence trends were declining globally, but the incidence, or the absolute number of IHD occurrences, remained high and caused a considerable illness burden. The burden of IHD varies across SDI regions, and the increase in incidence over the past 30 years has not always been proportional to a country's socioeconomic development. Many countries who have the financial means to provide improved health care for the IHD population are unable to do so because of the unfavorable period and cohort effects. Controlling IHD risks and reducing health inequalities between countries with varying levels of development requires learning from what has worked well in the past. These results support the necessity of establishing stronger, effective, and evidence-based primary and secondary prevention interventions. This also emphasizes the importance of conducting IHD monitoring studies of high quality and standardization. Especially in middle- and low-income regions, it is crucial to fund and create new surveillance investigations.

## Data Availability

The original contributions presented in the study are included in the article/Supplementary Material, further inquiries can be directed to the corresponding authors.
