# Supplementary material for: Global, regional, and national time trends in ischaemic heart disease incidence over three decades (1990–2019): an age-period-cohort analysis of the global burden of disease study 2019
Source: Front Cardiovasc Med. 2024 Nov 1;11:1396380. doi: 10.3389/fcvm.2024.1396380 (PMC11563781; doi:10.3389/fcvm.2024.1396380)
Supplement: Supplementary file 1 [file Datasheet1.docx]

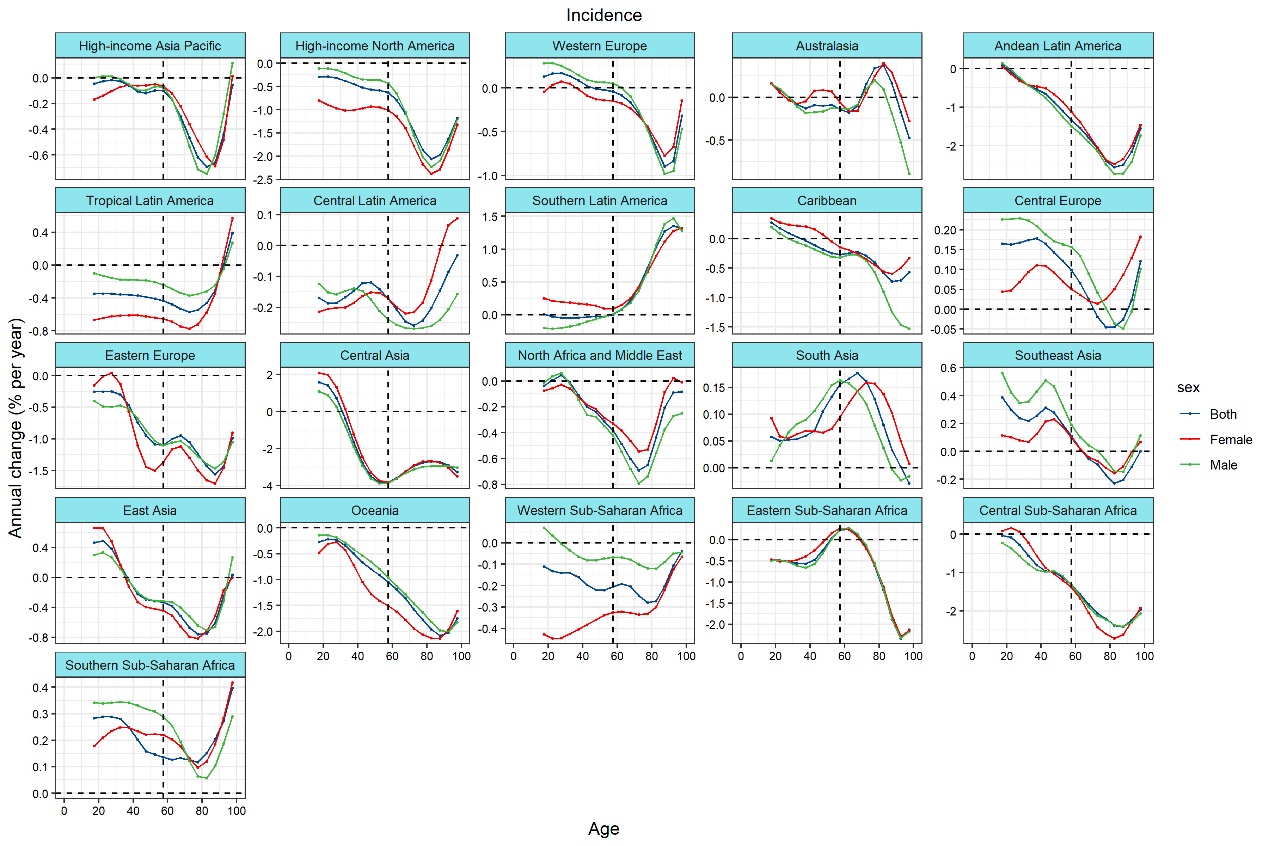


Figure S1. Ischemic heart disease incidence local drifts in all age groups in global, 1990-2019. Local drifts of ischemic heart disease incidence (estimates from age-period-cohort models) for 17 age groups (15–19 to 95+ years), 1990–2019. The dots and shaded areas indicate the annual percentage change of incidence (% per year) and the corresponding 95% CIs.


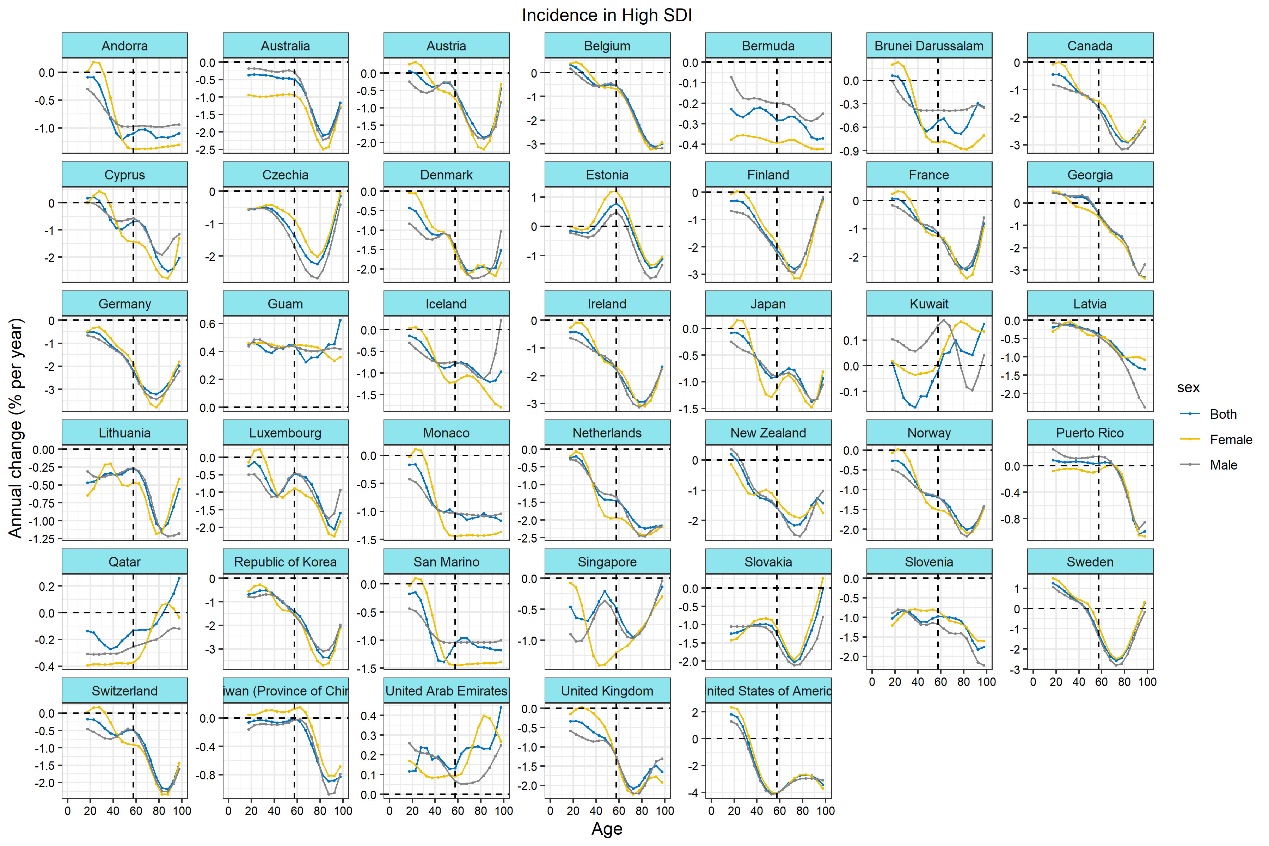


Figure S2. Ischemic heart disease incidence local drifts in all age groups in high-SDI quintiles, 1990-2019. Local drifts of ischemic heart disease incidence (estimates from age-period-cohort models) for 17 age groups (15–19 to 95+ years), 1990–2019. The dots and shaded areas indicate the annual percentage change of incidence (% per year) and the corresponding 95% CIs.


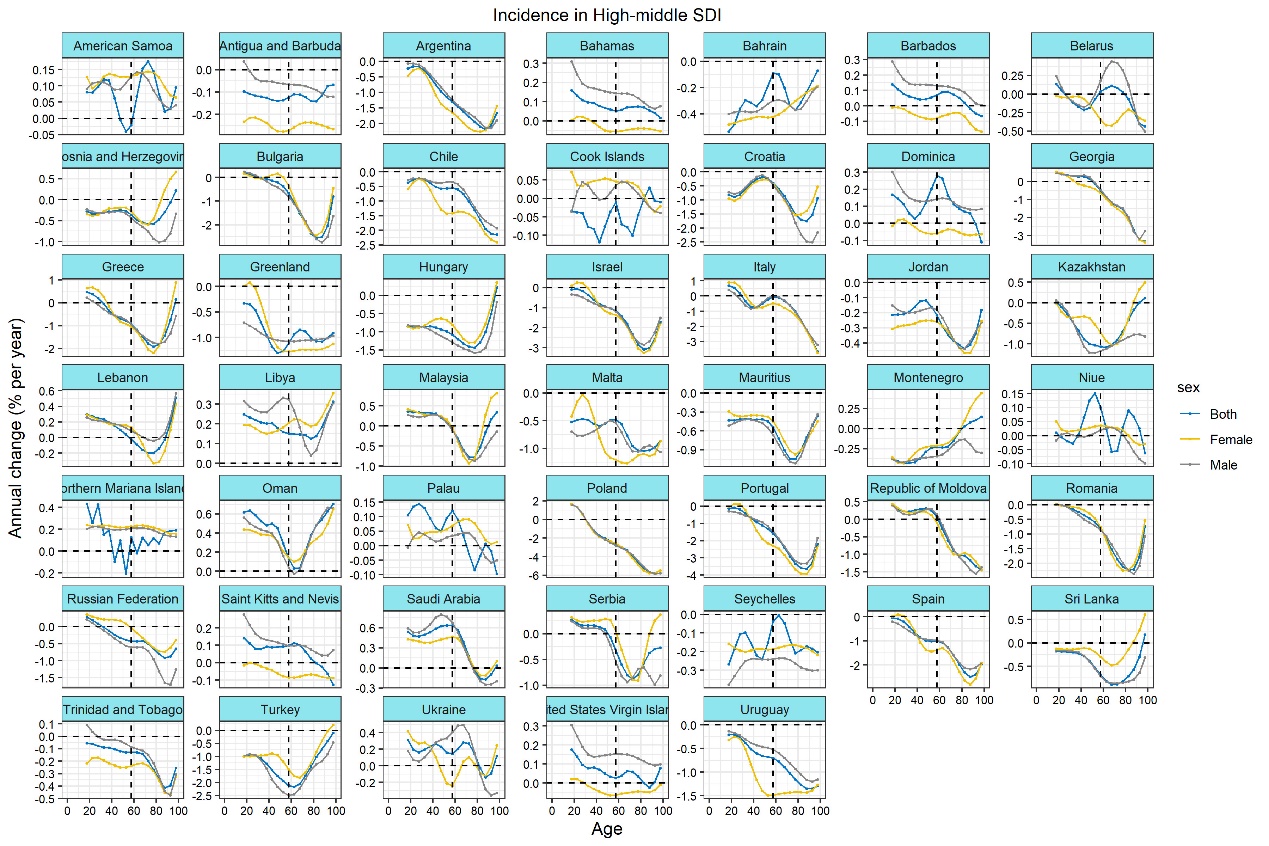


Figure S3. Ischemic heart disease incidence local drifts in all age groups in high-middle SDI quintiles, 1990-2019. Local drifts of ischemic heart disease incidence (estimates from age-period-cohort models) for 17 age groups (15–19 to 95+ years), 1990–2019. The dots and shaded areas indicate the annual percentage change of incidence (% per year) and the corresponding 95% CIs.


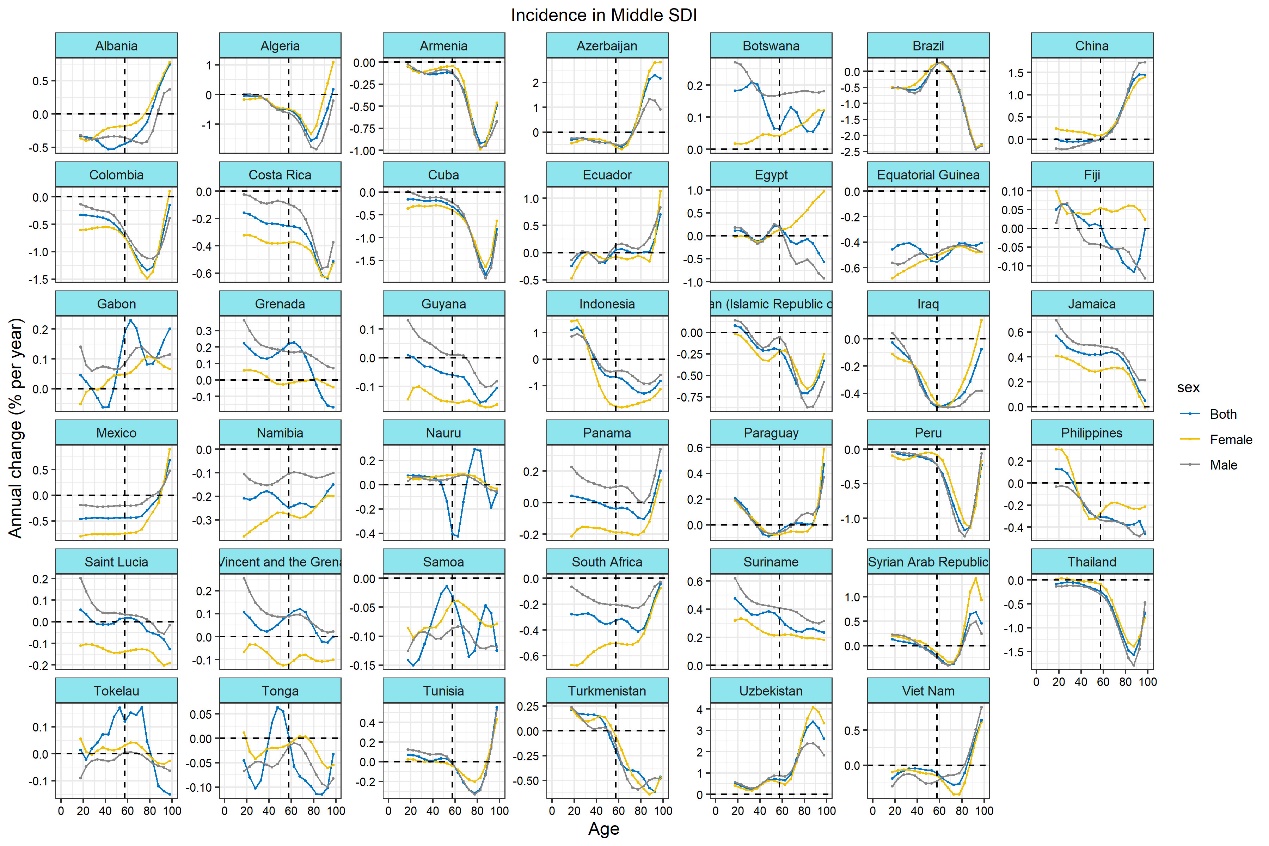


Figure S4. Ischemic heart disease incidence local drifts in all age groups in middle-SDI quintiles, 1990-2019. Local drifts of ischemic heart disease incidence (estimates from age-period-cohort models) for 17 age groups (15–19 to 95+ years), 1990–2019. The dots and shaded areas indicate the annual percentage change of incidence (% per year) and the corresponding 95% CIs.


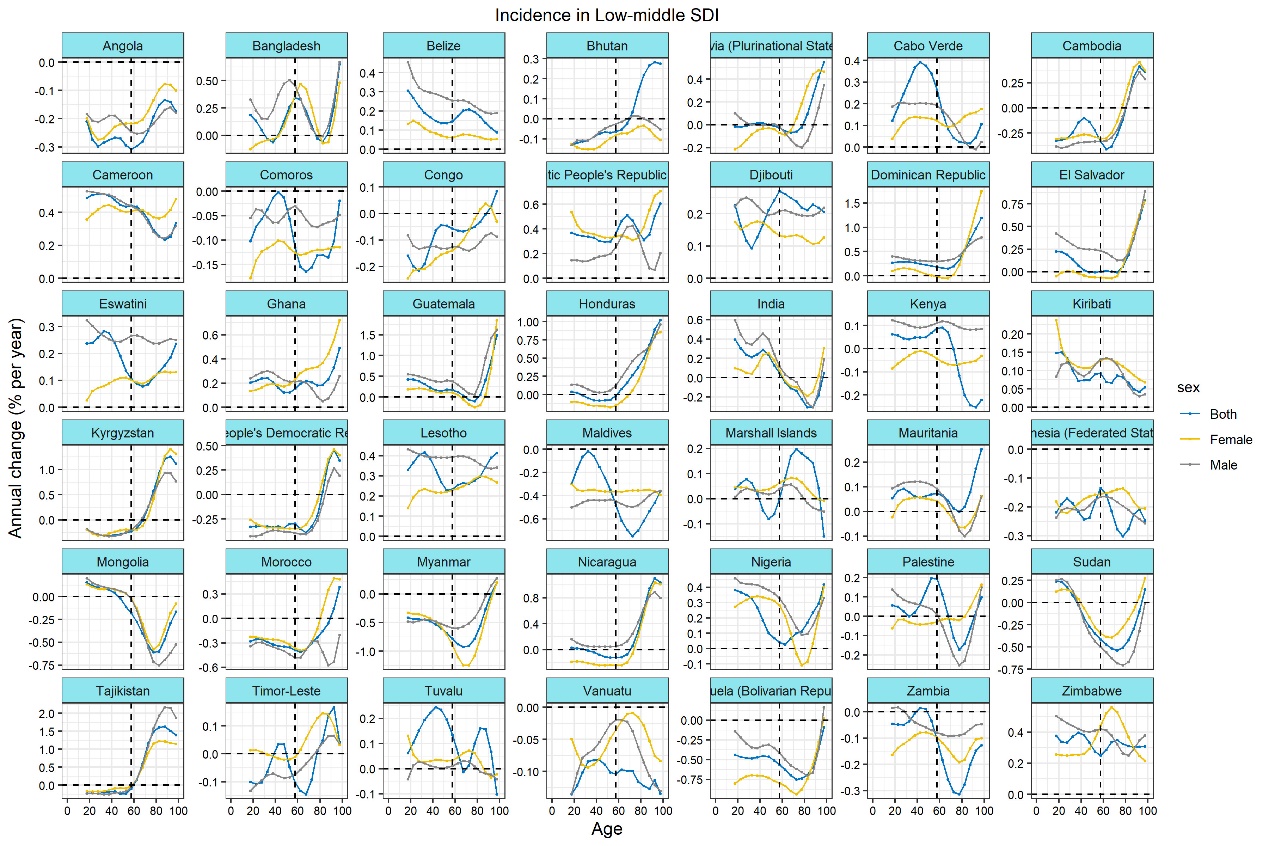


Figure S5. Ischemic heart disease incidence local drifts in all age groups in low-middle SDI quintiles, 1990-2019. Local drifts of ischemic heart disease incidence (estimates from age-period-cohort models) for 17 age groups (15–19 to 95+ years), 1990–2019. The dots and shaded areas indicate the annual percentage change of incidence (% per year) and the corresponding 95% CIs.


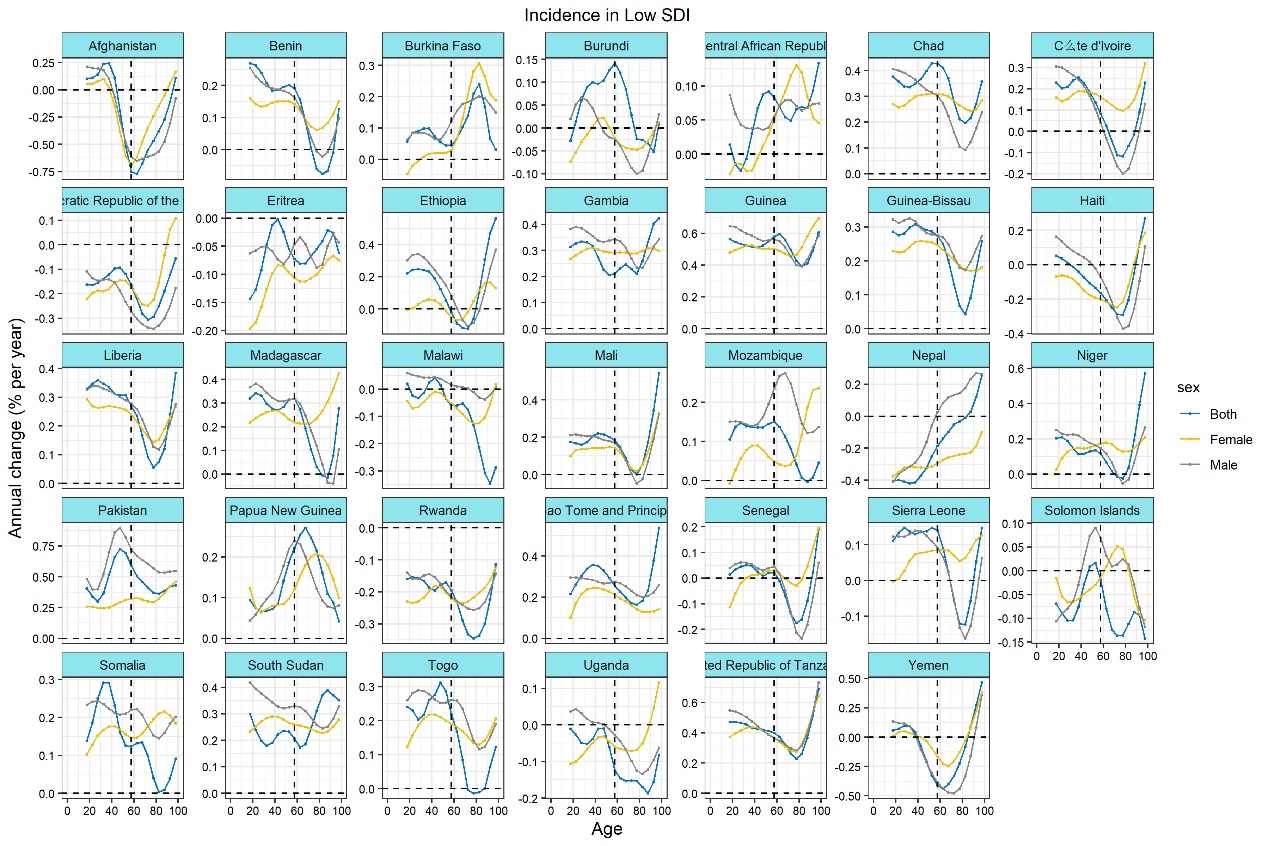


Figure S6. Ischemic heart disease incidence local drifts in all age groups in low-SDI quintiles, 1990-2019. Local drifts of ischemic heart disease incidence (estimates from age-period-cohort models) for 17 age groups (15–19 to 95+ years), 1990–2019. The dots and shaded areas indicate the annual percentage change of incidence (% per year) and the corresponding 95% CIs.


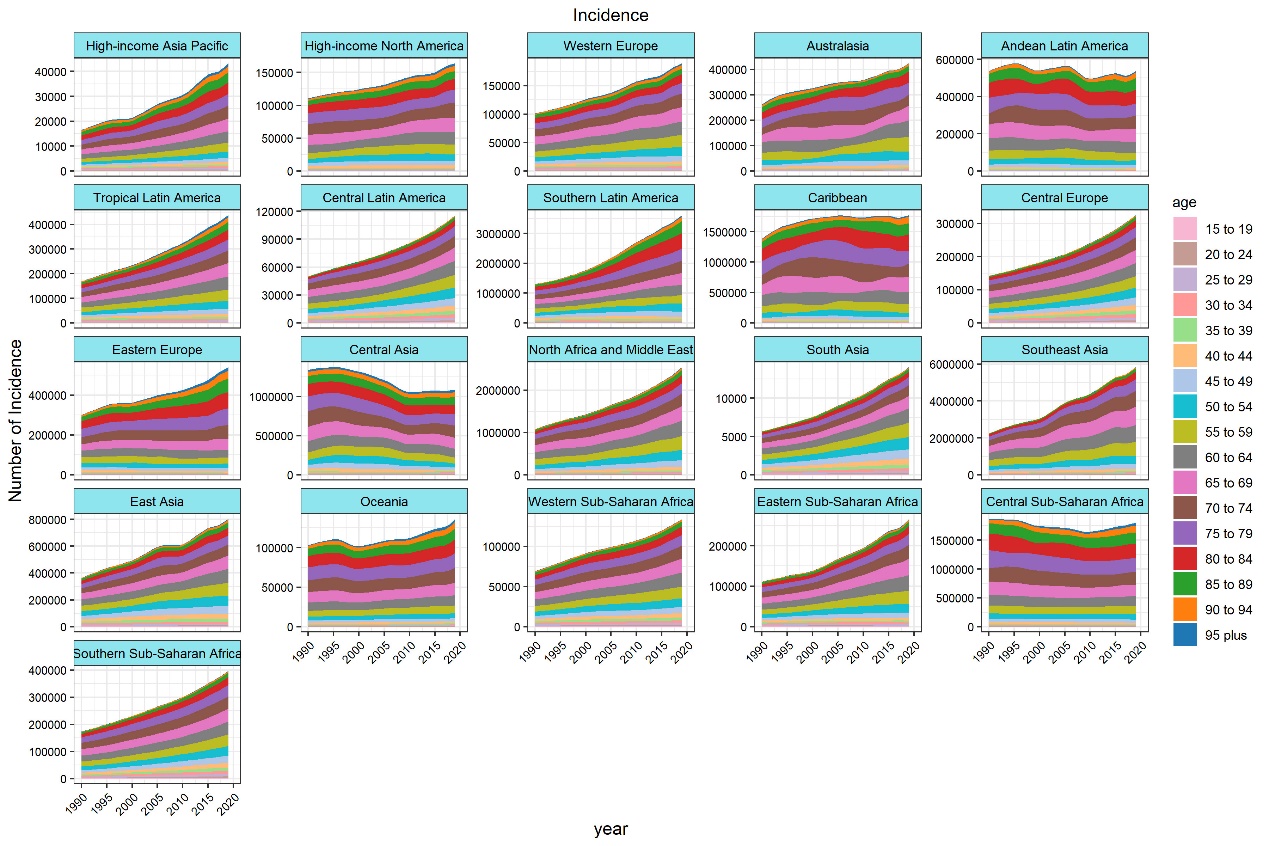


Figure S7. Age distribution of absolute cases of ischemic heart disease incidence in global, 1990-2019.


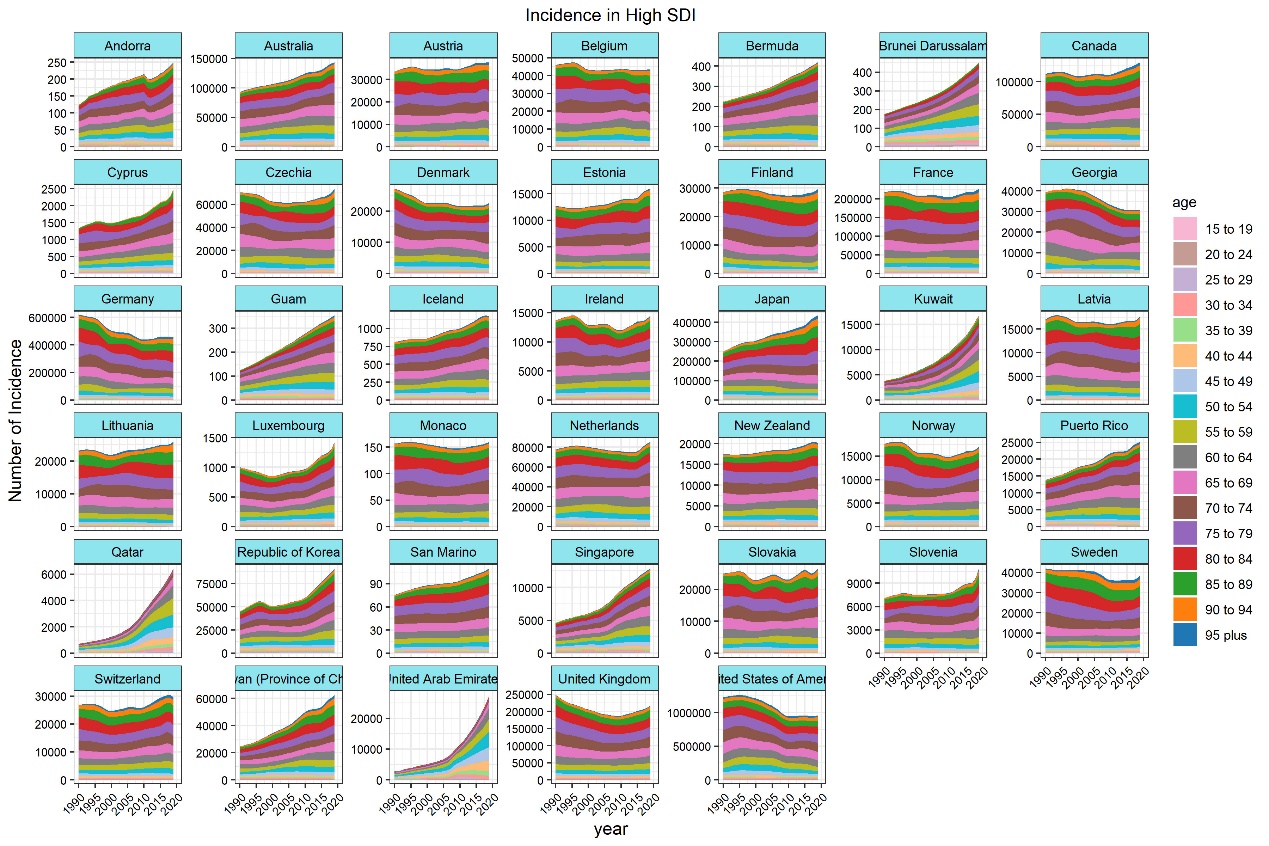


Figure S8. Age distribution of absolute cases of ischemic heart disease incidence in high-SDI countries, 1990-2019.


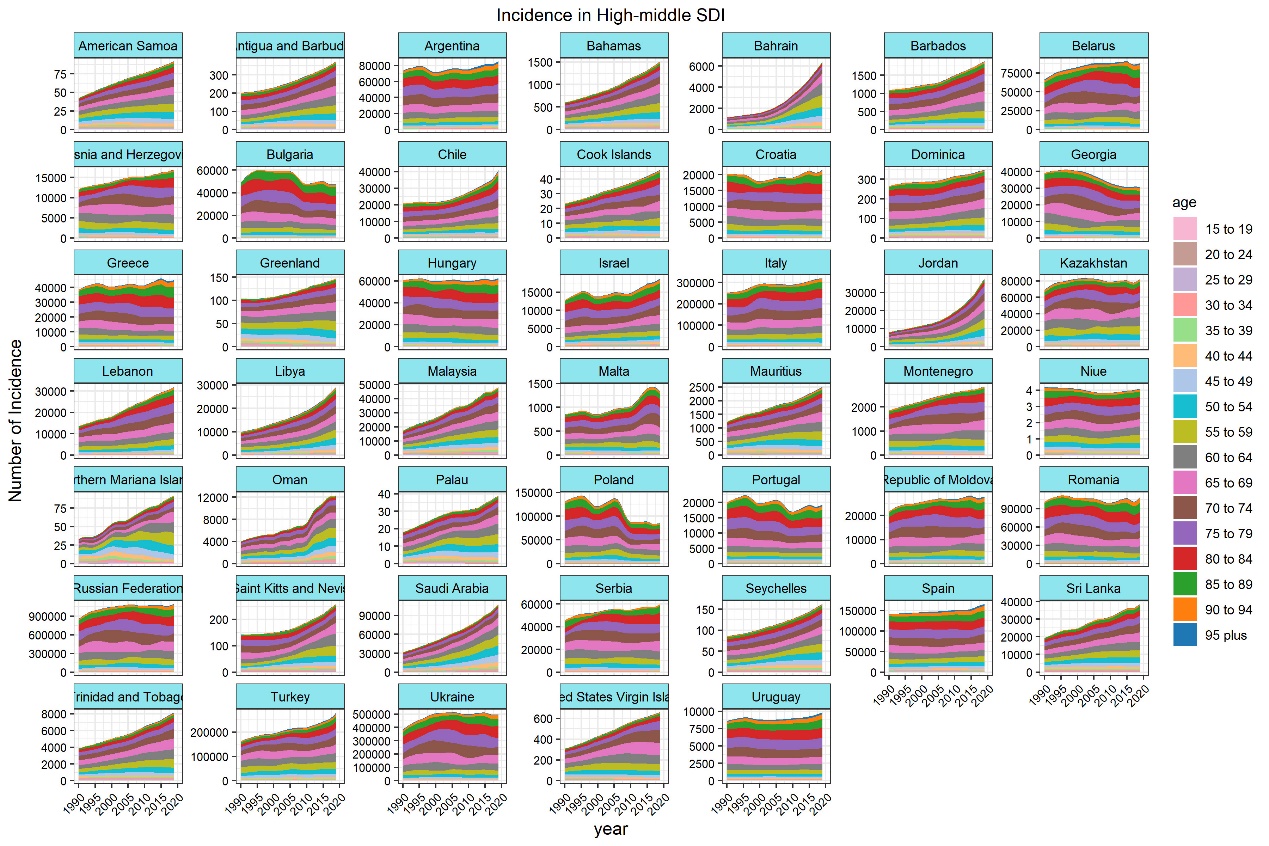


Figure S9. Age distribution of absolute cases of ischemic heart disease incidence in high-middle SDI countries, 1990-2019.


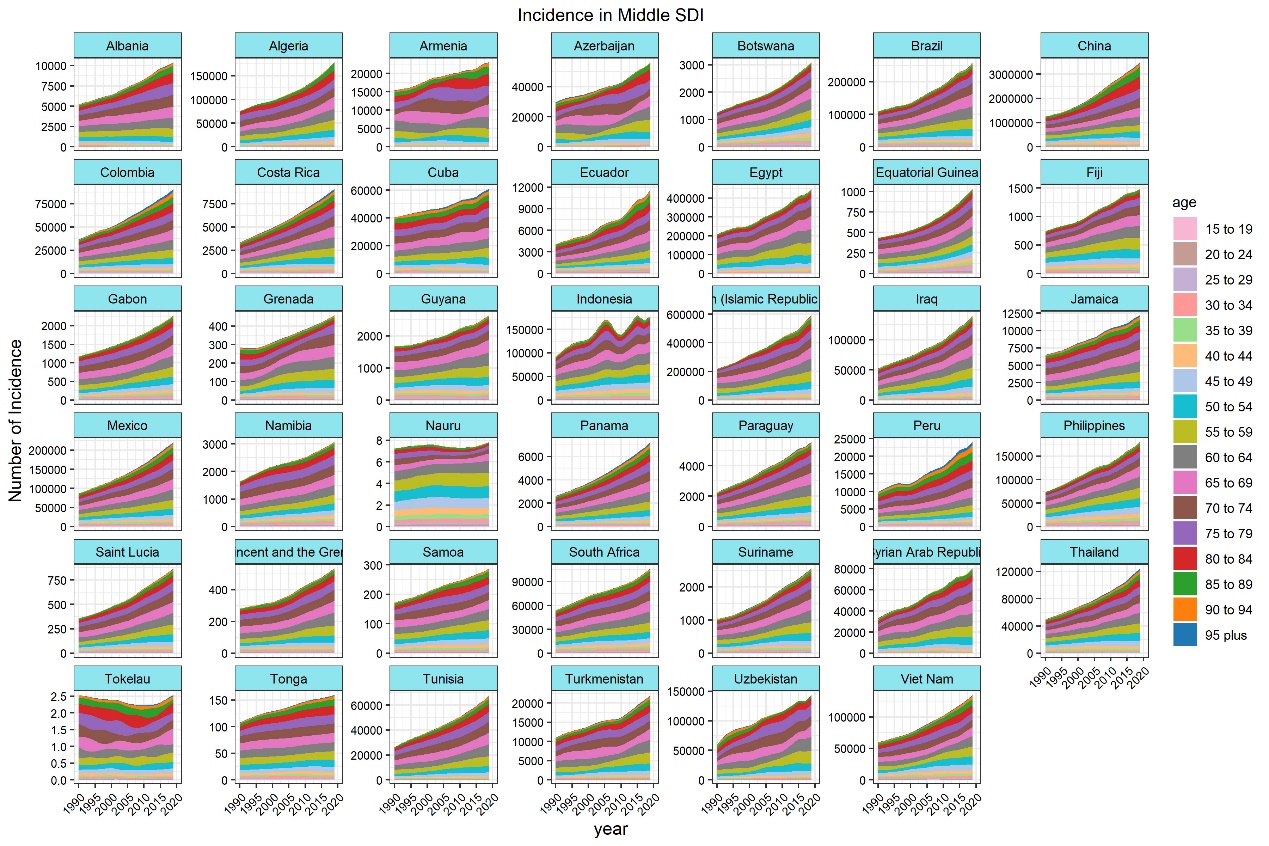


Figure S10. Age distribution of absolute cases of ischemic heart disease incidence in middle-SDI countries, 1990-2019.


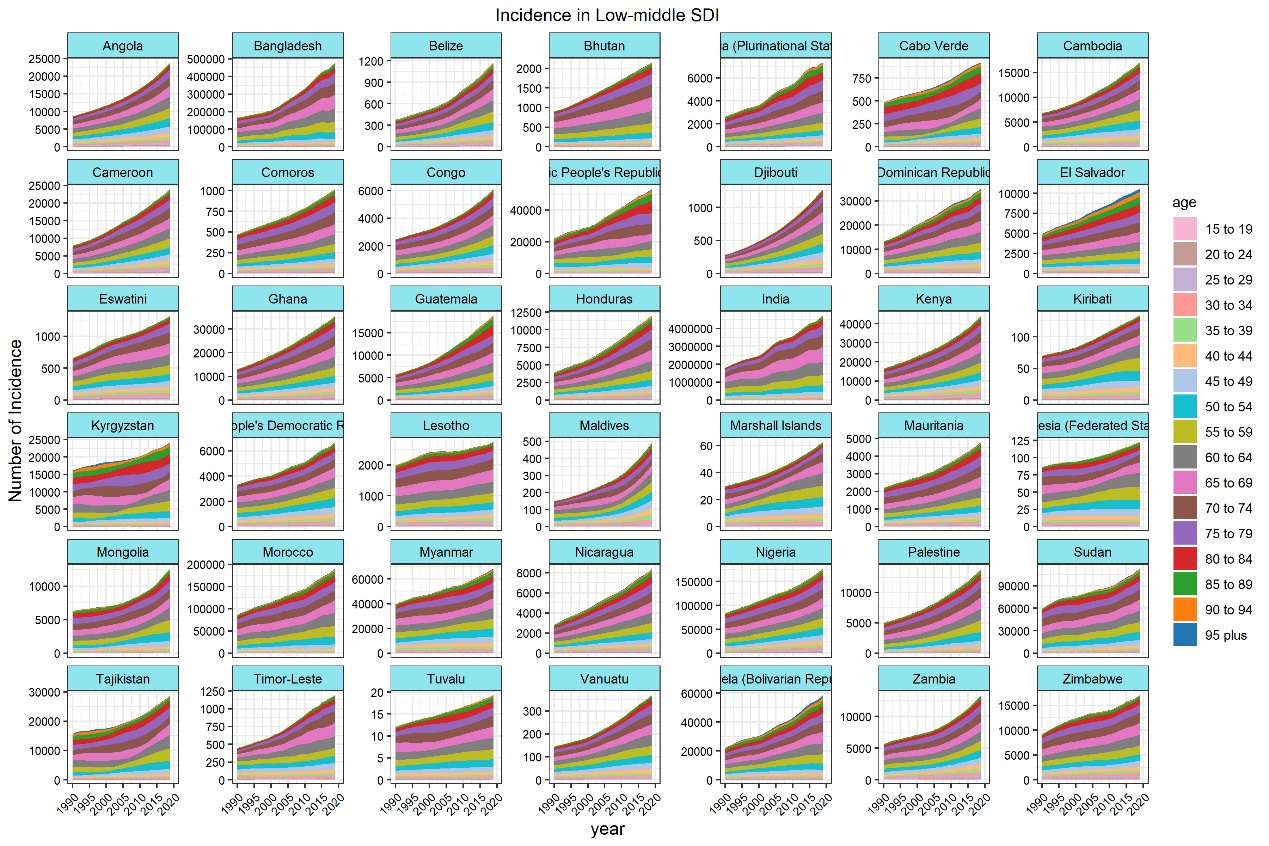


Figure S11. Age distribution of absolute cases of ischemic heart disease incidence in low-middle SDI countries, 1990-2019.


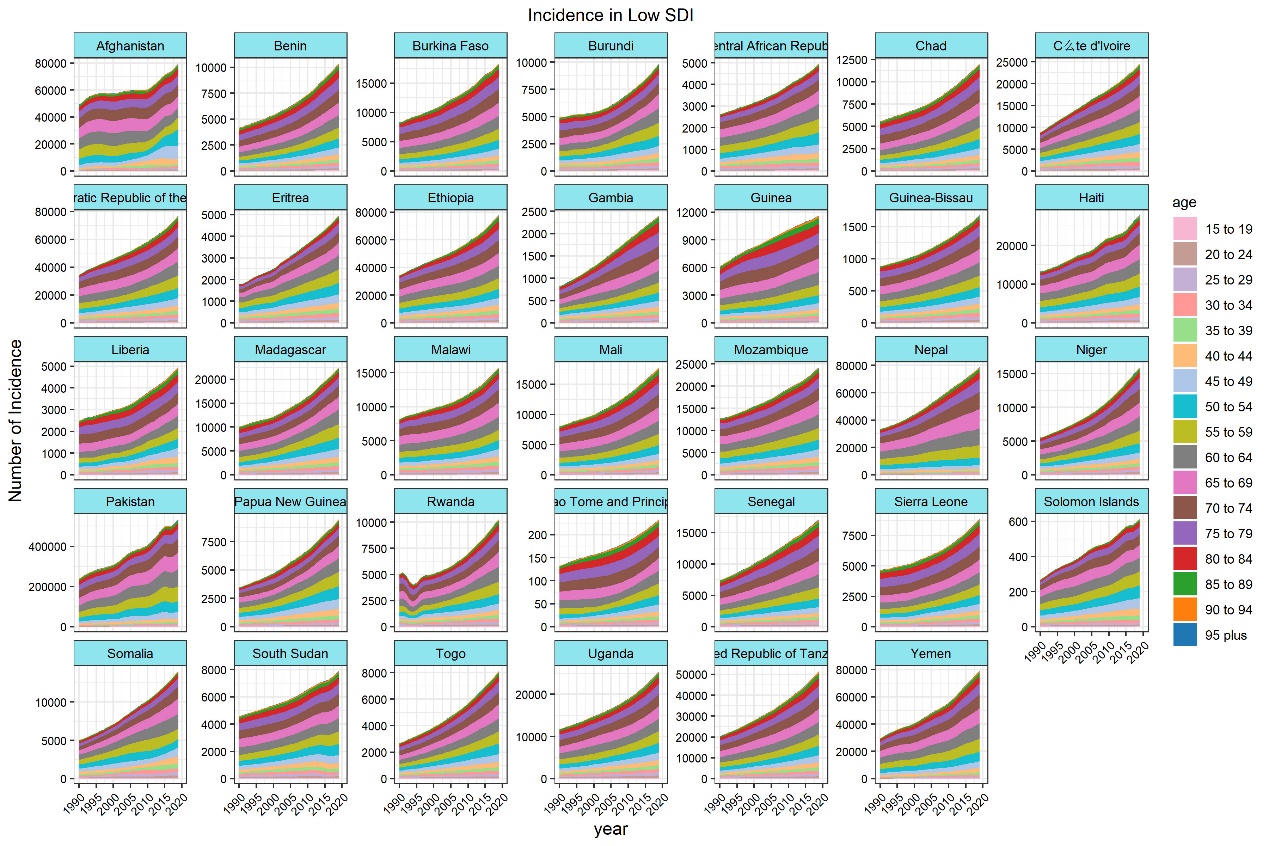


Figure S12. Age distribution of absolute cases of ischemic heart disease incidence in low-SDI countries, 1990-2019.


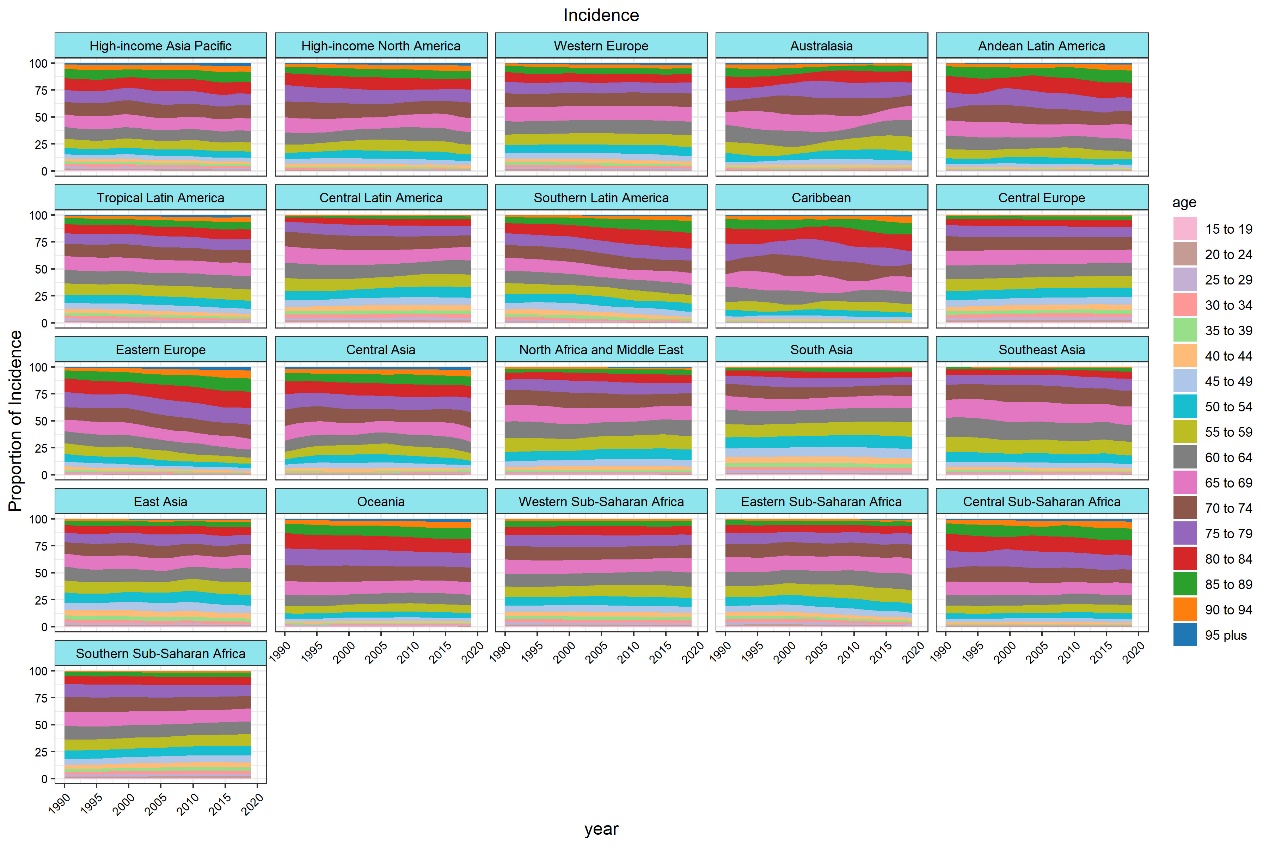


Figure S13. Age distribution of the relative proportion of ischemic heart disease incidence in global, 1990–2019.


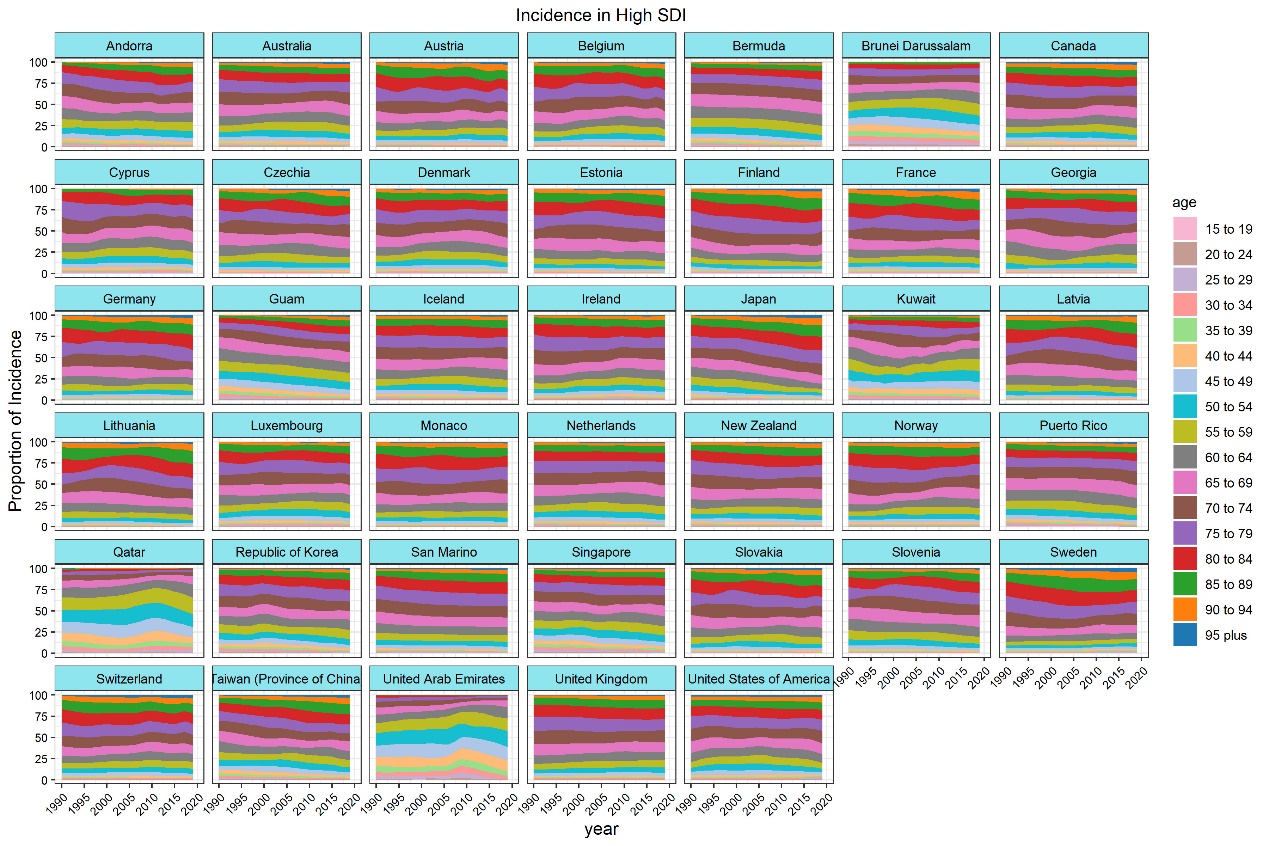


Figure S14. Age distribution of the relative proportion of ischemic heart disease incidence in high-SDI quintiles, 1990–2019.


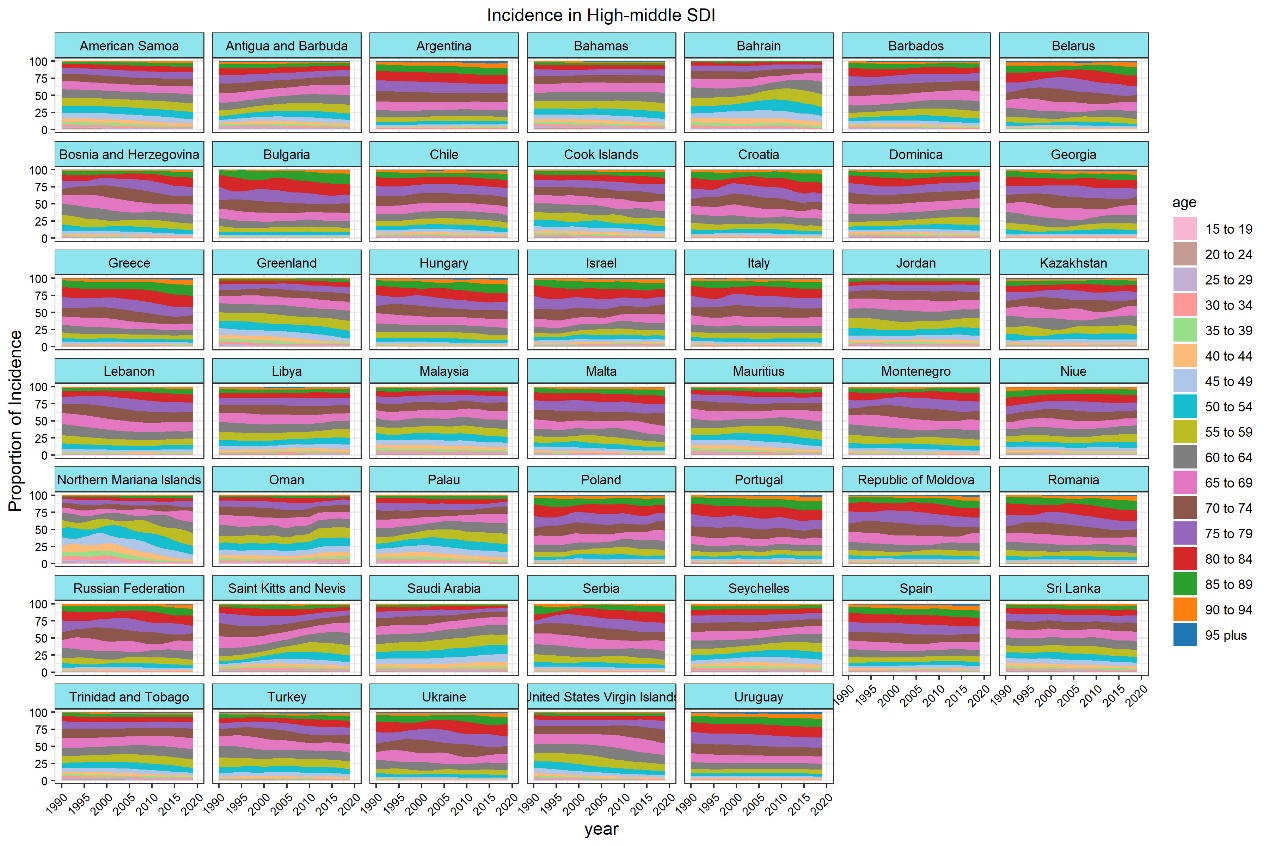


Figure S15. Age distribution of the relative proportion of ischemic heart disease incidence in high-middle SDI quintiles, 1990–2019.


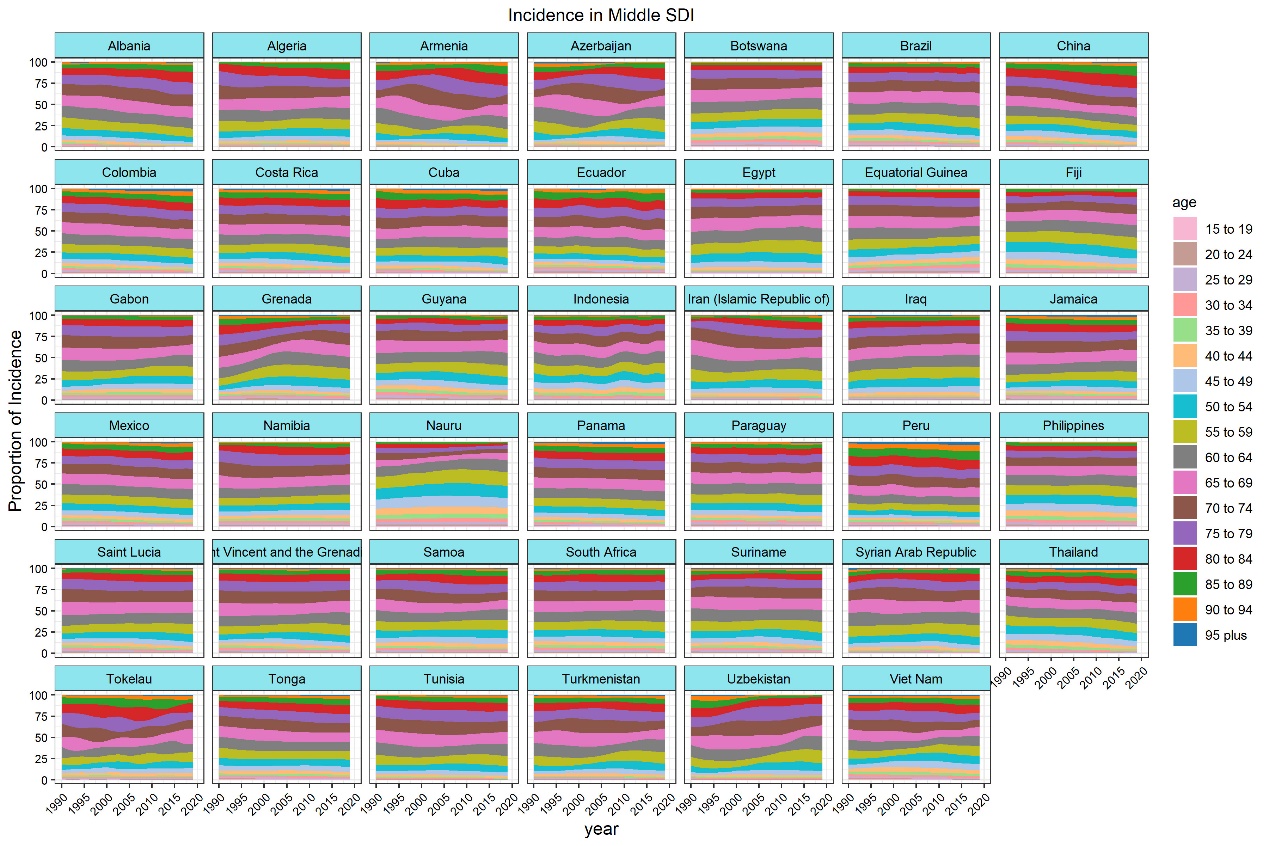


Figure S16. Age distribution of the relative proportion of ischemic heart disease incidence in middle-SDI quintiles, 1990–2019.


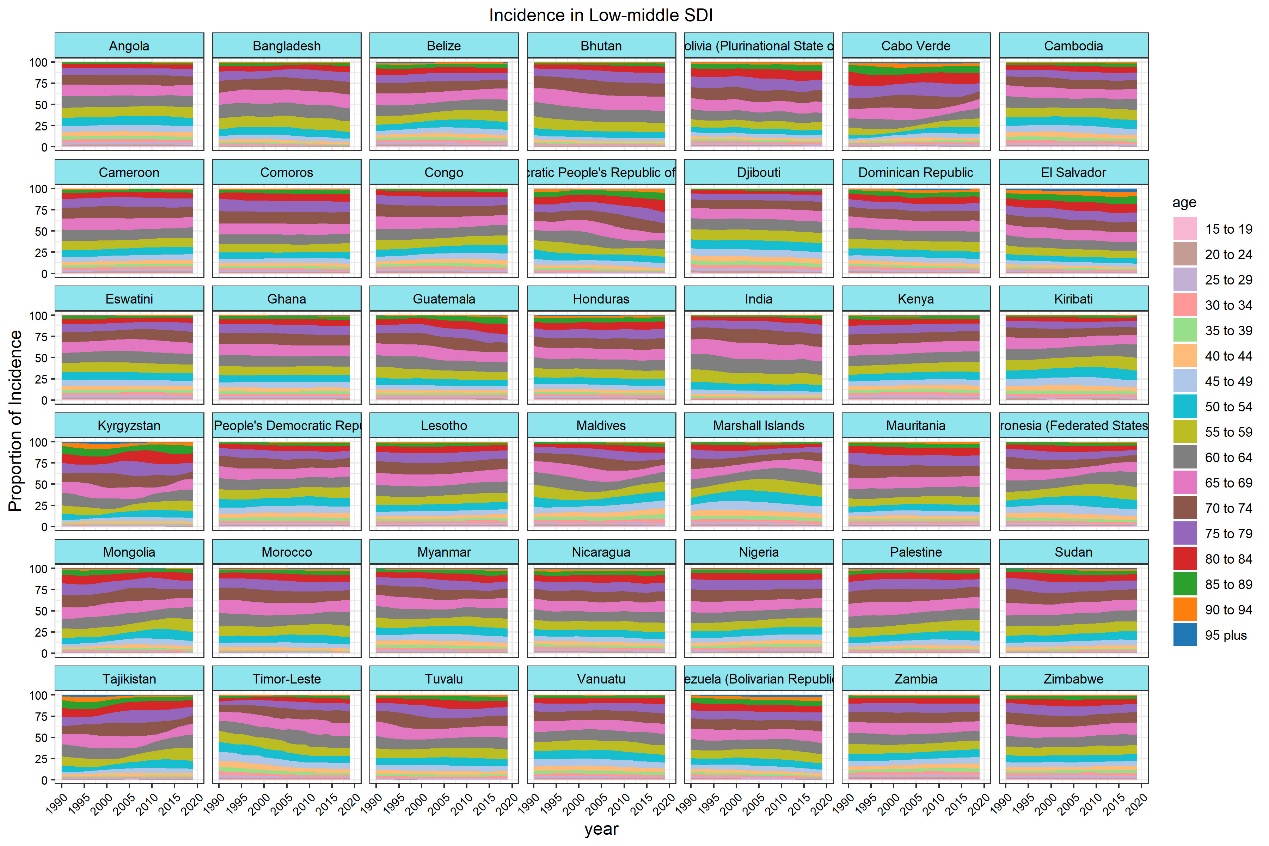


Figure S17. Age distribution of the relative proportion of ischemic heart disease incidence in low-middle SDI quintiles, 1990–2019.


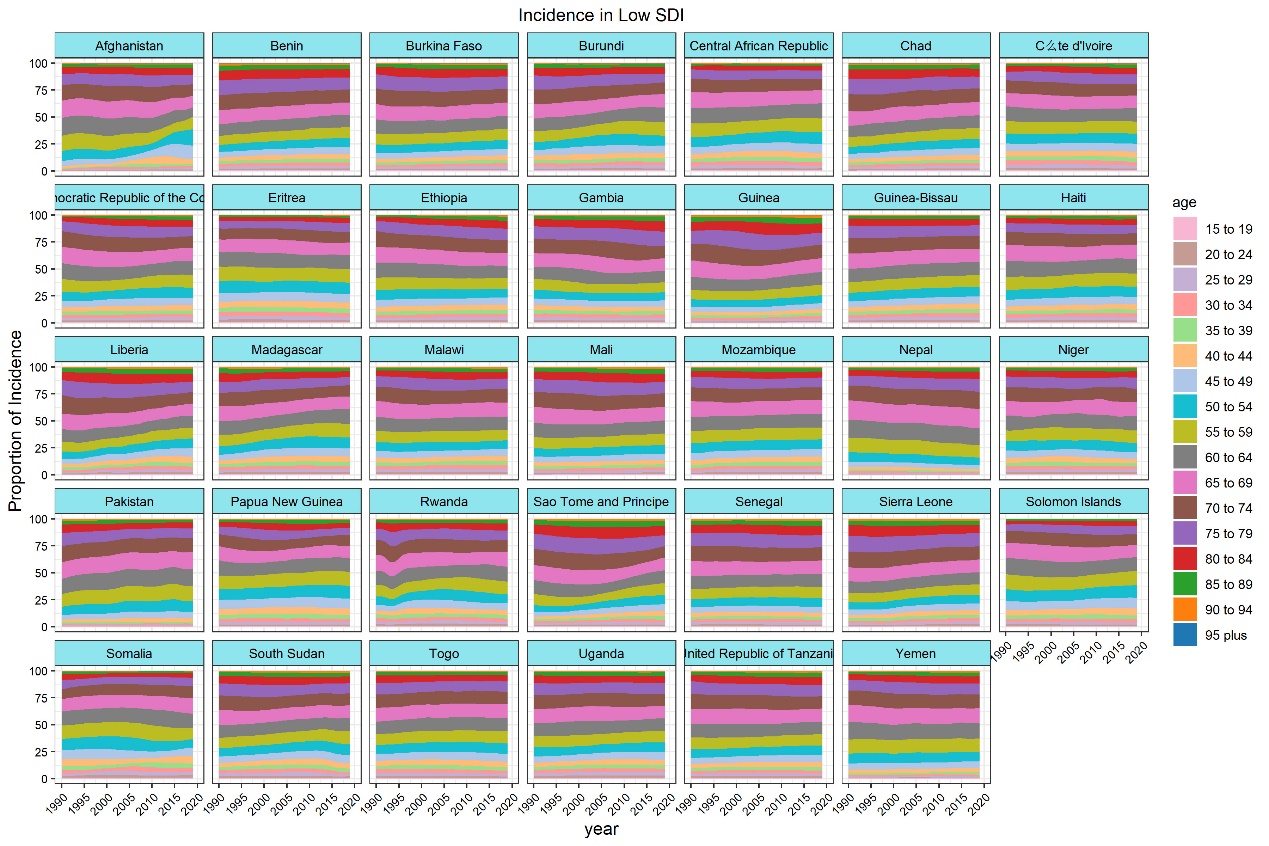


Figure S18. Age distribution of the relative proportion of ischemic heart disease incidence in low-SDI quintiles, 1990–2019.


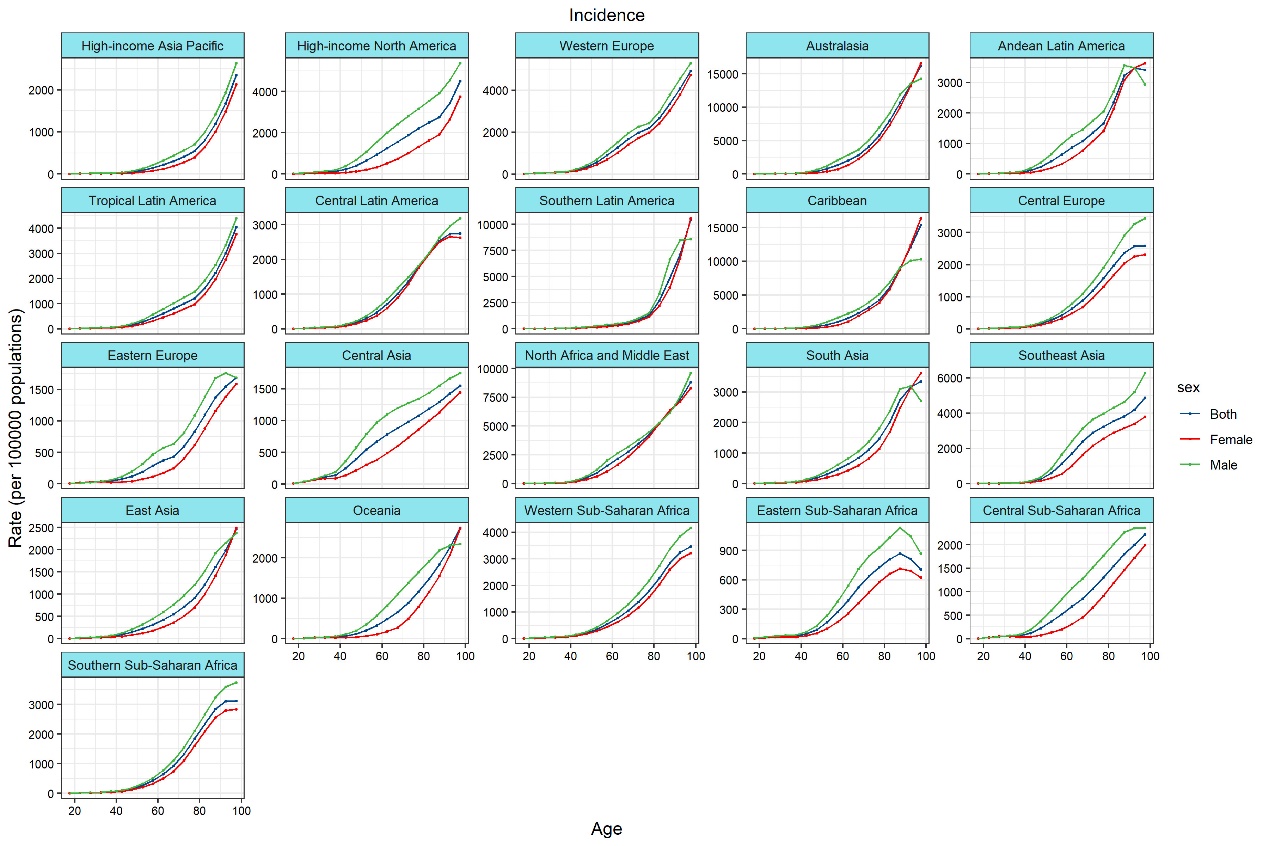


Figure S19. Age effects on incidence of ischemic heart disease in global, 1990–2019. Age effects are shown by the fitted longitudinal age curves of incidence (per 100,000 person-years) adjusted for period deviations.


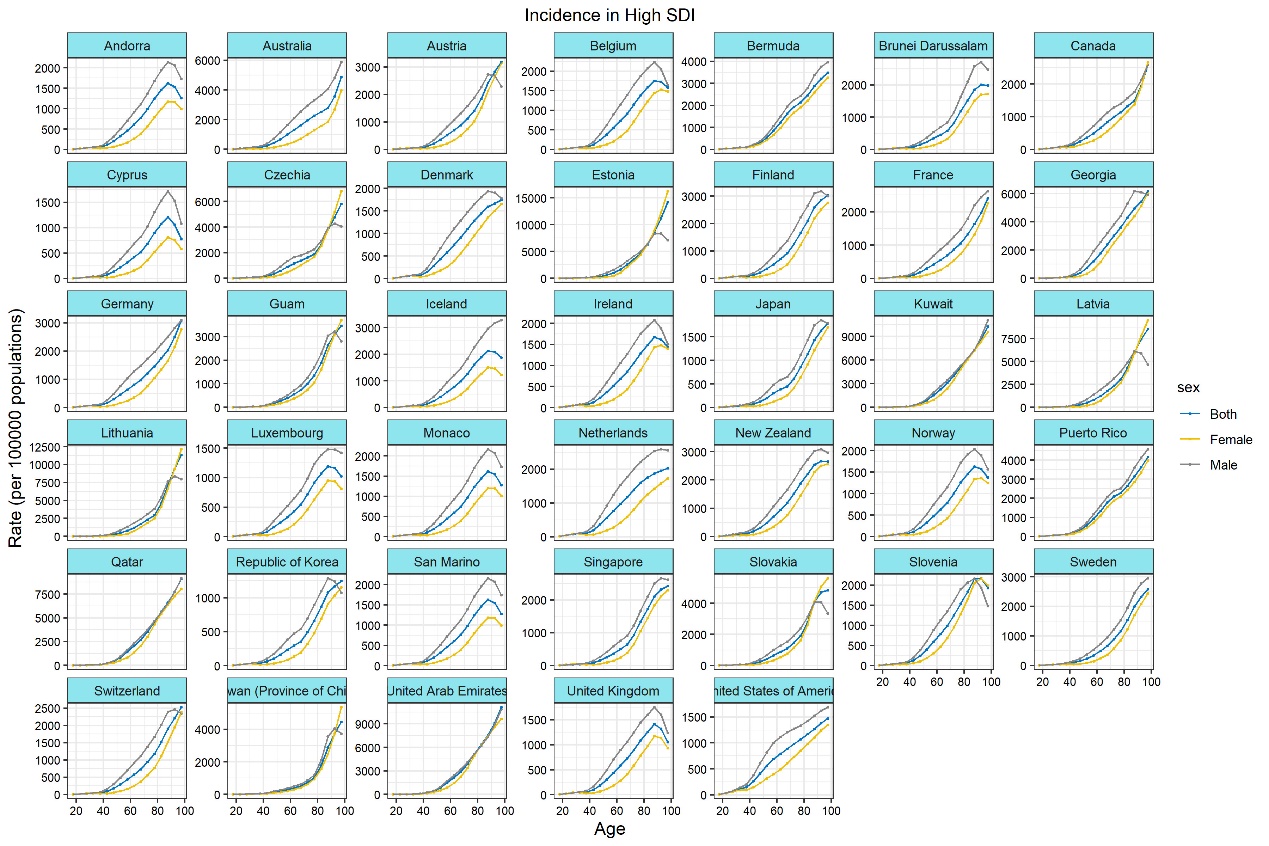


Figure S20. Age effects on incidence of ischemic heart disease in high-SDI countries, 1990–2019. Age effects are shown by the fitted longitudinal age curves of incidence (per 100,000 person-years) adjusted for period deviations.


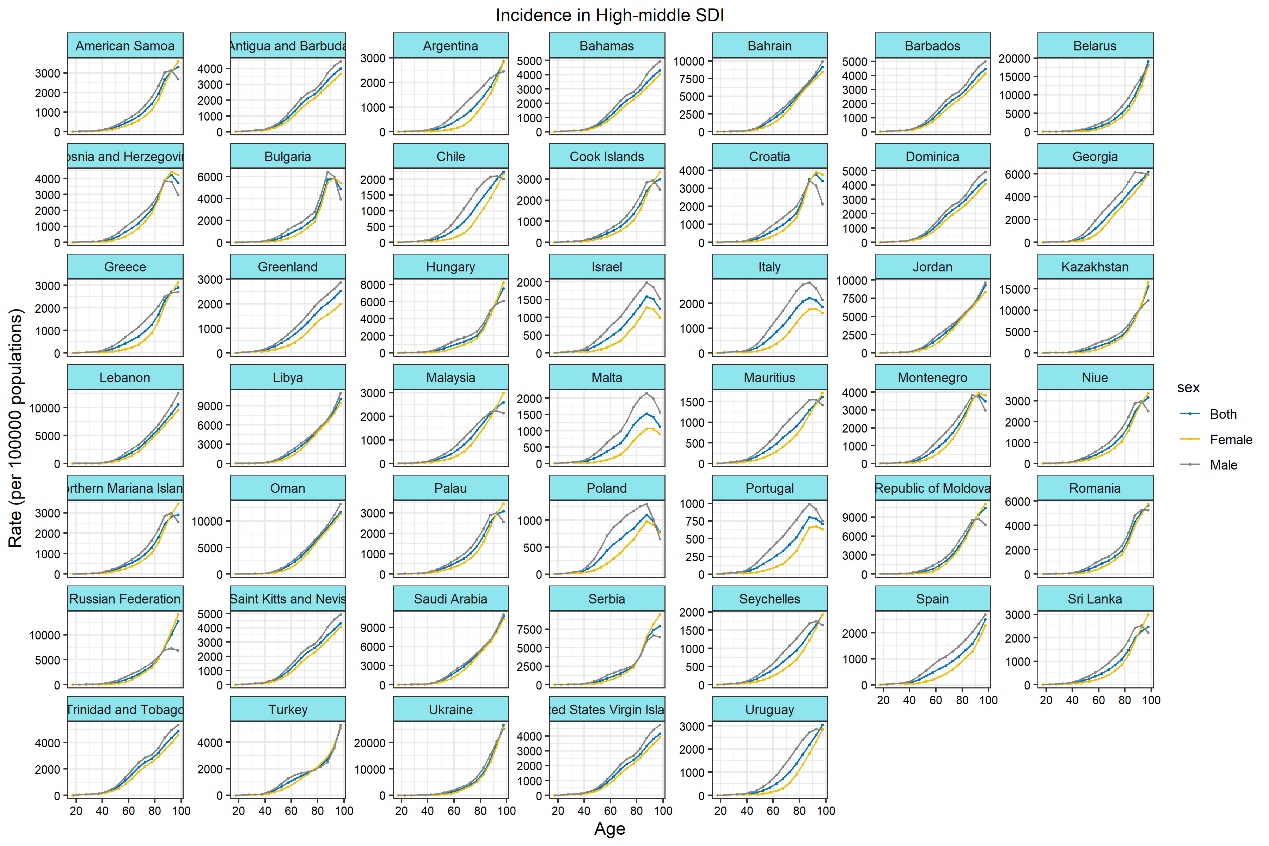


Figure S21. Age effects on incidence of ischemic heart disease in high-middle SDI countries, 1990–2019. Age effects are shown by the fitted longitudinal age curves of incidence (per 100,000 person-years) adjusted for period deviations.


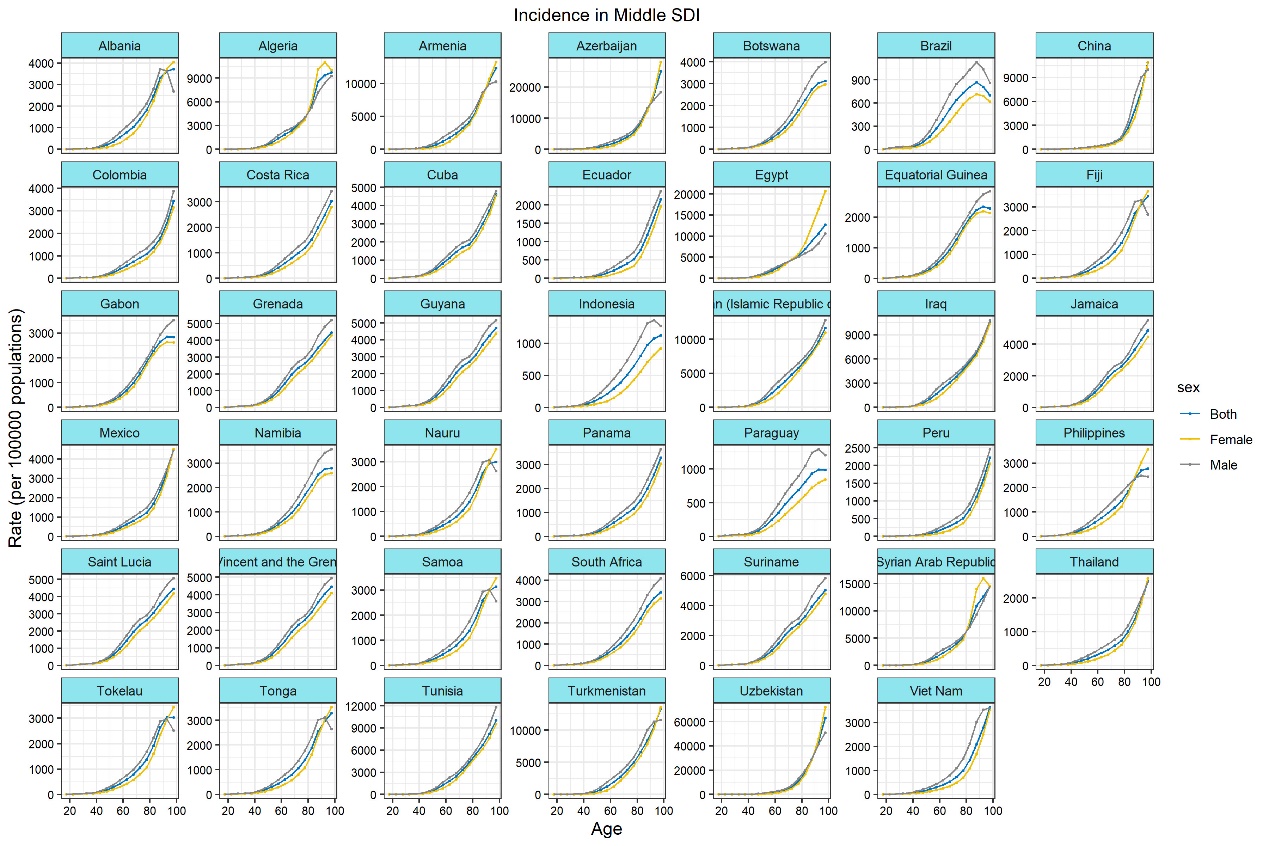


Figure S22. Age effects on incidence of ischemic heart disease in middle-SDI countries, 1990–2019. Age effects are shown by the fitted longitudinal age curves of incidence (per 100,000 person-years) adjusted for period deviations.


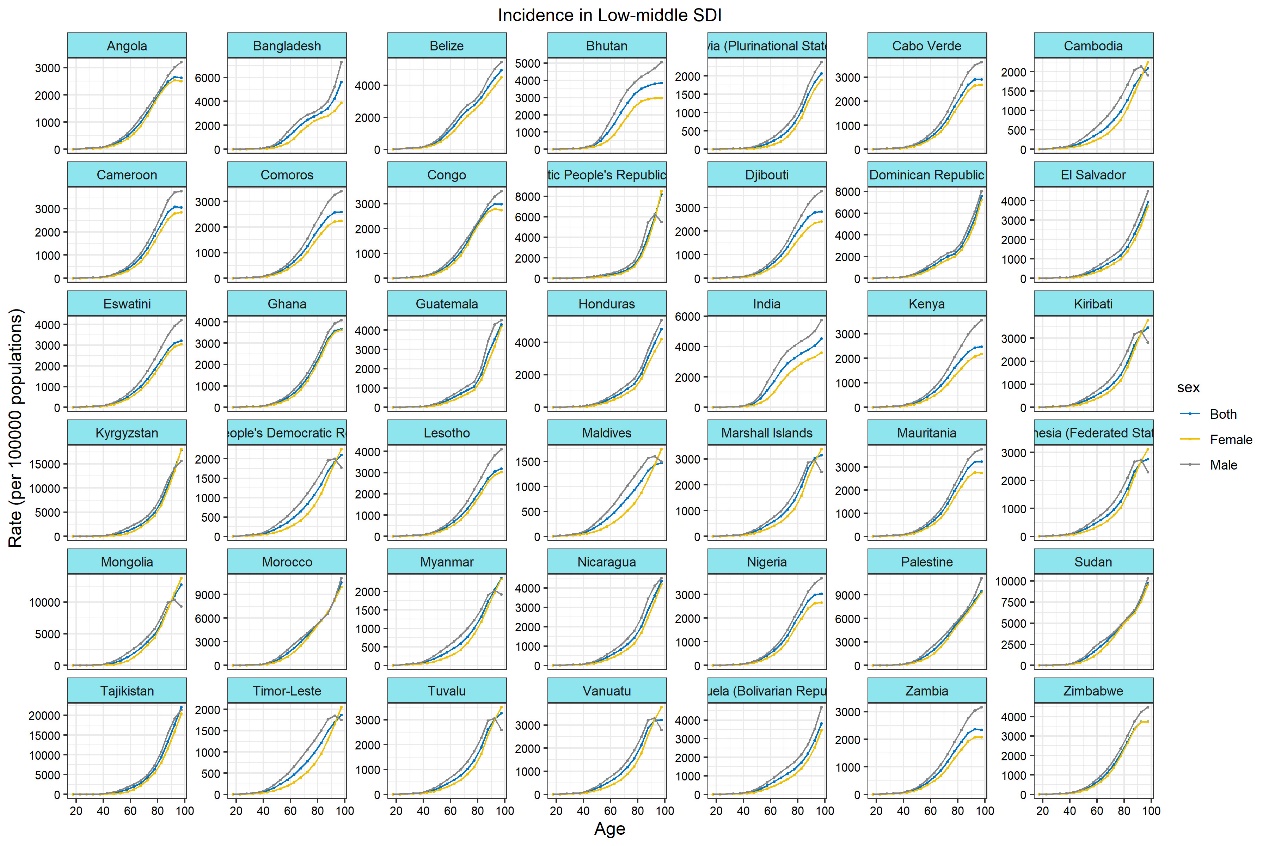


Figure S23. Age effects on incidence of ischemic heart disease in low-middle SDI countries, 1990–2019. Age effects are shown by the fitted longitudinal age curves of incidence (per 100,000 person-years) adjusted for period deviations.


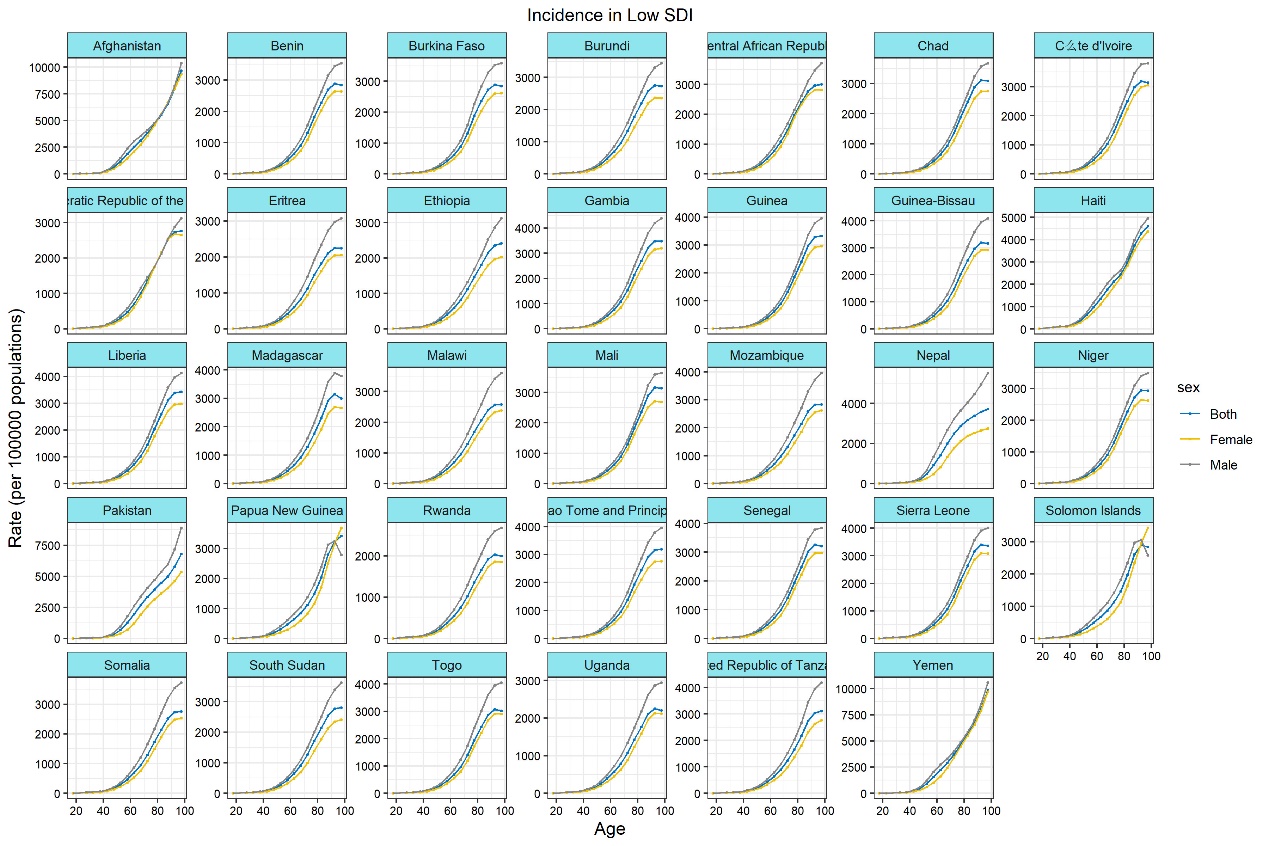


Figure S24. Age effects on incidence of ischemic heart disease in low-SDI countries, 1990–2019. Age effects are shown by the fitted longitudinal age curves of incidence (per 100,000 person-years) adjusted for period deviations.


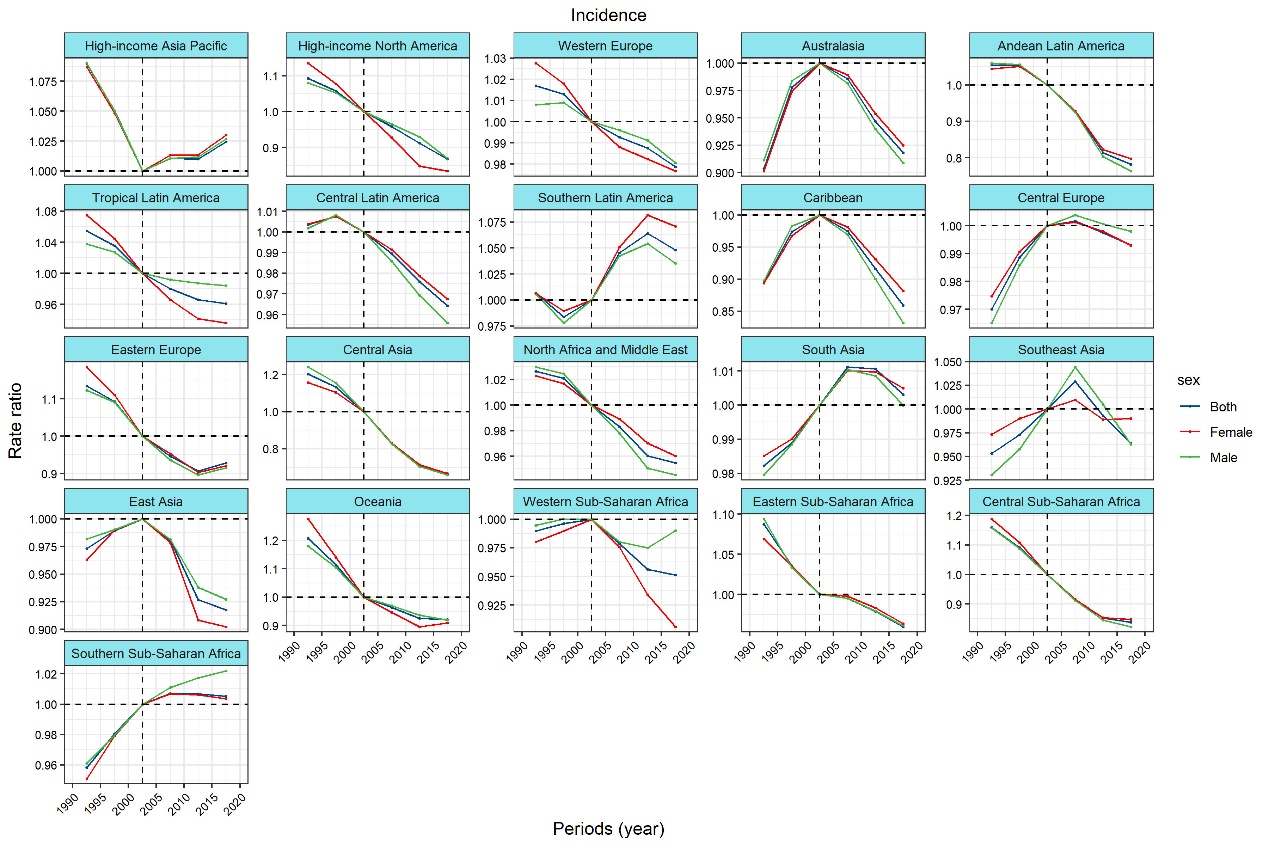


Figure S25. Period effects on incidence of ischemic heart disease in global, 1990-2019. Period effects are shown by the relative risk of incidence (incidence rate ratio) and computed as the ratio of age-specific rates from 1990–1994 to 2015–2019 (with 2000–2005 as the referent period). The dots and shaded areas denote incidence rates or rate ratios and their corresponding 95% CIs.


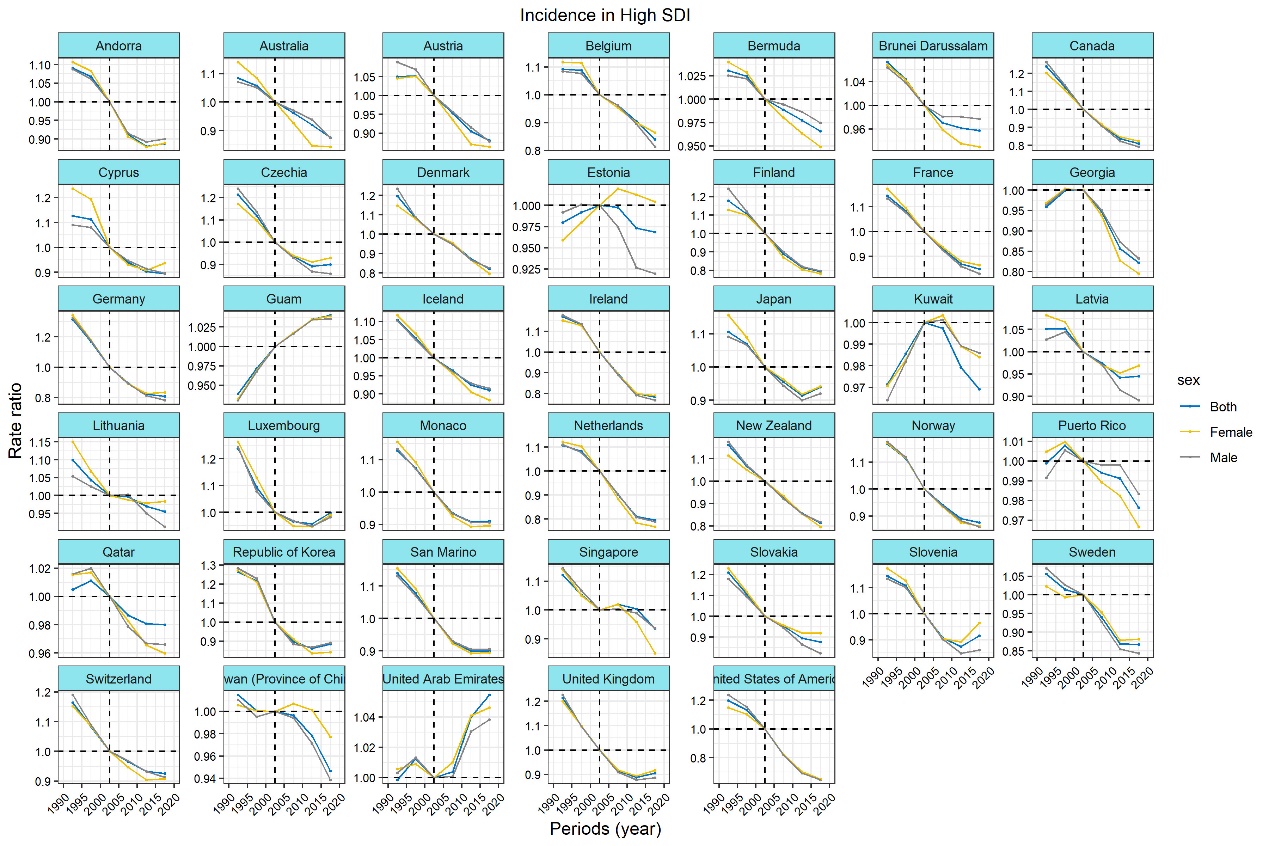


Figure S26. Period effects on incidence of ischemic heart disease in high-SDI countries, 1990-2019. Period effects are shown by the relative risk of incidence (incidence rate ratio) and computed as the ratio of age-specific rates from 1990–1994 to 2015–2019 (with 2000–2005 as the referent period). The dots and shaded areas denote incidence rates or rate ratios and their corresponding 95% CIs.


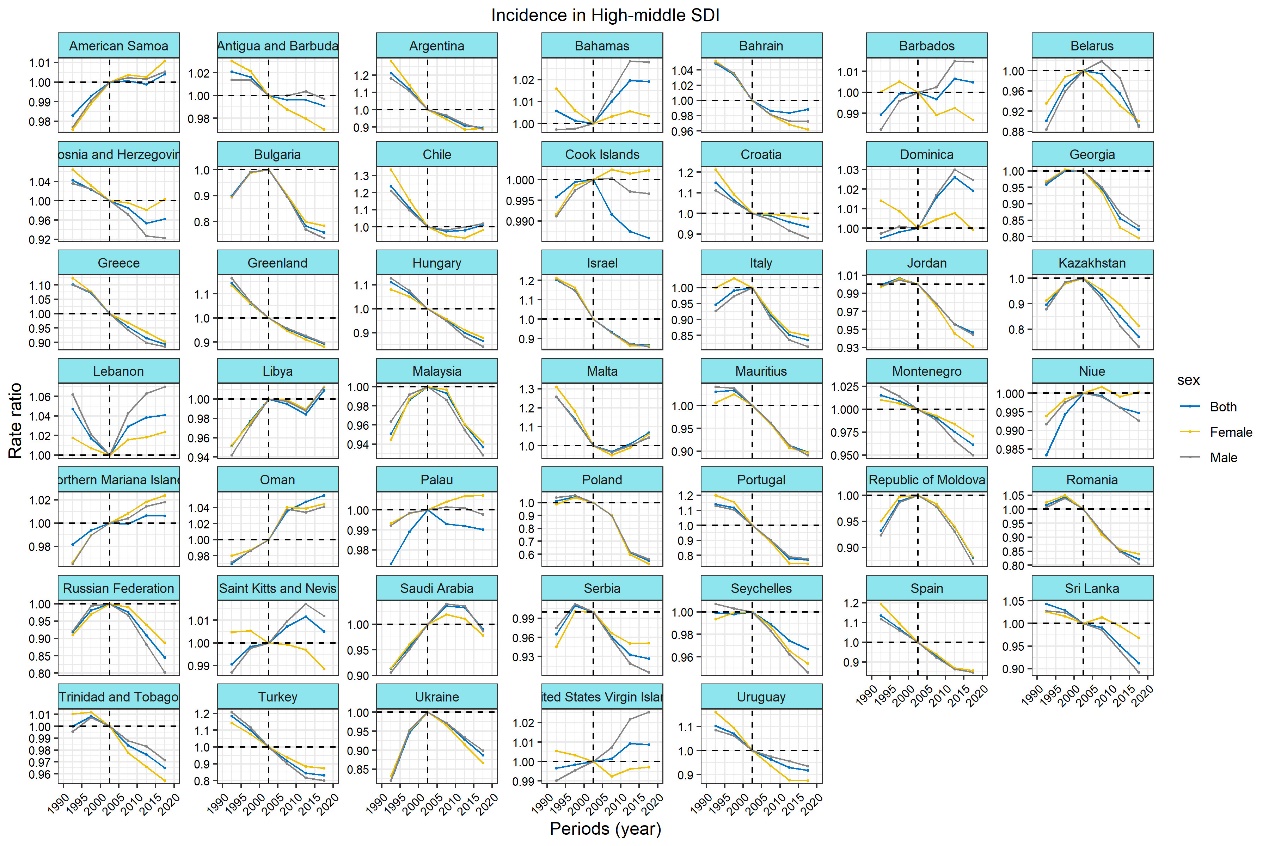


Figure S27. Period effects on incidence of ischemic heart disease in high-middle SDI countries, 1990-2019. Period effects are shown by the relative risk of incidence (incidence rate ratio) and computed as the ratio of age-specific rates from 1990–1994 to 2015–2019 (with 2000–2005 as the referent period). The dots and shaded areas denote incidence rates or rate ratios and their corresponding 95% CIs.


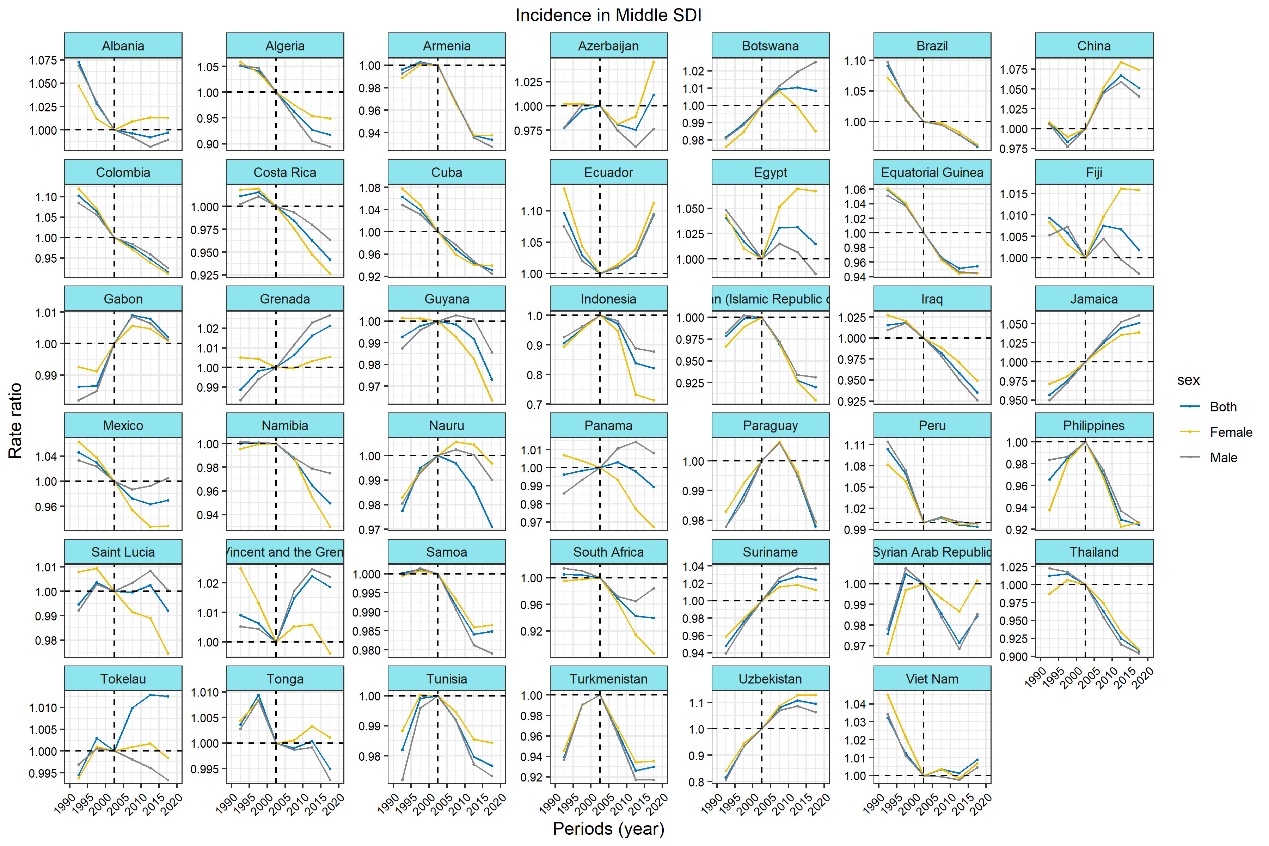


Figure S28. Period effects on incidence of ischemic heart disease in middle-SDI countries, 1990-2019. Period effects are shown by the relative risk of incidence (incidence rate ratio) and computed as the ratio of age-specific rates from 1990–1994 to 2015–2019 (with 2000–2005 as the referent period). The dots and shaded areas denote incidence rates or rate ratios and their corresponding 95% CIs.


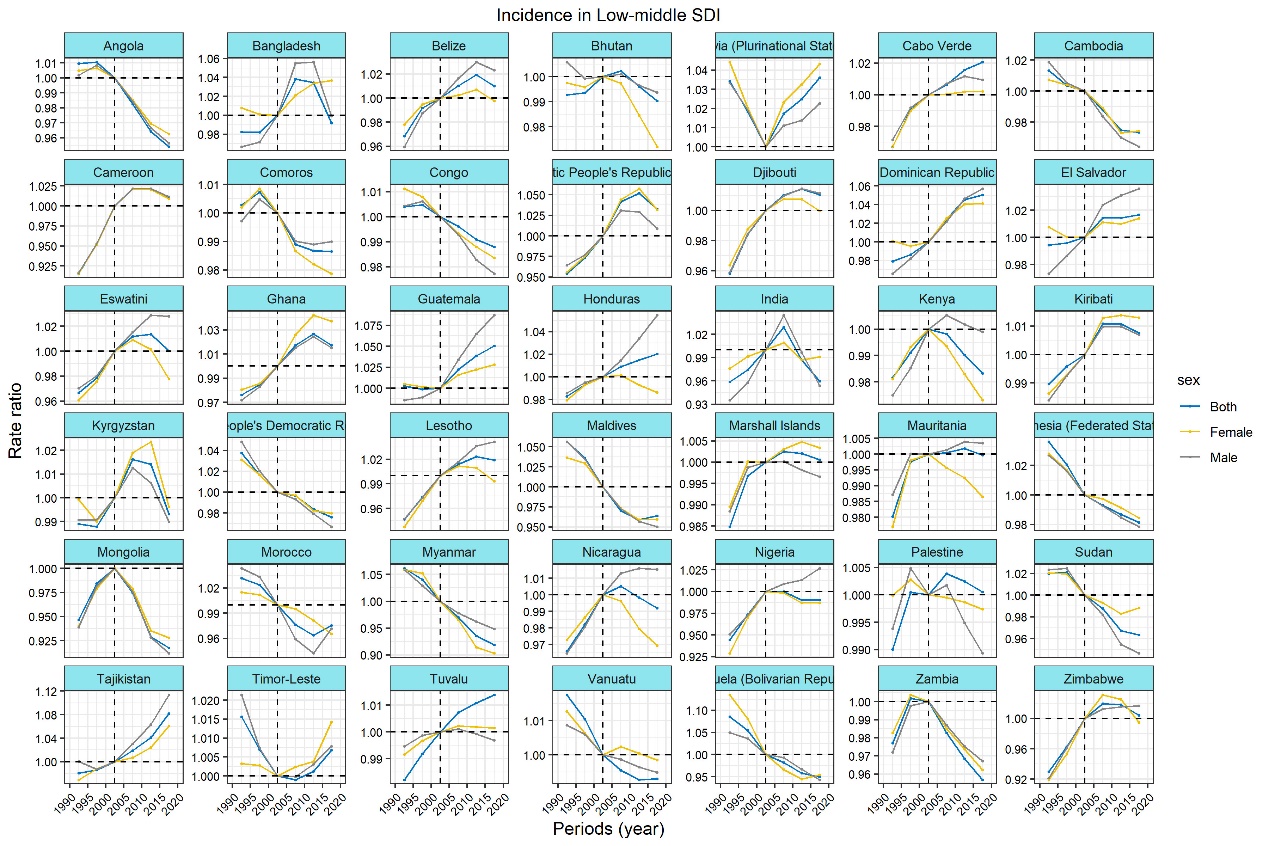


Figure S29. Period effects on incidence of ischemic heart disease in low-middle SDI countries, 1990-2019. Period effects are shown by the relative risk of incidence (incidence rate ratio) and computed as the ratio of age-specific rates from 1990–1994 to 2015–2019 (with 2000–2005 as the referent period). The dots and shaded areas denote incidence rates or rate ratios and their corresponding 95% CIs.


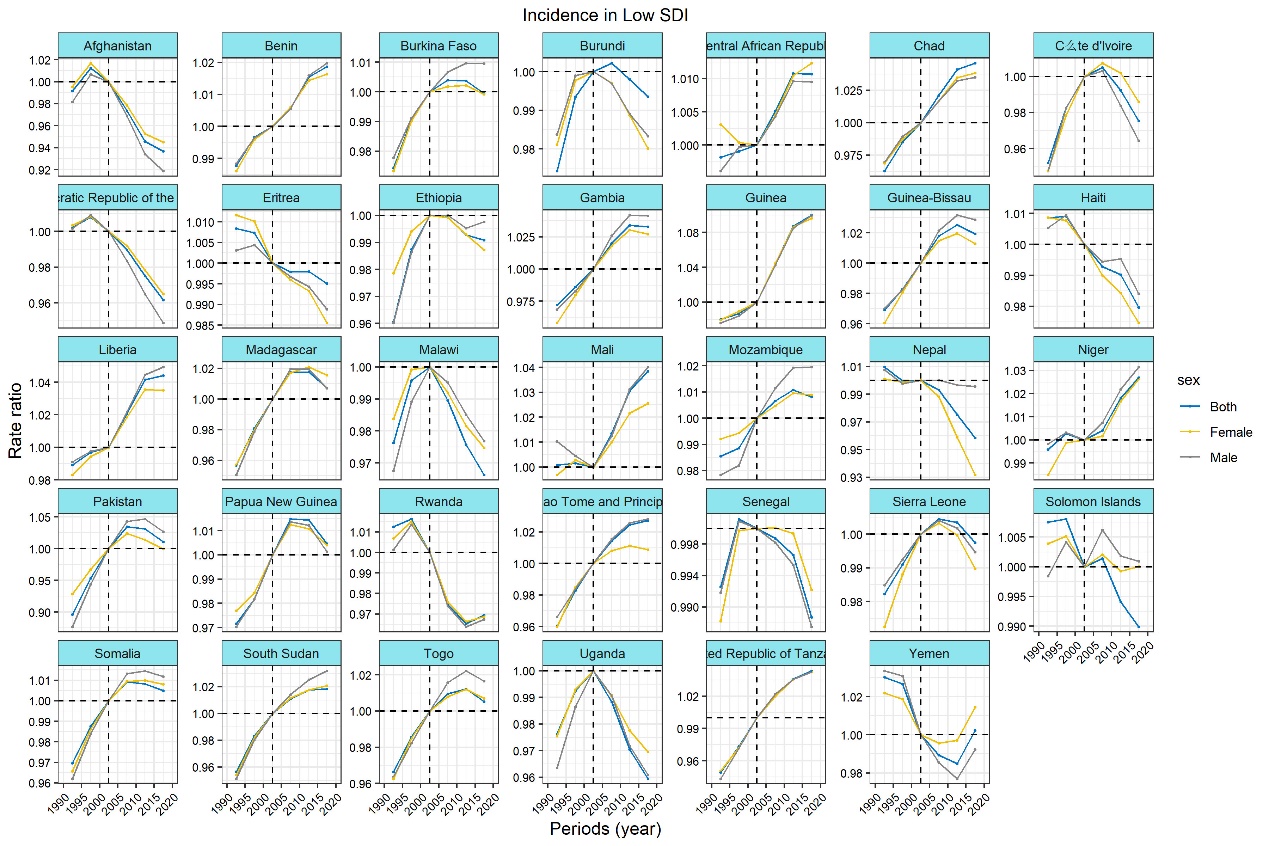


Figure S30. Period effects on incidence of ischemic heart disease in low-SDI countries, 1990-2019. Period effects are shown by the relative risk of incidence (incidence rate ratio) and computed as the ratio of age-specific rates from 1990–1994 to 2015–2019 (with 2000–2005 as the referent period). The dots and shaded areas denote incidence rates or rate ratios and their corresponding 95% CIs.


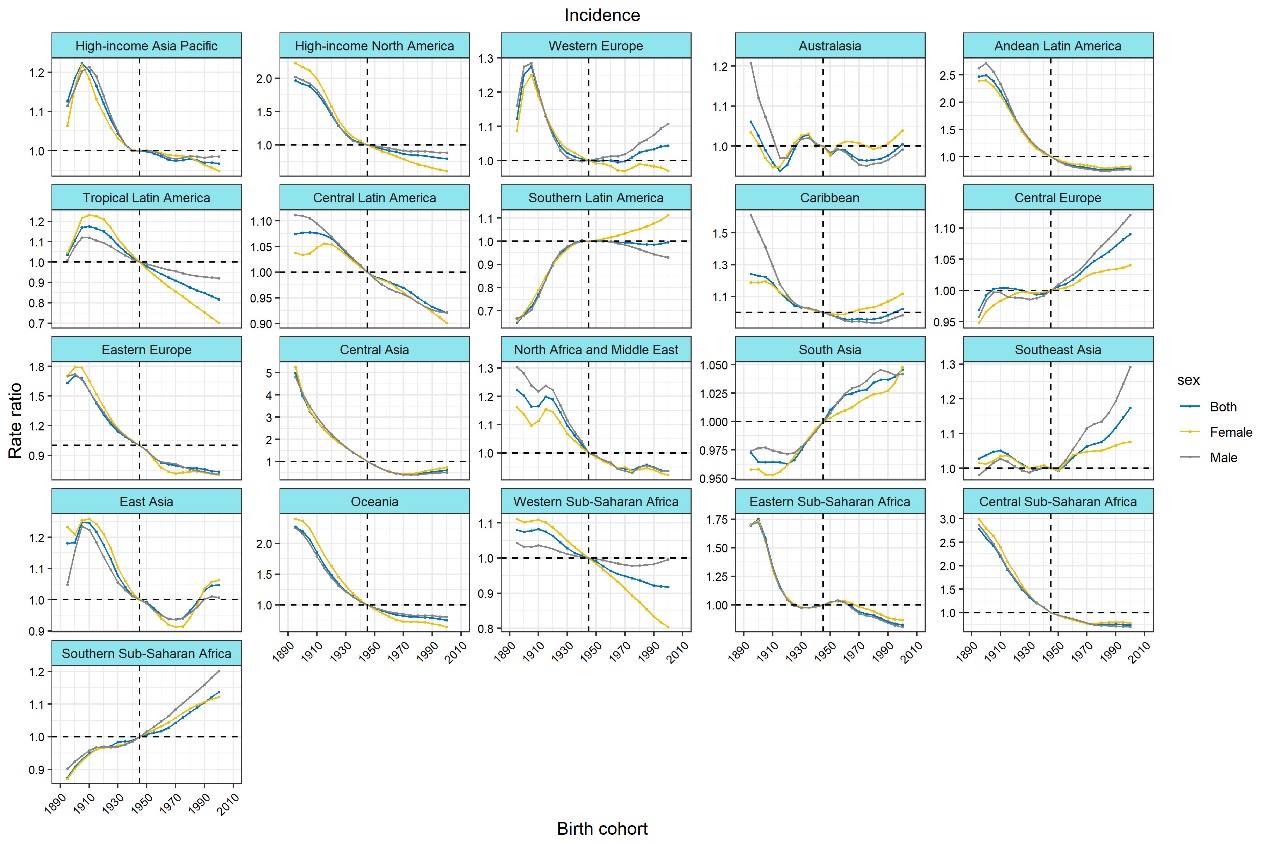


Figure S31. Cohort effects on incidence of ischemic heart disease incidence in global, 1990-2019. Cohort effects are shown by the relative risk of incidence and computed as the ratio of age-specific rates from the 1895 cohort to the 2000 cohort, with the referent cohort set at 1945. The dots and shaded areas denote incidence rates or rate ratios and their corresponding 95% CIs.


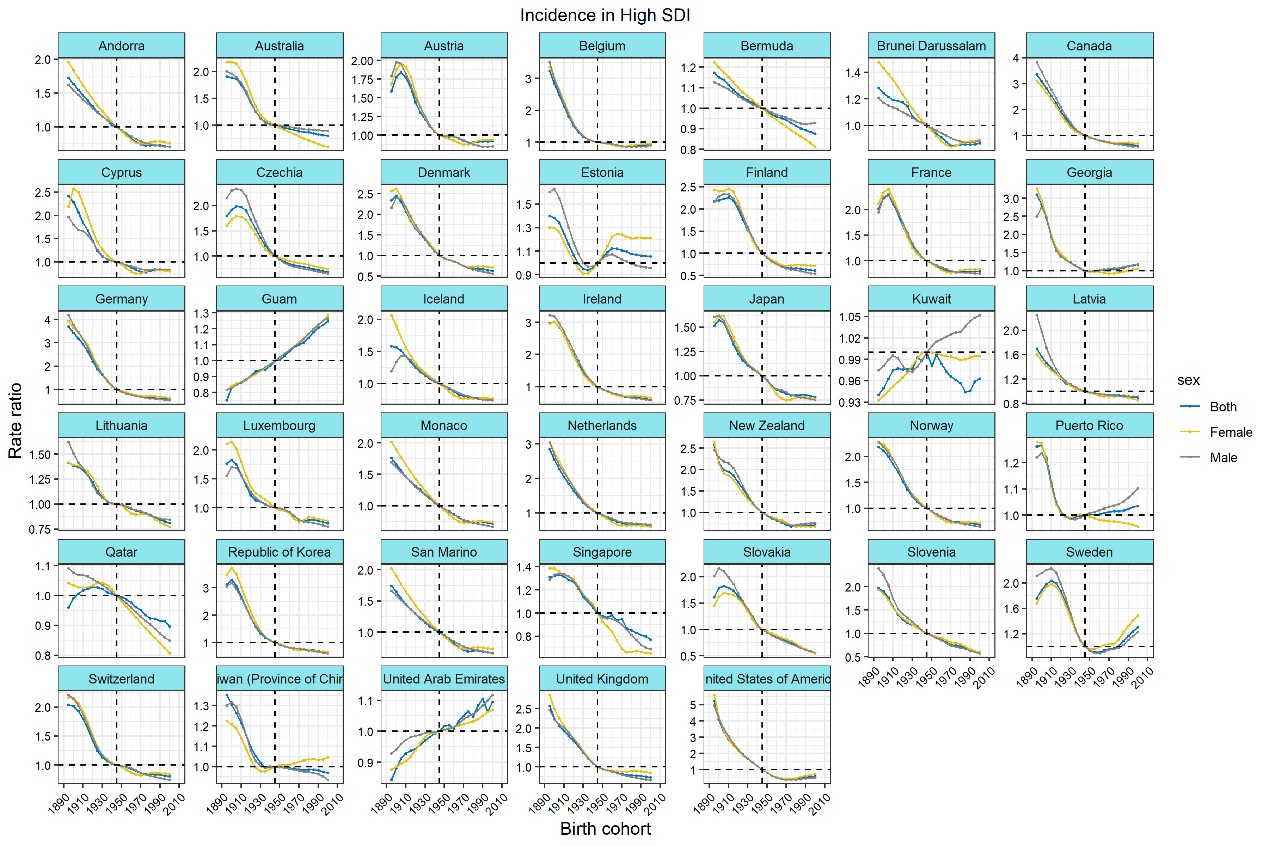


Figure S32. Cohort effects on incidence of ischemic heart disease incidence in high-SDI countries, 1990-2019. Cohort effects are shown by the relative risk of incidence and computed as the ratio of age-specific rates from the 1895 cohort to the 2000 cohort, with the referent cohort set at 1945. The dots and shaded areas denote incidence rates or rate ratios and their corresponding 95% CIs.


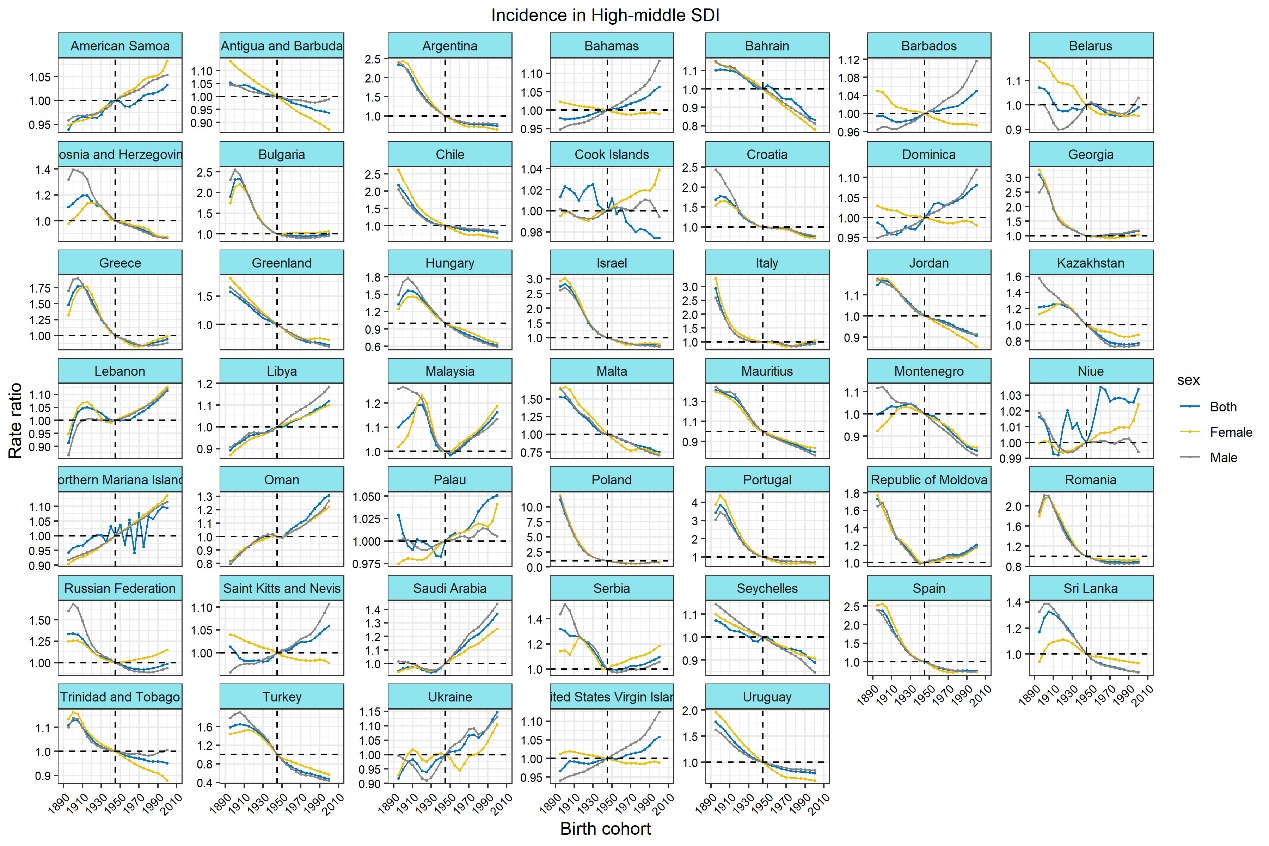


Figure S33. Cohort effects on incidence of ischemic heart disease incidence in high-middle SDI countries, 1990-2019. Cohort effects are shown by the relative risk of incidence and computed as the ratio of age-specific rates from the 1895 cohort to the 2000 cohort, with the referent cohort set at 1945. The dots and shaded areas denote incidence rates or rate ratios and their corresponding 95% CIs.


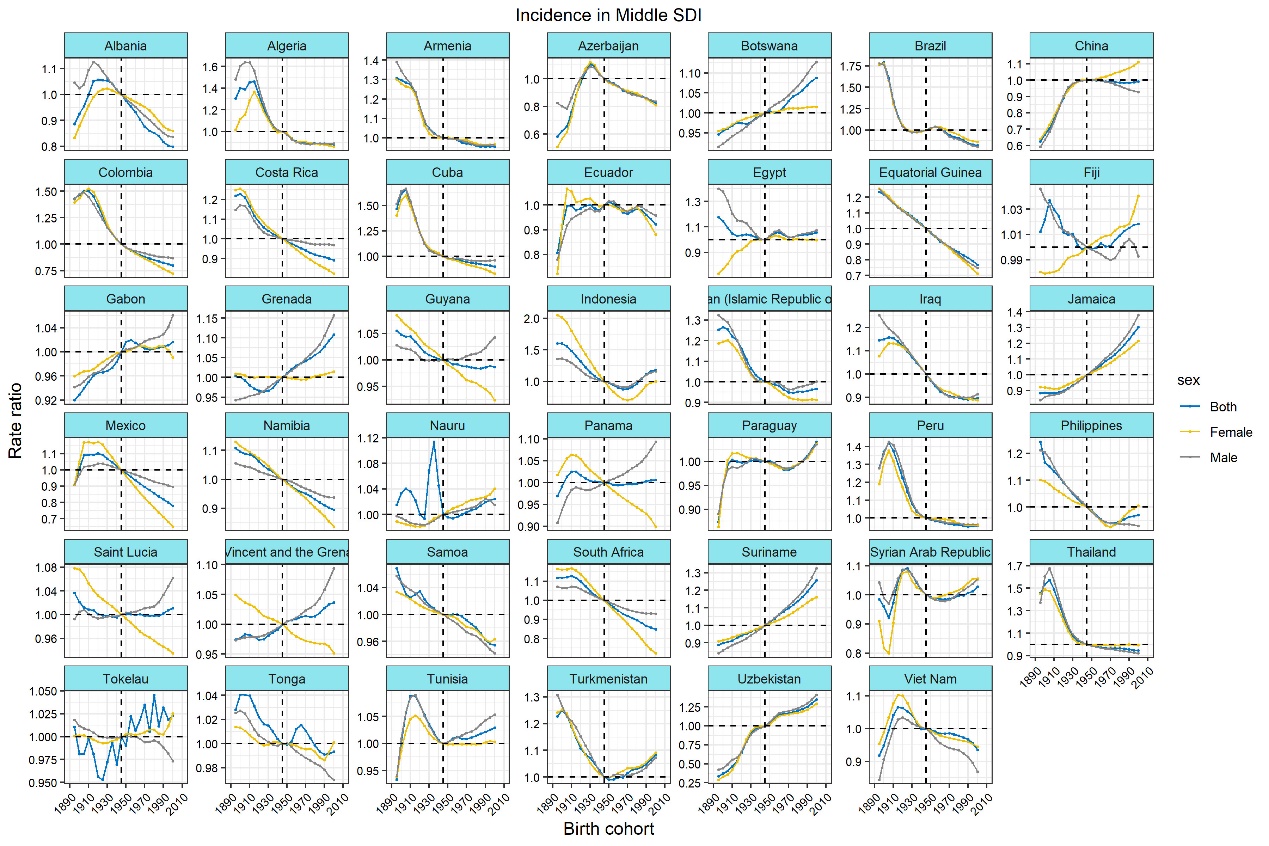


Figure S34. Cohort effects on incidence of ischemic heart disease incidence in middle-SDI countries, 1990-2019. Cohort effects are shown by the relative risk of incidence and computed as the ratio of age-specific rates from the 1895 cohort to the 2000 cohort, with the referent cohort set at 1945. The dots and shaded areas denote incidence rates or rate ratios and their corresponding 95% CIs.


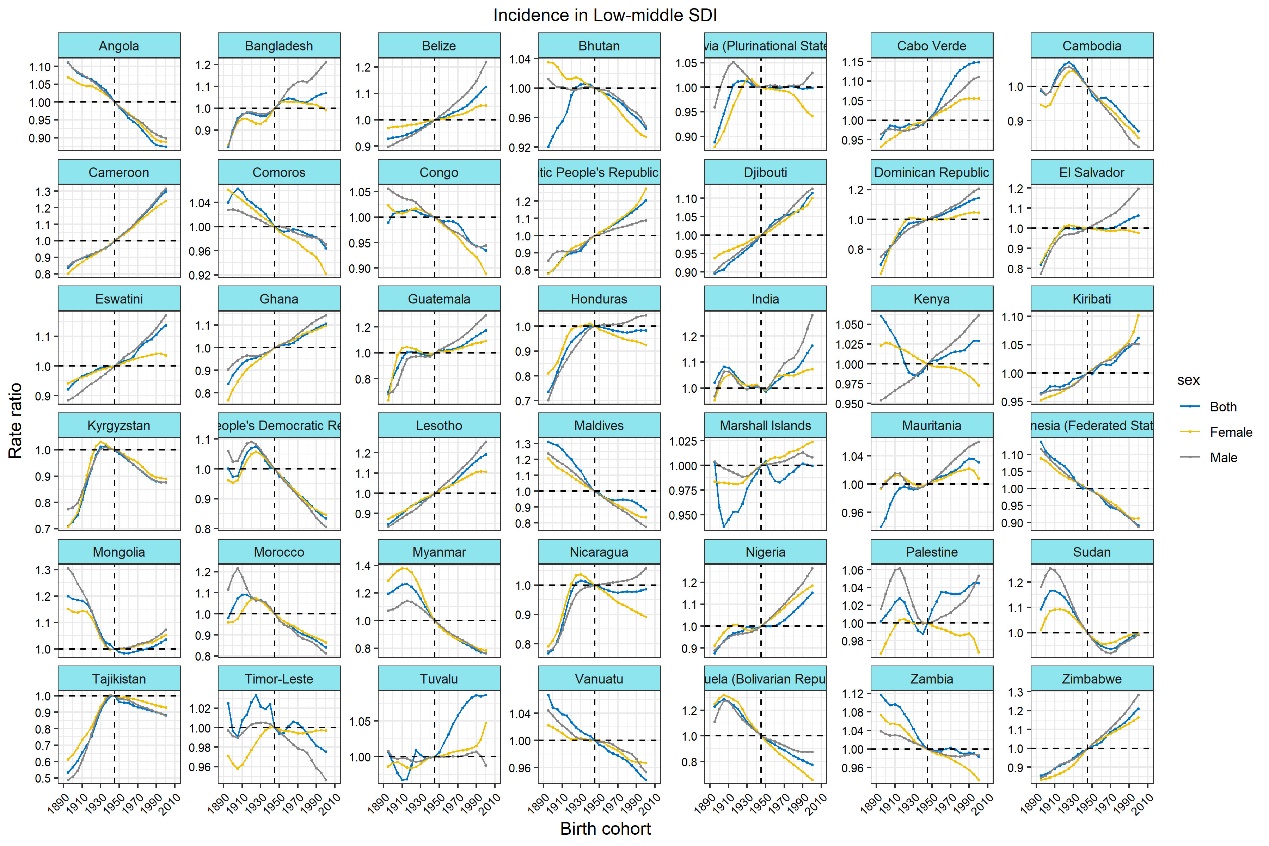


Figure S35. Cohort effects on incidence of ischemic heart disease incidence in low-middle SDI countries, 1990-2019. Cohort effects are shown by the relative risk of incidence and computed as the ratio of age-specific rates from the 1895 cohort to the 2000 cohort, with the referent cohort set at 1945. The dots and shaded areas denote incidence rates or rate ratios and their corresponding 95% CIs.


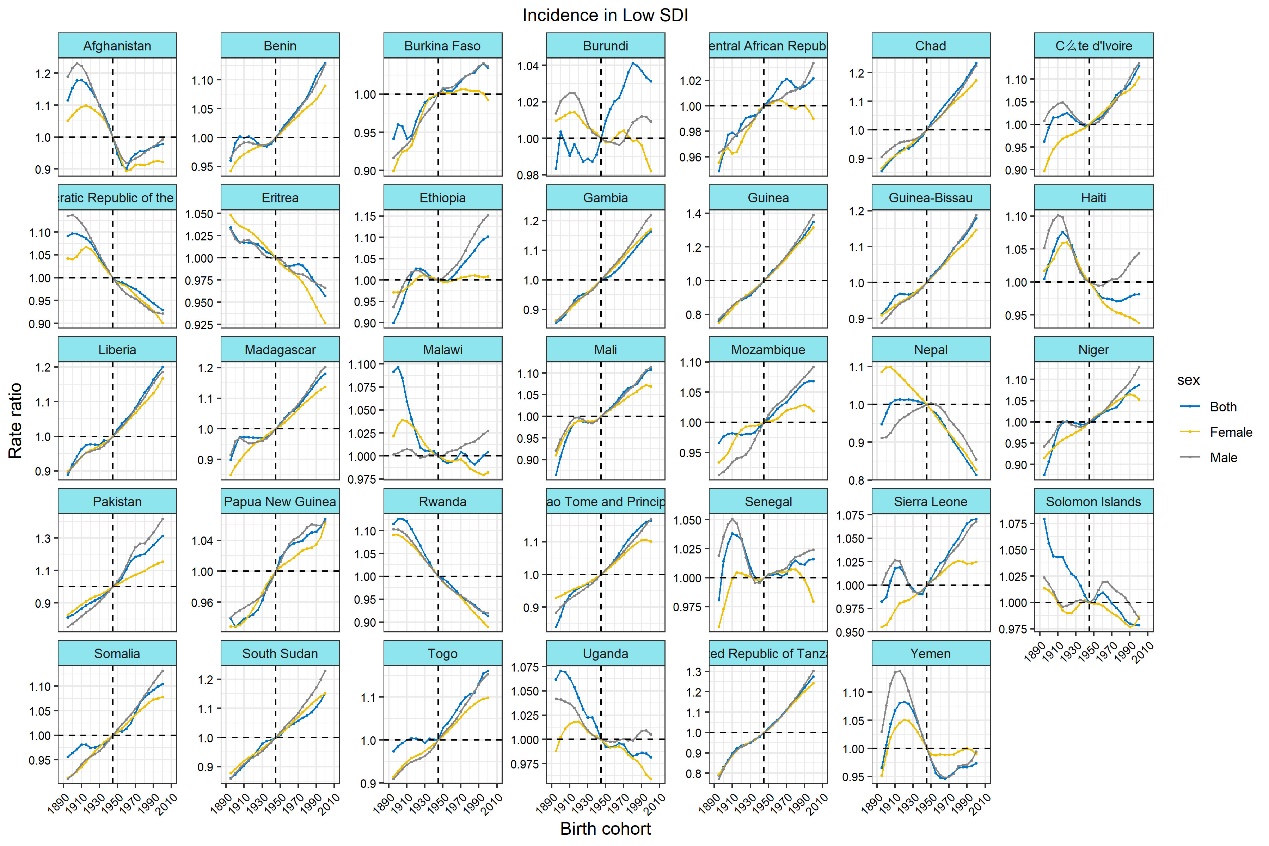


Figure S36. Cohort effects on incidence of ischemic heart disease incidence in low-SDI countries, 1990-2019. Cohort effects are shown by the relative risk of incidence and computed as the ratio of age-specific rates from the 1895 cohort to the 2000 cohort, with the referent cohort set at 1945. The dots and shaded areas denote incidence rates or rate ratios and their corresponding 95% CIs.


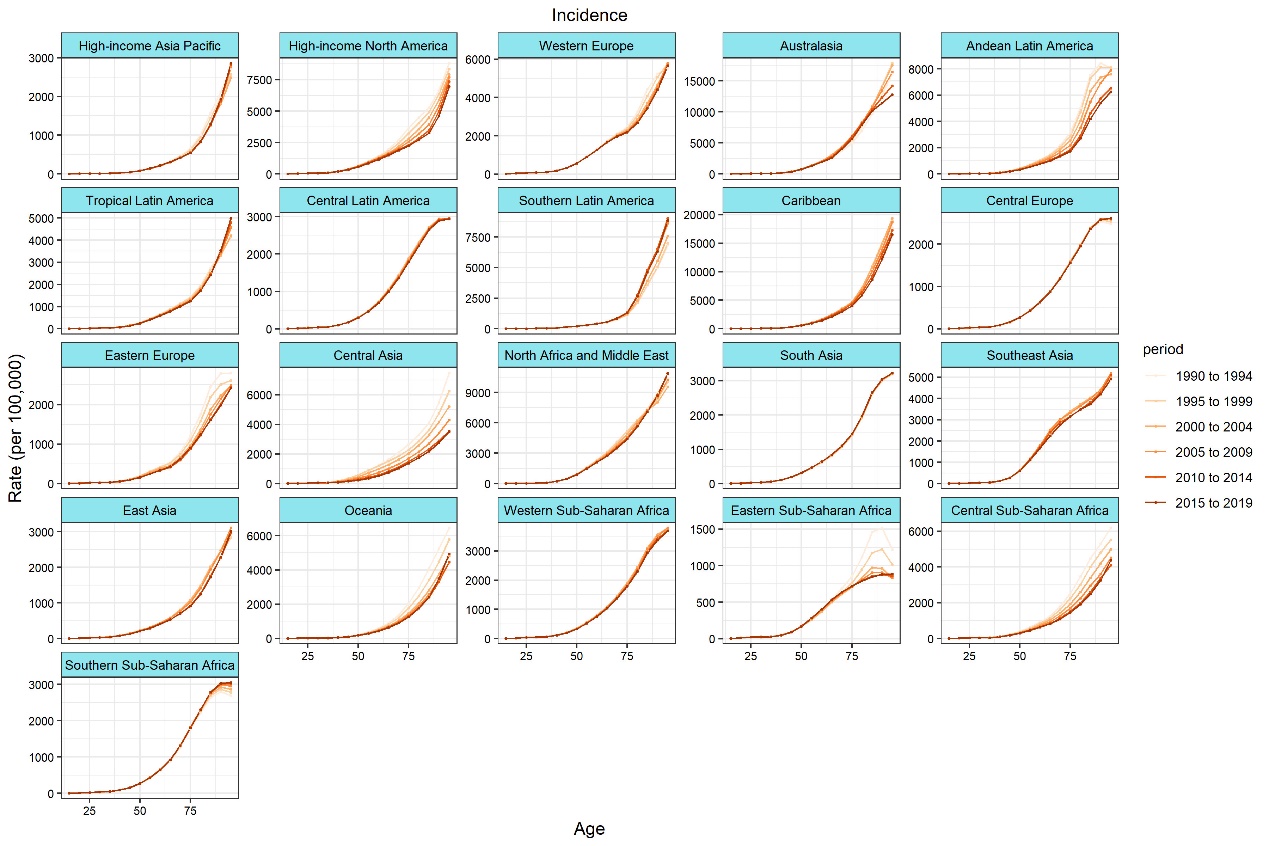


Figure S37. Ischemic heart disease incidence rates across different age groups by period in global, 1990-2019.


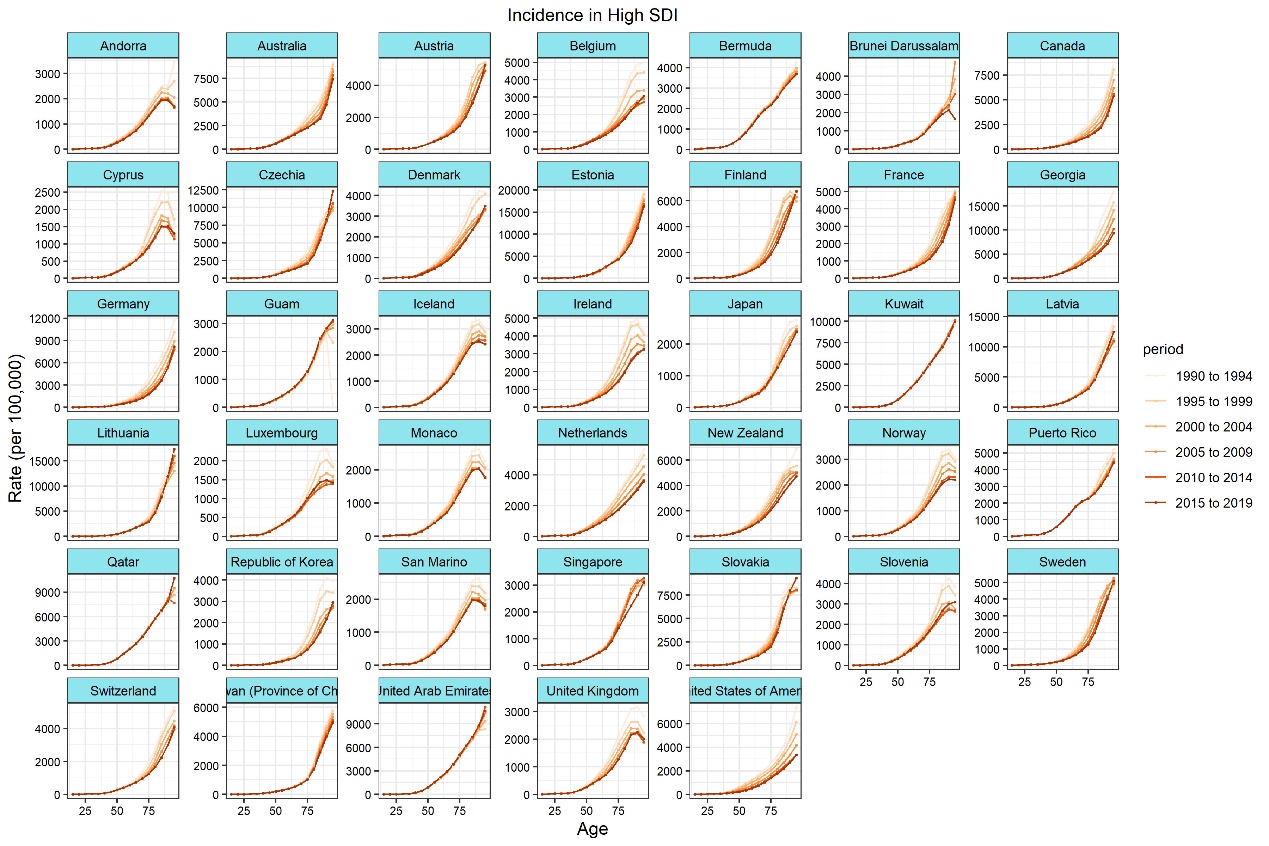


Figure S38. Ischemic heart disease incidence rates across different age groups by period in high-SDI countries, 1990-2019.


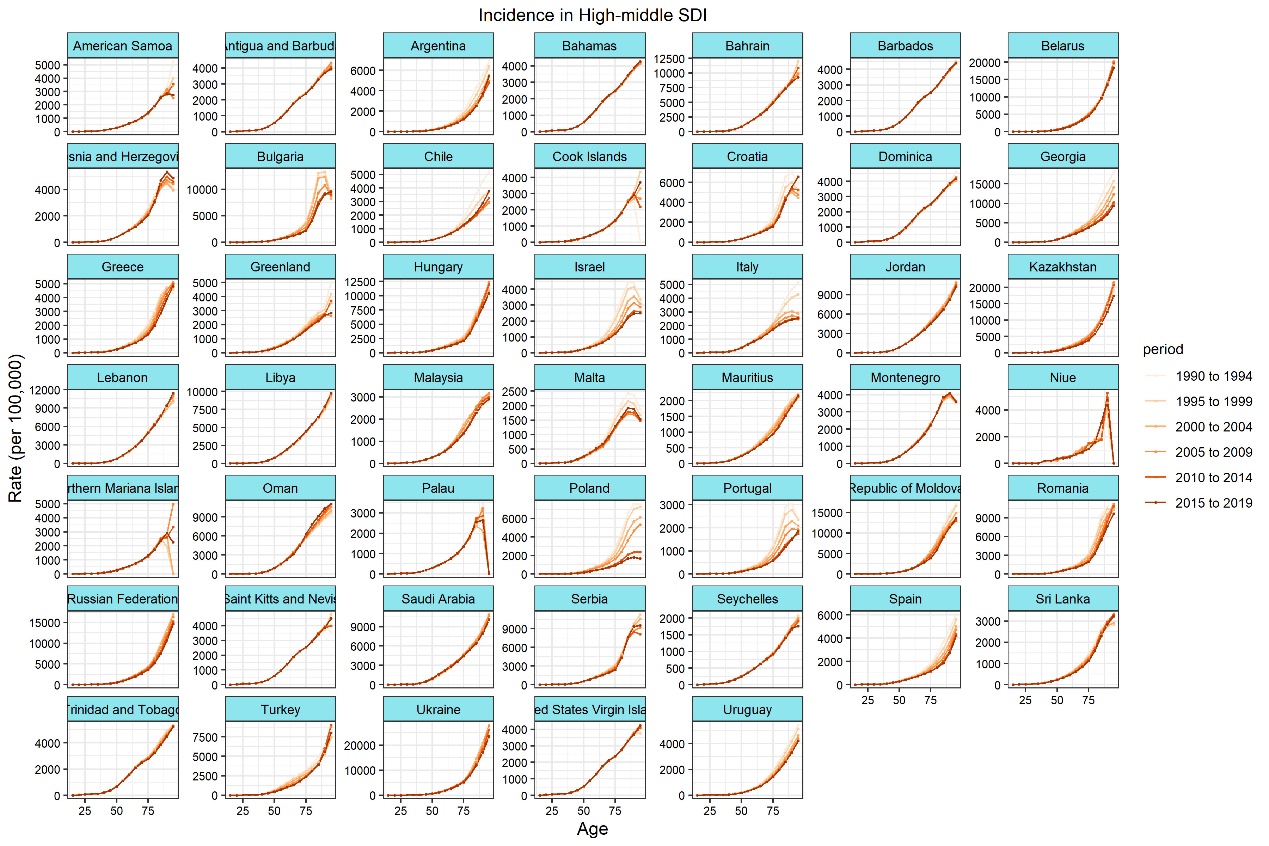


Figure S39. Ischemic heart disease incidence rates across different age groups by period in high-middle SDI countries, 1990-2019.


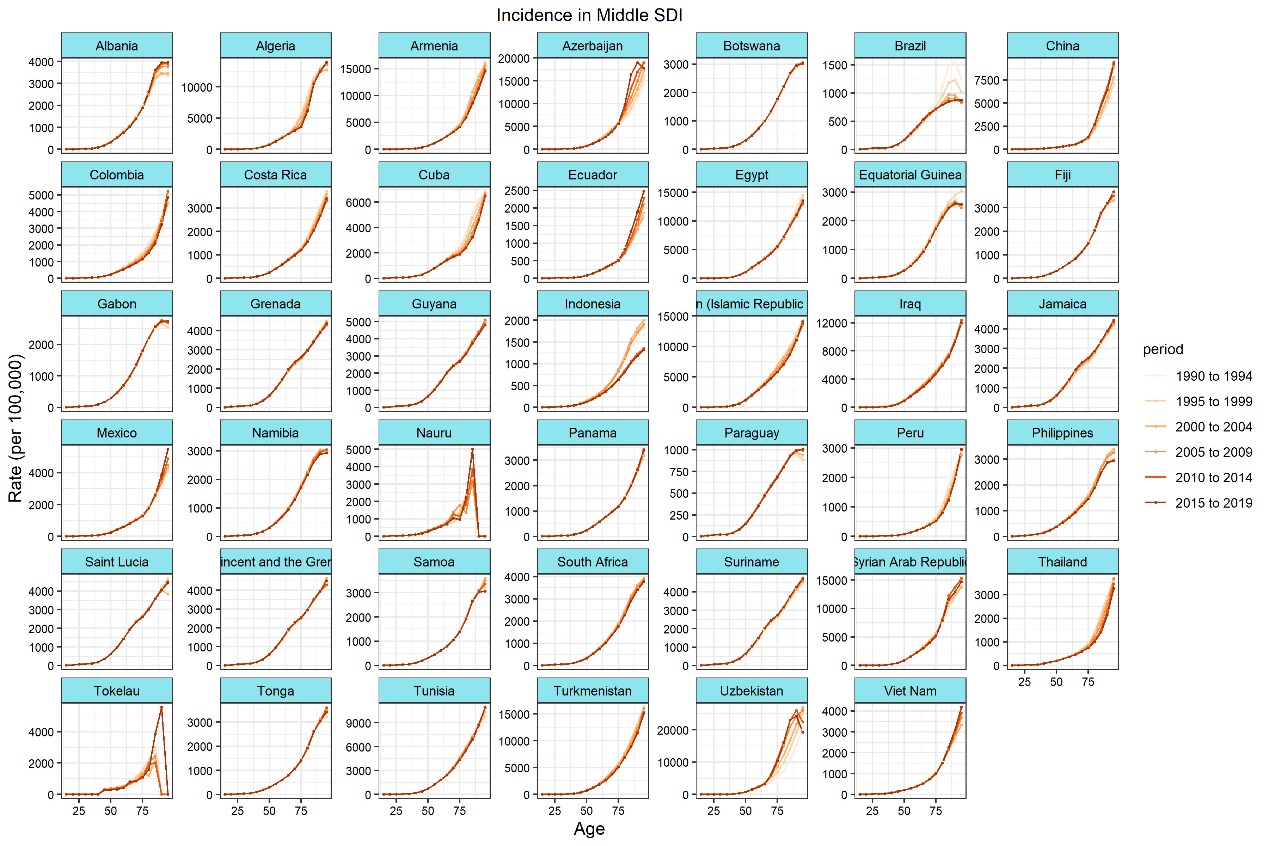


Figure S40. Ischemic heart disease incidence rates across different age groups by period in middle-SDI countries, 1990-2019.


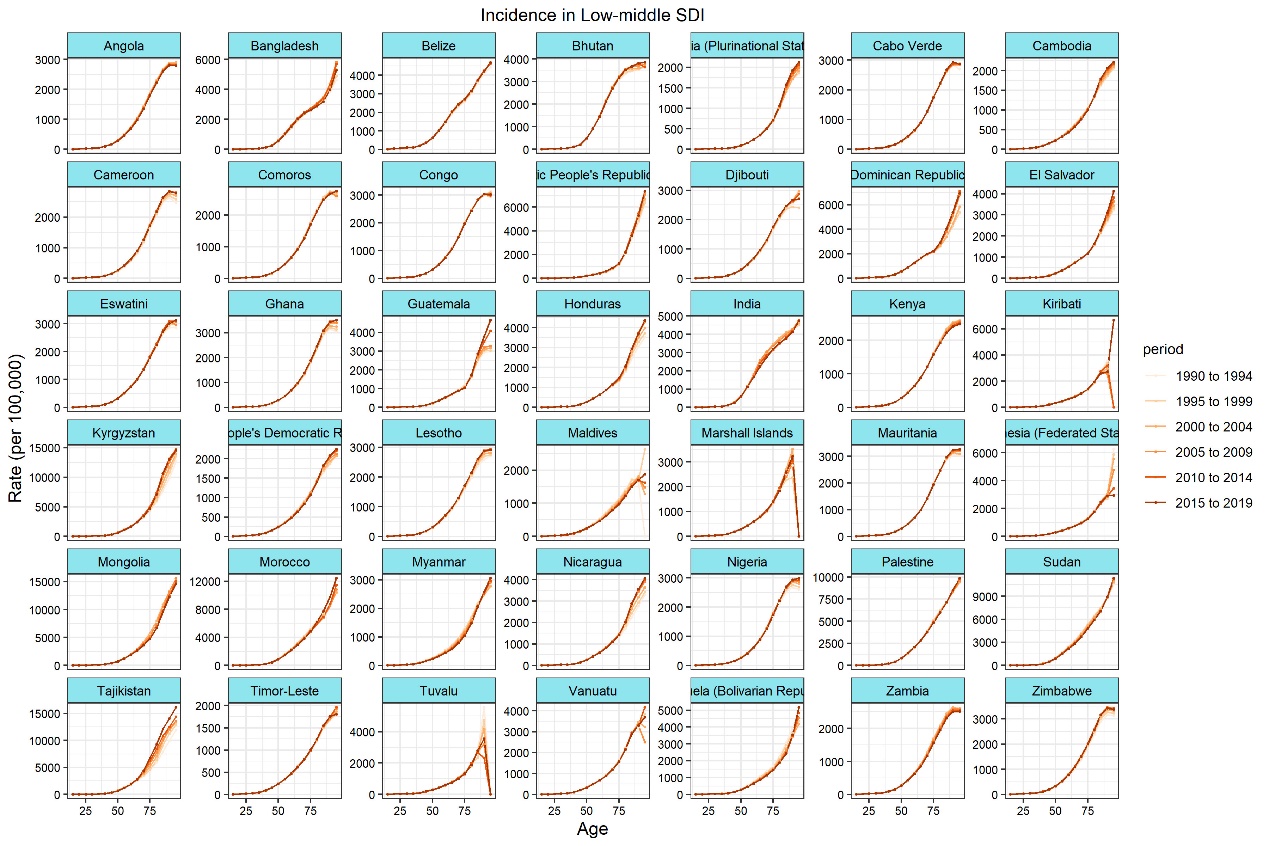


Figure S41. Ischemic heart disease incidence rates across different age groups by period in low-middle SDI countries, 1990-2019.


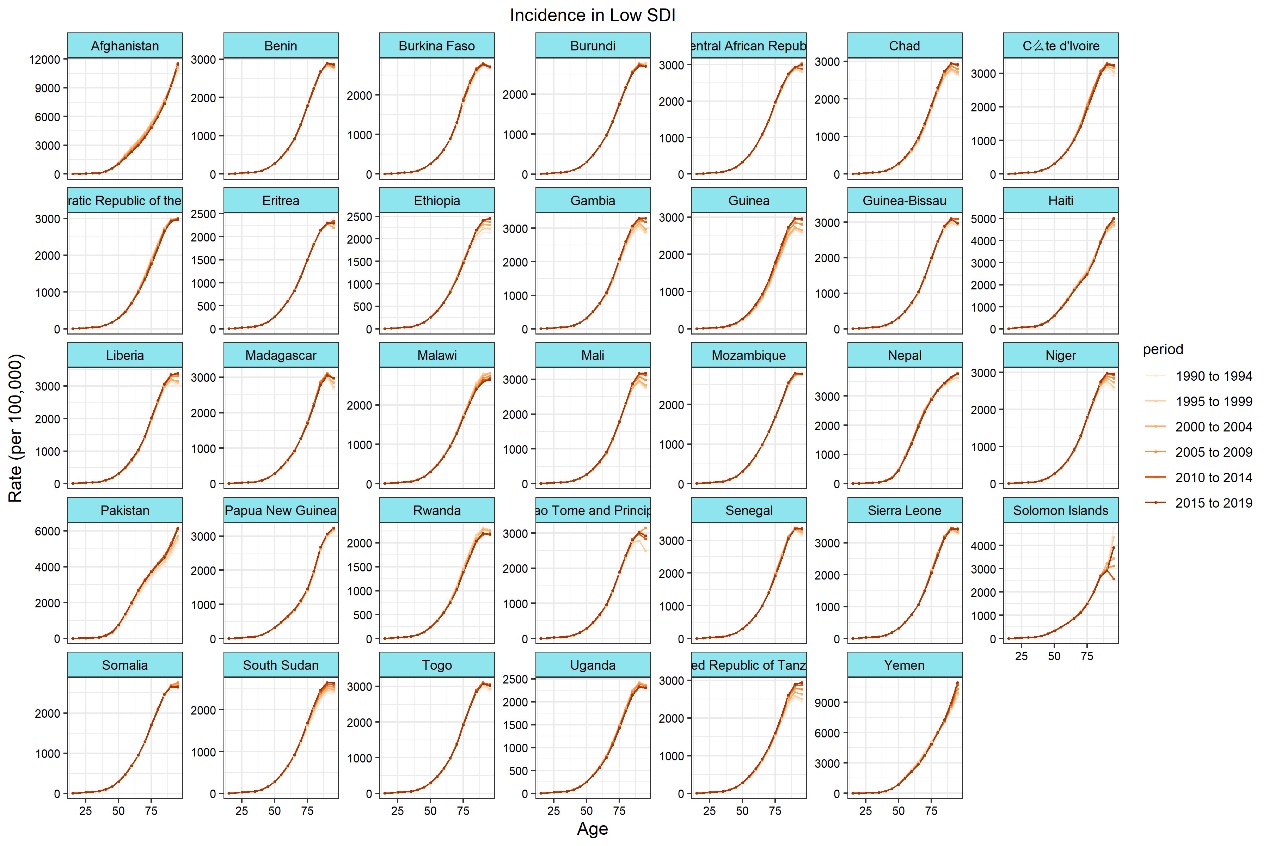


Figure S42. Ischemic heart disease incidence rates across different age groups by period in low-SDI countries, 1990-2019.


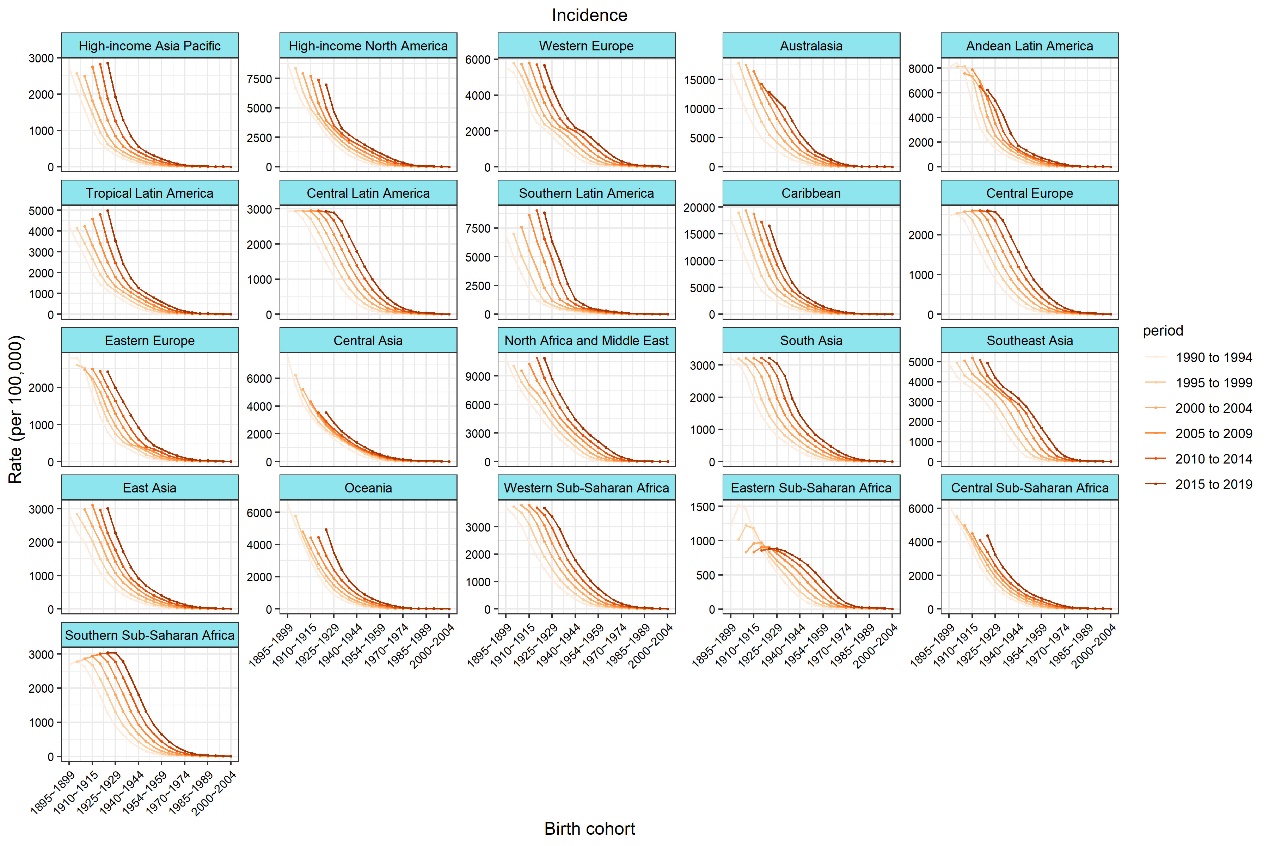


Figure S43. Ischemic heart disease incidence rates across different birth cohorts by period in global, 1990-2019.


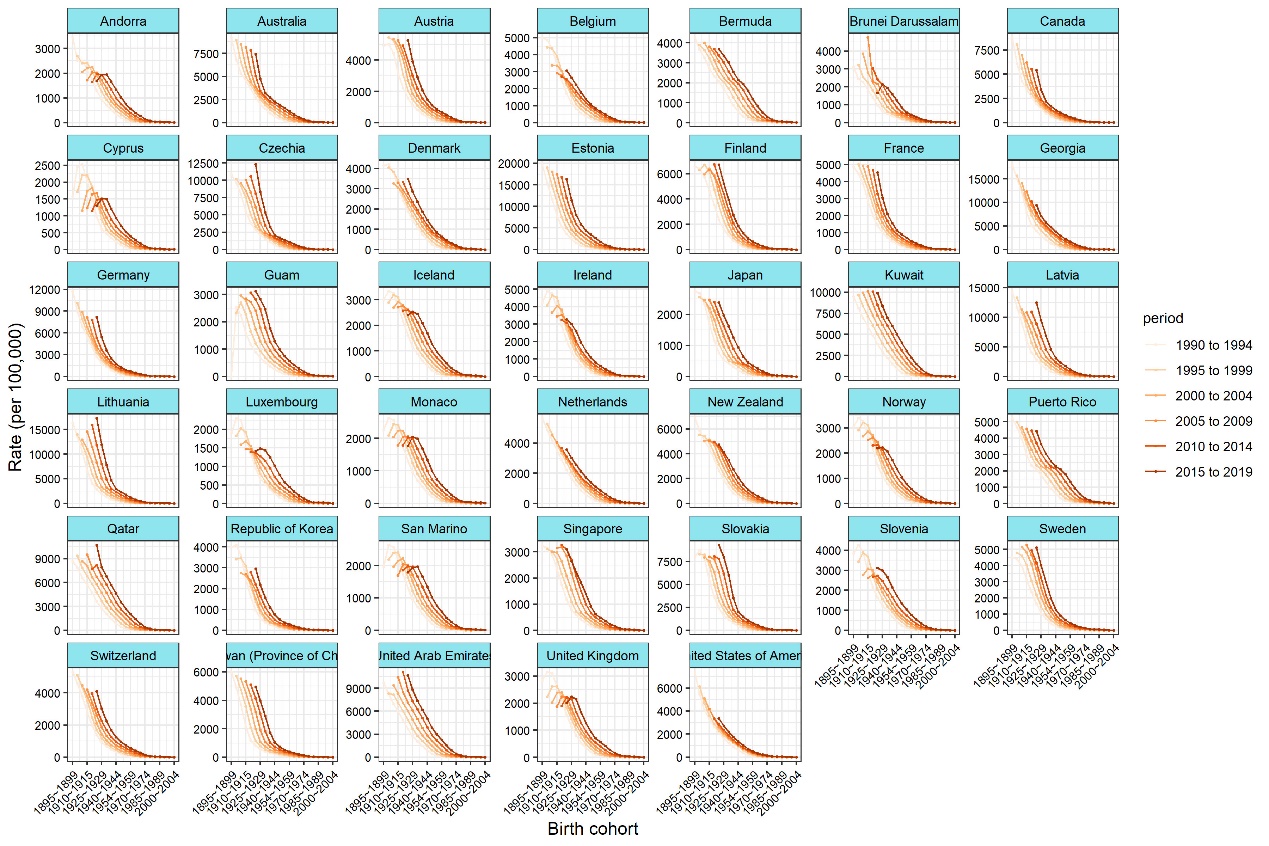


Figure S44. Ischemic heart disease incidence rates across different birth cohorts by period in high-SDI countries, 1990-2019.


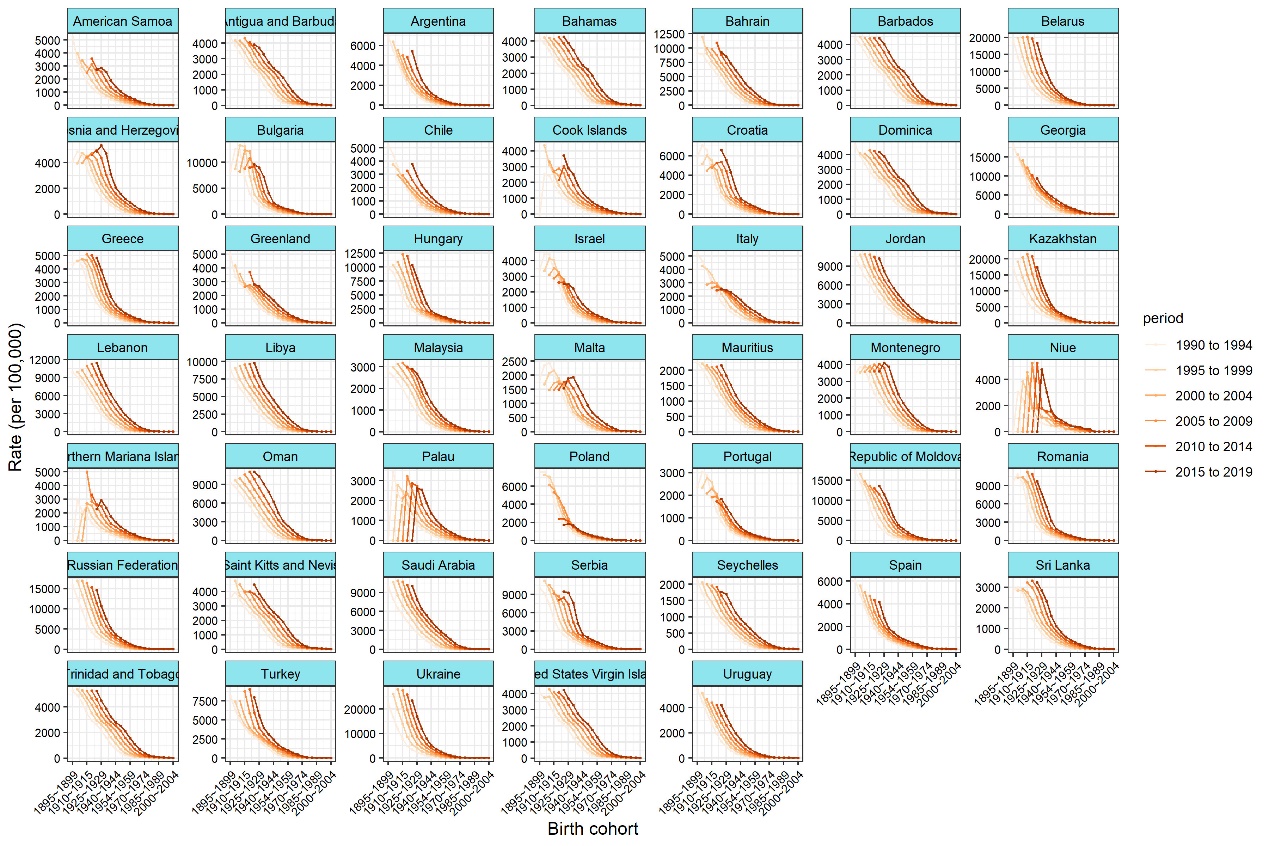


Figure S45. Ischemic heart disease incidence rates across different birth cohorts by period in high-middle SDI countries, 1990-2019.


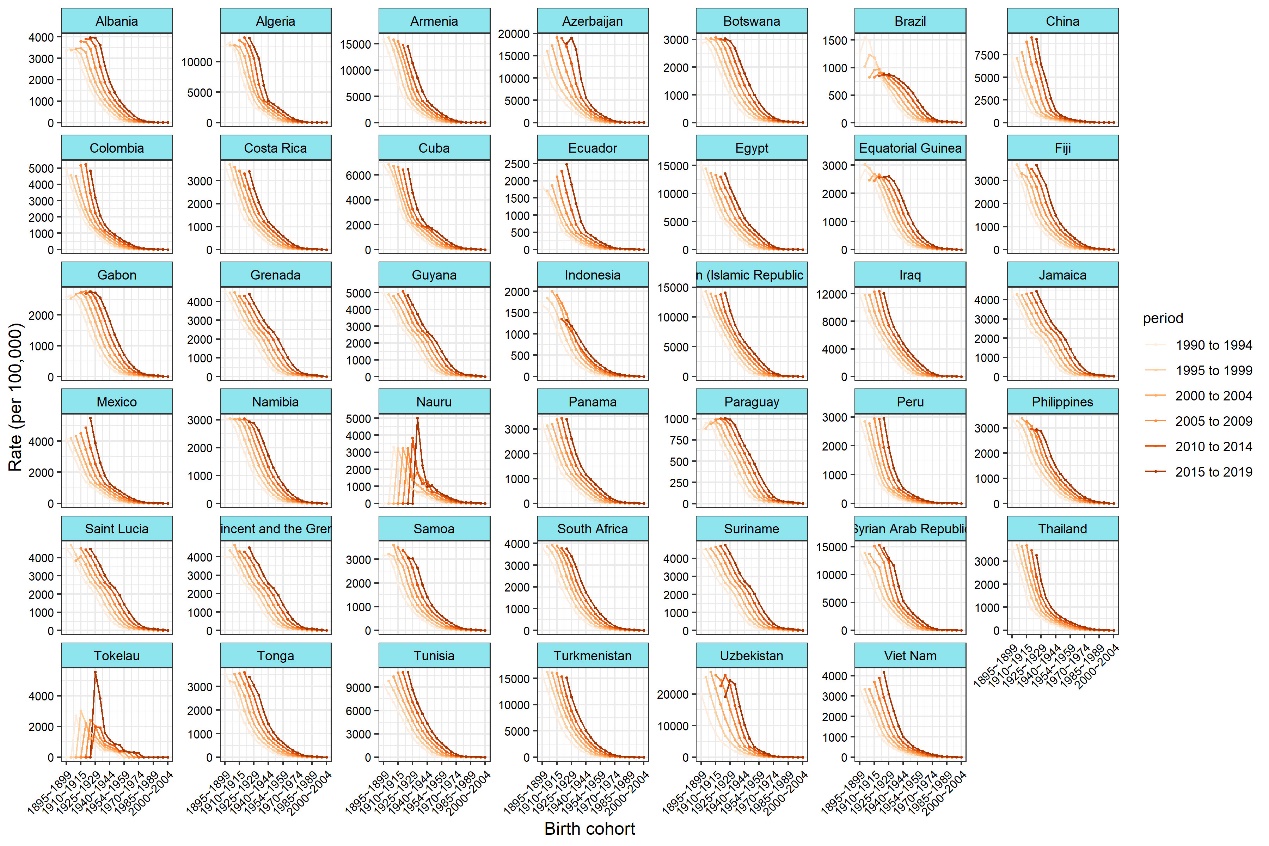


Figure S46. Ischemic heart disease incidence rates across different birth cohorts by period in middle-SDI countries, 1990-2019.


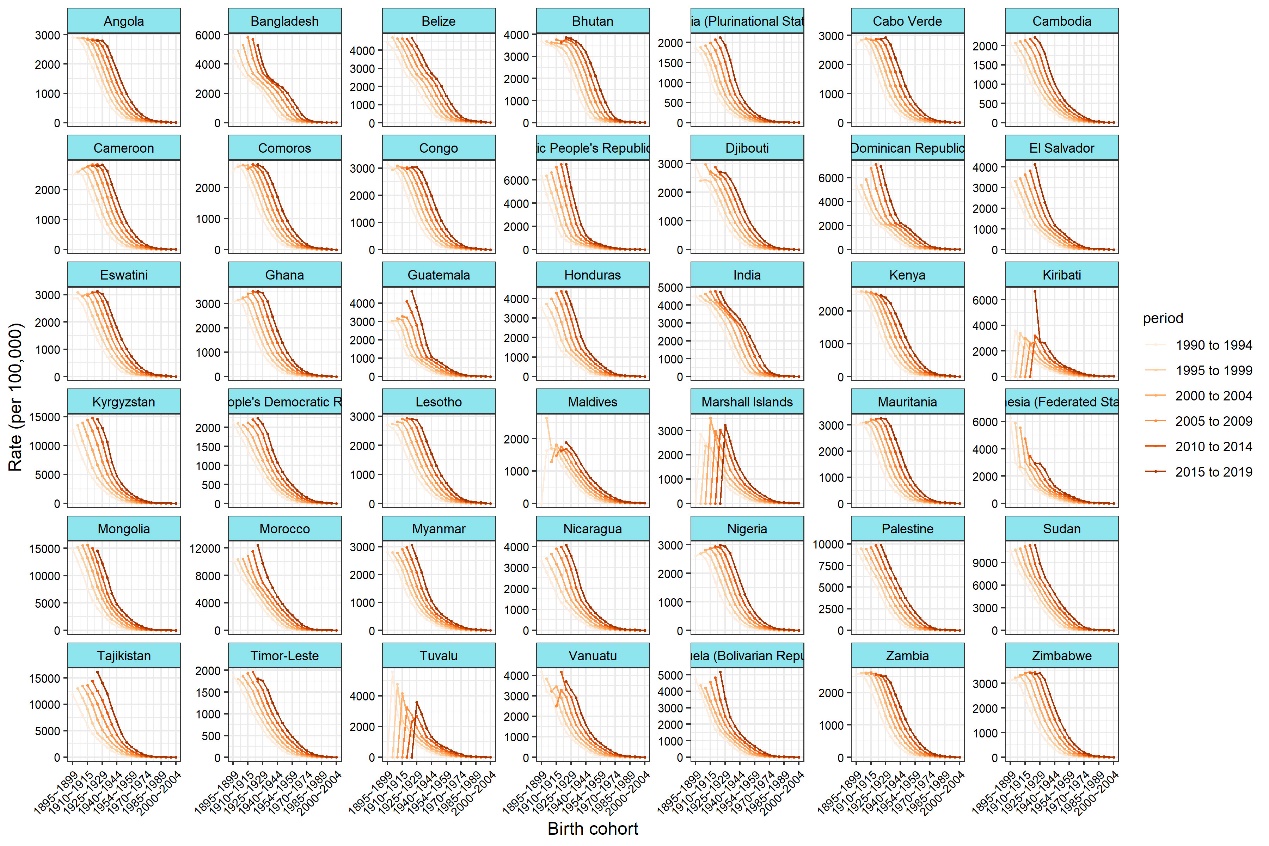


Figure S47. Ischemic heart disease incidence rates across different birth cohorts by period in low-middle SDI countries, 1990-2019.


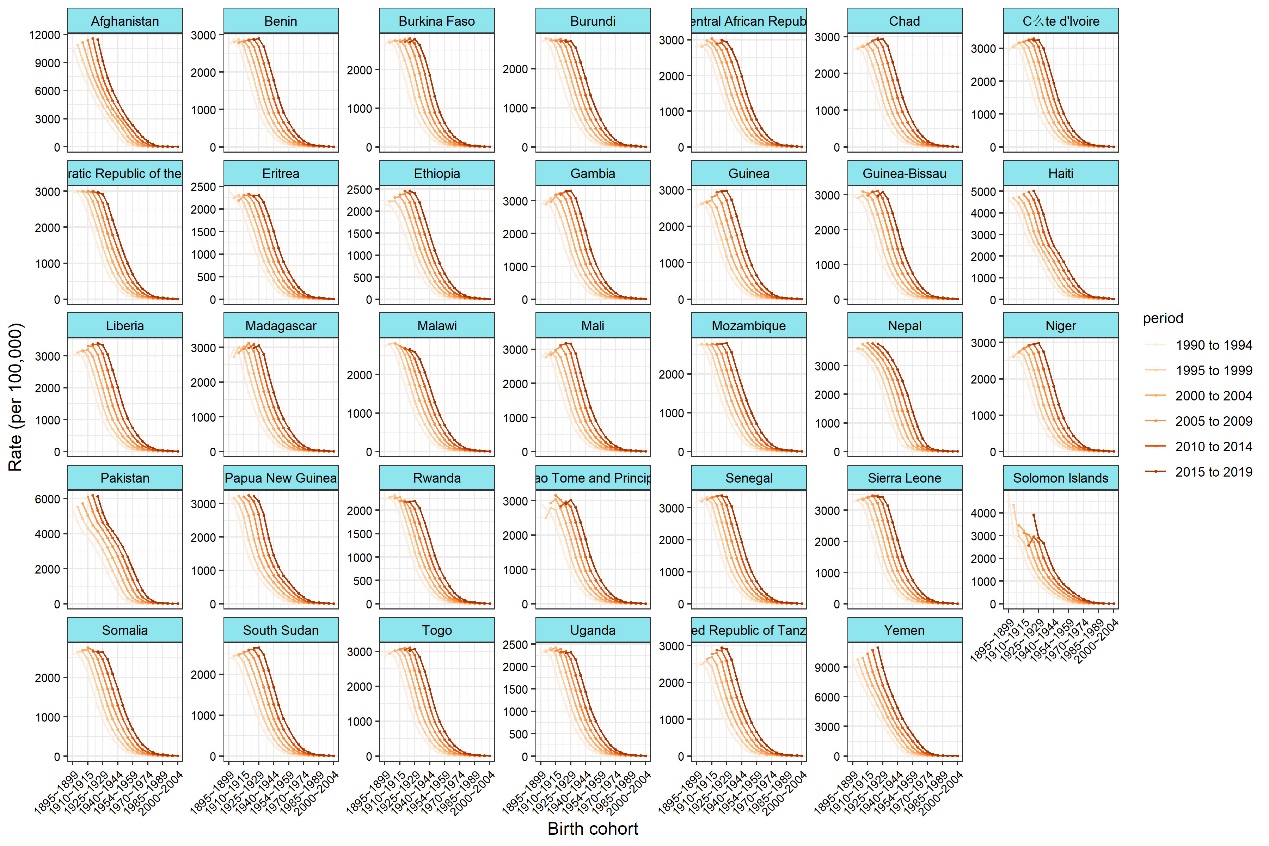


Figure S48. Ischemic heart disease incidence rates across different birth cohorts by period in low-SDI countries, 1990-2019.


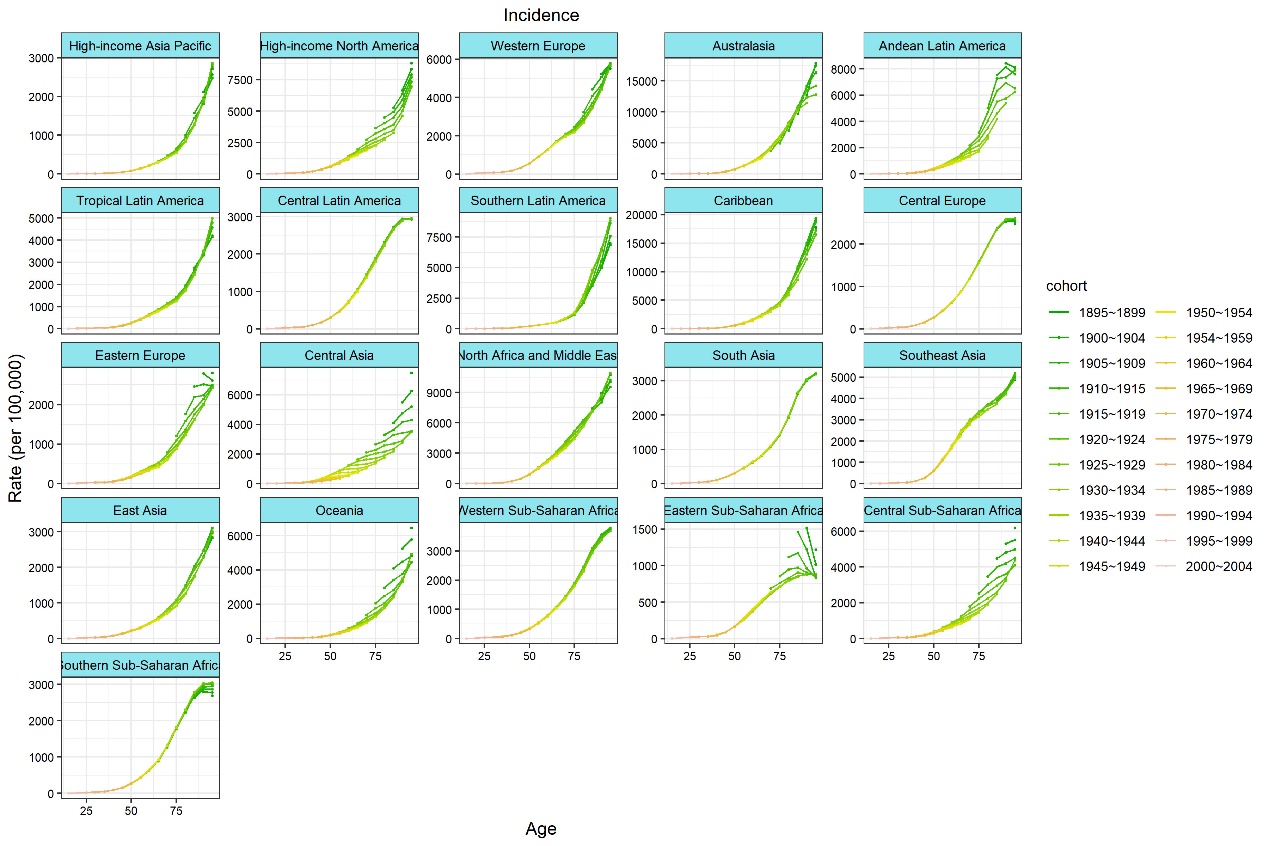


Figure S49. Ischemic heart disease incidence rates across different age groups by birth cohorts in the global, 1990-2019.


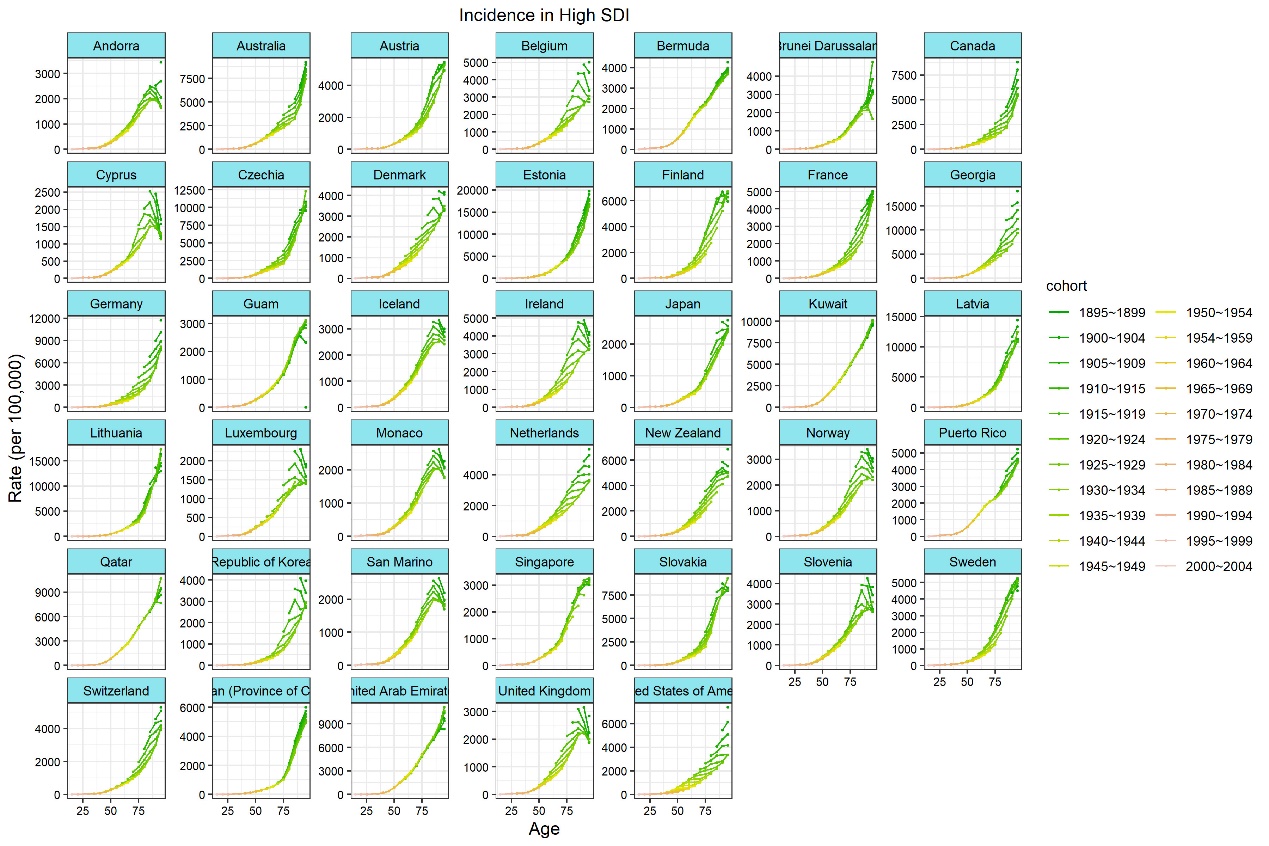


Figure S50. Ischemic heart disease incidence rates across different age groups by birth cohorts in high-SDI countries, 1990-2019.


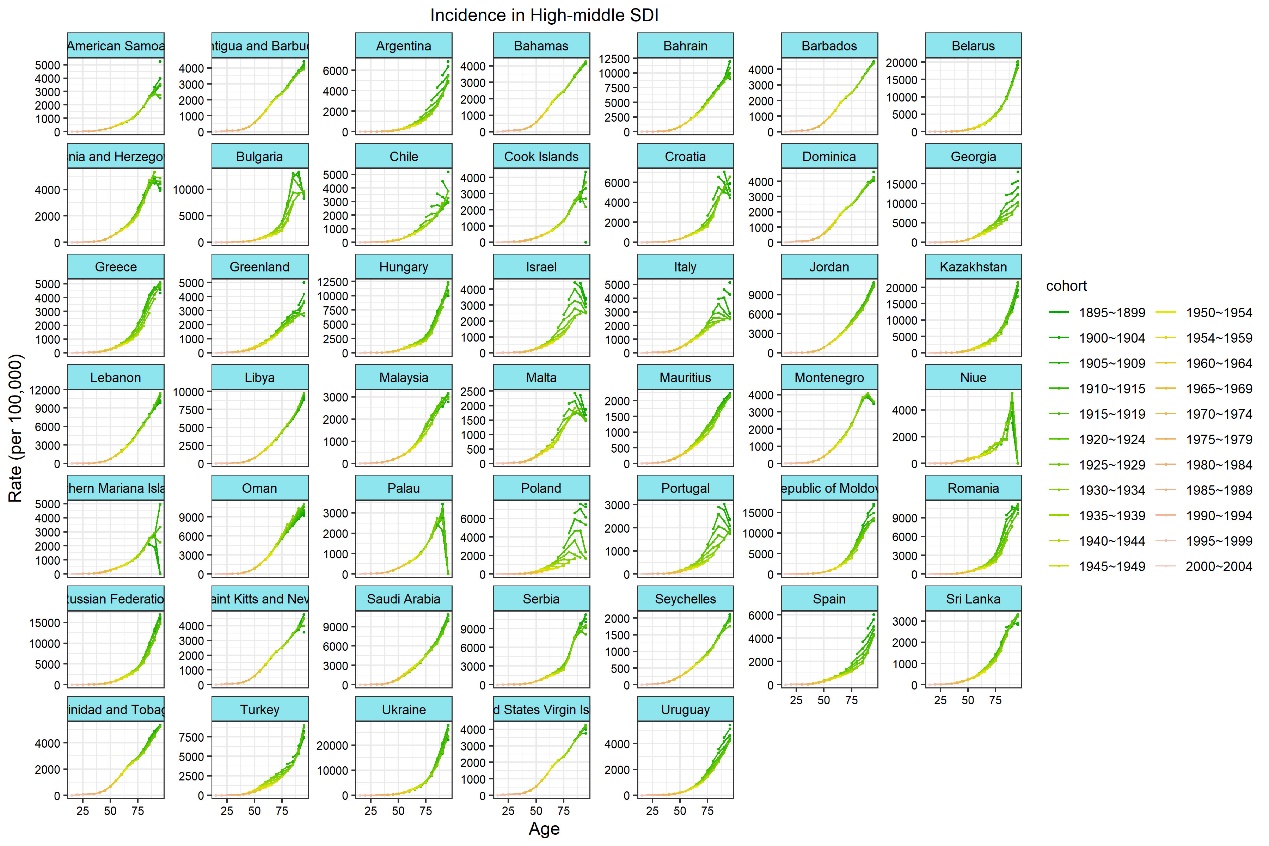


Figure S51. Ischemic heart disease incidence rates across different age groups by birth cohorts in high-middle SDI countries, 1990-2019.


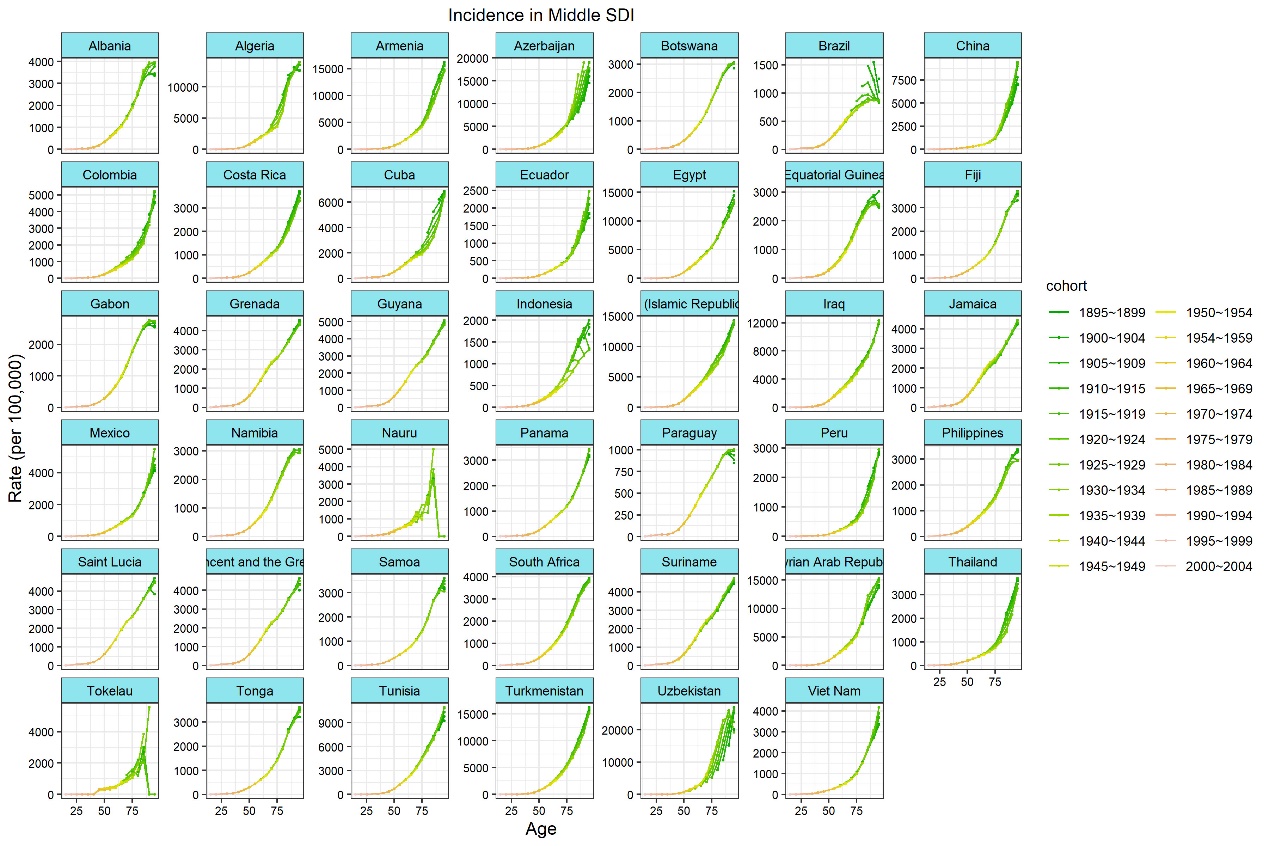


Figure S52. Ischemic heart disease incidence rates across different age groups by birth cohorts in middle-SDI countries, 1990-2019.


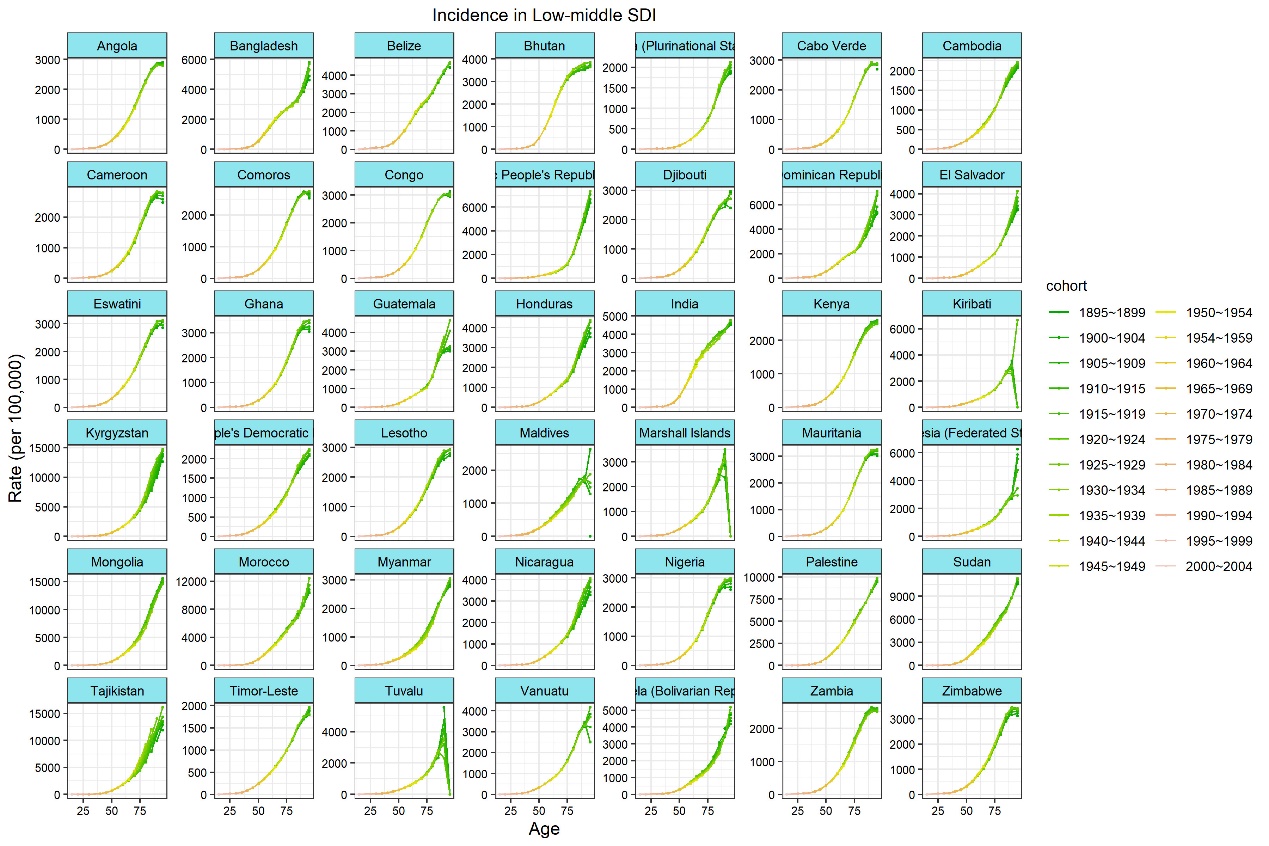


Figure S53. Ischemic heart disease incidence rates across different age groups by birth cohorts in low-middle SDI countries, 1990-2019.


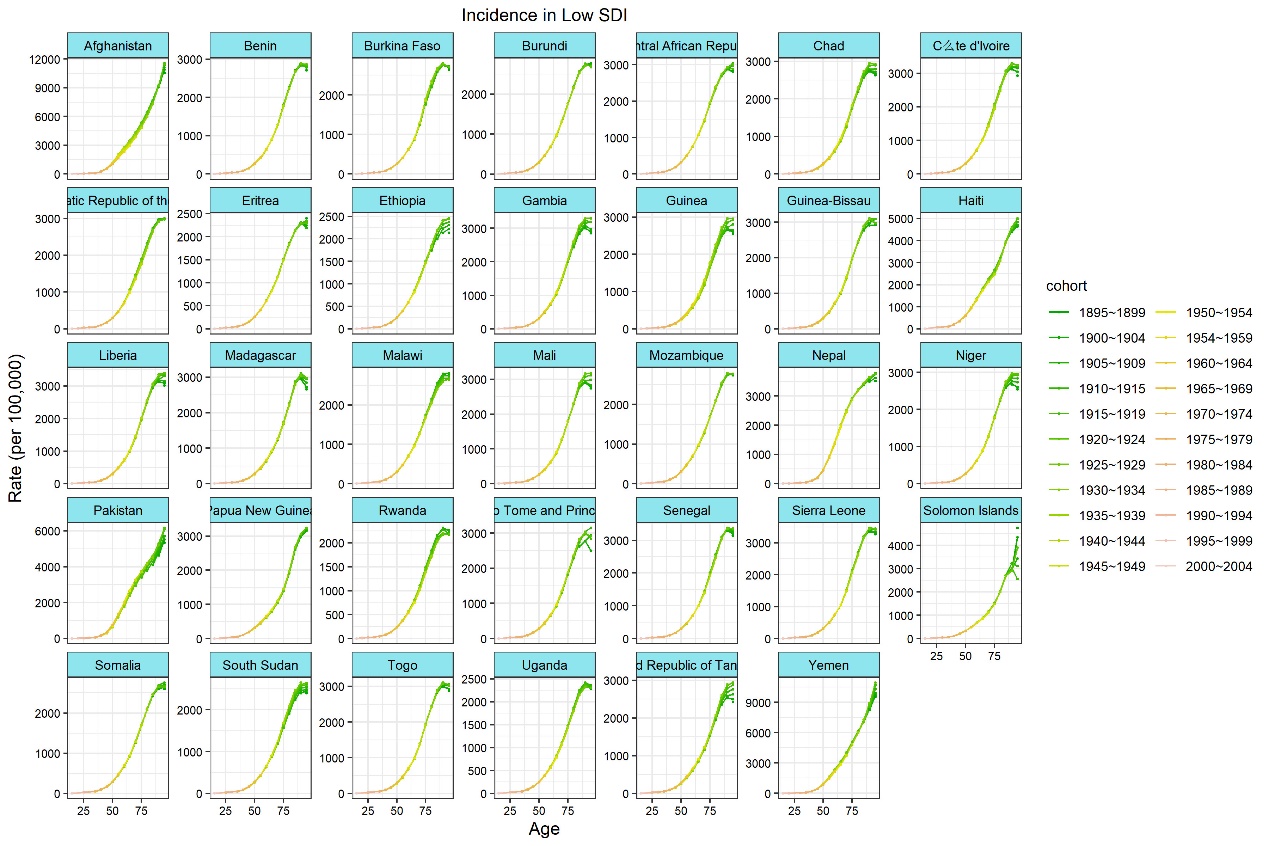


Figure S54. Ischemic heart disease incidence rates across different age groups by birth cohorts in low-SDI countries, 1990-2019.
