# Supplementary material for: Global, regional, and national time trends in ischaemic heart disease incidence over three decades (1990–2019): an age-period-cohort analysis of the global burden of disease study 2019
Source: Front Cardiovasc Med. 2024 Nov 1;11:1396380. doi: 10.3389/fcvm.2024.1396380 (PMC11563781; doi:10.3389/fcvm.2024.1396380)
Supplement: Supplementary file 2 [file Table1.docx]

Table S1. Trends in ischemic heart disease incidence for both sexes in 204 countries, 1990-2019

Age-standardized incidence rate is computed by direct standardization with global standard population in GBD 2019. Net drifts are estimates derived from the age-period-cohort model and denotes overall annual percentage change in incidence, which captures the contribution of the effects from calendar time and successive birth cohorts. SDI=Socio-demographic Index; APC=age-period-cohort;AAPCs=Average annual percentage changes.

| location | case_1990 | case_2019 | case_percent_change | ASR_1990 | ASR_2019 | ASR_percent_change | AAPC | netdrift |
| --- | --- | --- | --- | --- | --- | --- | --- | --- |
| Afghanistan | 49280 (44300, 55119) | 79901 (71092, 89426) | 62.13 (51.05, 73.18) | 728.15 (654.64, 806.76) | 672.22 (600.78, 751.08) | -7.68 (-12.61, -2.82) | -0.36 (-0.47, -0.24) | -0.29 (-0.35, -0.24) |
| Albania | 5251 (4509, 6025) | 10442 (9013, 12001) | 98.85 (87.82, 110.52) | 261.47 (224.96, 300.23) | 246.29 (214.02, 280.66) | -5.8 (-9.88, -1.12) | -0.22 (-0.29, -0.14) | -0.27 (-0.45, -0.09) |
| Algeria | 75154 (67094, 83681) | 179174 (159671, 199669) | 138.41 (125.92, 151.37) | 704.92 (639.53, 774.87) | 577.82 (525.25, 637.52) | -18.03 (-22.94, -13.55) | -0.7 (-0.79, -0.61) | -0.61 (-0.85, -0.37) |
| American Samoa | 43 (37, 49) | 92 (79, 105) | 115.06 (105.31, 126.64) | 195.14 (168.72, 223.29) | 200.79 (174.32, 228.58) | 2.89 (-1.08, 7.34) | 0.09 (0.08, 0.11) | 0.07 (-1.38, 1.55) |
| Andorra | 124 (106, 143) | 250 (217, 287) | 101.98 (90.03, 114.67) | 228.27 (196.54, 262.8) | 180.58 (156.09, 207.73) | -20.89 (-24.8, -16.89) | -0.85 (-0.9, -0.8) | -0.97 (-2.05, 0.13) |
| Angola | 8645 (7490, 9916) | 23998 (20799, 27295) | 177.6 (166.81, 188.65) | 236.59 (205.57, 270.77) | 225.41 (196.85, 256.49) | -4.72 (-8.6, -1.14) | -0.18 (-0.2, -0.16) | -0.25 (-0.36, -0.15) |
| Antigua and Barbuda | 202 (175, 232) | 375 (322, 429) | 85.2 (74.26, 96.49) | 379.47 (329.64, 434.84) | 370.42 (320.77, 423.4) | -2.39 (-6.45, 2.05) | -0.1 (-0.15, -0.04) | -0.12 (-0.83, 0.59) |
| Argentina | 73677 (66855, 81239) | 85335 (76873, 94583) | 15.82 (9.18, 23.66) | 241.36 (219.95, 265.42) | 156.85 (141.19, 173.67) | -35.01 (-38.76, -30.68) | -1.62 (-2.11, -1.14) | -1.23 (-1.38, -1.08) |
| Armenia | 15383 (13453, 17471) | 23143 (20339, 26172) | 50.44 (42.96, 58.06) | 626.07 (552.79, 702.87) | 566.14 (503.36, 635.54) | -9.57 (-13.39, -5.43) | -0.33 (-0.37, -0.28) | -0.32 (-0.44, -0.19) |
| Australia | 93253 (84251, 103315) | 143991 (126055, 163937) | 54.41 (43.46, 66.04) | 485.43 (438.22, 538.41) | 360.29 (315.78, 408.9) | -25.78 (-30.61, -20.48) | -1.06 (-1.18, -0.94) | -0.87 (-0.95, -0.79) |
| Austria | 33450 (30386, 37033) | 37747 (33979, 41734) | 12.85 (6.03, 20.75) | 283.82 (258.74, 313.79) | 215.43 (193.53, 239.22) | -24.1 (-28.47, -18.77) | -0.91 (-1.02, -0.81) | -0.78 (-0.88, -0.67) |
| Azerbaijan | 29604 (26270, 33303) | 56378 (50442, 63091) | 90.44 (81.08, 101.09) | 621.24 (557.59, 696.22) | 736.83 (675.14, 805.42) | 18.61 (12.47, 26.31) | 0.53 (0.35, 0.72) | 0.05 (-0.11, 0.21) |
| Bahamas | 605 (527, 690) | 1517 (1314, 1743) | 150.82 (139.53, 162.46) | 384.59 (334.61, 440.16) | 389.76 (338.13, 448.28) | 1.34 (-3.08, 5.78) | 0.03 (-0.05, 0.11) | 0.07 (-0.3, 0.45) |
| Bahrain | 1152 (993, 1316) | 6461 (5475, 7566) | 461.02 (425.92, 495.09) | 675.42 (583.42, 768.99) | 643.3 (556.88, 746.52) | -4.75 (-8.99, -0.26) | -0.19 (-0.24, -0.15) | -0.26 (-0.64, 0.12) |
| Bangladesh | 166916 (146874, 188099) | 481768 (429467, 539708) | 188.63 (170.08, 208.93) | 373.33 (329.2, 422.81) | 375.64 (337.24, 418.96) | 0.62 (-5.6, 7.72) | 0 (-0.14, 0.13) | 0.14 (0.11, 0.17) |
| Barbados | 1097 (949, 1269) | 1886 (1616, 2187) | 71.97 (62.75, 82.14) | 385.01 (335.84, 441.7) | 393.39 (340.93, 452.47) | 2.18 (-2.32, 6.82) | 0.09 (-0.07, 0.24) | 0.06 (-0.3, 0.42) |
| Belarus | 66663 (60716, 73214) | 87861 (81181, 95251) | 31.8 (25.54, 38.74) | 535.33 (490.9, 585.04) | 546.15 (503.44, 590.79) | 2.02 (-2.62, 6.88) | 0.08 (-0.11, 0.27) | -0.06 (-0.21, 0.09) |
| Belgium | 45897 (40956, 51341) | 43494 (38044, 49603) | -5.24 (-11.39, 2.05) | 300.09 (268.25, 334.46) | 197.53 (173.6, 224.05) | -34.17 (-37.99, -29.74) | -1.38 (-1.82, -0.94) | -1.08 (-1.31, -0.85) |
| Belize | 380 (329, 434) | 1184 (1024, 1352) | 211.4 (196.76, 225.97) | 398.97 (342.75, 457.66) | 421.2 (364.94, 480.34) | 5.57 (1.1, 10.39) | 0.14 (0.08, 0.2) | 0.17 (-0.25, 0.6) |
| Benin | 4174 (3615, 4741) | 10395 (9103, 11836) | 149.06 (137, 160.76) | 214.42 (185.13, 245.9) | 218.75 (189.88, 249.65) | 2.02 (-2.28, 6.22) | 0.05 (0, 0.1) | 0.12 (-0.02, 0.26) |
| Bermuda | 224 (193, 257) | 421 (362, 487) | 87.58 (77.87, 97.97) | 359.63 (309.33, 411.08) | 338.61 (294.28, 388.78) | -5.84 (-10.01, -0.97) | -0.24 (-0.3, -0.17) | -0.27 (-1.08, 0.54) |
| Bhutan | 902 (767, 1041) | 2160 (1857, 2493) | 139.53 (125.26, 154.13) | 391.74 (334.79, 454.02) | 395.08 (341.54, 456.46) | 0.85 (-4.46, 6.07) | 0.03 (0.01, 0.04) | 0 (-0.38, 0.38) |
| Bolivia (Plurinational State of) | 2606 (2252, 2969) | 7386 (6388, 8460) | 183.38 (169.99, 198.6) | 87.51 (75.37, 100.11) | 89.53 (77.65, 102.17) | 2.31 (-2.61, 7.48) | 0.14 (-0.38, 0.67) | 0.03 (-0.15, 0.2) |
| Bosnia and Herzegovina | 12160 (10562, 13924) | 16922 (14731, 19385) | 39.17 (31.58, 47.68) | 320.77 (282.84, 361.6) | 293.72 (257.81, 332.71) | -8.43 (-12.15, -4.11) | -0.36 (-0.49, -0.24) | -0.36 (-0.5, -0.21) |
| Botswana | 1251 (1075, 1425) | 3103 (2701, 3527) | 148.13 (137.17, 158.97) | 227.25 (196.04, 261.22) | 232.75 (203.47, 265.17) | 2.42 (-1.99, 6.77) | 0.08 (0.02, 0.15) | 0.12 (-0.15, 0.39) |
| Brazil | 109326 (96373, 123064) | 260661 (230100, 293617) | 138.43 (131.65, 145.33) | 125.34 (110.39, 140.81) | 109.94 (97.07, 123.55) | -12.29 (-14.85, -9.71) | -0.51 (-0.61, -0.4) | -0.47 (-0.55, -0.39) |
| Brunei Darussalam | 177 (155, 202) | 455 (394, 521) | 156.62 (143.5, 170.16) | 178.4 (154.99, 204.22) | 155.2 (133.9, 178.04) | -13.01 (-16.66, -9.21) | -0.5 (-0.54, -0.45) | -0.48 (-1.26, 0.31) |
| Bulgaria | 49291 (44640, 54128) | 47610 (43105, 52197) | -3.41 (-8.28, 2.8) | 451.37 (416.42, 489.09) | 332.72 (302.19, 364.93) | -26.29 (-29.97, -22.26) | -1.33 (-1.92, -0.75) | -0.95 (-1.16, -0.74) |
| Burkina Faso | 8285 (7145, 9477) | 18430 (16046, 20783) | 122.44 (110.21, 133.91) | 203.99 (177.01, 234.16) | 212.02 (184.47, 240.66) | 3.94 (-1.18, 9.06) | 0.08 (-0.01, 0.17) | 0.1 (-0.01, 0.21) |
| Burundi | 4925 (4301, 5627) | 9914 (8539, 11383) | 101.31 (90.69, 112.67) | 216.99 (187.78, 250.52) | 224.31 (193.68, 257.69) | 3.38 (-1, 8.01) | 0.1 (0.09, 0.12) | 0.06 (-0.08, 0.21) |
| Cabo Verde | 485 (415, 560) | 925 (804, 1060) | 90.83 (79.72, 103.9) | 205.91 (177.82, 237.57) | 216.38 (187.05, 248.85) | 5.08 (0.64, 10.5) | 0.17 (0.15, 0.2) | 0.2 (-0.27, 0.67) |
| Cambodia | 6815 (5962, 7734) | 17133 (14952, 19514) | 151.41 (141.26, 163.98) | 153.44 (133.63, 174.4) | 147.25 (129.39, 166.81) | -4.03 (-7.97, 0.52) | -0.13 (-0.32, 0.07) | -0.17 (-0.28, -0.06) |
| Cameroon | 7977 (6903, 9094) | 24075 (21024, 27258) | 201.8 (188.2, 217.22) | 191.09 (164.77, 218.41) | 209.98 (182.23, 236.99) | 9.88 (4.75, 15.76) | 0.32 (0.27, 0.37) | 0.41 (0.31, 0.51) |
| Canada | 111981 (101219, 123375) | 130013 (115147, 146441) | 16.1 (8.77, 23.32) | 350.04 (316.28, 385.18) | 196.05 (173.52, 220.72) | -43.99 (-47.38, -40.84) | -2.06 (-2.19, -1.94) | -1.75 (-1.81, -1.69) |
| Central African Republic | 2627 (2257, 2998) | 4985 (4298, 5718) | 89.76 (81.45, 98.09) | 243.77 (211.11, 278.75) | 248.13 (214.7, 285.98) | 1.79 (-2.44, 6.7) | 0.05 (0.05, 0.06) | 0.06 (-0.15, 0.26) |
| Chad | 5557 (4813, 6366) | 12090 (10564, 13695) | 117.57 (106.96, 128.33) | 204.35 (176.57, 235.2) | 223.77 (195.01, 256.83) | 9.5 (4.78, 14.53) | 0.29 (0.26, 0.33) | 0.34 (0.21, 0.47) |
| Chile | 20901 (18382, 23496) | 41144 (35831, 47245) | 96.85 (86.25, 109.24) | 224.28 (197.4, 252.61) | 172.52 (150.56, 197.63) | -23.08 (-27.04, -18.22) | -0.96 (-1.16, -0.76) | -0.81 (-0.98, -0.64) |
| China | 1262226 (1111162, 1421101) | 3500281 (3086107, 3939530) | 177.31 (166.99, 188.52) | 177.1 (156.84, 199.19) | 197.39 (175.75, 222.36) | 11.46 (9.44, 13.58) | 0.38 (0.32, 0.44) | 0.29 (0.21, 0.36) |
| Colombia | 36731 (32608, 41090) | 91324 (81140, 102931) | 148.63 (137.19, 160.61) | 217.36 (193.56, 243.77) | 169.68 (150.74, 191.01) | -21.93 (-25.62, -18.01) | -0.89 (-1, -0.78) | -0.74 (-0.79, -0.68) |
| Comoros | 471 (407, 544) | 1023 (890, 1170) | 117.11 (107.79, 126.9) | 218.97 (188.26, 252.19) | 214.75 (185.43, 246.25) | -1.92 (-5.87, 2.68) | -0.07 (-0.09, -0.06) | -0.09 (-0.55, 0.37) |
| Congo | 2474 (2136, 2842) | 6140 (5313, 6955) | 148.19 (136.51, 161.9) | 246.96 (213.7, 283.87) | 245.81 (212.96, 280.61) | -0.47 (-4.28, 3.83) | -0.02 (-0.04, 0) | -0.07 (-0.3, 0.16) |
| Cook Islands | 23 (20, 27) | 46 (40, 53) | 97.42 (87.33, 108.2) | 192.31 (167.07, 219.93) | 191.68 (165.4, 219.25) | -0.33 (-4.65, 4.14) | -0.02 (-0.04, 0) | -0.05 (-2.31, 2.25) |
| Costa Rica | 3340 (2907, 3794) | 9136 (7899, 10481) | 173.56 (161.31, 185.03) | 190.32 (164.97, 217.17) | 176.75 (153.34, 202.23) | -7.13 (-10.99, -3.08) | -0.27 (-0.31, -0.24) | -0.3 (-0.46, -0.15) |
| Croatia | 20229 (18124, 22550) | 21547 (19223, 24141) | 6.51 (0.84, 12.84) | 335.81 (303.99, 372.5) | 249.5 (223.51, 278.16) | -25.7 (-29.2, -21.84) | -1.04 (-1.4, -0.68) | -0.78 (-1.06, -0.5) |
| Cuba | 40393 (36009, 44894) | 61056 (53608, 68517) | 51.15 (43.8, 58.92) | 395.86 (353.31, 440.44) | 327.46 (289.23, 366.18) | -17.28 (-21.36, -13.44) | -0.65 (-0.75, -0.55) | -0.56 (-0.64, -0.47) |
| Cyprus | 1339 (1143, 1552) | 2504 (2148, 2868) | 86.93 (76.57, 97.85) | 169.7 (147.17, 194.86) | 132.09 (113.99, 150.48) | -22.16 (-26.06, -18.04) | -1.01 (-1.38, -0.63) | -1.04 (-1.44, -0.64) |
| Czechia | 70558 (65033, 77011) | 73703 (66031, 82082) | 4.46 (-2.32, 11.78) | 526.59 (485.02, 573.57) | 364.17 (325.03, 405.19) | -30.84 (-35.21, -26.4) | -1.41 (-1.65, -1.17) | -1.27 (-1.4, -1.13) |
| C么te d'Ivoire | 8893 (7735, 10141) | 24686 (21643, 28105) | 177.57 (165.69, 188.65) | 235.63 (203.56, 268.06) | 240.82 (209.4, 272.57) | 2.2 (-2.31, 6.32) | 0.07 (0.02, 0.12) | 0.09 (-0.01, 0.19) |
| Democratic People's Republic of Korea | 22143 (19496, 25058) | 53583 (47813, 59712) | 141.99 (128.69, 156.06) | 164.85 (145.56, 186.46) | 182.09 (164.35, 201.81) | 10.46 (4.68, 17.29) | 0.32 (0.25, 0.39) | 0.38 (0.31, 0.45) |
| Democratic Republic of the Congo | 34706 (30153, 39311) | 77445 (67858, 87027) | 123.14 (112.49, 134.53) | 236.33 (206.82, 265.92) | 226.14 (199.26, 253.91) | -4.31 (-9.05, 0.38) | -0.18 (-0.21, -0.15) | -0.18 (-0.24, -0.12) |
| Denmark | 27261 (25757, 29009) | 22683 (19542, 25964) | -16.79 (-26.65, -6.79) | 344.56 (326.27, 365.94) | 207.38 (179.37, 235.86) | -39.81 (-46.24, -33.19) | -1.86 (-1.99, -1.74) | -1.46 (-1.63, -1.28) |
| Djibouti | 286 (250, 327) | 1284 (1111, 1465) | 348.33 (326.46, 367.94) | 206.17 (178.03, 237.76) | 221.82 (191.04, 254.98) | 7.59 (2.55, 12.17) | 0.25 (0.23, 0.26) | 0.21 (-0.34, 0.76) |
| Dominica | 267 (231, 307) | 349 (302, 403) | 30.9 (23.7, 38.08) | 379.02 (330.59, 433.8) | 391.97 (340.52, 450) | 3.42 (-1.14, 7.95) | 0.1 (0.04, 0.16) | 0.13 (-0.61, 0.87) |
| Dominican Republic | 13359 (11561, 15293) | 35108 (30976, 39457) | 162.81 (148.01, 176.61) | 352.89 (305.96, 404.12) | 378.48 (333.9, 426.73) | 7.25 (0.93, 13.17) | 0.24 (0.04, 0.45) | 0.31 (0.24, 0.39) |
| Ecuador | 4093 (3587, 4645) | 11797 (10426, 13337) | 188.25 (172.98, 202.03) | 79.34 (69.29, 90.47) | 81.27 (72.14, 91.68) | 2.43 (-2.71, 7.48) | 0.01 (-0.37, 0.39) | -0.01 (-0.15, 0.14) |
| Egypt | 208528 (190882, 228246) | 450548 (411983, 491750) | 116.06 (104.52, 128.79) | 782.15 (723.68, 848.99) | 759.93 (705.89, 819.39) | -2.84 (-7.9, 2.35) | -0.16 (-0.38, 0.06) | -0.03 (-0.1, 0.04) |
| El Salvador | 4966 (4320, 5650) | 10582 (9286, 11954) | 113.07 (103.86, 122.38) | 168.16 (145.15, 192.18) | 173.79 (151.59, 196.44) | 3.35 (-1.21, 7.58) | 0.07 (-0.04, 0.17) | 0.1 (-0.04, 0.24) |
| Equatorial Guinea | 435 (377, 501) | 1036 (906, 1175) | 137.96 (126.86, 150.05) | 237.24 (205.09, 274.57) | 213.68 (184.54, 245.44) | -9.93 (-13.85, -5.88) | -0.38 (-0.41, -0.35) | -0.47 (-0.94, 0.01) |
| Eritrea | 1819 (1570, 2082) | 4978 (4313, 5670) | 173.63 (162.16, 184.77) | 194.75 (167.68, 223.7) | 192.65 (166.84, 220.47) | -1.08 (-5.12, 3.3) | -0.04 (-0.05, -0.02) | -0.06 (-0.31, 0.2) |
| Estonia | 12690 (11390, 14158) | 15950 (13797, 18196) | 25.69 (16.3, 36.6) | 642.63 (581.81, 714.56) | 594.61 (518.24, 675.19) | -7.47 (-14.31, -0.22) | -0.24 (-0.35, -0.12) | -0.07 (-0.24, 0.1) |
| Eswatini | 663 (574, 759) | 1329 (1154, 1515) | 100.3 (92.36, 109.2) | 231.17 (199.34, 265.41) | 236.34 (203.82, 270.89) | 2.24 (-1.72, 6.68) | 0.09 (0.04, 0.13) | 0.17 (-0.2, 0.53) |
| Ethiopia | 34336 (29728, 39271) | 77896 (68401, 88330) | 126.86 (119.91, 134.61) | 183.53 (159.75, 209.44) | 188.88 (164.5, 215.48) | 2.91 (0.43, 5.43) | 0.09 (0.06, 0.13) | 0.1 (0.04, 0.16) |
| Fiji | 742 (643, 847) | 1490 (1283, 1707) | 100.76 (90.92, 110.84) | 215.46 (186.13, 247.97) | 213.62 (185.95, 243.99) | -0.85 (-5.02, 3.41) | 0 (-0.17, 0.17) | -0.02 (-0.39, 0.36) |
| Finland | 28612 (25560, 31879) | 29663 (26215, 33421) | 3.67 (-2.84, 10.68) | 405.18 (363.17, 452.6) | 247.91 (219.4, 279.03) | -38.81 (-42.28, -35.32) | -1.84 (-1.96, -1.73) | -1.69 (-1.89, -1.5) |
| France | 218032 (196024, 240168) | 227286 (202317, 253721) | 4.24 (-1.8, 11.53) | 261.39 (235.86, 288.36) | 170.47 (151.37, 191.33) | -34.78 (-38.34, -30.7) | -1.51 (-1.61, -1.42) | -1.26 (-1.35, -1.17) |
| Gabon | 1182 (1014, 1366) | 2283 (1968, 2606) | 93.11 (83.64, 101.94) | 220.94 (190.52, 254.51) | 225.92 (193.14, 258.43) | 2.26 (-2.3, 6.69) | 0.08 (0.06, 0.11) | 0.09 (-0.2, 0.38) |
| Gambia | 817 (710, 938) | 2415 (2106, 2755) | 195.48 (181.26, 210.01) | 238.91 (206.01, 275.44) | 254.71 (219.49, 292.5) | 6.62 (1.71, 11.77) | 0.21 (0.2, 0.23) | 0.27 (-0.06, 0.6) |
| Georgia | 39290 (35017, 43591) | 30816 (27675, 34129) | -21.57 (-25.85, -17.11) | 692.36 (626.14, 759.28) | 506.96 (454.27, 559.64) | -26.78 (-30.58, -22.56) | -1.13 (-1.25, -1.02) | -0.73 (-0.9, -0.57) |
| Germany | 616174 (572340, 663978) | 458738 (419827, 498662) | -25.55 (-30.59, -19.6) | 484.56 (449.35, 522.96) | 242.94 (220.93, 264.97) | -49.86 (-53.07, -46.37) | -2.31 (-2.62, -2) | -2.03 (-2.16, -1.9) |
| Ghana | 13190 (11394, 15014) | 35839 (31442, 40442) | 171.71 (158.61, 184.82) | 224.68 (193.27, 255.07) | 233.91 (205.04, 263.21) | 4.11 (-1.16, 9.2) | 0.14 (0.11, 0.17) | 0.2 (0.12, 0.28) |
| Greece | 38784 (34277, 43462) | 45114 (40132, 50537) | 16.32 (10.05, 23.23) | 259.11 (231.34, 289.84) | 190.41 (169.35, 212.16) | -26.51 (-30.06, -22.49) | -1.05 (-1.32, -0.77) | -0.89 (-0.98, -0.81) |
| Greenland | 104 (90, 119) | 148 (127, 171) | 42.49 (34.7, 50.68) | 301.31 (262.51, 345.3) | 222.6 (193.47, 254.51) | -26.12 (-29.09, -22.82) | -1.06 (-1.11, -1.02) | -0.97 (-2.11, 0.19) |
| Grenada | 285 (247, 328) | 459 (394, 521) | 60.88 (51.06, 70.86) | 395.46 (343.23, 453.8) | 411.75 (356.54, 466.39) | 4.12 (-0.47, 9.01) | 0.14 (0.1, 0.17) | 0.13 (-0.5, 0.76) |
| Guam | 125 (108, 143) | 354 (307, 404) | 183.86 (165.93, 202.93) | 168.91 (145.81, 192.41) | 188.82 (164.23, 215.34) | 11.79 (7.19, 16.83) | 0.4 (0.37, 0.43) | 0.41 (-0.55, 1.39) |
| Guatemala | 5728 (4984, 6507) | 18902 (16522, 21450) | 230 (213.44, 247.03) | 167.04 (145.36, 189.61) | 174.92 (152.65, 198.38) | 4.72 (-0.23, 9.82) | 0.19 (-0.02, 0.41) | 0.21 (0.09, 0.34) |
| Guinea | 6138 (5298, 7051) | 11687 (10158, 13257) | 90.41 (81.16, 100.22) | 194.53 (168.09, 223.16) | 218.62 (189.21, 249.29) | 12.38 (7.11, 17.51) | 0.38 (0.34, 0.42) | 0.52 (0.39, 0.65) |
| Guinea-Bissau | 883 (764, 1003) | 1681 (1463, 1904) | 90.35 (81.15, 99.32) | 232.84 (201.39, 266.64) | 243.18 (209.82, 277.67) | 4.44 (-0.22, 9.24) | 0.14 (0.12, 0.16) | 0.23 (-0.12, 0.58) |
| Guyana | 1685 (1459, 1914) | 2647 (2290, 3049) | 57.08 (50.15, 64.52) | 433.39 (374.98, 495.88) | 423.12 (367.68, 482.82) | -2.37 (-6.4, 2.11) | -0.07 (-0.16, 0.01) | -0.07 (-0.32, 0.18) |
| Haiti | 13235 (11456, 14908) | 27826 (24566, 31596) | 110.24 (102.06, 120.73) | 409.5 (359.67, 459.33) | 393.68 (348.75, 443.97) | -3.87 (-7.53, 0.61) | -0.18 (-0.22, -0.13) | -0.12 (-0.21, -0.03) |
| Honduras | 3889 (3385, 4461) | 12072 (10506, 13711) | 210.45 (196.71, 225.43) | 190.97 (165.47, 218.88) | 206.79 (180.15, 235.88) | 8.28 (3.35, 14.02) | 0.25 (0.12, 0.39) | 0.15 (0.01, 0.28) |
| Hungary | 60750 (55370, 66660) | 62412 (56705, 68974) | 2.74 (-2.48, 8.31) | 431.4 (395.22, 472.67) | 325.81 (294.93, 360.03) | -24.48 (-28.18, -20.6) | -1.01 (-1.22, -0.8) | -1.03 (-1.11, -0.95) |
| Iceland | 815 (705, 933) | 1175 (1009, 1354) | 44.15 (36.94, 50.75) | 287.94 (249.28, 328.85) | 218.8 (189.08, 251.76) | -24.01 (-27.5, -20.67) | -0.94 (-1.14, -0.74) | -0.79 (-1.23, -0.35) |
| India | 1808643 (1555281, 2061069) | 4742991 (4139933, 5365446) | 162.24 (155.56, 168.69) | 431.06 (375.58, 488.2) | 427.41 (373.08, 482.75) | -0.85 (-2.05, 0.46) | -0.15 (-0.54, 0.23) | 0.04 (-0.07, 0.15) |
| Indonesia | 91757 (78227, 106070) | 178374 (155170, 202466) | 94.4 (86.71, 102.21) | 97.81 (83.97, 112.38) | 84.48 (74.16, 95.31) | -13.64 (-17.06, -10.04) | -0.52 (-2.13, 1.13) | -0.52 (-0.62, -0.42) |
| Iran (Islamic Republic of) | 218181 (187231, 250909) | 593001 (515830, 676050) | 171.79 (163.74, 180.39) | 898.31 (783.95, 1026.45) | 829.14 (719.94, 945.22) | -7.7 (-9.48, -5.95) | -0.24 (-0.35, -0.14) | -0.32 (-0.37, -0.27) |
| Iraq | 52300 (46753, 58385) | 140746 (125823, 156094) | 169.11 (155.44, 183.58) | 705.34 (633.1, 787.44) | 644.43 (582.41, 711.6) | -8.64 (-13.01, -4.28) | -0.34 (-0.39, -0.29) | -0.35 (-0.39, -0.31) |
| Ireland | 13669 (12031, 15363) | 14498 (12588, 16566) | 6.06 (-0.64, 13.67) | 337.31 (298.64, 378.4) | 199 (172.96, 227.88) | -41 (-44.58, -37.26) | -1.84 (-2.12, -1.56) | -1.79 (-1.91, -1.67) |
| Israel | 12861 (11206, 14482) | 18848 (16182, 21428) | 46.56 (37.45, 57.15) | 267.13 (233.9, 299.39) | 165.71 (143.01, 188.5) | -37.97 (-41.46, -33.94) | -1.63 (-2.07, -1.18) | -1.44 (-1.55, -1.33) |
| Italy | 252492 (216599, 289655) | 319847 (282760, 359927) | 26.68 (20.93, 33.02) | 290.68 (252.44, 329.85) | 241.14 (213.07, 270.21) | -17.04 (-20.78, -12.56) | -0.55 (-0.66, -0.44) | -0.66 (-0.84, -0.49) |
| Jamaica | 6541 (5688, 7490) | 12208 (10628, 13916) | 86.66 (77.43, 96.11) | 366.26 (317.31, 419.67) | 404.37 (350.73, 462.08) | 10.4 (5.64, 15.18) | 0.34 (0.09, 0.6) | 0.4 (0.28, 0.52) |
| Japan | 250546 (216681, 286152) | 436411 (373003, 503862) | 74.18 (64.02, 84.36) | 153.26 (133.38, 174.33) | 126.3 (109.72, 143.96) | -17.59 (-19.14, -16.16) | -0.67 (-0.83, -0.51) | -0.76 (-0.87, -0.65) |
| Jordan | 8011 (6926, 9194) | 37937 (32682, 43525) | 373.59 (351.82, 395.38) | 641.66 (555.09, 731.87) | 603.64 (522.47, 687.55) | -5.92 (-10.24, -1.78) | -0.19 (-0.31, -0.07) | -0.26 (-0.36, -0.16) |
| Kazakhstan | 69320 (62365, 76400) | 82285 (73039, 92439) | 18.7 (13.26, 24.67) | 589.62 (534.51, 645.52) | 523.92 (472.88, 581.99) | -11.14 (-15.33, -7.06) | -0.51 (-0.68, -0.33) | -0.72 (-0.8, -0.64) |
| Kenya | 16638 (14490, 19000) | 44462 (38818, 50655) | 167.24 (162.81, 172.53) | 205.07 (177.05, 235.54) | 204.36 (176.88, 234.27) | -0.35 (-1.08, 0.48) | -0.01 (-0.03, 0.02) | 0 (-0.07, 0.07) |
| Kiribati | 70 (61, 80) | 134 (116, 153) | 91.2 (82.19, 100.43) | 204.92 (177.79, 234.73) | 209.71 (182.66, 238.7) | 2.34 (-1.85, 6.68) | 0.06 (-0.01, 0.13) | 0.08 (-1.24, 1.42) |
| Kuwait | 3815 (3325, 4349) | 16837 (14644, 19359) | 341.29 (322.59, 362.48) | 634.6 (546.48, 731.26) | 648.35 (559.18, 747.07) | 2.17 (-2.41, 6.84) | 0.08 (-0.02, 0.17) | -0.02 (-0.16, 0.12) |
| Kyrgyzstan | 16155 (14084, 18451) | 24371 (21534, 27313) | 50.86 (42.44, 60.31) | 546.72 (477.88, 620.11) | 589.32 (526.17, 656.25) | 7.79 (2.13, 14.48) | 0.26 (0.2, 0.32) | 0.07 (-0.03, 0.17) |
| Lao People's Democratic Republic | 3321 (2909, 3812) | 6715 (5848, 7613) | 102.21 (92.11, 112.58) | 163.52 (143.34, 187.41) | 154.99 (134.83, 175.62) | -5.22 (-10.07, -0.54) | -0.2 (-0.26, -0.15) | -0.23 (-0.41, -0.06) |
| Latvia | 17105 (15219, 19079) | 17764 (15690, 20062) | 3.85 (-2.55, 10.15) | 486.78 (436.11, 541.11) | 432.51 (382.37, 486.07) | -11.15 (-16.01, -6.09) | -0.51 (-0.76, -0.26) | -0.51 (-0.69, -0.33) |
| Lebanon | 13671 (11935, 15541) | 32025 (28355, 35958) | 134.25 (122.24, 145.97) | 641.67 (566.01, 724.82) | 619.68 (547.83, 696.09) | -3.43 (-7.68, 0.75) | -0.09 (-0.27, 0.09) | 0.04 (-0.08, 0.15) |
| Lesotho | 1990 (1728, 2270) | 2748 (2366, 3121) | 38.07 (32.22, 44.44) | 209.58 (182.2, 239.94) | 225.97 (194.3, 257.46) | 7.82 (2.79, 12.67) | 0.27 (0.25, 0.29) | 0.3 (0.07, 0.54) |
| Liberia | 2494 (2149, 2863) | 4963 (4311, 5623) | 98.96 (86.69, 112.25) | 236.97 (204.75, 271.78) | 248 (215.04, 283.45) | 4.65 (-0.97, 9.64) | 0.14 (0.09, 0.18) | 0.24 (0.04, 0.44) |
| Libya | 9890 (8562, 11332) | 29205 (25487, 33143) | 195.3 (180.2, 210.75) | 552.58 (475.13, 635.68) | 594.31 (515.85, 677.47) | 7.55 (2.03, 13.39) | 0.28 (0.23, 0.34) | 0.18 (0.08, 0.28) |
| Lithuania | 23143 (20873, 25588) | 25844 (23276, 28492) | 11.67 (5.68, 17.49) | 519.01 (469.23, 571.61) | 430.98 (386.63, 478.13) | -16.96 (-21.07, -12.85) | -0.68 (-0.86, -0.51) | -0.52 (-0.76, -0.29) |
| Luxembourg | 1001 (863, 1147) | 1429 (1229, 1647) | 42.78 (35.53, 50.42) | 185.48 (160.86, 211.97) | 145.59 (125.61, 167.33) | -21.5 (-25.09, -17.42) | -0.95 (-1.11, -0.79) | -0.86 (-1.29, -0.43) |
| Madagascar | 10102 (8813, 11517) | 22586 (19756, 25676) | 123.59 (111.71, 136.12) | 207.34 (179.94, 236.07) | 218.42 (190.31, 249.2) | 5.34 (0.71, 9.94) | 0.15 (0.08, 0.22) | 0.22 (0.13, 0.31) |
| Malawi | 8188 (7099, 9372) | 15838 (13804, 17919) | 93.42 (85.09, 101.84) | 221.66 (192.98, 253.9) | 219.35 (190.74, 249.7) | -1.04 (-5.35, 3.47) | -0.04 (-0.07, -0.01) | -0.07 (-0.18, 0.04) |
| Malaysia | 17447 (15374, 19494) | 48487 (42960, 54263) | 177.91 (164.84, 191.1) | 189.99 (166.85, 213.46) | 186.15 (165.99, 207) | -2.02 (-7.03, 2.78) | -0.09 (-0.47, 0.3) | -0.09 (-0.16, -0.02) |
| Maldives | 149 (127, 172) | 497 (436, 561) | 233.8 (212.08, 255.15) | 168.08 (145.28, 192.66) | 148.52 (129.36, 169.37) | -11.63 (-15.97, -7.22) | -0.43 (-0.48, -0.38) | -0.41 (-1.3, 0.49) |
| Mali | 8052 (6951, 9222) | 17859 (15591, 20330) | 121.8 (111.39, 131.59) | 211.38 (182.97, 241.79) | 218.2 (190.11, 248.97) | 3.23 (-1.33, 7.59) | 0.14 (0.04, 0.23) | 0.16 (0.05, 0.27) |
| Malta | 854 (736, 980) | 1334 (1134, 1535) | 56.13 (48.14, 65.7) | 203.22 (176.02, 232.38) | 152.03 (131.79, 173.14) | -25.19 (-28.62, -21.48) | -1.03 (-1.68, -0.38) | -0.68 (-1.15, -0.21) |
| Marshall Islands | 30 (26, 34) | 63 (54, 72) | 109.5 (98.55, 120.2) | 190.65 (165.01, 217.29) | 197.36 (169.8, 226.69) | 3.52 (-0.96, 7.52) | 0.12 (0.11, 0.12) | 0.06 (-1.82, 1.97) |
| Mauritania | 2222 (1922, 2538) | 4817 (4197, 5497) | 116.81 (107.93, 126.46) | 230.6 (199.43, 265.23) | 237.45 (205.01, 273.13) | 2.97 (-1.27, 7.34) | 0.1 (0.08, 0.11) | 0.06 (-0.15, 0.27) |
| Mauritius | 1236 (1079, 1408) | 2514 (2155, 2908) | 103.49 (93.17, 115.36) | 168.83 (147.94, 192.89) | 146.74 (127.1, 167.79) | -13.08 (-16.97, -8.61) | -0.5 (-0.66, -0.34) | -0.62 (-0.91, -0.34) |
| Mexico | 86934 (75954, 98713) | 221747 (194028, 252210) | 155.08 (149.98, 160.29) | 205.82 (179.05, 234.4) | 193.39 (169.07, 220.1) | -6.04 (-7.25, -4.75) | -0.21 (-0.25, -0.18) | -0.35 (-0.4, -0.29) |
| Micronesia (Federated States of) | 87 (75, 100) | 123 (106, 142) | 42.06 (33.54, 51.14) | 198.51 (172.8, 227.59) | 187.54 (161.42, 215.21) | -5.53 (-9.55, -1.59) | -0.21 (-0.24, -0.18) | -0.22 (-1.41, 0.99) |
| Monaco | 156 (133, 182) | 160 (137, 186) | 2.26 (-2.55, 7.15) | 231.12 (199.55, 264.35) | 178.65 (155.43, 205.42) | -22.7 (-26.04, -19.02) | -0.9 (-0.92, -0.88) | -0.94 (-2.44, 0.59) |
| Mongolia | 6287 (5469, 7120) | 12651 (10952, 14467) | 101.22 (90.1, 113.4) | 658.83 (577.57, 746.58) | 626.73 (547.22, 712.93) | -4.87 (-9.34, -0.74) | -0.17 (-0.23, -0.11) | -0.2 (-0.35, -0.06) |
| Montenegro | 1861 (1600, 2135) | 2838 (2429, 3283) | 52.52 (44.22, 60.42) | 304.17 (262.9, 348.79) | 294.81 (254.87, 338.09) | -3.08 (-7.35, 0.96) | -0.12 (-0.15, -0.1) | -0.22 (-0.56, 0.12) |
| Morocco | 86529 (77550, 96572) | 190736 (173097, 211943) | 120.43 (109.83, 132.3) | 674.27 (609.3, 748.83) | 644.78 (589.1, 707.25) | -4.37 (-9, 0.53) | -0.15 (-0.21, -0.1) | -0.28 (-0.35, -0.21) |
| Mozambique | 12761 (11091, 14573) | 24328 (21173, 27587) | 90.64 (82.48, 99.14) | 223.88 (193.83, 256.21) | 226.29 (196.66, 258.96) | 1.08 (-3.34, 5.34) | 0.03 (-0.04, 0.1) | 0.11 (0.02, 0.2) |
| Myanmar | 39725 (35111, 44782) | 68580 (60592, 76777) | 72.64 (64.7, 80.14) | 178.68 (157.82, 199.91) | 153.83 (137.37, 171.26) | -13.91 (-17.48, -10.34) | -0.53 (-0.67, -0.39) | -0.61 (-0.66, -0.56) |
| Namibia | 1645 (1425, 1890) | 3077 (2664, 3512) | 87.1 (77.33, 96.93) | 235.14 (203.18, 270.8) | 222.03 (191.67, 253.7) | -5.57 (-9.89, -0.95) | -0.21 (-0.24, -0.19) | -0.22 (-0.46, 0.03) |
| Nauru | 7 (6, 8) | 8 (7, 9) | 8.3 (3.25, 13.78) | 201.31 (173.1, 230.44) | 199.15 (172.76, 227.82) | -1.07 (-4.92, 3.53) | -0.04 (-0.06, -0.02) | -0.03 (-4.6, 4.75) |
| Nepal | 33442 (28775, 38752) | 78824 (68514, 89690) | 135.7 (120.26, 150.5) | 380.34 (329.27, 438.73) | 365.37 (319.23, 413.83) | -3.93 (-9.99, 1.96) | -0.15 (-0.19, -0.11) | -0.19 (-0.26, -0.13) |
| Netherlands | 77542 (69475, 86266) | 85281 (73902, 97226) | 9.98 (2.61, 18.44) | 394.87 (352.23, 439.57) | 267.17 (232.61, 302.42) | -32.34 (-36.41, -27.79) | -1.35 (-1.4, -1.3) | -1.49 (-1.54, -1.43) |
| New Zealand | 17409 (14962, 19889) | 20219 (18499, 22100) | 16.14 (6.52, 28.38) | 447.49 (385.27, 508) | 265.58 (243.38, 288.7) | -40.65 (-45.36, -34.6) | -1.68 (-1.79, -1.57) | -1.43 (-1.55, -1.32) |
| Nicaragua | 2848 (2485, 3245) | 8443 (7333, 9621) | 196.48 (183.05, 209.1) | 185.62 (160.84, 213.22) | 197.92 (171.14, 226.26) | 6.62 (2.01, 11.19) | 0.2 (0.12, 0.28) | 0.11 (-0.05, 0.26) |
| Niger | 5507 (4753, 6291) | 15977 (13908, 18176) | 190.15 (177.62, 203.53) | 211.55 (181.88, 242.84) | 217.45 (189.14, 247.65) | 2.79 (-2.25, 7.8) | 0.12 (0.08, 0.15) | 0.12 (-0.01, 0.25) |
| Nigeria | 83944 (73017, 95672) | 177043 (155106, 200783) | 110.91 (106.93, 115.63) | 200.33 (173.41, 229.48) | 210.9 (182.98, 241.69) | 5.27 (4.24, 6.36) | 0.17 (0.11, 0.23) | 0.17 (0.13, 0.2) |
| Niue | 4 (4, 5) | 4 (4, 5) | -2.73 (-7.46, 2.9) | 188.91 (164.12, 215.19) | 192.34 (166.26, 219.17) | 1.82 (-2.86, 6.94) | 0.05 (0.04, 0.06) | 0.04 (-6.59, 7.14) |
| North Macedonia | 5195 (4470, 5958) | 8524 (7364, 9773) | 64.08 (55.45, 71.99) | 292.58 (254.31, 334.75) | 280.69 (245.38, 318.99) | -4.06 (-8.73, 0.06) | -0.12 (-0.37, 0.13) | -0.28 (-0.48, -0.08) |
| Northern Mariana Islands | 34 (29, 39) | 92 (78, 107) | 174.28 (150.86, 199.3) | 183.44 (159.3, 210.67) | 188.79 (163.15, 215.77) | 2.91 (-1.08, 7.38) | 0.09 (0.06, 0.13) | 0.09 (-1.8, 2.02) |
| Norway | 17653 (15188, 20239) | 17012 (14640, 19576) | -3.63 (-6.66, -0.64) | 264.38 (229.96, 301.36) | 185.03 (159.9, 211.64) | -30.02 (-31.4, -28.64) | -1.25 (-1.51, -1) | -1.23 (-1.35, -1.12) |
| Oman | 4035 (3489, 4592) | 12346 (10824, 14055) | 206.01 (190.02, 222.73) | 656.17 (569.13, 748.19) | 731.24 (639.41, 831.31) | 11.44 (6.13, 17.42) | 0.29 (0.22, 0.37) | 0.36 (0.2, 0.53) |
| Pakistan | 240110 (208738, 272756) | 536036 (466396, 606178) | 123.25 (113.95, 133.54) | 436.95 (379.17, 495.64) | 509.06 (444.85, 572.86) | 16.5 (12.46, 20.58) | 0.39 (0.18, 0.6) | 0.5 (0.44, 0.55) |
| Palau | 18 (16, 21) | 39 (34, 46) | 116.04 (102.46, 129.85) | 194.01 (167.46, 221.94) | 197.32 (171.74, 226.89) | 1.71 (-2.82, 6) | 0.06 (0.05, 0.06) | 0.05 (-2.38, 2.55) |
| Palestine | 5072 (4405, 5788) | 13978 (12080, 16022) | 175.59 (160.83, 191.27) | 614.47 (530.79, 702.6) | 623.64 (542.22, 706.33) | 1.49 (-2.99, 6.11) | 0.03 (-0.06, 0.12) | 0.04 (-0.1, 0.17) |
| Panama | 2624 (2266, 2979) | 7254 (6275, 8337) | 176.5 (163.94, 189.51) | 175.61 (151.45, 200.74) | 173.8 (149.92, 200.18) | -1.03 (-5.12, 3.42) | -0.05 (-0.07, -0.04) | -0.02 (-0.2, 0.16) |
| Papua New Guinea | 3483 (3036, 3982) | 9507 (8303, 10880) | 172.97 (160.75, 185.7) | 200.85 (174.63, 229.63) | 209.79 (184.41, 237.6) | 4.45 (-0.19, 8.76) | 0.15 (0.07, 0.24) | 0.16 (0, 0.32) |
| Paraguay | 2252 (1952, 2567) | 5573 (4823, 6389) | 147.49 (137.25, 160.07) | 99.44 (85.83, 113.64) | 99.22 (85.77, 113.95) | -0.22 (-4.63, 4.49) | -0.06 (-0.18, 0.06) | 0.02 (-0.17, 0.2) |
| Peru | 9841 (8633, 11188) | 24128 (21086, 27310) | 145.18 (134.13, 157.94) | 86.51 (75.8, 98.29) | 74.32 (64.64, 84.16) | -14.1 (-18.17, -9.79) | -0.53 (-0.86, -0.2) | -0.41 (-0.51, -0.32) |
| Philippines | 74030 (64813, 83987) | 180434 (157631, 205627) | 143.73 (140, 147.34) | 243.73 (212.7, 276.3) | 230.56 (202.9, 261.61) | -5.4 (-6.48, -4.3) | -0.19 (-0.27, -0.11) | -0.24 (-0.28, -0.21) |
| Poland | 130894 (111103, 150527) | 85754 (78676, 93679) | -34.49 (-40.41, -27.75) | 312.57 (266.96, 357.38) | 124.77 (114.94, 135.8) | -60.08 (-63.7, -55.91) | -3.4 (-4.28, -2.51) | -2.67 (-2.8, -2.54) |
| Portugal | 20101 (17665, 22726) | 19264 (16943, 21854) | -4.16 (-10.1, 2.06) | 148.86 (131.65, 166.92) | 82.67 (72.88, 93.72) | -44.47 (-47.59, -41.37) | -2.23 (-3.28, -1.17) | -1.78 (-1.91, -1.65) |
| Puerto Rico | 13839 (12112, 15643) | 25146 (21701, 29055) | 81.71 (72.49, 92.17) | 385.65 (338.22, 435.1) | 375.31 (323.52, 428.13) | -2.68 (-7.08, 2.07) | -0.12 (-0.18, -0.07) | -0.1 (-0.2, 0) |
| Qatar | 723 (623, 835) | 6440 (5456, 7538) | 790.89 (737.41, 851.18) | 592.89 (511.01, 684.54) | 596.6 (512.23, 688.58) | 0.63 (-4.17, 6.13) | 0 (-0.03, 0.04) | -0.13 (-0.54, 0.27) |
| Republic of Korea | 44909 (40070, 50378) | 91192 (79409, 103974) | 103.06 (90.38, 117.54) | 176.7 (158.68, 197.05) | 105.68 (92.77, 119.9) | -40.19 (-43.75, -36.07) | -1.77 (-2.24, -1.3) | -1.66 (-1.78, -1.53) |
| Republic of Moldova | 21894 (19668, 24367) | 28056 (25232, 31196) | 28.15 (21.69, 34.97) | 573.97 (521.37, 632.23) | 488.36 (440.55, 541.87) | -14.92 (-19.4, -10.57) | -0.57 (-0.72, -0.41) | -0.26 (-0.43, -0.09) |
| Romania | 101625 (92748, 111708) | 109537 (99826, 119931) | 7.79 (2.26, 13.5) | 403.72 (373.77, 437.26) | 292.96 (266.41, 320.6) | -27.43 (-31.14, -23.78) | -1.25 (-1.51, -0.99) | -0.99 (-1.15, -0.84) |
| Russian Federation | 857622 (746756, 976033) | 1099256 (961900, 1243093) | 28.17 (24.74, 31.86) | 516.04 (453.84, 581.36) | 470.09 (414.32, 529.78) | -8.91 (-10.16, -7.68) | -0.39 (-0.48, -0.3) | -0.38 (-0.44, -0.32) |
| Rwanda | 5065 (4379, 5797) | 10311 (8929, 11757) | 103.55 (94.91, 112.77) | 183.94 (158.78, 211.72) | 176.67 (153.5, 202.22) | -3.95 (-7.94, 0.31) | -0.15 (-0.19, -0.12) | -0.23 (-0.37, -0.08) |
| Saint Kitts and Nevis | 142 (122, 165) | 259 (221, 301) | 82.65 (68.43, 99.53) | 386.35 (334.94, 444.37) | 394.09 (340.27, 452.21) | 2 (-2.21, 6.31) | 0.04 (-0.07, 0.15) | 0.07 (-0.87, 1.01) |
| Saint Lucia | 358 (311, 410) | 868 (753, 994) | 142.65 (130.28, 155.16) | 404.52 (351.71, 463.24) | 406.52 (353.32, 465.76) | 0.49 (-4.15, 5.11) | -0.02 (-0.07, 0.02) | -0.01 (-0.54, 0.52) |
| Saint Vincent and the Grenadines | 283 (245, 326) | 534 (460, 614) | 89.01 (79.69, 98.66) | 391.56 (341.1, 451.47) | 399.27 (344.63, 455.91) | 1.97 (-2.29, 6.5) | 0.03 (-0.04, 0.1) | 0.06 (-0.56, 0.69) |
| Samoa | 172 (149, 197) | 288 (249, 328) | 67.07 (59.39, 75.01) | 205.43 (178.91, 235.44) | 202.97 (176.38, 231.31) | -1.2 (-5.08, 2.81) | -0.05 (-0.07, -0.03) | -0.08 (-0.84, 0.69) |
| San Marino | 76 (64, 87) | 110 (94, 127) | 45.13 (37.89, 52.74) | 233.79 (201.14, 268.73) | 178.54 (154.44, 205.11) | -23.63 (-26.92, -20.23) | -0.94 (-0.97, -0.91) | -1.02 (-2.57, 0.56) |
| Sao Tome and Principe | 133 (114, 153) | 234 (203, 266) | 76.83 (66.67, 86.7) | 214.61 (183.53, 247.11) | 229.61 (197.48, 263.76) | 6.99 (2.55, 11.32) | 0.23 (0.21, 0.25) | 0.27 (-0.62, 1.17) |
| Saudi Arabia | 31769 (27971, 35748) | 108673 (96041, 122753) | 242.07 (220.95, 266.21) | 568.57 (499.12, 643.5) | 612.58 (545.39, 686.37) | 7.74 (1.99, 13.77) | 0.22 (0.17, 0.27) | 0.38 (0.33, 0.44) |
| Senegal | 7380 (6388, 8421) | 17168 (15033, 19421) | 132.62 (123.73, 142.19) | 239.67 (207.81, 273.67) | 237.04 (206.93, 270.06) | -1.1 (-5.1, 3.22) | -0.04 (-0.05, -0.03) | -0.02 (-0.13, 0.09) |
| Serbia | 45884 (40684, 51249) | 60265 (54026, 66827) | 31.34 (24.67, 38.96) | 425.29 (383.29, 471.26) | 405.76 (364.64, 447.91) | -4.59 (-9.05, -0.07) | -0.25 (-0.41, -0.08) | -0.27 (-0.4, -0.14) |
| Seychelles | 86 (75, 99) | 163 (140, 187) | 88.57 (78.38, 99.6) | 152.73 (132.65, 175.41) | 148.09 (128.05, 169.49) | -3.04 (-7.24, 1.24) | -0.09 (-0.13, -0.05) | -0.14 (-1.21, 0.93) |
| Sierra Leone | 4684 (4059, 5368) | 8864 (7731, 10068) | 89.22 (80.57, 98.64) | 252.37 (218.93, 288.83) | 252.97 (219.03, 289.52) | 0.24 (-3.7, 5.11) | 0 (-0.01, 0.01) | 0.07 (-0.08, 0.21) |
| Singapore | 4655 (4063, 5251) | 12873 (12200, 13510) | 176.57 (148.81, 209.67) | 215.32 (186.67, 244.33) | 168 (159.09, 176.46) | -21.98 (-30.23, -11.93) | -0.89 (-0.98, -0.8) | -0.58 (-0.72, -0.45) |
| Slovakia | 24938 (22563, 27578) | 26586 (23723, 29643) | 6.61 (0.25, 13.03) | 430.07 (391.76, 472.89) | 296.53 (266.79, 329.23) | -31.05 (-34.84, -26.98) | -1.36 (-1.92, -0.79) | -1.3 (-1.48, -1.12) |
| Slovenia | 7136 (6161, 8218) | 10913 (9356, 12530) | 52.94 (44.92, 61.17) | 295.29 (256.76, 339.1) | 257.58 (222.47, 295.12) | -12.77 (-16.89, -8.44) | -0.75 (-1.03, -0.48) | -1.09 (-1.29, -0.89) |
| Solomon Islands | 268 (231, 307) | 614 (531, 705) | 128.9 (117.48, 139.56) | 215.11 (186.81, 245.84) | 212.99 (184.99, 243.39) | -0.99 (-5.24, 3.42) | -0.08 (-0.13, -0.02) | -0.07 (-0.83, 0.68) |
| Somalia | 5044 (4379, 5727) | 14060 (12238, 15955) | 178.75 (166.9, 192.34) | 212.09 (182.6, 243) | 219.7 (191.43, 249.44) | 3.59 (-0.52, 8.26) | 0.12 (0.1, 0.13) | 0.14 (0.01, 0.28) |
| South Africa | 54377 (47361, 61698) | 106721 (93024, 121899) | 96.26 (92.54, 100.2) | 263.42 (228.58, 300.44) | 244.17 (213.23, 279) | -7.31 (-8.99, -5.53) | -0.23 (-0.28, -0.18) | -0.32 (-0.36, -0.28) |
| South Sudan | 4580 (3956, 5233) | 7912 (6863, 9049) | 72.76 (64.29, 81.56) | 196.29 (168.46, 226.47) | 213.15 (184.21, 246.18) | 8.59 (3.57, 13.82) | 0.27 (0.25, 0.3) | 0.24 (0.1, 0.39) |
| Spain | 141960 (127415, 158132) | 166334 (148058, 185406) | 17.17 (10.35, 24.17) | 268.68 (241.32, 297.68) | 182.14 (160.74, 204.12) | -32.21 (-36.21, -28.16) | -1.33 (-1.36, -1.3) | -1.19 (-1.28, -1.1) |
| Sri Lanka | 19547 (17306, 21909) | 38707 (34008, 43365) | 98.02 (88.38, 106.77) | 188.72 (168.2, 210.84) | 159.76 (141.66, 178.07) | -15.34 (-18.94, -11.57) | -0.65 (-0.74, -0.55) | -0.52 (-0.6, -0.45) |
| Sudan | 59866 (53662, 66645) | 113150 (101102, 126115) | 89 (80.12, 99.39) | 686.65 (617.95, 761.94) | 636.36 (572.22, 705.45) | -7.32 (-11.77, -2.35) | -0.28 (-0.38, -0.18) | -0.26 (-0.31, -0.22) |
| Suriname | 1037 (900, 1183) | 2557 (2206, 2921) | 146.59 (135.4, 158) | 393.61 (339.6, 449.17) | 427.93 (370.75, 489.77) | 8.72 (3.99, 13.55) | 0.29 (0.24, 0.35) | 0.32 (0.04, 0.61) |
| Sweden | 42007 (39320, 45062) | 38484 (34706, 42926) | -8.39 (-15.09, -0.72) | 273.83 (257.2, 291.87) | 187.8 (170.71, 206.83) | -31.42 (-35.92, -26.24) | -1.38 (-1.5, -1.26) | -0.86 (-0.94, -0.78) |
| Switzerland | 26885 (23713, 30112) | 29752 (26040, 33813) | 10.66 (4.02, 17.87) | 257.25 (227.95, 287.99) | 174 (152.36, 197.72) | -32.36 (-35.94, -28.26) | -1.26 (-1.46, -1.05) | -0.92 (-1.02, -0.83) |
| Syrian Arab Republic | 33270 (29442, 37257) | 80332 (71888, 89818) | 141.45 (128.25, 153.93) | 681.87 (610.39, 758.02) | 712.84 (644.73, 785.67) | 4.54 (-0.84, 10.12) | 0.14 (-0.06, 0.34) | -0.04 (-0.1, 0.02) |
| Taiwan (Province of China) | 24804 (21656, 28224) | 62511 (54696, 70983) | 152.02 (138.97, 166.9) | 183.4 (161.15, 207.77) | 158.47 (139.21, 179.48) | -13.59 (-17.29, -9.7) | -0.34 (-0.82, 0.14) | -0.24 (-0.31, -0.17) |
| Tajikistan | 15785 (13856, 17909) | 28953 (25642, 32599) | 83.42 (70.66, 95.28) | 570.72 (500.02, 644.64) | 722.55 (656.41, 793.82) | 26.6 (18.67, 34.83) | 0.89 (0.69, 1.1) | 0.38 (0.29, 0.47) |
| Thailand | 49846 (43591, 56491) | 124433 (108901, 139994) | 149.63 (138.04, 161.75) | 144.58 (127.44, 162.86) | 123.27 (108.65, 138.08) | -14.74 (-18.18, -11.04) | -0.56 (-0.6, -0.51) | -0.49 (-0.55, -0.43) |
| Timor-Leste | 450 (388, 513) | 1194 (1035, 1363) | 165.47 (146.79, 183.97) | 151.11 (130.69, 173.98) | 149.63 (130.29, 171.17) | -0.98 (-5.45, 3.39) | -0.04 (-0.11, 0.03) | -0.04 (-0.46, 0.4) |
| Togo | 2701 (2346, 3084) | 8139 (7066, 9293) | 201.3 (187.84, 215.83) | 227.02 (196.71, 261.14) | 233.94 (203.09, 267.71) | 3.05 (-1.68, 7.57) | 0.09 (0.05, 0.14) | 0.16 (-0.01, 0.33) |
| Tokelau | 3 (2, 3) | 3 (2, 3) | 0.38 (-4.98, 5.75) | 192.55 (167.37, 221.42) | 198.13 (171, 227.14) | 2.9 (-1.18, 7.28) | 0.1 (0.07, 0.14) | 0.07 (-7.82, 8.64) |
| Tonga | 108 (94, 123) | 160 (138, 182) | 47.82 (40.79, 55.32) | 203.2 (175.84, 232.43) | 202.29 (174.71, 231.48) | -0.44 (-4.67, 4.05) | -0.01 (-0.04, 0.02) | -0.04 (-1.04, 0.97) |
| Trinidad and Tobago | 3909 (3417, 4468) | 8185 (7089, 9432) | 109.39 (98.56, 119.72) | 462.33 (402.66, 528.18) | 444.89 (388.65, 508.62) | -3.77 (-8.34, 0.65) | -0.15 (-0.29, 0) | -0.17 (-0.34, 0) |
| Tunisia | 26494 (23065, 30250) | 68220 (60187, 77244) | 157.49 (145.05, 170.54) | 561.05 (493.94, 632.63) | 558.1 (496.56, 627.1) | -0.53 (-5.57, 4.99) | -0.04 (-0.1, 0.02) | -0.05 (-0.13, 0.02) |
| Turkey | 163797 (148272, 180469) | 281417 (252886, 312699) | 71.81 (62.59, 82.28) | 474.24 (432.28, 517.87) | 325.48 (293.75, 359.9) | -31.37 (-34.89, -27.42) | -1.38 (-1.53, -1.23) | -1.5 (-1.67, -1.33) |
| Turkmenistan | 10925 (9632, 12304) | 22119 (19555, 24866) | 102.45 (92.17, 112.43) | 636.33 (561.59, 711.22) | 625.3 (558.26, 698.5) | -1.73 (-6.45, 3.17) | -0.16 (-0.32, 0.01) | -0.17 (-0.27, -0.06) |
| Tuvalu | 12 (10, 14) | 19 (17, 22) | 59.78 (52.08, 68.64) | 193.03 (167.23, 221.29) | 199.58 (172.62, 227.47) | 3.39 (-0.91, 7.75) | 0.11 (0.1, 0.12) | 0.13 (-3.04, 3.4) |
| Uganda | 11760 (10198, 13443) | 25558 (22310, 29061) | 117.33 (108.03, 126.86) | 186.97 (160.88, 214.25) | 183.14 (159.03, 208.59) | -2.05 (-6.17, 2.65) | -0.08 (-0.1, -0.06) | -0.09 (-0.18, -0.01) |
| Ukraine | 384314 (338201, 436829) | 499577 (437913, 568362) | 29.99 (23.8, 36.58) | 577.02 (512.07, 652.75) | 654.28 (575.33, 741.49) | 13.39 (8.7, 18.53) | 0.37 (0.16, 0.59) | 0.17 (0.07, 0.28) |
| United Arab Emirates | 2723 (2359, 3159) | 27614 (23612, 32368) | 914.21 (845.13, 986.54) | 605.71 (522.32, 703.14) | 654 (565.64, 754.13) | 7.97 (3.3, 13.24) | 0.28 (0.2, 0.37) | 0.2 (-0.1, 0.51) |
| United Kingdom | 248194 (212335, 285890) | 218032 (191100, 247063) | -12.15 (-16.01, -7.69) | 277.88 (239.21, 317.98) | 182.99 (161.65, 205.7) | -34.15 (-36.87, -31.05) | -1.43 (-1.53, -1.34) | -1.24 (-1.35, -1.14) |
| United Republic of Tanzania | 20359 (17656, 23247) | 51710 (45448, 58207) | 153.99 (141.54, 167.14) | 195.08 (168.92, 222.59) | 214.25 (188.16, 241) | 9.83 (4.78, 15.01) | 0.33 (0.31, 0.34) | 0.39 (0.32, 0.46) |
| United States Virgin Islands | 310 (269, 356) | 659 (564, 770) | 112.8 (98.52, 127.14) | 365.37 (316.97, 418.89) | 368.78 (321.46, 424.35) | 0.93 (-3.83, 5.83) | 0.03 (-0.08, 0.14) | 0.05 (-0.61, 0.72) |
| United States of America | 1228300 (1058035, 1403012) | 959514 (873685, 1057614) | -21.88 (-27.46, -15.74) | 384.98 (334.4, 441.06) | 175.48 (160.46, 192.6) | -54.42 (-57.62, -50.74) | -2.75 (-2.91, -2.59) | -2.63 (-2.83, -2.44) |
| Uruguay | 8624 (7494, 9800) | 9857 (8471, 11352) | 14.3 (7.49, 21.48) | 223.61 (195.29, 253.3) | 176.32 (151.91, 202.19) | -21.15 (-25.28, -16.64) | -0.92 (-1.05, -0.78) | -0.78 (-0.96, -0.61) |
| Uzbekistan | 59808 (53981, 66201) | 143735 (130590, 157240) | 140.33 (124.25, 157.82) | 561.01 (507.99, 617.96) | 1011.62 (952.08, 1078.8) | 80.32 (68.32, 93.2) | 2.04 (1.7, 2.38) | 1.18 (1.02, 1.34) |
| Vanuatu | 144 (125, 165) | 369 (320, 421) | 155.85 (144.15, 168.13) | 231.47 (201.07, 264.91) | 225.45 (195.06, 256.61) | -2.6 (-6.8, 2.16) | -0.09 (-0.16, -0.03) | -0.1 (-0.94, 0.74) |
| Venezuela (Bolivarian Republic of) | 22354 (19857, 24912) | 58773 (51747, 65879) | 162.92 (152.29, 175.44) | 235.17 (208, 262.52) | 204.33 (180.77, 229.51) | -13.11 (-16.6, -9.17) | -0.52 (-0.73, -0.32) | -0.55 (-0.63, -0.48) |
| Viet Nam | 59848 (53373, 67222) | 134765 (120727, 149514) | 125.18 (114.39, 136.39) | 153.46 (137.14, 171.64) | 150.77 (136.96, 166.01) | -1.75 (-6.25, 2.86) | -0.08 (-0.2, 0.04) | -0.08 (-0.13, -0.04) |
| Yemen | 29686 (26137, 33323) | 79100 (70449, 88561) | 166.45 (153.82, 181.43) | 656.28 (578.94, 736.86) | 636.71 (572.39, 709.07) | -2.98 (-7.78, 2.41) | -0.11 (-0.19, -0.02) | -0.16 (-0.22, -0.1) |
| Zambia | 5676 (4920, 6471) | 13377 (11696, 15228) | 135.69 (124.82, 145.97) | 206.79 (178.06, 238.27) | 201.72 (174.66, 230.86) | -2.45 (-7.02, 1.99) | -0.1 (-0.14, -0.07) | -0.13 (-0.26, 0) |
| Zimbabwe | 9221 (8018, 10481) | 17081 (14963, 19466) | 85.23 (77.12, 93.17) | 234.58 (203.43, 268.47) | 255.61 (223.69, 290.97) | 8.96 (4.02, 13.85) | 0.26 (0.19, 0.33) | 0.33 (0.23, 0.43) |
