# Supplementary material for: Global, regional, and national time trends in ischaemic heart disease incidence over three decades (1990–2019): an age-period-cohort analysis of the global burden of disease study 2019
Source: Front Cardiovasc Med. 2024 Nov 1;11:1396380. doi: 10.3389/fcvm.2024.1396380 (PMC11563781; doi:10.3389/fcvm.2024.1396380)
Supplement: Supplementary file 3 [file Table2.docx]

Table S2. Tests for ischemic heart disease incidence for both sexes across socio-demographic index quintiles and 21 regions, 1990-2019

| location | sex | | | All Age Deviations = 0 | | All Cohort Deviations = 0 | | All Cohort RR = 1 | | All Gradient Shifts = CAT | | All Higher-Order Age Deviations = 0 | | All Higher-Order Cohort Deviations = 0 | | | All Higher-Order Period Deviations = 0 | | All Local Drifts = Net Drift | All Period Deviations = 0 | | All Period RR = 1 | | NetDrift = 0 | THETAa = 0 | THETAc = 0 | THETAp = 0 |
| --- | --- | --- | --- | --- | --- | --- | --- | --- | --- | --- | --- | --- | --- | --- | --- | --- | --- | --- | --- | --- | --- | --- | --- | --- | --- | --- | --- |
| Global | Both | | | < 0.001 | | < 0.001 | | < 0.001 | | < 0.001 | | < 0.001 | | < 0.001 | | | 0 | | < 0.001 | < 0.001 | | < 0.001 | | < 0.001 | < 0.001 | < 0.001 | < 0.001 |
| Global | Female | | | < 0.001 | | < 0.001 | | < 0.001 | | < 0.001 | | < 0.001 | | < 0.001 | | | < 0.001 | | < 0.001 | < 0.001 | | < 0.001 | | < 0.001 | < 0.001 | < 0.001 | < 0.001 |
| Global | Male | | | < 0.001 | | < 0.001 | | < 0.001 | | < 0.001 | | < 0.001 | | < 0.001 | | | 0.02 | | < 0.001 | < 0.001 | | < 0.001 | | < 0.001 | < 0.001 | < 0.001 | < 0.001 |
| High SDI | Both | | | < 0.001 | | < 0.001 | | < 0.001 | | < 0.001 | | < 0.001 | | < 0.001 | | | < 0.001 | | < 0.001 | < 0.001 | | < 0.001 | | < 0.001 | < 0.001 | < 0.001 | < 0.001 |
| High SDI | Female | | | < 0.001 | | < 0.001 | | < 0.001 | | < 0.001 | | < 0.001 | | < 0.001 | | | < 0.001 | | < 0.001 | < 0.001 | | < 0.001 | | < 0.001 | < 0.001 | < 0.001 | < 0.001 |
| High SDI | Male | | | < 0.001 | | < 0.001 | | < 0.001 | | < 0.001 | | < 0.001 | | < 0.001 | | | < 0.001 | | < 0.001 | < 0.001 | | < 0.001 | | < 0.001 | < 0.001 | < 0.001 | < 0.001 |
| High-middle SDI | Both | | | < 0.001 | | < 0.001 | | < 0.001 | | < 0.001 | | < 0.001 | | < 0.001 | | | < 0.001 | | < 0.001 | < 0.001 | | < 0.001 | | < 0.001 | < 0.001 | < 0.001 | < 0.001 |
| High-middle SDI | Female | | | < 0.001 | | < 0.001 | | < 0.001 | | < 0.001 | | < 0.001 | | < 0.001 | | | < 0.001 | | < 0.001 | < 0.001 | | < 0.001 | | < 0.001 | < 0.001 | < 0.001 | < 0.001 |
| High-middle SDI | Male | | | < 0.001 | | < 0.001 | | < 0.001 | | < 0.001 | | < 0.001 | | < 0.001 | | | < 0.001 | | < 0.001 | < 0.001 | | < 0.001 | | < 0.001 | < 0.001 | < 0.001 | < 0.001 |
| Middle SDI | Both | | | < 0.001 | | < 0.001 | | < 0.001 | | < 0.001 | | < 0.001 | | < 0.001 | | | < 0.001 | | < 0.001 | < 0.001 | | < 0.001 | | < 0.001 | < 0.001 | 0.076 | < 0.001 |
| Middle SDI | Female | | | < 0.001 | | < 0.001 | | < 0.001 | | < 0.001 | | < 0.001 | | < 0.001 | | | 0.01 | | < 0.001 | < 0.001 | | < 0.001 | | < 0.001 | < 0.001 | 0.259 | < 0.001 |
| Middle SDI | Male | | | < 0.001 | | < 0.001 | | < 0.001 | | < 0.001 | | < 0.001 | | < 0.001 | | | < 0.001 | | < 0.001 | < 0.001 | | < 0.001 | | < 0.001 | < 0.001 | 0.005 | < 0.001 |
| Low-middle SDI | Both | | | < 0.001 | | 0.08 | | 0.013 | | 0.07 | | < 0.001 | | 0.836 | | | < 0.001 | | 0.048 | < 0.001 | | < 0.001 | | 0 | < 0.001 | < 0.001 | < 0.001 |
| Low-middle SDI | Female | | | < 0.001 | | < 0.001 | | < 0.001 | | < 0.001 | | < 0.001 | | < 0.001 | | | 0.02 | | 0.001 | 0.035 | | 0 | | 0 | < 0.001 | < 0.001 | 0.32 |
| Low-middle SDI | Male | | | < 0.001 | | 0.21 | | < 0.001 | | 0.4 | | < 0.001 | | 0.97 | | | < 0.001 | | 0.17 | < 0.001 | | < 0.001 | | < 0.001 | < 0.001 | 0.01 | < 0.001 |
| Low SDI | Both | | | < 0.001 | | < 0.001 | | < 0.001 | | < 0.001 | | < 0.001 | | < 0.001 | | | 0.01 | | < 0.001 | < 0.001 | | < 0.001 | | < 0.001 | < 0.001 | < 0.001 | < 0.001 |
| Low SDI | Female | | | < 0.001 | | < 0.001 | | < 0.001 | | < 0.001 | | < 0.001 | | < 0.001 | | | 0.22 | | < 0.001 | < 0.001 | | < 0.001 | | < 0.001 | < 0.001 | < 0.001 | < 0.001 |
| Low SDI | Male | | | < 0.001 | | < 0.001 | | < 0.001 | | 0 | | < 0.001 | | < 0.001 | | | < 0.001 | | < 0.001 | < 0.001 | | < 0.001 | | < 0.001 | < 0.001 | < 0.001 | < 0.001 |
| High-income Asia Pacific | Both | | | < 0.001 | | < 0.001 | | < 0.001 | | < 0.001 | | < 0.001 | | 0.005 | | | < 0.001 | | < 0.001 | < 0.001 | | < 0.001 | | < 0.001 | < 0.001 | < 0.001 | < 0.001 |
| High-income Asia Pacific | Female | | | < 0.001 | | < 0.001 | | < 0.001 | | < 0.001 | | < 0.001 | | < 0.001 | | | < 0.001 | | < 0.001 | < 0.001 | | < 0.001 | | < 0.001 | < 0.001 | < 0.001 | < 0.001 |
| High-income Asia Pacific | Male | | | < 0.001 | | < 0.001 | | < 0.001 | | < 0.001 | | < 0.001 | | 0.063 | | | < 0.001 | | < 0.001 | < 0.001 | | < 0.001 | | < 0.001 | < 0.001 | < 0.001 | < 0.001 |
| High-income North America | Both | | | < 0.001 | | < 0.001 | | < 0.001 | | < 0.001 | | < 0.001 | | < 0.001 | | | < 0.001 | | < 0.001 | < 0.001 | | < 0.001 | | < 0.001 | < 0.001 | < 0.001 | 0.211 |
| High-income North America | Female | | | < 0.001 | | < 0.001 | | < 0.001 | | < 0.001 | | < 0.001 | | < 0.001 | | | < 0.001 | | < 0.001 | < 0.001 | | < 0.001 | | < 0.001 | < 0.001 | < 0.001 | 0.002 |
| High-income North America | Male | | | < 0.001 | | < 0.001 | | < 0.001 | | < 0.001 | | < 0.001 | | < 0.001 | | | < 0.001 | | < 0.001 | < 0.001 | | < 0.001 | | < 0.001 | < 0.001 | < 0.001 | 0.752 |
| Western Europe | Both | | | < 0.001 | | < 0.001 | | < 0.001 | | < 0.001 | | < 0.001 | | < 0.001 | | | < 0.001 | | < 0.001 | < 0.001 | | < 0.001 | | < 0.001 | < 0.001 | < 0.001 | < 0.001 |
| Western Europe | Female | | | < 0.001 | | < 0.001 | | < 0.001 | | < 0.001 | | < 0.001 | | < 0.001 | | | < 0.001 | | < 0.001 | < 0.001 | | < 0.001 | | < 0.001 | 0.008 | < 0.001 | < 0.001 |
| Western Europe | Male | | | < 0.001 | | < 0.001 | | < 0.001 | | < 0.001 | | < 0.001 | | < 0.001 | | | < 0.001 | | < 0.001 | < 0.001 | | < 0.001 | | < 0.001 | < 0.001 | < 0.001 | < 0.001 |
| Australasia | Both | | | < 0.001 | | < 0.001 | | < 0.001 | | < 0.001 | | < 0.001 | | < 0.001 | | | 0.41 | | < 0.001 | 0.387 | | < 0.001 | | < 0.001 | < 0.001 | < 0.001 | 0.243 |
| Australasia | Female | | | < 0.001 | | < 0.001 | | < 0.001 | | < 0.001 | | < 0.001 | | < 0.001 | | | < 0.001 | | < 0.001 | < 0.001 | | < 0.001 | | < 0.001 | < 0.001 | < 0.001 | 0.048 |
| Australasia | Male | | | < 0.001 | | < 0.001 | | < 0.001 | | < 0.001 | | < 0.001 | | < 0.001 | | | 0.01 | | < 0.001 | < 0.001 | | < 0.001 | | < 0.001 | < 0.001 | < 0.001 | < 0.001 |
| Andean Latin America | Both | | | < 0.001 | | < 0.001 | | < 0.001 | | < 0.001 | | < 0.001 | | 0.03 | | | 0.04 | | < 0.001 | < 0.001 | | < 0.001 | | < 0.001 | < 0.001 | < 0.001 | < 0.001 |
| Andean Latin America | Female | | | < 0.001 | | 0.17 | | < 0.001 | | 0.07 | | < 0.001 | | 0.865 | | | 0.4 | | 0.077 | < 0.001 | | < 0.001 | | 0 | < 0.001 | 0.058 | < 0.001 |
| Andean Latin America | Male | | | < 0.001 | | < 0.001 | | < 0.001 | | 0.11 | | < 0.001 | | 0.012 | | | 0.15 | | < 0.001 | < 0.001 | | < 0.001 | | < 0.001 | < 0.001 | 0.002 | < 0.001 |
| Tropical Latin America | Both | | | < 0.001 | | < 0.001 | | < 0.001 | | < 0.001 | | < 0.001 | | < 0.001 | | | 0.05 | | < 0.001 | 0.001 | | < 0.001 | | < 0.001 | < 0.001 | < 0.001 | < 0.001 |
| Tropical Latin America | Female | | | < 0.001 | | < 0.001 | | < 0.001 | | < 0.001 | | < 0.001 | | < 0.001 | | | 0.24 | | < 0.001 | 0.141 | | < 0.001 | | < 0.001 | < 0.001 | < 0.001 | 0.053 |
| Tropical Latin America | Male | | | < 0.001 | | < 0.001 | | < 0.001 | | < 0.001 | | < 0.001 | | < 0.001 | | | 0.02 | | < 0.001 | < 0.001 | | < 0.001 | | < 0.001 | < 0.001 | < 0.001 | < 0.001 |
| Central Latin America | Both | | | < 0.001 | | < 0.001 | | < 0.001 | | < 0.001 | | < 0.001 | | < 0.001 | | | 0.19 | | < 0.001 | < 0.001 | | < 0.001 | | < 0.001 | < 0.001 | 0.255 | < 0.001 |
| Central Latin America | Female | | | < 0.001 | | < 0.001 | | < 0.001 | | < 0.001 | | < 0.001 | | < 0.001 | | | 0.19 | | < 0.001 | < 0.001 | | < 0.001 | | < 0.001 | < 0.001 | 0.006 | 0.001 |
| Central Latin America | Male | | | < 0.001 | | < 0.001 | | < 0.001 | | 0 | | < 0.001 | | < 0.001 | | | 0.02 | | < 0.001 | < 0.001 | | < 0.001 | | < 0.001 | < 0.001 | 0.489 | < 0.001 |
| Southern Latin America | Both | | | < 0.001 | | < 0.001 | | < 0.001 | | < 0.001 | | < 0.001 | | 0.992 | | | 0.15 | | < 0.001 | < 0.001 | | < 0.001 | | < 0.001 | < 0.001 | < 0.001 | < 0.001 |
| Southern Latin America | Female | | | < 0.001 | | < 0.001 | | < 0.001 | | < 0.001 | | < 0.001 | | 0.585 | | | 0.16 | | < 0.001 | < 0.001 | | < 0.001 | | < 0.001 | < 0.001 | < 0.001 | < 0.001 |
| Southern Latin America | Male | | | < 0.001 | | < 0.001 | | < 0.001 | | < 0.001 | | < 0.001 | | 1 | | | 0.05 | | < 0.001 | < 0.001 | | < 0.001 | | < 0.001 | < 0.001 | < 0.001 | < 0.001 |
| Caribbean | Both | | | < 0.001 | | < 0.001 | | < 0.001 | | < 0.001 | | < 0.001 | | < 0.001 | | | 0.69 | | < 0.001 | 0.819 | | < 0.001 | | < 0.001 | < 0.001 | < 0.001 | 0.844 |
| Caribbean | Female | | | < 0.001 | | < 0.001 | | < 0.001 | | < 0.001 | | < 0.001 | | < 0.001 | | | 0.71 | | < 0.001 | 0.304 | | < 0.001 | | < 0.001 | < 0.001 | < 0.001 | 0.083 |
| Caribbean | Male | | | < 0.001 | | < 0.001 | | < 0.001 | | < 0.001 | | < 0.001 | | < 0.001 | | | 0.77 | | < 0.001 | 0.618 | | < 0.001 | | < 0.001 | < 0.001 | < 0.001 | 0.225 |
| Central Europe | Both | | | < 0.001 | | < 0.001 | | < 0.001 | | < 0.001 | | < 0.001 | | < 0.001 | | | < 0.001 | | < 0.001 | < 0.001 | | < 0.001 | | < 0.001 | < 0.001 | < 0.001 | < 0.001 |
| Central Europe | Female | | | < 0.001 | | < 0.001 | | < 0.001 | | < 0.001 | | < 0.001 | | < 0.001 | | | < 0.001 | | < 0.001 | < 0.001 | | < 0.001 | | < 0.001 | < 0.001 | < 0.001 | < 0.001 |
| Central Europe | Male | | | < 0.001 | | < 0.001 | | < 0.001 | | < 0.001 | | < 0.001 | | < 0.001 | | | < 0.001 | | < 0.001 | < 0.001 | | < 0.001 | | < 0.001 | < 0.001 | < 0.001 | < 0.001 |
| Eastern Europe | Both | | | < 0.001 | | < 0.001 | | < 0.001 | | < 0.001 | | < 0.001 | | < 0.001 | | | < 0.001 | | < 0.001 | < 0.001 | | < 0.001 | | < 0.001 | < 0.001 | < 0.001 | < 0.001 |
| Eastern Europe | Female | | | < 0.001 | | < 0.001 | | < 0.001 | | < 0.001 | | < 0.001 | | 0.003 | | | < 0.001 | | < 0.001 | < 0.001 | | < 0.001 | | 0 | < 0.001 | < 0.001 | < 0.001 |
| Eastern Europe | Male | | | < 0.001 | | < 0.001 | | < 0.001 | | < 0.001 | | < 0.001 | | < 0.001 | | | < 0.001 | | < 0.001 | < 0.001 | | < 0.001 | | < 0.001 | < 0.001 | < 0.001 | < 0.001 |
| Central Asia | Both | | | < 0.001 | | < 0.001 | | < 0.001 | | 0.97 | | < 0.001 | | < 0.001 | | | < 0.001 | | < 0.001 | < 0.001 | | < 0.001 | | 0.71 | < 0.001 | 0.714 | < 0.001 |
| Central Asia | Female | | | < 0.001 | | < 0.001 | | < 0.001 | | 0.96 | | < 0.001 | | < 0.001 | | | 0.02 | | < 0.001 | < 0.001 | | < 0.001 | | 0.6 | < 0.001 | 0.764 | < 0.001 |
| Central Asia | Male | | | < 0.001 | | 0.02 | | 0.008 | | 0.64 | | < 0.001 | | 0.025 | | | < 0.001 | | 0.014 | < 0.001 | | < 0.001 | | 0.05 | < 0.001 | 0.439 | < 0.001 |
| North Africa and Middle East | Both | | | < 0.001 | | < 0.001 | | < 0.001 | | < 0.001 | | < 0.001 | | < 0.001 | | | 0.02 | | < 0.001 | 0.049 | | < 0.001 | | < 0.001 | < 0.001 | < 0.001 | 0.707 |
| North Africa and Middle East | Female | | | < 0.001 | | < 0.001 | | < 0.001 | | 0 | | < 0.001 | | < 0.001 | | | 0.59 | | < 0.001 | 0.679 | | < 0.001 | | < 0.001 | < 0.001 | 0.095 | 0.418 |
| North Africa and Middle East | Male | | | < 0.001 | | < 0.001 | | < 0.001 | | < 0.001 | | < 0.001 | | < 0.001 | | | < 0.001 | | < 0.001 | < 0.001 | | < 0.001 | | < 0.001 | < 0.001 | < 0.001 | 0.555 |
| South Asia | Both | | | < 0.001 | | < 0.001 | | < 0.001 | | 0.17 | | < 0.001 | | 0.88 | | | < 0.001 | | < 0.001 | < 0.001 | | < 0.001 | | 0.02 | < 0.001 | 0.008 | < 0.001 |
| South Asia | Female | | | < 0.001 | | < 0.001 | | < 0.001 | | 0.69 | | < 0.001 | | 0.111 | | | 0.01 | | < 0.001 | < 0.001 | | < 0.001 | | 0.08 | < 0.001 | 0.11 | < 0.001 |
| South Asia | Male | | | < 0.001 | | < 0.001 | | < 0.001 | | 0.27 | | < 0.001 | | 0.775 | | | < 0.001 | | < 0.001 | < 0.001 | | < 0.001 | | < 0.001 | < 0.001 | 0.019 | < 0.001 |
| Southeast Asia | Both | | | < 0.001 | | < 0.001 | | < 0.001 | | < 0.001 | | < 0.001 | | < 0.001 | | | < 0.001 | | < 0.001 | < 0.001 | | < 0.001 | | < 0.001 | < 0.001 | < 0.001 | < 0.001 |
| Southeast Asia | Female | | | < 0.001 | | < 0.001 | | < 0.001 | | < 0.001 | | < 0.001 | | < 0.001 | | | < 0.001 | | < 0.001 | < 0.001 | | < 0.001 | | < 0.001 | < 0.001 | < 0.001 | < 0.001 |
| Southeast Asia | Male | | | < 0.001 | | < 0.001 | | < 0.001 | | < 0.001 | | < 0.001 | | < 0.001 | | | < 0.001 | | < 0.001 | < 0.001 | | < 0.001 | | < 0.001 | < 0.001 | < 0.001 | < 0.001 |
| East Asia | Both | | | < 0.001 | | < 0.001 | | < 0.001 | | < 0.001 | | < 0.001 | | < 0.001 | | | < 0.001 | | < 0.001 | < 0.001 | | < 0.001 | | < 0.001 | < 0.001 | < 0.001 | 0.409 |
| East Asia | Female | | | < 0.001 | | < 0.001 | | < 0.001 | | < 0.001 | | < 0.001 | | < 0.001 | | | < 0.001 | | < 0.001 | < 0.001 | | < 0.001 | | < 0.001 | 0.334 | < 0.001 | 0.055 |
| East Asia | Male | | | < 0.001 | | < 0.001 | | < 0.001 | | < 0.001 | | < 0.001 | | < 0.001 | | | < 0.001 | | < 0.001 | < 0.001 | | < 0.001 | | < 0.001 | < 0.001 | < 0.001 | 0.925 |
| Oceania | Both | | | < 0.001 | | 1 | | 0.998 | | 1 | | < 0.001 | | 1 | | | 0.94 | | 1 | 0.715 | | 0.54 | | 0.11 | < 0.001 | 0.96 | 0.249 |
| Oceania | Female | | | < 0.001 | | 1 | | 1 | | 1 | | < 0.001 | | 1 | | | 0.99 | | 1 | 0.976 | | 0.94 | | 0.33 | < 0.001 | 0.908 | 0.599 |
| Oceania | Male | | | < 0.001 | | 1 | | 1 | | 1 | | < 0.001 | | 1 | | | 0.98 | | 1 | 0.79 | | 0.78 | | 0.3 | < 0.001 | 0.945 | 0.266 |
| Western Sub-Saharan Africa | Both | | | < 0.001 | | 0.01 | | < 0.001 | | 0.01 | | < 0.001 | | 0.056 | | | 0.32 | | 0.006 | < 0.001 | | < 0.001 | | < 0.001 | < 0.001 | 0.207 | < 0.001 |
| Western Sub-Saharan Africa | Female | | | < 0.001 | | 0.64 | | < 0.001 | | 0.99 | | < 0.001 | | 0.877 | | | 0.49 | | 0.446 | < 0.001 | | < 0.001 | | < 0.001 | < 0.001 | 0.695 | < 0.001 |
| Western Sub-Saharan Africa | Male | | | < 0.001 | | < 0.001 | | < 0.001 | | 0.05 | | < 0.001 | | 0.408 | | | 0.74 | | < 0.001 | < 0.001 | | < 0.001 | | < 0.001 | < 0.001 | 0.001 | < 0.001 |
| Eastern Sub-Saharan Africa | Both | | | < 0.001 | | < 0.001 | | < 0.001 | | 0.03 | | < 0.001 | | 0.913 | | | 0.33 | | < 0.001 | < 0.001 | | < 0.001 | | < 0.001 | < 0.001 | 0.001 | < 0.001 |
| Eastern Sub-Saharan Africa | Female | | | < 0.001 | | 1 | | 0.857 | | 1 | | < 0.001 | | 1 | | | 0.87 | | 0.999 | < 0.001 | | < 0.001 | | < 0.001 | < 0.001 | 0.947 | < 0.001 |
| Eastern Sub-Saharan Africa | Male | | | < 0.001 | | 0.02 | | < 0.001 | | 0.05 | | < 0.001 | | 0.998 | | | 0.49 | | 0.007 | < 0.001 | | < 0.001 | | < 0.001 | < 0.001 | 0.011 | < 0.001 |
| Central Sub-Saharan Africa | Both | | | < 0.001 | | 0.98 | | < 0.001 | | 1 | | < 0.001 | | 0.995 | | | 0.61 | | 0.922 | 0.047 | | < 0.001 | | < 0.001 | < 0.001 | 0.906 | 0.003 |
| Central Sub-Saharan Africa | Female | | | < 0.001 | | 1 | | < 0.001 | | 0.92 | | < 0.001 | | 1 | | | 0.9 | | 0.999 | 0.43 | | < 0.001 | | < 0.001 | < 0.001 | 0.371 | 0.057 |
| Central Sub-Saharan Africa | Male | | | < 0.001 | | 1 | | < 0.001 | | 0.98 | | < 0.001 | | 1 | | | 0.56 | | 0.999 | 0.071 | | < 0.001 | | < 0.001 | < 0.001 | 0.55 | 0.006 |
| Southern Sub-Saharan Africa | Both | | | < 0.001 | | 0.88 | | < 0.001 | | 0.87 | | < 0.001 | | 0.932 | | | < 0.001 | | 0.76 | < 0.001 | | < 0.001 | | < 0.001 | < 0.001 | 0.537 | < 0.001 |
| Southern Sub-Saharan Africa | Female | | | < 0.001 | | 1 | | < 0.001 | | 0.37 | | < 0.001 | | 1 | | | 0.01 | | 0.976 | < 0.001 | | < 0.001 | | < 0.001 | < 0.001 | 0.044 | < 0.001 |
| Southern Sub-Saharan Africa | Male | | | < 0.001 | | 1 | | 0.799 | | 0.97 | | < 0.001 | | 1 | | | < 0.001 | | 1 | < 0.001 | | < 0.001 | | 0.01 | < 0.001 | 0.48 | 0.524 |
| Afghanistan | Both | | | < 0.001 | | < 0.001 | | < 0.001 | | < 0.001 | | < 0.001 | | < 0.001 | | | < 0.001 | | < 0.001 | < 0.001 | | < 0.001 | | < 0.001 | < 0.001 | < 0.001 | < 0.001 |
| Afghanistan | Female | | | < 0.001 | | < 0.001 | | < 0.001 | | 0 | | < 0.001 | | < 0.001 | | | 0.01 | | < 0.001 | < 0.001 | | < 0.001 | | < 0.001 | < 0.001 | 0.435 | 0.002 |
| Afghanistan | Male | | | < 0.001 | | < 0.001 | | < 0.001 | | < 0.001 | | < 0.001 | | < 0.001 | | | < 0.001 | | < 0.001 | < 0.001 | | < 0.001 | | < 0.001 | < 0.001 | < 0.001 | < 0.001 |
| Albania | Both | | | < 0.001 | | 0.23 | | 0.006 | | 0.01 | | < 0.001 | | 0.991 | | | 0.91 | | 0.116 | 0.059 | | 0.01 | | 0 | < 0.001 | 0.065 | 0.003 |
| Albania | Female | | | < 0.001 | | 0.99 | | 0.996 | | 0.46 | | < 0.001 | | 1 | | | 0.87 | | 0.973 | 0.725 | | 0.82 | | 0.56 | < 0.001 | 0.221 | 0.195 |
| Albania | Male | | | < 0.001 | | 1 | | 0.154 | | 0.93 | | < 0.001 | | 1 | | | 0.99 | | 1 | 0.314 | | 0.08 | | 0.01 | < 0.001 | 0.615 | 0.033 |
| Algeria | Both | | | < 0.001 | | < 0.001 | | < 0.001 | | 0.86 | | < 0.001 | | 0.253 | | | 0.49 | | < 0.001 | 0.64 | | < 0.001 | | < 0.001 | < 0.001 | 0.181 | 0.945 |
| Algeria | Female | | | < 0.001 | | 0.25 | | < 0.001 | | 0.97 | | < 0.001 | | 0.738 | | | 0.95 | | 0.141 | 0.913 | | 0.32 | | 0.02 | < 0.001 | 0.824 | 0.489 |
| Algeria | Male | | | < 0.001 | | < 0.001 | | < 0.001 | | 0.06 | | < 0.001 | | 0.027 | | | 0.04 | | < 0.001 | 0.085 | | < 0.001 | | < 0.001 | < 0.001 | 0.003 | 0.599 |
| American Samoa | Both | | | 0.866 | | 1 | | 1 | | 1 | | 1 | | 1 | | | 1 | | 1 | 1 | | 1 | | 0.92 | 0.037 | 0.996 | 0.959 |
| American Samoa | Female | | | 1 | | 1 | | 1 | | 1 | | 1 | | 1 | | | 1 | | 1 | 1 | | 1 | | 0.91 | 0.339 | 0.998 | 0.968 |
| American Samoa | Male | | | 0.935 | | 1 | | 1 | | 1 | | 1 | | 1 | | | 1 | | 1 | 1 | | 1 | | 0.92 | 0.082 | 0.994 | 0.952 |
| Andorra | Both | | | < 0.001 | | 1 | | 0.986 | | 1 | | 0.89 | | 1 | | | 0.92 | | 1 | 0.948 | | 0.62 | | 0.09 | 0.002 | 0.8 | 0.701 |
| Andorra | Female | | | 0.595 | | 1 | | 1 | | 1 | | 0.63 | | 1 | | | 0.98 | | 1 | 0.991 | | 0.93 | | 0.28 | 0.618 | 0.74 | 0.783 |
| Andorra | Male | | | 0.004 | | 1 | | 1 | | 1 | | 1 | | 1 | | | 0.96 | | 1 | 0.973 | | 0.89 | | 0.25 | 0.006 | 0.925 | 0.71 |
| Angola | Both | | | < 0.001 | | 1 | | 0.005 | | 1 | | < 0.001 | | 1 | | | 0.78 | | 1 | 0.75 | | < 0.001 | | < 0.001 | < 0.001 | 0.702 | 0.267 |
| Angola | Female | | | < 0.001 | | 1 | | 0.977 | | 1 | | < 0.001 | | 1 | | | 0.93 | | 1 | 0.951 | | 0.15 | | 0.01 | < 0.001 | 0.673 | 0.509 |
| Angola | Male | | | < 0.001 | | 1 | | 0.717 | | 1 | | < 0.001 | | 1 | | | 0.87 | | 1 | 0.795 | | 0.09 | | 0.01 | < 0.001 | 0.998 | 0.256 |
| Antigua and Barbuda | Both | | | < 0.001 | | 1 | | 1 | | 1 | | 0.12 | | 1 | | | 1 | | 1 | 1 | | 1 | | 0.74 | < 0.001 | 0.994 | 0.92 |
| Antigua and Barbuda | Female | | | 0.035 | | 1 | | 1 | | 1 | | 0.68 | | 1 | | | 1 | | 1 | 1 | | 1 | | 0.64 | 0.008 | 0.994 | 0.959 |
| Antigua and Barbuda | Male | | | < 0.001 | | 1 | | 1 | | 1 | | 0.76 | | 1 | | | 1 | | 1 | 1 | | 1 | | 0.9 | < 0.001 | 0.973 | 0.964 |
| Argentina | Both | | | < 0.001 | | < 0.001 | | < 0.001 | | < 0.001 | | < 0.001 | | 0.898 | | | 0.1 | | < 0.001 | < 0.001 | | < 0.001 | | < 0.001 | < 0.001 | < 0.001 | < 0.001 |
| Argentina | Female | | | < 0.001 | | < 0.001 | | < 0.001 | | < 0.001 | | < 0.001 | | 0.02 | | | 0.13 | | < 0.001 | < 0.001 | | < 0.001 | | < 0.001 | < 0.001 | < 0.001 | < 0.001 |
| Argentina | Male | | | < 0.001 | | < 0.001 | | < 0.001 | | < 0.001 | | < 0.001 | | 0.999 | | | 0.03 | | < 0.001 | < 0.001 | | < 0.001 | | < 0.001 | < 0.001 | < 0.001 | < 0.001 |
| Armenia | Both | | | < 0.001 | | < 0.001 | | < 0.001 | | 0 | | < 0.001 | | 0.12 | | | 0.02 | | < 0.001 | 0.005 | | < 0.001 | | < 0.001 | < 0.001 | 0.018 | 0.018 |
| Armenia | Female | | | < 0.001 | | < 0.001 | | < 0.001 | | 0.05 | | < 0.001 | | 0.15 | | | 0.09 | | < 0.001 | 0.075 | | 0 | | 0.01 | < 0.001 | 0.09 | 0.093 |
| Armenia | Male | | | < 0.001 | | < 0.001 | | < 0.001 | | 0.07 | | < 0.001 | | 0.843 | | | 0.16 | | < 0.001 | 0.046 | | < 0.001 | | < 0.001 | < 0.001 | 0.052 | 0.023 |
| Australia | Both | | | < 0.001 | | < 0.001 | | < 0.001 | | < 0.001 | | < 0.001 | | < 0.001 | | | 0.25 | | < 0.001 | 0.193 | | < 0.001 | | < 0.001 | < 0.001 | < 0.001 | 0.142 |
| Australia | Female | | | < 0.001 | | < 0.001 | | < 0.001 | | < 0.001 | | < 0.001 | | < 0.001 | | | < 0.001 | | < 0.001 | < 0.001 | | < 0.001 | | < 0.001 | < 0.001 | < 0.001 | 0.003 |
| Australia | Male | | | < 0.001 | | < 0.001 | | < 0.001 | | < 0.001 | | < 0.001 | | < 0.001 | | | 0 | | < 0.001 | < 0.001 | | < 0.001 | | < 0.001 | < 0.001 | < 0.001 | < 0.001 |
| Austria | Both | | | < 0.001 | | < 0.001 | | < 0.001 | | < 0.001 | | < 0.001 | | < 0.001 | | | < 0.001 | | < 0.001 | < 0.001 | | < 0.001 | | < 0.001 | < 0.001 | < 0.001 | 0.018 |
| Austria | Female | | | < 0.001 | | < 0.001 | | < 0.001 | | < 0.001 | | < 0.001 | | < 0.001 | | | < 0.001 | | < 0.001 | < 0.001 | | < 0.001 | | < 0.001 | 0.019 | < 0.001 | 0.049 |
| Austria | Male | | | < 0.001 | | < 0.001 | | < 0.001 | | < 0.001 | | < 0.001 | | < 0.001 | | | 0.15 | | < 0.001 | 0.252 | | < 0.001 | | < 0.001 | < 0.001 | < 0.001 | 0.737 |
| Azerbaijan | Both | | | < 0.001 | | < 0.001 | | < 0.001 | | < 0.001 | | < 0.001 | | < 0.001 | | | 0.06 | | < 0.001 | 0.089 | | 0.12 | | 0.54 | < 0.001 | < 0.001 | 0.673 |
| Azerbaijan | Female | | | < 0.001 | | < 0.001 | | < 0.001 | | < 0.001 | | < 0.001 | | < 0.001 | | | 0.19 | | < 0.001 | 0.02 | | 0.02 | | 0.41 | 0.054 | < 0.001 | 0.018 |
| Azerbaijan | Male | | | < 0.001 | | < 0.001 | | < 0.001 | | < 0.001 | | < 0.001 | | < 0.001 | | | 0.01 | | < 0.001 | 0.024 | | 0.03 | | 0.18 | < 0.001 | < 0.001 | 0.328 |
| Bahamas | Both | | | < 0.001 | | 1 | | 1 | | 1 | | < 0.001 | | 1 | | | 1 | | 1 | 0.999 | | 1 | | 0.7 | < 0.001 | 0.938 | 0.843 |
| Bahamas | Female | | | < 0.001 | | 1 | | 1 | | 1 | | < 0.001 | | 1 | | | 1 | | 1 | 1 | | 1 | | 0.9 | < 0.001 | 0.963 | 0.875 |
| Bahamas | Male | | | < 0.001 | | 1 | | 1 | | 1 | | < 0.001 | | 1 | | | 1 | | 1 | 1 | | 1 | | 0.61 | < 0.001 | 0.927 | 0.925 |
| Bahrain | Both | | | < 0.001 | | 1 | | 0.996 | | 1 | | < 0.001 | | 1 | | | 0.98 | | 1 | 0.797 | | 0.71 | | 0.18 | < 0.001 | 0.884 | 0.305 |
| Bahrain | Female | | | < 0.001 | | 1 | | 1 | | 1 | | 0.03 | | 1 | | | 1 | | 1 | 0.994 | | 0.87 | | 0.18 | < 0.001 | 0.885 | 0.722 |
| Bahrain | Male | | | < 0.001 | | 1 | | 0.999 | | 1 | | < 0.001 | | 1 | | | 0.98 | | 1 | 0.926 | | 0.86 | | 0.26 | < 0.001 | 0.956 | 0.505 |
| Bangladesh | Both | | | < 0.001 | | < 0.001 | | < 0.001 | | 0 | | < 0.001 | | < 0.001 | | | < 0.001 | | < 0.001 | < 0.001 | | < 0.001 | | < 0.001 | < 0.001 | 0.048 | < 0.001 |
| Bangladesh | Female | | | < 0.001 | | < 0.001 | | < 0.001 | | < 0.001 | | < 0.001 | | < 0.001 | | | 0 | | < 0.001 | < 0.001 | | < 0.001 | | < 0.001 | < 0.001 | 0.012 | 0.003 |
| Bangladesh | Male | | | < 0.001 | | < 0.001 | | < 0.001 | | 0.03 | | < 0.001 | | < 0.001 | | | < 0.001 | | < 0.001 | < 0.001 | | < 0.001 | | < 0.001 | < 0.001 | 0.382 | < 0.001 |
| Barbados | Both | | | < 0.001 | | 1 | | 1 | | 1 | | < 0.001 | | 1 | | | 1 | | 1 | 0.999 | | 1 | | 0.76 | < 0.001 | 0.919 | 0.932 |
| Barbados | Female | | | < 0.001 | | 1 | | 1 | | 1 | | < 0.001 | | 1 | | | 1 | | 1 | 1 | | 1 | | 0.8 | < 0.001 | 0.952 | 0.959 |
| Barbados | Male | | | < 0.001 | | 1 | | 1 | | 1 | | < 0.001 | | 1 | | | 1 | | 1 | 1 | | 1 | | 0.63 | < 0.001 | 0.909 | 0.917 |
| Belarus | Both | | | < 0.001 | | 0.15 | | 0.157 | | 0.6 | | < 0.001 | | 0.254 | | | 0.86 | | 0.144 | < 0.001 | | < 0.001 | | 0.42 | < 0.001 | 0.812 | < 0.001 |
| Belarus | Female | | | < 0.001 | | 0.66 | | < 0.001 | | 0.98 | | < 0.001 | | 0.63 | | | < 0.001 | | 0.743 | < 0.001 | | < 0.001 | | 0.01 | 0.008 | 0.38 | < 0.001 |
| Belarus | Male | | | < 0.001 | | < 0.001 | | < 0.001 | | 0.07 | | < 0.001 | | < 0.001 | | | 0.01 | | < 0.001 | < 0.001 | | < 0.001 | | 0.15 | < 0.001 | 0.824 | < 0.001 |
| Belgium | Both | | | < 0.001 | | < 0.001 | | < 0.001 | | < 0.001 | | < 0.001 | | 0.052 | | | 0.22 | | < 0.001 | 0.092 | | < 0.001 | | < 0.001 | < 0.001 | < 0.001 | 0.065 |
| Belgium | Female | | | < 0.001 | | < 0.001 | | < 0.001 | | < 0.001 | | < 0.001 | | 0.069 | | | 0.02 | | < 0.001 | 0.048 | | < 0.001 | | < 0.001 | < 0.001 | < 0.001 | 0.894 |
| Belgium | Male | | | < 0.001 | | < 0.001 | | < 0.001 | | < 0.001 | | < 0.001 | | 0.085 | | | 0.48 | | < 0.001 | 0.018 | | < 0.001 | | < 0.001 | < 0.001 | < 0.001 | 0.002 |
| Belize | Both | | | < 0.001 | | 1 | | 1 | | 1 | | < 0.001 | | 1 | | | 1 | | 1 | 0.993 | | 0.98 | | 0.43 | < 0.001 | 0.936 | 0.674 |
| Belize | Female | | | < 0.001 | | 1 | | 1 | | 1 | | 0.01 | | 1 | | | 1 | | 1 | 1 | | 1 | | 0.81 | < 0.001 | 0.958 | 0.823 |
| Belize | Male | | | < 0.001 | | 1 | | 1 | | 1 | | 0 | | 1 | | | 1 | | 1 | 0.997 | | 0.97 | | 0.36 | < 0.001 | 0.896 | 0.731 |
| Benin | Both | | | < 0.001 | | 1 | | 1 | | 0.98 | | < 0.001 | | 1 | | | 0.99 | | 1 | 0.999 | | 0.66 | | 0.08 | < 0.001 | 0.335 | 0.974 |
| Benin | Female | | | < 0.001 | | 1 | | 1 | | 1 | | < 0.001 | | 1 | | | 1 | | 1 | 1 | | 0.93 | | 0.26 | < 0.001 | 0.868 | 0.901 |
| Benin | Male | | | < 0.001 | | 1 | | 1 | | 1 | | < 0.001 | | 1 | | | 1 | | 1 | 1 | | 0.87 | | 0.2 | < 0.001 | 0.6 | 0.977 |
| Bermuda | Both | | | < 0.001 | | 1 | | 1 | | 1 | | 0.21 | | 1 | | | 1 | | 1 | 1 | | 0.99 | | 0.51 | < 0.001 | 0.957 | 0.968 |
| Bermuda | Female | | | 0.008 | | 1 | | 1 | | 1 | | 0.79 | | 1 | | | 1 | | 1 | 1 | | 1 | | 0.52 | 0.031 | 0.985 | 0.979 |
| Bermuda | Male | | | < 0.001 | | 1 | | 1 | | 1 | | 0.79 | | 1 | | | 1 | | 1 | 1 | | 1 | | 0.73 | 0.004 | 0.966 | 0.988 |
| Bhutan | Both | | | < 0.001 | | 1 | | 1 | | 1 | | < 0.001 | | 1 | | | 1 | | 1 | 0.997 | | 1 | | 1 | < 0.001 | 0.705 | 0.743 |
| Bhutan | Female | | | < 0.001 | | 1 | | 1 | | 1 | | < 0.001 | | 1 | | | 1 | | 1 | 0.999 | | 1 | | 0.76 | < 0.001 | 0.943 | 0.781 |
| Bhutan | Male | | | < 0.001 | | 1 | | 1 | | 1 | | < 0.001 | | 1 | | | 1 | | 1 | 1 | | 1 | | 0.88 | < 0.001 | 0.938 | 0.994 |
| Bolivia (Plurinational State of) | Both | | | < 0.001 | | 1 | | 1 | | 0.96 | | < 0.001 | | 1 | | | 0.86 | | 1 | 0.549 | | 0.62 | | 0.77 | < 0.001 | 0.528 | 0.107 |
| Bolivia (Plurinational State of) | Female | | | < 0.001 | | 1 | | 1 | | 0.96 | | < 0.001 | | 1 | | | 0.91 | | 1 | 0.802 | | 0.87 | | 0.84 | 0.178 | 0.349 | 0.245 |
| Bolivia (Plurinational State of) | Male | | | < 0.001 | | 1 | | 1 | | 1 | | < 0.001 | | 1 | | | 0.96 | | 1 | 0.84 | | 0.92 | | 0.78 | < 0.001 | 0.911 | 0.271 |
| Bosnia and Herzegovina | Both | | | < 0.001 | | 0.55 | | < 0.001 | | 0.97 | | < 0.001 | | 0.549 | | | 0.28 | | 0.478 | 0.218 | | < 0.001 | | < 0.001 | < 0.001 | 0.934 | 0.204 |
| Bosnia and Herzegovina | Female | | | < 0.001 | | 0.08 | | < 0.001 | | 0.34 | | < 0.001 | | 0.119 | | | 0.84 | | 0.049 | 0.067 | | 0.03 | | 0.03 | < 0.001 | 0.552 | 0.006 |
| Bosnia and Herzegovina | Male | | | < 0.001 | | 0.35 | | < 0.001 | | 0.71 | | < 0.001 | | 0.999 | | | 0.28 | | 0.212 | 0.394 | | < 0.001 | | < 0.001 | < 0.001 | 0.24 | 0.56 |
| Botswana | Both | | | < 0.001 | | 1 | | 1 | | 1 | | < 0.001 | | 1 | | | 1 | | 1 | 0.994 | | 0.97 | | 0.39 | < 0.001 | 0.861 | 0.69 |
| Botswana | Female | | | < 0.001 | | 1 | | 1 | | 1 | | 0 | | 1 | | | 1 | | 1 | 0.966 | | 0.99 | | 0.78 | < 0.001 | 0.924 | 0.503 |
| Botswana | Male | | | < 0.001 | | 1 | | 1 | | 1 | | < 0.001 | | 1 | | | 1 | | 1 | 1 | | 0.98 | | 0.4 | < 0.001 | 0.968 | 0.934 |
| Brazil | Both | | | < 0.001 | | < 0.001 | | < 0.001 | | < 0.001 | | < 0.001 | | < 0.001 | | | 0.06 | | < 0.001 | 0.001 | | < 0.001 | | < 0.001 | < 0.001 | < 0.001 | < 0.001 |
| Brazil | Female | | | < 0.001 | | < 0.001 | | < 0.001 | | < 0.001 | | < 0.001 | | < 0.001 | | | 0.24 | | < 0.001 | 0.132 | | < 0.001 | | < 0.001 | < 0.001 | < 0.001 | 0.047 |
| Brazil | Male | | | < 0.001 | | < 0.001 | | < 0.001 | | < 0.001 | | < 0.001 | | < 0.001 | | | 0.02 | | < 0.001 | < 0.001 | | < 0.001 | | < 0.001 | < 0.001 | < 0.001 | < 0.001 |
| Brunei Darussalam | Both | | | 0.018 | | 1 | | 1 | | 1 | | 0.22 | | 1 | | | 1 | | 1 | 0.992 | | 0.91 | | 0.23 | 0.037 | 0.871 | 0.664 |
| Brunei Darussalam | Female | | | 0.004 | | 1 | | 1 | | 1 | | 0.02 | | 1 | | | 1 | | 1 | 1 | | 0.97 | | 0.34 | 0.776 | 0.73 | 0.884 |
| Brunei Darussalam | Male | | | 0.004 | | 1 | | 1 | | 1 | | 0.84 | | 1 | | | 1 | | 1 | 0.997 | | 1 | | 0.73 | 0.11 | 0.985 | 0.714 |
| Bulgaria | Both | | | < 0.001 | | < 0.001 | | < 0.001 | | < 0.001 | | < 0.001 | | < 0.001 | | | < 0.001 | | < 0.001 | < 0.001 | | < 0.001 | | < 0.001 | < 0.001 | < 0.001 | < 0.001 |
| Bulgaria | Female | | | < 0.001 | | < 0.001 | | < 0.001 | | < 0.001 | | < 0.001 | | < 0.001 | | | < 0.001 | | < 0.001 | < 0.001 | | < 0.001 | | < 0.001 | < 0.001 | < 0.001 | < 0.001 |
| Bulgaria | Male | | | < 0.001 | | < 0.001 | | < 0.001 | | < 0.001 | | < 0.001 | | 0.014 | | | < 0.001 | | < 0.001 | < 0.001 | | < 0.001 | | < 0.001 | < 0.001 | < 0.001 | < 0.001 |
| Burkina Faso | Both | | | < 0.001 | | 1 | | 0.981 | | 1 | | < 0.001 | | 1 | | | 1 | | 1 | 0.615 | | 0.44 | | 0.08 | < 0.001 | 0.788 | 0.104 |
| Burkina Faso | Female | | | < 0.001 | | 1 | | 0.998 | | 1 | | < 0.001 | | 1 | | | 1 | | 1 | 0.894 | | 0.84 | | 0.25 | < 0.001 | 0.438 | 0.295 |
| Burkina Faso | Male | | | < 0.001 | | 1 | | 0.998 | | 1 | | < 0.001 | | 1 | | | 1 | | 1 | 0.96 | | 0.71 | | 0.1 | < 0.001 | 0.728 | 0.433 |
| Burundi | Both | | | < 0.001 | | 1 | | 1 | | 1 | | < 0.001 | | 1 | | | 0.99 | | 1 | 0.765 | | 0.8 | | 0.38 | < 0.001 | 0.811 | 0.178 |
| Burundi | Female | | | < 0.001 | | 1 | | 1 | | 1 | | < 0.001 | | 1 | | | 1 | | 1 | 0.931 | | 0.97 | | 0.86 | < 0.001 | 0.984 | 0.363 |
| Burundi | Male | | | < 0.001 | | 1 | | 1 | | 1 | | < 0.001 | | 1 | | | 0.99 | | 1 | 0.932 | | 0.97 | | 0.84 | < 0.001 | 0.825 | 0.371 |
| Cabo Verde | Both | | | < 0.001 | | 1 | | 1 | | 1 | | 0.05 | | 1 | | | 1 | | 1 | 0.999 | | 0.98 | | 0.4 | < 0.001 | 0.746 | 0.798 |
| Cabo Verde | Female | | | < 0.001 | | 1 | | 1 | | 1 | | 0.64 | | 1 | | | 1 | | 1 | 0.999 | | 1 | | 0.74 | < 0.001 | 0.98 | 0.781 |
| Cabo Verde | Male | | | < 0.001 | | 1 | | 1 | | 1 | | 0.62 | | 1 | | | 1 | | 1 | 1 | | 1 | | 0.65 | < 0.001 | 0.877 | 0.811 |
| Cambodia | Both | | | < 0.001 | | 0.6 | | 0.01 | | 0.37 | | < 0.001 | | 0.609 | | | 0.91 | | 0.415 | 0.97 | | 0.09 | | 0 | < 0.001 | 0.157 | 0.981 |
| Cambodia | Female | | | < 0.001 | | 0.99 | | 0.917 | | 0.54 | | < 0.001 | | 1 | | | 0.94 | | 0.951 | 0.98 | | 0.58 | | 0.08 | < 0.001 | 0.108 | 0.861 |
| Cambodia | Male | | | < 0.001 | | 1 | | 0.287 | | 0.75 | | < 0.001 | | 1 | | | 0.97 | | 0.993 | 0.993 | | 0.18 | | 0.01 | < 0.001 | 0.228 | 0.997 |
| Cameroon | Both | | | < 0.001 | | 1 | | < 0.001 | | 0.95 | | < 0.001 | | 1 | | | 0.72 | | 1 | < 0.001 | | < 0.001 | | < 0.001 | < 0.001 | 0.284 | < 0.001 |
| Cameroon | Female | | | < 0.001 | | 1 | | 0.015 | | 1 | | < 0.001 | | 1 | | | 0.89 | | 1 | 0.006 | | < 0.001 | | < 0.001 | < 0.001 | 0.969 | < 0.001 |
| Cameroon | Male | | | < 0.001 | | 1 | | < 0.001 | | 0.99 | | < 0.001 | | 1 | | | 0.86 | | 1 | 0.001 | | < 0.001 | | < 0.001 | < 0.001 | 0.42 | < 0.001 |
| Canada | Both | | | < 0.001 | | < 0.001 | | < 0.001 | | < 0.001 | | < 0.001 | | < 0.001 | | | < 0.001 | | < 0.001 | < 0.001 | | < 0.001 | | < 0.001 | < 0.001 | < 0.001 | < 0.001 |
| Canada | Female | | | < 0.001 | | < 0.001 | | < 0.001 | | < 0.001 | | < 0.001 | | < 0.001 | | | < 0.001 | | < 0.001 | < 0.001 | | < 0.001 | | < 0.001 | < 0.001 | < 0.001 | < 0.001 |
| Canada | Male | | | < 0.001 | | < 0.001 | | < 0.001 | | < 0.001 | | < 0.001 | | < 0.001 | | | < 0.001 | | < 0.001 | < 0.001 | | < 0.001 | | < 0.001 | < 0.001 | < 0.001 | < 0.001 |
| Central African Republic | Both | | | < 0.001 | | 1 | | 1 | | 1 | | < 0.001 | | 1 | | | 1 | | 1 | 1 | | 1 | | 0.58 | < 0.001 | 0.887 | 0.932 |
| Central African Republic | Female | | | < 0.001 | | 1 | | 1 | | 1 | | < 0.001 | | 1 | | | 1 | | 1 | 1 | | 1 | | 0.75 | < 0.001 | 0.838 | 0.84 |
| Central African Republic | Male | | | < 0.001 | | 1 | | 1 | | 1 | | < 0.001 | | 1 | | | 1 | | 1 | 1 | | 1 | | 0.73 | < 0.001 | 0.975 | 0.995 |
| Chad | Both | | | < 0.001 | | 1 | | 0.01 | | 1 | | < 0.001 | | 1 | | | 0.94 | | 1 | 0.915 | | < 0.001 | | < 0.001 | < 0.001 | 0.68 | 0.499 |
| Chad | Female | | | < 0.001 | | 1 | | 0.96 | | 1 | | < 0.001 | | 1 | | | 0.99 | | 1 | 0.991 | | 0.13 | | 0 | < 0.001 | 0.959 | 0.727 |
| Chad | Male | | | < 0.001 | | 1 | | 0.929 | | 1 | | < 0.001 | | 1 | | | 0.98 | | 1 | 0.975 | | 0.1 | | 0 | < 0.001 | 0.506 | 0.619 |
| Chile | Both | | | < 0.001 | | < 0.001 | | < 0.001 | | < 0.001 | | < 0.001 | | 0.651 | | | 0.57 | | < 0.001 | < 0.001 | | < 0.001 | | < 0.001 | < 0.001 | < 0.001 | < 0.001 |
| Chile | Female | | | < 0.001 | | 0.05 | | < 0.001 | | 0 | | < 0.001 | | 0.998 | | | 0.64 | | 0.016 | < 0.001 | | < 0.001 | | < 0.001 | 0.013 | < 0.001 | < 0.001 |
| Chile | Male | | | < 0.001 | | < 0.001 | | < 0.001 | | < 0.001 | | < 0.001 | | 0.134 | | | 0.18 | | < 0.001 | < 0.001 | | < 0.001 | | < 0.001 | < 0.001 | < 0.001 | < 0.001 |
| China | Both | | | < 0.001 | | < 0.001 | | < 0.001 | | < 0.001 | | < 0.001 | | < 0.001 | | | < 0.001 | | < 0.001 | < 0.001 | | < 0.001 | | < 0.001 | < 0.001 | < 0.001 | 0.303 |
| China | Female | | | < 0.001 | | < 0.001 | | < 0.001 | | < 0.001 | | < 0.001 | | < 0.001 | | | < 0.001 | | < 0.001 | < 0.001 | | < 0.001 | | < 0.001 | 0.642 | < 0.001 | 0.036 |
| China | Male | | | < 0.001 | | < 0.001 | | < 0.001 | | < 0.001 | | < 0.001 | | < 0.001 | | | < 0.001 | | < 0.001 | < 0.001 | | < 0.001 | | < 0.001 | < 0.001 | < 0.001 | 0.709 |
| Colombia | Both | | | < 0.001 | | < 0.001 | | < 0.001 | | < 0.001 | | < 0.001 | | < 0.001 | | | 0.02 | | < 0.001 | < 0.001 | | < 0.001 | | < 0.001 | < 0.001 | < 0.001 | 0.003 |
| Colombia | Female | | | < 0.001 | | < 0.001 | | < 0.001 | | 0.14 | | < 0.001 | | < 0.001 | | | 0.28 | | < 0.001 | 0.004 | | < 0.001 | | < 0.001 | < 0.001 | 0.003 | < 0.001 |
| Colombia | Male | | | < 0.001 | | < 0.001 | | < 0.001 | | < 0.001 | | < 0.001 | | < 0.001 | | | 0.03 | | < 0.001 | 0.031 | | < 0.001 | | < 0.001 | < 0.001 | < 0.001 | 0.187 |
| Comoros | Both | | | < 0.001 | | 1 | | 1 | | 1 | | 0.04 | | 1 | | | 1 | | 1 | 1 | | 1 | | 0.71 | < 0.001 | 0.932 | 0.991 |
| Comoros | Female | | | < 0.001 | | 1 | | 1 | | 1 | | 0.77 | | 1 | | | 1 | | 1 | 1 | | 1 | | 0.74 | < 0.001 | 0.995 | 0.951 |
| Comoros | Male | | | < 0.001 | | 1 | | 1 | | 1 | | 0.36 | | 1 | | | 1 | | 1 | 1 | | 1 | | 0.87 | < 0.001 | 0.993 | 0.969 |
| Congo | Both | | | < 0.001 | | 1 | | 1 | | 1 | | < 0.001 | | 1 | | | 1 | | 1 | 1 | | 1 | | 0.54 | < 0.001 | 0.8 | 0.897 |
| Congo | Female | | | < 0.001 | | 1 | | 1 | | 1 | | < 0.001 | | 1 | | | 1 | | 1 | 1 | | 0.99 | | 0.46 | < 0.001 | 0.776 | 0.985 |
| Congo | Male | | | < 0.001 | | 1 | | 1 | | 1 | | < 0.001 | | 1 | | | 1 | | 1 | 0.999 | | 0.99 | | 0.55 | < 0.001 | 0.99 | 0.808 |
| Cook Islands | Both | | | 0.999 | | 1 | | 1 | | 1 | | 1 | | 1 | | | 1 | | 1 | 1 | | 1 | | 0.96 | 0.18 | 0.993 | 0.978 |
| Cook Islands | Female | | | 1 | | 1 | | 1 | | 1 | | 1 | | 1 | | | 1 | | 1 | 1 | | 1 | | 0.98 | 0.555 | 0.994 | 0.988 |
| Cook Islands | Male | | | 0.999 | | 1 | | 1 | | 1 | | 1 | | 1 | | | 1 | | 1 | 1 | | 1 | | 0.99 | 0.231 | 0.998 | 0.978 |
| Costa Rica | Both | | | < 0.001 | | 1 | | 0.087 | | 0.8 | | < 0.001 | | 1 | | | 0.97 | | 0.999 | 0.814 | | 0 | | < 0.001 | < 0.001 | 0.291 | 0.222 |
| Costa Rica | Female | | | < 0.001 | | 1 | | 0.603 | | 1 | | < 0.001 | | 1 | | | 0.97 | | 1 | 0.947 | | 0.02 | | 0 | < 0.001 | 0.679 | 0.431 |
| Costa Rica | Male | | | < 0.001 | | 1 | | 0.987 | | 0.88 | | < 0.001 | | 1 | | | 0.99 | | 1 | 0.95 | | 0.51 | | 0.1 | < 0.001 | 0.351 | 0.419 |
| Croatia | Both | | | < 0.001 | | < 0.001 | | < 0.001 | | < 0.001 | | < 0.001 | | 0.657 | | | 0.51 | | < 0.001 | 0.033 | | < 0.001 | | < 0.001 | < 0.001 | 0.157 | 0.004 |
| Croatia | Female | | | < 0.001 | | 0.03 | | < 0.001 | | 0.48 | | < 0.001 | | 0.447 | | | 0.22 | | 0.01 | < 0.001 | | < 0.001 | | < 0.001 | 0.002 | 0.426 | < 0.001 |
| Croatia | Male | | | < 0.001 | | < 0.001 | | < 0.001 | | < 0.001 | | < 0.001 | | < 0.001 | | | 0.66 | | < 0.001 | 0.764 | | < 0.001 | | < 0.001 | < 0.001 | < 0.001 | 0.6 |
| Cuba | Both | | | < 0.001 | | < 0.001 | | < 0.001 | | < 0.001 | | < 0.001 | | < 0.001 | | | 0.44 | | < 0.001 | 0.237 | | < 0.001 | | < 0.001 | < 0.001 | < 0.001 | 0.124 |
| Cuba | Female | | | < 0.001 | | < 0.001 | | < 0.001 | | < 0.001 | | < 0.001 | | < 0.001 | | | 0.11 | | < 0.001 | < 0.001 | | < 0.001 | | < 0.001 | < 0.001 | < 0.001 | < 0.001 |
| Cuba | Male | | | < 0.001 | | < 0.001 | | < 0.001 | | < 0.001 | | < 0.001 | | < 0.001 | | | 0.76 | | < 0.001 | 0.831 | | < 0.001 | | < 0.001 | < 0.001 | < 0.001 | 0.544 |
| Cyprus | Both | | | < 0.001 | | 0 | | < 0.001 | | 0.25 | | < 0.001 | | 0.751 | | | 0.28 | | 0.001 | 0.213 | | < 0.001 | | < 0.001 | < 0.001 | 0.061 | 0.226 |
| Cyprus | Female | | | < 0.001 | | 0.83 | | < 0.001 | | 0.61 | | < 0.001 | | 1 | | | 0.42 | | 0.664 | 0.104 | | 0.01 | | 0 | 0.311 | 0.213 | 0.035 |
| Cyprus | Male | | | < 0.001 | | 0.56 | | < 0.001 | | 0.96 | | < 0.001 | | 0.998 | | | 0.7 | | 0.368 | 0.789 | | 0.02 | | < 0.001 | < 0.001 | 0.301 | 0.681 |
| Czechia | Both | | | < 0.001 | | < 0.001 | | < 0.001 | | 0.2 | | < 0.001 | | < 0.001 | | | 0 | | < 0.001 | < 0.001 | | < 0.001 | | < 0.001 | < 0.001 | < 0.001 | < 0.001 |
| Czechia | Female | | | < 0.001 | | < 0.001 | | < 0.001 | | 0.53 | | < 0.001 | | < 0.001 | | | 0.01 | | < 0.001 | < 0.001 | | < 0.001 | | < 0.001 | < 0.001 | 0.007 | < 0.001 |
| Czechia | Male | | | < 0.001 | | < 0.001 | | < 0.001 | | 0.01 | | < 0.001 | | < 0.001 | | | < 0.001 | | < 0.001 | < 0.001 | | < 0.001 | | < 0.001 | < 0.001 | < 0.001 | < 0.001 |
| C么te d'Ivoire | Both | | | < 0.001 | | 0.92 | | 0.897 | | 0.91 | | < 0.001 | | 1 | | | 0.97 | | 0.815 | < 0.001 | | < 0.001 | | 0.08 | < 0.001 | 0.258 | < 0.001 |
| C么te d'Ivoire | Female | | | < 0.001 | | 1 | | 1 | | 1 | | < 0.001 | | 1 | | | 1 | | 1 | 0.092 | | 0.07 | | 0.04 | < 0.001 | 0.981 | 0.006 |
| C么te d'Ivoire | Male | | | < 0.001 | | 0.95 | | 0.967 | | 0.77 | | < 0.001 | | 1 | | | 0.92 | | 0.869 | < 0.001 | | 0 | | 0.45 | < 0.001 | 0.271 | < 0.001 |
| Democratic People's Republic of Korea | Both | | | < 0.001 | | 0.3 | | < 0.001 | | 0.94 | | < 0.001 | | 0.308 | | | < 0.001 | | 0.299 | < 0.001 | | < 0.001 | | < 0.001 | < 0.001 | 0.359 | < 0.001 |
| Democratic People's Republic of Korea | Female | | | < 0.001 | | 0.98 | | < 0.001 | | 0.48 | | < 0.001 | | 0.994 | | | 0 | | 0.949 | < 0.001 | | < 0.001 | | < 0.001 | < 0.001 | 0.644 | < 0.001 |
| Democratic People's Republic of Korea | Male | | | < 0.001 | | 0.92 | | < 0.001 | | 0.94 | | < 0.001 | | 0.942 | | | 0.08 | | 0.823 | < 0.001 | | < 0.001 | | < 0.001 | < 0.001 | 0.66 | < 0.001 |
| Democratic Republic of the Congo | Both | | | < 0.001 | | 0.76 | | < 0.001 | | 1 | | < 0.001 | | 0.987 | | | 0.73 | | 0.58 | 0.056 | | < 0.001 | | < 0.001 | < 0.001 | 0.508 | 0.003 |
| Democratic Republic of the Congo | Female | | | < 0.001 | | 1 | | 0.002 | | 0.98 | | < 0.001 | | 1 | | | 0.94 | | 1 | 0.435 | | < 0.001 | | < 0.001 | < 0.001 | 0.593 | 0.057 |
| Democratic Republic of the Congo | Male | | | < 0.001 | | 1 | | < 0.001 | | 0.91 | | < 0.001 | | 1 | | | 0.64 | | 0.99 | 0.088 | | < 0.001 | | < 0.001 | < 0.001 | 0.395 | 0.007 |
| Denmark | Both | | | < 0.001 | | < 0.001 | | < 0.001 | | 0.01 | | < 0.001 | | 0.112 | | | 0.28 | | < 0.001 | 0.16 | | < 0.001 | | < 0.001 | < 0.001 | < 0.001 | 0.107 |
| Denmark | Female | | | < 0.001 | | 0.06 | | < 0.001 | | 0.01 | | < 0.001 | | 0.58 | | | 0.76 | | 0.025 | 0.64 | | < 0.001 | | < 0.001 | < 0.001 | < 0.001 | 0.239 |
| Denmark | Male | | | < 0.001 | | < 0.001 | | < 0.001 | | 0.12 | | < 0.001 | | < 0.001 | | | 0.08 | | < 0.001 | < 0.001 | | < 0.001 | | < 0.001 | < 0.001 | 0.007 | < 0.001 |
| Djibouti | Both | | | < 0.001 | | 1 | | 1 | | 1 | | 0 | | 1 | | | 1 | | 1 | 0.992 | | 0.98 | | 0.46 | < 0.001 | 0.954 | 0.621 |
| Djibouti | Female | | | 0.007 | | 1 | | 1 | | 1 | | 0.71 | | 1 | | | 1 | | 1 | 0.999 | | 1 | | 0.73 | < 0.001 | 0.976 | 0.751 |
| Djibouti | Male | | | < 0.001 | | 1 | | 1 | | 1 | | 0.13 | | 1 | | | 1 | | 1 | 0.997 | | 1 | | 0.61 | < 0.001 | 0.989 | 0.707 |
| Dominica | Both | | | < 0.001 | | 1 | | 1 | | 1 | | 0.2 | | 1 | | | 1 | | 1 | 1 | | 1 | | 0.74 | < 0.001 | 0.969 | 0.975 |
| Dominica | Female | | | 0.024 | | 1 | | 1 | | 1 | | 0.83 | | 1 | | | 1 | | 1 | 1 | | 1 | | 0.94 | 0.008 | 0.98 | 0.969 |
| Dominica | Male | | | < 0.001 | | 1 | | 1 | | 1 | | 0.74 | | 1 | | | 1 | | 1 | 1 | | 1 | | 0.79 | 0.001 | 0.964 | 0.988 |
| Dominican Republic | Both | | | < 0.001 | | 0.01 | | < 0.001 | | < 0.001 | | < 0.001 | | 0.062 | | | 0.34 | | 0.003 | 0.491 | | < 0.001 | | < 0.001 | < 0.001 | 0.006 | 0.848 |
| Dominican Republic | Female | | | < 0.001 | | < 0.001 | | < 0.001 | | < 0.001 | | < 0.001 | | 0.011 | | | 0.41 | | < 0.001 | 0.549 | | 0.01 | | < 0.001 | < 0.001 | 0.001 | 0.452 |
| Dominican Republic | Male | | | < 0.001 | | 1 | | < 0.001 | | 0.55 | | < 0.001 | | 1 | | | 0.87 | | 0.989 | 0.94 | | < 0.001 | | < 0.001 | < 0.001 | 0.234 | 0.92 |
| Ecuador | Both | | | < 0.001 | | 1 | | 0.999 | | 0.92 | | < 0.001 | | 0.998 | | | 0.9 | | 0.997 | < 0.001 | | < 0.001 | | 0.93 | < 0.001 | 0.224 | < 0.001 |
| Ecuador | Female | | | < 0.001 | | 1 | | 1 | | 0.98 | | < 0.001 | | 1 | | | 0.94 | | 1 | < 0.001 | | < 0.001 | | 0.66 | < 0.001 | 0.453 | < 0.001 |
| Ecuador | Male | | | < 0.001 | | 1 | | 0.995 | | 0.83 | | < 0.001 | | 1 | | | 0.95 | | 0.997 | < 0.001 | | < 0.001 | | 0.4 | < 0.001 | 0.171 | < 0.001 |
| Egypt | Both | | | < 0.001 | | < 0.001 | | < 0.001 | | 0.26 | | < 0.001 | | < 0.001 | | | < 0.001 | | < 0.001 | < 0.001 | | < 0.001 | | 0.39 | < 0.001 | 0.076 | 0.03 |
| Egypt | Female | | | < 0.001 | | < 0.001 | | < 0.001 | | < 0.001 | | < 0.001 | | 0.559 | | | < 0.001 | | < 0.001 | < 0.001 | | < 0.001 | | < 0.001 | < 0.001 | < 0.001 | < 0.001 |
| Egypt | Male | | | < 0.001 | | < 0.001 | | < 0.001 | | < 0.001 | | < 0.001 | | < 0.001 | | | < 0.001 | | < 0.001 | < 0.001 | | < 0.001 | | < 0.001 | < 0.001 | < 0.001 | 0.145 |
| El Salvador | Both | | | < 0.001 | | 0.93 | | 0.824 | | 0.17 | | < 0.001 | | 0.98 | | | 0.94 | | 0.829 | 0.979 | | 0.77 | | 0.15 | < 0.001 | 0.425 | 0.911 |
| El Salvador | Female | | | < 0.001 | | 0.99 | | 0.981 | | 0.38 | | < 0.001 | | 1 | | | 0.98 | | 0.953 | 0.992 | | 0.99 | | 0.7 | < 0.001 | 0.294 | 0.745 |
| El Salvador | Male | | | < 0.001 | | 1 | | 0.913 | | 0.94 | | < 0.001 | | 1 | | | 0.96 | | 1 | 0.962 | | 0.13 | | 0 | < 0.001 | 0.872 | 0.636 |
| Equatorial Guinea | Both | | | < 0.001 | | 1 | | 0.999 | | 1 | | 0.01 | | 1 | | | 0.98 | | 1 | 0.969 | | 0.56 | | 0.05 | < 0.001 | 0.989 | 0.605 |
| Equatorial Guinea | Female | | | < 0.001 | | 1 | | 1 | | 1 | | 0.34 | | 1 | | | 0.99 | | 1 | 0.992 | | 0.77 | | 0.12 | < 0.001 | 0.894 | 0.743 |
| Equatorial Guinea | Male | | | < 0.001 | | 1 | | 1 | | 1 | | 0.28 | | 1 | | | 0.99 | | 1 | 0.996 | | 0.9 | | 0.22 | < 0.001 | 0.955 | 0.818 |
| Eritrea | Both | | | < 0.001 | | 1 | | 1 | | 1 | | < 0.001 | | 1 | | | 1 | | 1 | 1 | | 1 | | 0.67 | < 0.001 | 0.976 | 0.909 |
| Eritrea | Female | | | < 0.001 | | 1 | | 1 | | 1 | | < 0.001 | | 1 | | | 1 | | 1 | 1 | | 1 | | 0.54 | < 0.001 | 0.944 | 0.993 |
| Eritrea | Male | | | < 0.001 | | 1 | | 1 | | 1 | | < 0.001 | | 1 | | | 1 | | 1 | 1 | | 1 | | 0.79 | < 0.001 | 0.998 | 0.914 |
| Estonia | Both | | | < 0.001 | | < 0.001 | | < 0.001 | | < 0.001 | | < 0.001 | | < 0.001 | | | 0.57 | | < 0.001 | 0.069 | | 0.08 | | 0.44 | < 0.001 | 0.015 | 0.008 |
| Estonia | Female | | | < 0.001 | | < 0.001 | | < 0.001 | | < 0.001 | | < 0.001 | | < 0.001 | | | 0.91 | | < 0.001 | 0.189 | | 0.19 | | 0.17 | 0.028 | 0.07 | 0.02 |
| Estonia | Male | | | < 0.001 | | < 0.001 | | < 0.001 | | < 0.001 | | < 0.001 | | < 0.001 | | | 0.27 | | < 0.001 | 0.07 | | 0 | | 0 | < 0.001 | 0.045 | 0.023 |
| Eswatini | Both | | | < 0.001 | | 1 | | 1 | | 1 | | < 0.001 | | 1 | | | 0.99 | | 1 | 0.963 | | 0.94 | | 0.38 | < 0.001 | 0.877 | 0.504 |
| Eswatini | Female | | | < 0.001 | | 1 | | 1 | | 1 | | 0.22 | | 1 | | | 1 | | 1 | 0.958 | | 0.98 | | 0.7 | < 0.001 | 0.96 | 0.466 |
| Eswatini | Male | | | < 0.001 | | 1 | | 1 | | 1 | | 0.03 | | 1 | | | 1 | | 1 | 0.999 | | 0.98 | | 0.41 | < 0.001 | 0.983 | 0.852 |
| Ethiopia | Both | | | < 0.001 | | < 0.001 | | < 0.001 | | 0.01 | | < 0.001 | | 0.028 | | | 0.22 | | < 0.001 | < 0.001 | | < 0.001 | | 0 | < 0.001 | 0.75 | < 0.001 |
| Ethiopia | Female | | | < 0.001 | | 1 | | 1 | | 0.98 | | < 0.001 | | 1 | | | 0.95 | | 1 | 0.281 | | 0.41 | | 0.62 | < 0.001 | 0.774 | 0.025 |
| Ethiopia | Male | | | < 0.001 | | 0.05 | | 0.035 | | 0.18 | | < 0.001 | | 0.989 | | | 0.34 | | 0.02 | 0.004 | | 0 | | 0.01 | < 0.001 | 0.334 | < 0.001 |
| Fiji | Both | | | < 0.001 | | 1 | | 1 | | 1 | | 0 | | 1 | | | 1 | | 1 | 1 | | 1 | | 0.94 | < 0.001 | 0.88 | 0.964 |
| Fiji | Female | | | 0.059 | | 1 | | 1 | | 1 | | 0.85 | | 1 | | | 1 | | 1 | 1 | | 1 | | 0.87 | < 0.001 | 0.997 | 0.897 |
| Fiji | Male | | | < 0.001 | | 1 | | 1 | | 1 | | 0.03 | | 1 | | | 1 | | 1 | 1 | | 1 | | 0.92 | 0.002 | 0.953 | 0.965 |
| Finland | Both | | | < 0.001 | | < 0.001 | | < 0.001 | | < 0.001 | | < 0.001 | | < 0.001 | | | < 0.001 | | < 0.001 | < 0.001 | | < 0.001 | | < 0.001 | < 0.001 | < 0.001 | 0.022 |
| Finland | Female | | | < 0.001 | | < 0.001 | | < 0.001 | | < 0.001 | | < 0.001 | | < 0.001 | | | < 0.001 | | < 0.001 | < 0.001 | | < 0.001 | | < 0.001 | 0.002 | < 0.001 | 0.617 |
| Finland | Male | | | < 0.001 | | < 0.001 | | < 0.001 | | < 0.001 | | < 0.001 | | < 0.001 | | | 0.02 | | < 0.001 | < 0.001 | | < 0.001 | | < 0.001 | < 0.001 | 0.007 | < 0.001 |
| France | Both | | | < 0.001 | | < 0.001 | | < 0.001 | | < 0.001 | | < 0.001 | | < 0.001 | | | < 0.001 | | < 0.001 | < 0.001 | | < 0.001 | | < 0.001 | < 0.001 | < 0.001 | < 0.001 |
| France | Female | | | < 0.001 | | < 0.001 | | < 0.001 | | < 0.001 | | < 0.001 | | < 0.001 | | | 0.11 | | < 0.001 | < 0.001 | | < 0.001 | | < 0.001 | 0.491 | < 0.001 | < 0.001 |
| France | Male | | | < 0.001 | | < 0.001 | | < 0.001 | | < 0.001 | | < 0.001 | | < 0.001 | | | < 0.001 | | < 0.001 | < 0.001 | | < 0.001 | | < 0.001 | < 0.001 | < 0.001 | 0.037 |
| Gabon | Both | | | < 0.001 | | 1 | | 1 | | 1 | | < 0.001 | | 1 | | | 0.99 | | 1 | 0.991 | | 0.99 | | 0.56 | < 0.001 | 0.773 | 0.711 |
| Gabon | Female | | | < 0.001 | | 1 | | 1 | | 1 | | 0 | | 1 | | | 1 | | 1 | 1 | | 1 | | 0.82 | < 0.001 | 0.899 | 0.882 |
| Gabon | Male | | | < 0.001 | | 1 | | 1 | | 1 | | 0 | | 1 | | | 1 | | 1 | 0.996 | | 1 | | 0.68 | < 0.001 | 0.971 | 0.739 |
| Gambia | Both | | | < 0.001 | | 1 | | 1 | | 1 | | < 0.001 | | 1 | | | 0.99 | | 1 | 0.991 | | 0.75 | | 0.11 | < 0.001 | 0.994 | 0.76 |
| Gambia | Female | | | < 0.001 | | 1 | | 1 | | 1 | | 0.01 | | 1 | | | 1 | | 1 | 0.994 | | 0.92 | | 0.23 | < 0.001 | 0.997 | 0.689 |
| Gambia | Male | | | < 0.001 | | 1 | | 1 | | 1 | | < 0.001 | | 1 | | | 0.99 | | 1 | 0.994 | | 0.88 | | 0.2 | < 0.001 | 0.938 | 0.801 |
| Georgia | Both | | | < 0.001 | | < 0.001 | | < 0.001 | | < 0.001 | | < 0.001 | | < 0.001 | | | < 0.001 | | < 0.001 | < 0.001 | | < 0.001 | | < 0.001 | < 0.001 | < 0.001 | < 0.001 |
| Georgia | Female | | | < 0.001 | | < 0.001 | | < 0.001 | | < 0.001 | | < 0.001 | | < 0.001 | | | < 0.001 | | < 0.001 | < 0.001 | | < 0.001 | | < 0.001 | < 0.001 | < 0.001 | < 0.001 |
| Georgia | Male | | | < 0.001 | | < 0.001 | | < 0.001 | | < 0.001 | | < 0.001 | | < 0.001 | | | < 0.001 | | < 0.001 | < 0.001 | | < 0.001 | | < 0.001 | < 0.001 | < 0.001 | < 0.001 |
| Germany | Both | | | < 0.001 | | < 0.001 | | < 0.001 | | < 0.001 | | < 0.001 | | < 0.001 | | | < 0.001 | | < 0.001 | < 0.001 | | < 0.001 | | < 0.001 | < 0.001 | < 0.001 | < 0.001 |
| Germany | Female | | | < 0.001 | | < 0.001 | | < 0.001 | | < 0.001 | | < 0.001 | | < 0.001 | | | < 0.001 | | < 0.001 | < 0.001 | | < 0.001 | | < 0.001 | 0.472 | < 0.001 | < 0.001 |
| Germany | Male | | | < 0.001 | | < 0.001 | | < 0.001 | | < 0.001 | | < 0.001 | | < 0.001 | | | < 0.001 | | < 0.001 | < 0.001 | | < 0.001 | | < 0.001 | < 0.001 | < 0.001 | < 0.001 |
| Ghana | Both | | | < 0.001 | | 1 | | 0.037 | | 1 | | < 0.001 | | 1 | | | 0.39 | | 1 | 0.098 | | < 0.001 | | < 0.001 | < 0.001 | 0.679 | 0.078 |
| Ghana | Female | | | < 0.001 | | 1 | | 0.027 | | 0.92 | | < 0.001 | | 1 | | | 0.44 | | 1 | 0.48 | | < 0.001 | | < 0.001 | < 0.001 | 0.187 | 0.637 |
| Ghana | Male | | | < 0.001 | | 1 | | 0.484 | | 1 | | < 0.001 | | 1 | | | 0.76 | | 1 | 0.366 | | 0.01 | | < 0.001 | < 0.001 | 0.575 | 0.13 |
| Greece | Both | | | < 0.001 | | < 0.001 | | < 0.001 | | < 0.001 | | < 0.001 | | < 0.001 | | | < 0.001 | | < 0.001 | < 0.001 | | < 0.001 | | < 0.001 | < 0.001 | < 0.001 | 0.007 |
| Greece | Female | | | < 0.001 | | < 0.001 | | < 0.001 | | < 0.001 | | < 0.001 | | < 0.001 | | | 0.22 | | < 0.001 | 0.039 | | < 0.001 | | < 0.001 | < 0.001 | < 0.001 | 0.016 |
| Greece | Male | | | < 0.001 | | < 0.001 | | < 0.001 | | < 0.001 | | < 0.001 | | < 0.001 | | | < 0.001 | | < 0.001 | < 0.001 | | < 0.001 | | < 0.001 | < 0.001 | < 0.001 | 0.019 |
| Greenland | Both | | | 0.4 | | 1 | | 1 | | 1 | | 1 | | 1 | | | 1 | | 1 | 0.998 | | 0.74 | | 0.1 | 0.016 | 0.915 | 0.71 |
| Greenland | Female | | | 1 | | 1 | | 1 | | 1 | | 1 | | 1 | | | 1 | | 1 | 1 | | 0.95 | | 0.28 | 0.361 | 0.808 | 0.872 |
| Greenland | Male | | | 0.481 | | 1 | | 1 | | 1 | | 1 | | 1 | | | 1 | | 1 | 0.997 | | 0.89 | | 0.21 | 0.035 | 0.968 | 0.701 |
| Grenada | Both | | | < 0.001 | | 1 | | 1 | | 1 | | 0.03 | | 1 | | | 1 | | 1 | 1 | | 1 | | 0.69 | < 0.001 | 0.861 | 0.985 |
| Grenada | Female | | | 0.004 | | 1 | | 1 | | 1 | | 0.52 | | 1 | | | 1 | | 1 | 1 | | 1 | | 1 | 0.001 | 0.975 | 0.952 |
| Grenada | Male | | | < 0.001 | | 1 | | 1 | | 1 | | 0.53 | | 1 | | | 1 | | 1 | 1 | | 1 | | 0.69 | < 0.001 | 0.938 | 0.984 |
| Guam | Both | | | 0.01 | | 1 | | 1 | | 1 | | 0.93 | | 1 | | | 1 | | 1 | 0.999 | | 0.98 | | 0.4 | < 0.001 | 0.998 | 0.769 |
| Guam | Female | | | 0.991 | | 1 | | 1 | | 1 | | 1 | | 1 | | | 1 | | 1 | 1 | | 1 | | 0.57 | 0.105 | 0.984 | 0.829 |
| Guam | Male | | | 0.036 | | 1 | | 1 | | 1 | | 0.99 | | 1 | | | 1 | | 1 | 0.999 | | 0.99 | | 0.52 | < 0.001 | 0.99 | 0.759 |
| Guatemala | Both | | | < 0.001 | | 0.68 | | 0.446 | | 0.3 | | < 0.001 | | 0.77 | | | 0.72 | | 0.521 | 0.623 | | 0 | | < 0.001 | < 0.001 | 0.659 | 0.171 |
| Guatemala | Female | | | < 0.001 | | 1 | | 0.998 | | 0.95 | | < 0.001 | | 1 | | | 0.96 | | 0.986 | 0.976 | | 0.87 | | 0.3 | < 0.001 | 0.758 | 0.607 |
| Guatemala | Male | | | < 0.001 | | 0.72 | | 0.009 | | 0.27 | | < 0.001 | | 0.698 | | | 0.77 | | 0.558 | 0.689 | | < 0.001 | | < 0.001 | < 0.001 | 0.449 | 0.203 |
| Guinea | Both | | | < 0.001 | | 1 | | < 0.001 | | 1 | | < 0.001 | | 1 | | | 0.18 | | 1 | 0.196 | | < 0.001 | | < 0.001 | < 0.001 | 0.818 | 0.194 |
| Guinea | Female | | | < 0.001 | | 1 | | 0.013 | | 1 | | < 0.001 | | 1 | | | 0.53 | | 1 | 0.639 | | < 0.001 | | < 0.001 | < 0.001 | 0.886 | 0.479 |
| Guinea | Male | | | < 0.001 | | 1 | | < 0.001 | | 1 | | < 0.001 | | 1 | | | 0.5 | | 1 | 0.583 | | < 0.001 | | < 0.001 | < 0.001 | 0.653 | 0.374 |
| Guinea-Bissau | Both | | | < 0.001 | | 1 | | 1 | | 1 | | < 0.001 | | 1 | | | 0.99 | | 1 | 0.984 | | 0.87 | | 0.2 | < 0.001 | 0.819 | 0.619 |
| Guinea-Bissau | Female | | | < 0.001 | | 1 | | 1 | | 1 | | 0.04 | | 1 | | | 1 | | 1 | 0.993 | | 0.97 | | 0.38 | < 0.001 | 0.95 | 0.642 |
| Guinea-Bissau | Male | | | < 0.001 | | 1 | | 1 | | 1 | | 0 | | 1 | | | 1 | | 1 | 0.996 | | 0.95 | | 0.31 | < 0.001 | 0.93 | 0.772 |
| Guyana | Both | | | < 0.001 | | 1 | | 1 | | 1 | | < 0.001 | | 1 | | | 1 | | 1 | 0.962 | | 0.96 | | 0.59 | < 0.001 | 0.831 | 0.462 |
| Guyana | Female | | | < 0.001 | | 1 | | 1 | | 1 | | < 0.001 | | 1 | | | 1 | | 1 | 0.996 | | 0.97 | | 0.44 | < 0.001 | 0.953 | 0.674 |
| Guyana | Male | | | < 0.001 | | 1 | | 1 | | 1 | | < 0.001 | | 1 | | | 1 | | 1 | 0.989 | | 1 | | 0.98 | < 0.001 | 0.843 | 0.611 |
| Haiti | Both | | | < 0.001 | | 0.98 | | 0.042 | | 0.69 | | < 0.001 | | 0.999 | | | 0.94 | | 0.942 | 0.964 | | 0.15 | | 0.01 | < 0.001 | 0.665 | 0.667 |
| Haiti | Female | | | < 0.001 | | 1 | | 0.88 | | 0.96 | | < 0.001 | | 1 | | | 0.99 | | 1 | 0.995 | | 0.33 | | 0.02 | < 0.001 | 0.879 | 0.724 |
| Haiti | Male | | | < 0.001 | | 0.96 | | 0.638 | | 0.85 | | < 0.001 | | 1 | | | 0.92 | | 0.885 | 0.96 | | 0.78 | | 0.19 | < 0.001 | 0.302 | 0.707 |
| Honduras | Both | | | < 0.001 | | 0.56 | | 0.134 | | 0.03 | | < 0.001 | | 0.999 | | | 1 | | 0.372 | 0.999 | | 0.45 | | 0.03 | < 0.001 | 0.004 | 0.793 |
| Honduras | Female | | | < 0.001 | | 0.99 | | 0.991 | | 0.33 | | < 0.001 | | 1 | | | 1 | | 0.961 | 0.936 | | 0.98 | | 0.85 | < 0.001 | 0.074 | 0.376 |
| Honduras | Male | | | < 0.001 | | 0.99 | | 0.26 | | 0.62 | | < 0.001 | | 1 | | | 1 | | 0.948 | 0.966 | | 0.05 | | 0 | < 0.001 | 0.055 | 0.486 |
| Hungary | Both | | | < 0.001 | | < 0.001 | | < 0.001 | | < 0.001 | | < 0.001 | | < 0.001 | | | 0.05 | | < 0.001 | 0.063 | | < 0.001 | | < 0.001 | < 0.001 | 0.681 | 0.307 |
| Hungary | Female | | | < 0.001 | | < 0.001 | | < 0.001 | | 0.01 | | < 0.001 | | < 0.001 | | | 0.29 | | < 0.001 | 0.37 | | < 0.001 | | < 0.001 | < 0.001 | 0.95 | 0.437 |
| Hungary | Male | | | < 0.001 | | < 0.001 | | < 0.001 | | 0.51 | | < 0.001 | | 0.009 | | | 0.08 | | < 0.001 | 0.148 | | < 0.001 | | < 0.001 | < 0.001 | 0.151 | 0.98 |
| Iceland | Both | | | < 0.001 | | 1 | | 0.015 | | 0.99 | | 0.01 | | 1 | | | 0.99 | | 1 | 0.973 | | 0.03 | | < 0.001 | < 0.001 | 0.46 | 0.525 |
| Iceland | Female | | | < 0.001 | | 1 | | 0.467 | | 0.98 | | < 0.001 | | 1 | | | 1 | | 1 | 0.998 | | 0.38 | | 0.02 | 0.202 | 0.357 | 0.798 |
| Iceland | Male | | | < 0.001 | | 1 | | 0.302 | | 1 | | 0.13 | | 1 | | | 1 | | 1 | 0.98 | | 0.2 | | 0.01 | < 0.001 | 0.871 | 0.533 |
| India | Both | | | < 0.001 | | 0 | | 0.002 | | 0.3 | | < 0.001 | | 0.977 | | | < 0.001 | | < 0.001 | < 0.001 | | < 0.001 | | 0.45 | < 0.001 | 0.082 | < 0.001 |
| India | Female | | | < 0.001 | | < 0.001 | | < 0.001 | | 0.83 | | < 0.001 | | 0.122 | | | 0.01 | | 0.002 | < 0.001 | | < 0.001 | | 0.38 | < 0.001 | 0.445 | < 0.001 |
| India | Male | | | < 0.001 | | 0.01 | | 0.003 | | 0.34 | | < 0.001 | | 0.914 | | | < 0.001 | | 0.004 | < 0.001 | | < 0.001 | | 0.03 | < 0.001 | 0.095 | < 0.001 |
| Indonesia | Both | | | < 0.001 | | < 0.001 | | < 0.001 | | < 0.001 | | < 0.001 | | 0.012 | | | < 0.001 | | < 0.001 | < 0.001 | | < 0.001 | | < 0.001 | < 0.001 | < 0.001 | < 0.001 |
| Indonesia | Female | | | < 0.001 | | < 0.001 | | < 0.001 | | < 0.001 | | < 0.001 | | < 0.001 | | | < 0.001 | | < 0.001 | < 0.001 | | < 0.001 | | < 0.001 | < 0.001 | < 0.001 | < 0.001 |
| Indonesia | Male | | | < 0.001 | | < 0.001 | | < 0.001 | | < 0.001 | | < 0.001 | | 0.005 | | | < 0.001 | | < 0.001 | < 0.001 | | < 0.001 | | < 0.001 | < 0.001 | < 0.001 | < 0.001 |
| Iran (Islamic Republic of) | Both | | | < 0.001 | | < 0.001 | | < 0.001 | | < 0.001 | | < 0.001 | | < 0.001 | | | < 0.001 | | < 0.001 | < 0.001 | | < 0.001 | | < 0.001 | < 0.001 | < 0.001 | < 0.001 |
| Iran (Islamic Republic of) | Female | | | < 0.001 | | < 0.001 | | < 0.001 | | 0.26 | | < 0.001 | | 0.016 | | | < 0.001 | | < 0.001 | < 0.001 | | < 0.001 | | < 0.001 | < 0.001 | 0.013 | < 0.001 |
| Iran (Islamic Republic of) | Male | | | < 0.001 | | < 0.001 | | < 0.001 | | < 0.001 | | < 0.001 | | < 0.001 | | | < 0.001 | | < 0.001 | < 0.001 | | < 0.001 | | < 0.001 | < 0.001 | < 0.001 | < 0.001 |
| Iraq | Both | | | < 0.001 | | 0 | | < 0.001 | | 0 | | < 0.001 | | 0.002 | | | 0.25 | | < 0.001 | < 0.001 | | < 0.001 | | < 0.001 | < 0.001 | 0.03 | < 0.001 |
| Iraq | Female | | | < 0.001 | | 0.1 | | < 0.001 | | 0.05 | | < 0.001 | | 0.081 | | | 0.85 | | 0.043 | 0.615 | | < 0.001 | | < 0.001 | < 0.001 | 0.693 | 0.184 |
| Iraq | Male | | | < 0.001 | | 0.36 | | < 0.001 | | 0.03 | | < 0.001 | | 0.78 | | | 0.15 | | 0.198 | < 0.001 | | < 0.001 | | < 0.001 | < 0.001 | 0.003 | < 0.001 |
| Ireland | Both | | | < 0.001 | | < 0.001 | | < 0.001 | | < 0.001 | | < 0.001 | | < 0.001 | | | < 0.001 | | < 0.001 | < 0.001 | | < 0.001 | | < 0.001 | < 0.001 | < 0.001 | 0.189 |
| Ireland | Female | | | < 0.001 | | < 0.001 | | < 0.001 | | < 0.001 | | < 0.001 | | 0.002 | | | < 0.001 | | < 0.001 | < 0.001 | | < 0.001 | | < 0.001 | 0.025 | < 0.001 | 0.55 |
| Ireland | Male | | | < 0.001 | | < 0.001 | | < 0.001 | | < 0.001 | | < 0.001 | | < 0.001 | | | < 0.001 | | < 0.001 | < 0.001 | | < 0.001 | | < 0.001 | < 0.001 | < 0.001 | 0.492 |
| Israel | Both | | | < 0.001 | | < 0.001 | | < 0.001 | | < 0.001 | | < 0.001 | | < 0.001 | | | < 0.001 | | < 0.001 | < 0.001 | | < 0.001 | | < 0.001 | < 0.001 | < 0.001 | < 0.001 |
| Israel | Female | | | < 0.001 | | < 0.001 | | < 0.001 | | < 0.001 | | < 0.001 | | < 0.001 | | | < 0.001 | | < 0.001 | < 0.001 | | < 0.001 | | < 0.001 | < 0.001 | < 0.001 | < 0.001 |
| Israel | Male | | | < 0.001 | | < 0.001 | | < 0.001 | | < 0.001 | | < 0.001 | | < 0.001 | | | < 0.001 | | < 0.001 | < 0.001 | | < 0.001 | | < 0.001 | < 0.001 | < 0.001 | < 0.001 |
| Italy | Both | | | < 0.001 | | < 0.001 | | < 0.001 | | < 0.001 | | < 0.001 | | < 0.001 | | | < 0.001 | | < 0.001 | < 0.001 | | < 0.001 | | < 0.001 | < 0.001 | < 0.001 | < 0.001 |
| Italy | Female | | | < 0.001 | | < 0.001 | | < 0.001 | | < 0.001 | | < 0.001 | | < 0.001 | | | < 0.001 | | < 0.001 | < 0.001 | | < 0.001 | | < 0.001 | < 0.001 | < 0.001 | < 0.001 |
| Italy | Male | | | < 0.001 | | < 0.001 | | < 0.001 | | < 0.001 | | < 0.001 | | < 0.001 | | | < 0.001 | | < 0.001 | < 0.001 | | < 0.001 | | < 0.001 | < 0.001 | < 0.001 | < 0.001 |
| Jamaica | Both | | | < 0.001 | | 1 | | < 0.001 | | 0.87 | | < 0.001 | | 1 | | | 0.9 | | 1 | 0.811 | | < 0.001 | | < 0.001 | < 0.001 | 0.291 | 0.376 |
| Jamaica | Female | | | < 0.001 | | 1 | | 0.843 | | 0.99 | | < 0.001 | | 1 | | | 0.96 | | 1 | 0.977 | | 0.07 | | 0 | < 0.001 | 0.561 | 0.737 |
| Jamaica | Male | | | < 0.001 | | 1 | | 0.003 | | 0.98 | | < 0.001 | | 1 | | | 0.96 | | 1 | 0.923 | | < 0.001 | | < 0.001 | < 0.001 | 0.427 | 0.503 |
| Japan | Both | | | < 0.001 | | < 0.001 | | < 0.001 | | < 0.001 | | < 0.001 | | 0.012 | | | < 0.001 | | < 0.001 | < 0.001 | | < 0.001 | | < 0.001 | < 0.001 | < 0.001 | < 0.001 |
| Japan | Female | | | < 0.001 | | < 0.001 | | < 0.001 | | < 0.001 | | < 0.001 | | < 0.001 | | | < 0.001 | | < 0.001 | < 0.001 | | < 0.001 | | < 0.001 | < 0.001 | < 0.001 | < 0.001 |
| Japan | Male | | | < 0.001 | | < 0.001 | | < 0.001 | | < 0.001 | | < 0.001 | | 0.451 | | | < 0.001 | | < 0.001 | < 0.001 | | < 0.001 | | < 0.001 | < 0.001 | 0.002 | < 0.001 |
| Jordan | Both | | | < 0.001 | | 0.99 | | < 0.001 | | 0.98 | | < 0.001 | | 1 | | | 0.36 | | 0.965 | 0.263 | | < 0.001 | | < 0.001 | < 0.001 | 0.334 | 0.058 |
| Jordan | Female | | | < 0.001 | | 1 | | 0.143 | | 1 | | < 0.001 | | 1 | | | 0.67 | | 1 | 0.452 | | < 0.001 | | < 0.001 | < 0.001 | 0.709 | 0.08 |
| Jordan | Male | | | < 0.001 | | 1 | | 0.095 | | 1 | | < 0.001 | | 1 | | | 0.66 | | 0.999 | 0.497 | | < 0.001 | | < 0.001 | < 0.001 | 0.453 | 0.1 |
| Kazakhstan | Both | | | < 0.001 | | < 0.001 | | < 0.001 | | < 0.001 | | < 0.001 | | < 0.001 | | | < 0.001 | | < 0.001 | < 0.001 | | < 0.001 | | < 0.001 | < 0.001 | 0.773 | < 0.001 |
| Kazakhstan | Female | | | < 0.001 | | < 0.001 | | < 0.001 | | < 0.001 | | < 0.001 | | < 0.001 | | | 0.01 | | < 0.001 | < 0.001 | | < 0.001 | | < 0.001 | < 0.001 | 0.903 | < 0.001 |
| Kazakhstan | Male | | | < 0.001 | | < 0.001 | | < 0.001 | | < 0.001 | | < 0.001 | | < 0.001 | | | < 0.001 | | < 0.001 | < 0.001 | | < 0.001 | | < 0.001 | < 0.001 | 0.014 | < 0.001 |
| Kenya | Both | | | < 0.001 | | 0.89 | | 0.917 | | 0.44 | | < 0.001 | | 0.981 | | | 0.94 | | 0.766 | 0.152 | | 0.23 | | 0.96 | < 0.001 | 0.125 | 0.012 |
| Kenya | Female | | | < 0.001 | | 1 | | 1 | | 1 | | < 0.001 | | 1 | | | 0.96 | | 1 | 0.418 | | 0.35 | | 0.42 | < 0.001 | 0.922 | 0.051 |
| Kenya | Male | | | < 0.001 | | 1 | | 0.998 | | 1 | | < 0.001 | | 1 | | | 0.96 | | 1 | 0.449 | | 0.22 | | 0.03 | < 0.001 | 0.945 | 0.074 |
| Kiribati | Both | | | 0.507 | | 1 | | 1 | | 1 | | 1 | | 1 | | | 1 | | 1 | 1 | | 1 | | 0.9 | 0.087 | 0.987 | 0.949 |
| Kiribati | Female | | | 1 | | 1 | | 1 | | 1 | | 1 | | 1 | | | 1 | | 1 | 1 | | 1 | | 0.9 | 0.343 | 0.991 | 0.967 |
| Kiribati | Male | | | 0.769 | | 1 | | 1 | | 1 | | 1 | | 1 | | | 1 | | 1 | 1 | | 1 | | 0.93 | 0.234 | 0.995 | 0.946 |
| Kuwait | Both | | | < 0.001 | | 1 | | 1 | | 1 | | < 0.001 | | 1 | | | 0.88 | | 1 | 0.235 | | 0.26 | | 0.79 | < 0.001 | 0.499 | 0.026 |
| Kuwait | Female | | | < 0.001 | | 1 | | 1 | | 1 | | < 0.001 | | 1 | | | 0.98 | | 1 | 0.873 | | 0.94 | | 0.69 | < 0.001 | 0.698 | 0.316 |
| Kuwait | Male | | | < 0.001 | | 1 | | 1 | | 1 | | < 0.001 | | 1 | | | 0.93 | | 1 | 0.53 | | 0.65 | | 0.41 | < 0.001 | 0.833 | 0.092 |
| Kyrgyzstan | Both | | | < 0.001 | | < 0.001 | | < 0.001 | | < 0.001 | | < 0.001 | | < 0.001 | | | 0.14 | | < 0.001 | 0.022 | | 0.03 | | 0.19 | < 0.001 | < 0.001 | 0.024 |
| Kyrgyzstan | Female | | | < 0.001 | | < 0.001 | | < 0.001 | | < 0.001 | | < 0.001 | | < 0.001 | | | 0.15 | | < 0.001 | 0.149 | | 0.22 | | 0.48 | < 0.001 | < 0.001 | 0.27 |
| Kyrgyzstan | Male | | | < 0.001 | | < 0.001 | | < 0.001 | | < 0.001 | | < 0.001 | | 0.705 | | | 0.66 | | < 0.001 | 0.368 | | 0.5 | | 0.65 | < 0.001 | < 0.001 | 0.129 |
| Lao People's Democratic Republic | Both | | | < 0.001 | | 1 | | 0.442 | | 0.74 | | < 0.001 | | 0.999 | | | 0.98 | | 0.995 | 0.979 | | 0.23 | | 0.01 | < 0.001 | 0.289 | 0.582 |
| Lao People's Democratic Republic | Female | | | < 0.001 | | 1 | | 0.999 | | 0.91 | | 0.07 | | 1 | | | 1 | | 1 | 0.998 | | 0.81 | | 0.14 | < 0.001 | 0.339 | 0.791 |
| Lao People's Democratic Republic | Male | | | < 0.001 | | 1 | | 0.685 | | 0.99 | | < 0.001 | | 1 | | | 0.98 | | 1 | 0.981 | | 0.47 | | 0.04 | < 0.001 | 0.636 | 0.585 |
| Latvia | Both | | | < 0.001 | | < 0.001 | | < 0.001 | | < 0.001 | | < 0.001 | | 0.999 | | | 0.01 | | < 0.001 | 0.013 | | < 0.001 | | < 0.001 | < 0.001 | 0.019 | 0.334 |
| Latvia | Female | | | < 0.001 | | 0.29 | | < 0.001 | | 0.15 | | < 0.001 | | 0.998 | | | 0.03 | | 0.161 | < 0.001 | | < 0.001 | | < 0.001 | 0.008 | 0.153 | < 0.001 |
| Latvia | Male | | | < 0.001 | | < 0.001 | | < 0.001 | | < 0.001 | | < 0.001 | | 0.936 | | | 0.09 | | < 0.001 | 0.009 | | < 0.001 | | < 0.001 | < 0.001 | 0.004 | 0.009 |
| Lebanon | Both | | | < 0.001 | | 0.7 | | 0.309 | | 0.92 | | < 0.001 | | 0.906 | | | 0 | | 0.554 | < 0.001 | | < 0.001 | | 0.54 | < 0.001 | 0.315 | < 0.001 |
| Lebanon | Female | | | < 0.001 | | 0.62 | | 0.449 | | 0.99 | | < 0.001 | | 0.97 | | | 0.8 | | 0.433 | 0.742 | | 0.78 | | 0.61 | < 0.001 | 0.355 | 0.263 |
| Lebanon | Male | | | < 0.001 | | 1 | | 1 | | 1 | | < 0.001 | | 1 | | | 0 | | 1 | < 0.001 | | < 0.001 | | 0.13 | < 0.001 | 0.766 | < 0.001 |
| Lesotho | Both | | | < 0.001 | | 1 | | 0.993 | | 1 | | < 0.001 | | 1 | | | 1 | | 1 | 0.839 | | 0.19 | | 0.01 | < 0.001 | 0.929 | 0.24 |
| Lesotho | Female | | | < 0.001 | | 1 | | 1 | | 1 | | 0 | | 1 | | | 1 | | 1 | 0.756 | | 0.64 | | 0.17 | < 0.001 | 0.897 | 0.177 |
| Lesotho | Male | | | < 0.001 | | 1 | | 0.995 | | 1 | | < 0.001 | | 1 | | | 1 | | 1 | 0.99 | | 0.51 | | 0.04 | < 0.001 | 0.956 | 0.602 |
| Liberia | Both | | | < 0.001 | | 1 | | 0.998 | | 1 | | < 0.001 | | 1 | | | 0.92 | | 1 | 0.97 | | 0.27 | | 0.02 | < 0.001 | 0.634 | 0.802 |
| Liberia | Female | | | < 0.001 | | 1 | | 1 | | 1 | | < 0.001 | | 1 | | | 0.99 | | 1 | 0.997 | | 0.82 | | 0.15 | < 0.001 | 0.882 | 0.952 |
| Liberia | Male | | | < 0.001 | | 1 | | 1 | | 1 | | < 0.001 | | 1 | | | 0.95 | | 1 | 0.982 | | 0.52 | | 0.06 | < 0.001 | 0.774 | 0.751 |
| Libya | Both | | | < 0.001 | | 1 | | 0.61 | | 1 | | < 0.001 | | 1 | | | 0.02 | | 1 | 0.018 | | < 0.001 | | < 0.001 | < 0.001 | 0.874 | 0.059 |
| Libya | Female | | | < 0.001 | | 1 | | 0.953 | | 1 | | < 0.001 | | 1 | | | 0.37 | | 1 | 0.417 | | 0.11 | | 0.02 | < 0.001 | 0.79 | 0.235 |
| Libya | Male | | | < 0.001 | | 1 | | 0.31 | | 1 | | < 0.001 | | 1 | | | 0.1 | | 0.996 | 0.079 | | 0 | | < 0.001 | < 0.001 | 0.577 | 0.058 |
| Lithuania | Both | | | < 0.001 | | 0 | | < 0.001 | | 0.26 | | < 0.001 | | 0.31 | | | 0.48 | | < 0.001 | 0.116 | | < 0.001 | | < 0.001 | < 0.001 | 0.331 | 0.023 |
| Lithuania | Female | | | < 0.001 | | < 0.001 | | < 0.001 | | 0.93 | | < 0.001 | | 0.002 | | | 0.61 | | < 0.001 | < 0.001 | | < 0.001 | | < 0.001 | 0.527 | 0.34 | < 0.001 |
| Lithuania | Male | | | < 0.001 | | < 0.001 | | < 0.001 | | < 0.001 | | < 0.001 | | 0.316 | | | 0.12 | | < 0.001 | 0.041 | | < 0.001 | | < 0.001 | < 0.001 | 0.08 | 0.048 |
| Luxembourg | Both | | | < 0.001 | | 0.82 | | < 0.001 | | 0.54 | | < 0.001 | | 0.983 | | | 1 | | 0.673 | 0.004 | | < 0.001 | | < 0.001 | < 0.001 | 0.308 | < 0.001 |
| Luxembourg | Female | | | < 0.001 | | 1 | | 0.003 | | 0.84 | | < 0.001 | | 1 | | | 0.98 | | 0.992 | 0.146 | | 0.03 | | 0.01 | 0.372 | 0.228 | 0.01 |
| Luxembourg | Male | | | < 0.001 | | 1 | | 0.08 | | 0.98 | | 0.06 | | 1 | | | 0.98 | | 0.994 | 0.068 | | 0.01 | | 0 | < 0.001 | 0.707 | 0.004 |
| Madagascar | Both | | | < 0.001 | | 0.98 | | 0.036 | | 0.83 | | < 0.001 | | 1 | | | 0.93 | | 0.929 | 0.025 | | < 0.001 | | < 0.001 | < 0.001 | 0.218 | 0.002 |
| Madagascar | Female | | | < 0.001 | | 1 | | 0.877 | | 1 | | < 0.001 | | 1 | | | 0.99 | | 1 | 0.541 | | 0.03 | | < 0.001 | < 0.001 | 0.869 | 0.102 |
| Madagascar | Male | | | < 0.001 | | 1 | | 0.23 | | 0.87 | | < 0.001 | | 1 | | | 0.95 | | 0.999 | 0.062 | | < 0.001 | | < 0.001 | < 0.001 | 0.269 | 0.005 |
| Malawi | Both | | | < 0.001 | | 1 | | 1 | | 0.92 | | < 0.001 | | 1 | | | 0.77 | | 1 | 0.172 | | 0.12 | | 0.21 | < 0.001 | 0.312 | 0.016 |
| Malawi | Female | | | < 0.001 | | 1 | | 1 | | 1 | | < 0.001 | | 1 | | | 0.95 | | 1 | 0.83 | | 0.81 | | 0.46 | < 0.001 | 0.88 | 0.252 |
| Malawi | Male | | | < 0.001 | | 1 | | 1 | | 1 | | < 0.001 | | 1 | | | 0.95 | | 1 | 0.425 | | 0.57 | | 0.83 | < 0.001 | 0.849 | 0.054 |
| Malaysia | Both | | | < 0.001 | | < 0.001 | | < 0.001 | | < 0.001 | | < 0.001 | | < 0.001 | | | 0.15 | | < 0.001 | < 0.001 | | < 0.001 | | 0.01 | < 0.001 | < 0.001 | < 0.001 |
| Malaysia | Female | | | < 0.001 | | < 0.001 | | < 0.001 | | 0 | | < 0.001 | | < 0.001 | | | 0.3 | | < 0.001 | < 0.001 | | < 0.001 | | 0.31 | < 0.001 | 0.012 | < 0.001 |
| Malaysia | Male | | | < 0.001 | | < 0.001 | | < 0.001 | | 0.01 | | < 0.001 | | < 0.001 | | | 0.4 | | < 0.001 | < 0.001 | | < 0.001 | | < 0.001 | < 0.001 | < 0.001 | < 0.001 |
| Maldives | Both | | | < 0.001 | | 1 | | 1 | | 1 | | 0.99 | | 1 | | | 1 | | 1 | 0.993 | | 0.97 | | 0.37 | < 0.001 | 0.829 | 0.72 |
| Maldives | Female | | | 0.989 | | 1 | | 1 | | 1 | | 1 | | 1 | | | 1 | | 1 | 1 | | 1 | | 0.67 | 0.084 | 0.997 | 0.935 |
| Maldives | Male | | | < 0.001 | | 1 | | 1 | | 1 | | 1 | | 1 | | | 1 | | 1 | 0.999 | | 0.98 | | 0.42 | < 0.001 | 0.991 | 0.847 |
| Mali | Both | | | < 0.001 | | 1 | | 0.956 | | 1 | | < 0.001 | | 1 | | | 0.85 | | 0.999 | 0.735 | | 0.04 | | 0 | < 0.001 | 0.975 | 0.23 |
| Mali | Female | | | < 0.001 | | 1 | | 1 | | 1 | | < 0.001 | | 1 | | | 0.98 | | 1 | 0.99 | | 0.78 | | 0.16 | < 0.001 | 0.977 | 0.737 |
| Mali | Male | | | < 0.001 | | 1 | | 1 | | 1 | | < 0.001 | | 1 | | | 0.89 | | 1 | 0.678 | | 0.27 | | 0.08 | < 0.001 | 0.688 | 0.163 |
| Malta | Both | | | < 0.001 | | 1 | | 0.042 | | 1 | | < 0.001 | | 1 | | | 0.7 | | 1 | < 0.001 | | < 0.001 | | 0 | < 0.001 | 0.619 | < 0.001 |
| Malta | Female | | | < 0.001 | | 1 | | 0.61 | | 1 | | < 0.001 | | 1 | | | 0.85 | | 1 | 0.029 | | 0.02 | | 0.04 | 0.609 | 0.607 | 0.002 |
| Malta | Male | | | < 0.001 | | 1 | | 0.263 | | 1 | | 0.02 | | 1 | | | 0.85 | | 1 | 0.003 | | 0 | | 0.01 | < 0.001 | 0.801 | < 0.001 |
| Marshall Islands | Both | | | 0.977 | | 1 | | 1 | | 1 | | 1 | | 1 | | | 1 | | 1 | 1 | | 1 | | 0.95 | 0.162 | 0.984 | 0.956 |
| Marshall Islands | Female | | | 1 | | 1 | | 1 | | 1 | | 1 | | 1 | | | 1 | | 1 | 1 | | 1 | | 0.97 | 0.486 | 1 | 0.983 |
| Marshall Islands | Male | | | 0.988 | | 1 | | 1 | | 1 | | 1 | | 1 | | | 1 | | 1 | 1 | | 1 | | 0.99 | 0.264 | 0.997 | 0.969 |
| Mauritania | Both | | | < 0.001 | | 1 | | 1 | | 1 | | < 0.001 | | 1 | | | 0.99 | | 1 | 0.981 | | 0.98 | | 0.55 | < 0.001 | 0.967 | 0.548 |
| Mauritania | Female | | | < 0.001 | | 1 | | 1 | | 1 | | < 0.001 | | 1 | | | 0.99 | | 1 | 0.982 | | 1 | | 0.92 | < 0.001 | 0.912 | 0.55 |
| Mauritania | Male | | | < 0.001 | | 1 | | 1 | | 1 | | < 0.001 | | 1 | | | 1 | | 1 | 0.999 | | 1 | | 0.71 | < 0.001 | 0.795 | 0.799 |
| Mauritius | Both | | | < 0.001 | | 1 | | 0.002 | | 1 | | 0.01 | | 1 | | | 0.74 | | 0.996 | 0.801 | | < 0.001 | | < 0.001 | < 0.001 | 0.507 | 0.433 |
| Mauritius | Female | | | < 0.001 | | 1 | | 0.855 | | 1 | | 0.99 | | 1 | | | 0.85 | | 1 | 0.881 | | 0.19 | | 0.02 | < 0.001 | 0.595 | 0.46 |
| Mauritius | Male | | | < 0.001 | | 1 | | 0.052 | | 1 | | 0.18 | | 1 | | | 0.91 | | 1 | 0.942 | | 0.03 | | < 0.001 | < 0.001 | 0.665 | 0.559 |
| Mexico | Both | | | < 0.001 | | < 0.001 | | < 0.001 | | < 0.001 | | < 0.001 | | 0.026 | | | 0.02 | | < 0.001 | < 0.001 | | < 0.001 | | < 0.001 | < 0.001 | < 0.001 | < 0.001 |
| Mexico | Female | | | < 0.001 | | < 0.001 | | < 0.001 | | < 0.001 | | < 0.001 | | 0.007 | | | 0 | | < 0.001 | < 0.001 | | < 0.001 | | < 0.001 | < 0.001 | < 0.001 | 0.007 |
| Mexico | Male | | | < 0.001 | | 0 | | < 0.001 | | < 0.001 | | < 0.001 | | 0.385 | | | 0.02 | | 0.001 | < 0.001 | | < 0.001 | | < 0.001 | < 0.001 | < 0.001 | < 0.001 |
| Micronesia (Federated States of) | Both | | | 0.44 | | 1 | | 1 | | 1 | | 1 | | 1 | | | 1 | | 1 | 1 | | 1 | | 0.73 | 0.028 | 0.985 | 0.914 |
| Micronesia (Federated States of) | Female | | | 1 | | 1 | | 1 | | 1 | | 1 | | 1 | | | 1 | | 1 | 1 | | 1 | | 0.86 | 0.271 | 0.994 | 0.967 |
| Micronesia (Federated States of) | Male | | | 0.684 | | 1 | | 1 | | 1 | | 1 | | 1 | | | 1 | | 1 | 1 | | 1 | | 0.83 | 0.096 | 1 | 0.965 |
| Monaco | Both | | | 0.071 | | 1 | | 0.995 | | 1 | | 1 | | 1 | | | 0.99 | | 1 | 0.979 | | 0.88 | | 0.23 | 0.065 | 0.848 | 0.576 |
| Monaco | Female | | | 0.783 | | 1 | | 1 | | 1 | | 0.94 | | 1 | | | 1 | | 1 | 0.995 | | 0.97 | | 0.41 | 0.761 | 0.799 | 0.694 |
| Monaco | Male | | | 0.341 | | 1 | | 1 | | 1 | | 1 | | 1 | | | 1 | | 1 | 0.991 | | 0.96 | | 0.35 | 0.082 | 0.932 | 0.647 |
| Mongolia | Both | | | < 0.001 | | 0.43 | | < 0.001 | | 0.84 | | < 0.001 | | 0.999 | | | 0.05 | | 0.266 | < 0.001 | | < 0.001 | | 0.01 | < 0.001 | 0.052 | < 0.001 |
| Mongolia | Female | | | < 0.001 | | 0.97 | | 0.276 | | 0.98 | | < 0.001 | | 0.999 | | | 0.36 | | 0.903 | 0.026 | | 0.02 | | 0.27 | < 0.001 | 0.225 | 0.003 |
| Mongolia | Male | | | < 0.001 | | 0.44 | | 0.033 | | 0.81 | | < 0.001 | | 1 | | | 0.23 | | 0.267 | < 0.001 | | < 0.001 | | 0.09 | < 0.001 | 0.147 | < 0.001 |
| Montenegro | Both | | | < 0.001 | | 1 | | 1 | | 0.99 | | < 0.001 | | 1 | | | 1 | | 1 | 0.998 | | 0.87 | | 0.2 | < 0.001 | 0.543 | 0.728 |
| Montenegro | Female | | | < 0.001 | | 1 | | 1 | | 0.99 | | < 0.001 | | 1 | | | 1 | | 1 | 1 | | 1 | | 0.58 | < 0.001 | 0.625 | 0.846 |
| Montenegro | Male | | | < 0.001 | | 1 | | 1 | | 1 | | < 0.001 | | 1 | | | 1 | | 1 | 0.999 | | 0.85 | | 0.18 | < 0.001 | 0.847 | 0.797 |
| Morocco | Both | | | < 0.001 | | 0.17 | | < 0.001 | | 0.02 | | < 0.001 | | 0.521 | | | 0.01 | | 0.092 | < 0.001 | | < 0.001 | | < 0.001 | < 0.001 | 0.102 | 0.004 |
| Morocco | Female | | | < 0.001 | | < 0.001 | | < 0.001 | | < 0.001 | | < 0.001 | | < 0.001 | | | 0.83 | | < 0.001 | 0.351 | | < 0.001 | | < 0.001 | < 0.001 | 0.001 | 0.071 |
| Morocco | Male | | | < 0.001 | | 0.85 | | < 0.001 | | 0.99 | | < 0.001 | | 0.812 | | | < 0.001 | | 0.773 | < 0.001 | | < 0.001 | | < 0.001 | < 0.001 | 0.863 | < 0.001 |
| Mozambique | Both | | | < 0.001 | | 1 | | 0.976 | | 1 | | < 0.001 | | 1 | | | 0.93 | | 1 | 0.864 | | 0.29 | | 0.02 | < 0.001 | 0.581 | 0.425 |
| Mozambique | Female | | | < 0.001 | | 1 | | 1 | | 1 | | < 0.001 | | 1 | | | 1 | | 1 | 0.998 | | 0.92 | | 0.25 | < 0.001 | 0.737 | 0.87 |
| Mozambique | Male | | | < 0.001 | | 1 | | 0.525 | | 1 | | < 0.001 | | 1 | | | 0.9 | | 1 | 0.894 | | 0.12 | | 0 | < 0.001 | 0.925 | 0.547 |
| Myanmar | Both | | | < 0.001 | | < 0.001 | | < 0.001 | | < 0.001 | | < 0.001 | | < 0.001 | | | 0.05 | | < 0.001 | 0.069 | | < 0.001 | | < 0.001 | < 0.001 | 0.732 | 0.579 |
| Myanmar | Female | | | < 0.001 | | < 0.001 | | < 0.001 | | < 0.001 | | < 0.001 | | < 0.001 | | | < 0.001 | | < 0.001 | 0.003 | | < 0.001 | | < 0.001 | < 0.001 | 0.072 | 0.508 |
| Myanmar | Male | | | < 0.001 | | 0.32 | | < 0.001 | | 0.02 | | < 0.001 | | 0.412 | | | 0.98 | | 0.171 | 0.296 | | < 0.001 | | < 0.001 | < 0.001 | 0.083 | 0.031 |
| Namibia | Both | | | < 0.001 | | 1 | | 1 | | 1 | | < 0.001 | | 1 | | | 0.99 | | 1 | 0.939 | | 0.51 | | 0.09 | < 0.001 | 0.973 | 0.395 |
| Namibia | Female | | | < 0.001 | | 1 | | 1 | | 1 | | 0 | | 1 | | | 1 | | 1 | 0.902 | | 0.54 | | 0.13 | < 0.001 | 0.895 | 0.322 |
| Namibia | Male | | | < 0.001 | | 1 | | 1 | | 1 | | < 0.001 | | 1 | | | 1 | | 1 | 0.999 | | 0.99 | | 0.51 | < 0.001 | 0.972 | 0.854 |
| Nauru | Both | | | 1 | | 1 | | 1 | | 1 | | 1 | | 1 | | | 1 | | 1 | 1 | | 1 | | 0.99 | 0.606 | 0.999 | 0.948 |
| Nauru | Female | | | 1 | | 1 | | 1 | | 1 | | 1 | | 1 | | | 1 | | 1 | 1 | | 1 | | 0.99 | 0.797 | 0.999 | 0.984 |
| Nauru | Male | | | 1 | | 1 | | 1 | | 1 | | 1 | | 1 | | | 1 | | 1 | 1 | | 1 | | 0.99 | 0.681 | 0.998 | 0.972 |
| Nepal | Both | | | < 0.001 | | 0.1 | | < 0.001 | | 0.1 | | < 0.001 | | 1 | | | 0.55 | | 0.041 | 0.04 | | < 0.001 | | < 0.001 | < 0.001 | < 0.001 | 0.01 |
| Nepal | Female | | | < 0.001 | | 1 | | < 0.001 | | 1 | | < 0.001 | | 1 | | | 0.84 | | 1 | 0.009 | | < 0.001 | | < 0.001 | < 0.001 | 0.495 | < 0.001 |
| Nepal | Male | | | < 0.001 | | 0.27 | | 0.133 | | 0.22 | | < 0.001 | | 1 | | | 0.82 | | 0.14 | 0.911 | | 0.9 | | 0.42 | < 0.001 | 0.005 | 0.689 |
| Netherlands | Both | | | < 0.001 | | < 0.001 | | < 0.001 | | < 0.001 | | < 0.001 | | < 0.001 | | | < 0.001 | | < 0.001 | < 0.001 | | < 0.001 | | < 0.001 | < 0.001 | < 0.001 | 0.011 |
| Netherlands | Female | | | < 0.001 | | < 0.001 | | < 0.001 | | < 0.001 | | < 0.001 | | < 0.001 | | | < 0.001 | | < 0.001 | < 0.001 | | < 0.001 | | < 0.001 | < 0.001 | < 0.001 | 0.072 |
| Netherlands | Male | | | < 0.001 | | < 0.001 | | < 0.001 | | < 0.001 | | < 0.001 | | < 0.001 | | | < 0.001 | | < 0.001 | < 0.001 | | < 0.001 | | < 0.001 | < 0.001 | < 0.001 | 0.003 |
| New Zealand | Both | | | < 0.001 | | < 0.001 | | < 0.001 | | < 0.001 | | < 0.001 | | < 0.001 | | | 0.34 | | < 0.001 | 0.203 | | < 0.001 | | < 0.001 | < 0.001 | < 0.001 | 0.115 |
| New Zealand | Female | | | < 0.001 | | 0.03 | | < 0.001 | | 0.35 | | < 0.001 | | 0.124 | | | 0.71 | | 0.02 | 0.327 | | < 0.001 | | < 0.001 | < 0.001 | 0.01 | 0.072 |
| New Zealand | Male | | | < 0.001 | | < 0.001 | | < 0.001 | | < 0.001 | | < 0.001 | | < 0.001 | | | 0.5 | | < 0.001 | 0.09 | | < 0.001 | | < 0.001 | < 0.001 | < 0.001 | 0.019 |
| Nicaragua | Both | | | < 0.001 | | 0.21 | | 0.16 | | 0.01 | | < 0.001 | | 0.744 | | | 0.99 | | 0.099 | 0.601 | | 0.59 | | 0.18 | < 0.001 | 0.011 | 0.116 |
| Nicaragua | Female | | | < 0.001 | | 0.77 | | 0.807 | | 0.06 | | < 0.001 | | 0.993 | | | 0.98 | | 0.59 | 0.832 | | 0.88 | | 0.84 | < 0.001 | 0.035 | 0.247 |
| Nicaragua | Male | | | < 0.001 | | 1 | | 0.958 | | 0.74 | | < 0.001 | | 1 | | | 1 | | 0.991 | 0.929 | | 0.54 | | 0.05 | < 0.001 | 0.189 | 0.4 |
| Niger | Both | | | < 0.001 | | 1 | | 1 | | 0.99 | | < 0.001 | | 1 | | | 0.95 | | 1 | 0.916 | | 0.45 | | 0.08 | < 0.001 | 0.95 | 0.459 |
| Niger | Female | | | < 0.001 | | 1 | | 1 | | 1 | | < 0.001 | | 1 | | | 0.97 | | 1 | 0.99 | | 0.7 | | 0.12 | < 0.001 | 0.907 | 0.845 |
| Niger | Male | | | < 0.001 | | 1 | | 1 | | 1 | | < 0.001 | | 1 | | | 0.98 | | 1 | 0.953 | | 0.75 | | 0.2 | < 0.001 | 0.741 | 0.483 |
| Nigeria | Both | | | < 0.001 | | < 0.001 | | < 0.001 | | < 0.001 | | < 0.001 | | < 0.001 | | | < 0.001 | | < 0.001 | < 0.001 | | < 0.001 | | < 0.001 | < 0.001 | 0.135 | < 0.001 |
| Nigeria | Female | | | < 0.001 | | < 0.001 | | < 0.001 | | 0.35 | | < 0.001 | | 0.021 | | | 0 | | < 0.001 | < 0.001 | | < 0.001 | | < 0.001 | < 0.001 | 0.003 | < 0.001 |
| Nigeria | Male | | | < 0.001 | | 0 | | < 0.001 | | 0.18 | | < 0.001 | | 0.967 | | | 0.11 | | < 0.001 | < 0.001 | | < 0.001 | | < 0.001 | < 0.001 | 0.004 | < 0.001 |
| Niue | Both | | | 1 | | 1 | | 1 | | 1 | | 1 | | 1 | | | 1 | | 1 | 1 | | 1 | | 0.99 | 0.667 | 0.999 | 0.984 |
| Niue | Female | | | 1 | | 1 | | 1 | | 1 | | 1 | | 1 | | | 1 | | 1 | 1 | | 1 | | 1 | 0.848 | 0.999 | 0.996 |
| Niue | Male | | | 1 | | 1 | | 1 | | 1 | | 1 | | 1 | | | 1 | | 1 | 1 | | 1 | | 1 | 0.699 | 0.999 | 0.991 |
| North Macedonia | Both | | | < 0.001 | | 0.81 | | < 0.001 | | 0.39 | | < 0.001 | | 0.798 | | | 0.86 | | 0.675 | 0.685 | | 0.07 | | 0.01 | < 0.001 | 0.507 | 0.202 |
| North Macedonia | Female | | | < 0.001 | | 0.95 | | 0.852 | | 0.59 | | < 0.001 | | 0.986 | | | 0.99 | | 0.877 | 0.997 | | 0.93 | | 0.28 | < 0.001 | 0.407 | 0.809 |
| North Macedonia | Male | | | < 0.001 | | 1 | | < 0.001 | | 0.88 | | < 0.001 | | 0.997 | | | 0.8 | | 0.994 | 0.48 | | 0.03 | | 0.01 | < 0.001 | 0.836 | 0.102 |
| Northern Mariana Islands | Both | | | 0.835 | | 1 | | 1 | | 1 | | 1 | | 1 | | | 1 | | 1 | 1 | | 1 | | 0.93 | 0.082 | 0.987 | 0.963 |
| Northern Mariana Islands | Female | | | 1 | | 1 | | 1 | | 1 | | 1 | | 1 | | | 1 | | 1 | 1 | | 1 | | 0.88 | 0.436 | 0.995 | 0.966 |
| Northern Mariana Islands | Male | | | 0.93 | | 1 | | 1 | | 1 | | 1 | | 1 | | | 1 | | 1 | 1 | | 1 | | 0.88 | 0.151 | 0.995 | 0.944 |
| Norway | Both | | | < 0.001 | | < 0.001 | | < 0.001 | | < 0.001 | | < 0.001 | | 0.087 | | | < 0.001 | | < 0.001 | < 0.001 | | < 0.001 | | < 0.001 | < 0.001 | < 0.001 | < 0.001 |
| Norway | Female | | | < 0.001 | | < 0.001 | | < 0.001 | | < 0.001 | | < 0.001 | | 0.534 | | | 0.03 | | < 0.001 | 0.004 | | < 0.001 | | < 0.001 | < 0.001 | < 0.001 | 0.007 |
| Norway | Male | | | < 0.001 | | < 0.001 | | < 0.001 | | 0.01 | | < 0.001 | | 0.214 | | | 0.01 | | < 0.001 | < 0.001 | | < 0.001 | | < 0.001 | < 0.001 | < 0.001 | < 0.001 |
| Oman | Both | | | < 0.001 | | 0.68 | | 0.018 | | 0.76 | | < 0.001 | | 0.715 | | | 0.77 | | 0.554 | 0.82 | | 0 | | < 0.001 | < 0.001 | 0.772 | 0.651 |
| Oman | Female | | | < 0.001 | | 1 | | 0.999 | | 1 | | < 0.001 | | 1 | | | 0.84 | | 1 | 0.907 | | 0.37 | | 0.03 | < 0.001 | 0.964 | 0.754 |
| Oman | Male | | | < 0.001 | | 0.98 | | 0.754 | | 0.92 | | < 0.001 | | 0.981 | | | 0.75 | | 0.95 | 0.739 | | 0.33 | | 0.04 | < 0.001 | 0.991 | 0.466 |
| Pakistan | Both | | | < 0.001 | | < 0.001 | | < 0.001 | | 0.23 | | < 0.001 | | 0.029 | | | 0.27 | | < 0.001 | < 0.001 | | < 0.001 | | < 0.001 | < 0.001 | 0.567 | < 0.001 |
| Pakistan | Female | | | < 0.001 | | 1 | | < 0.001 | | 0.82 | | < 0.001 | | 0.999 | | | 0.04 | | 0.994 | < 0.001 | | < 0.001 | | < 0.001 | < 0.001 | 0.125 | < 0.001 |
| Pakistan | Male | | | < 0.001 | | 0.12 | | < 0.001 | | 0.2 | | < 0.001 | | 0.408 | | | 0.38 | | 0.052 | < 0.001 | | < 0.001 | | < 0.001 | < 0.001 | 0.808 | < 0.001 |
| Palau | Both | | | 0.999 | | 1 | | 1 | | 1 | | 1 | | 1 | | | 1 | | 1 | 1 | | 1 | | 0.97 | 0.26 | 0.978 | 0.94 |
| Palau | Female | | | 1 | | 1 | | 1 | | 1 | | 1 | | 1 | | | 1 | | 1 | 1 | | 1 | | 0.98 | 0.613 | 1 | 0.995 |
| Palau | Male | | | 0.999 | | 1 | | 1 | | 1 | | 1 | | 1 | | | 1 | | 1 | 1 | | 1 | | 0.99 | 0.36 | 0.997 | 0.981 |
| Palestine | Both | | | < 0.001 | | 1 | | 0.997 | | 1 | | < 0.001 | | 0.999 | | | 0.99 | | 0.978 | 0.98 | | 0.99 | | 0.61 | < 0.001 | 0.672 | 0.549 |
| Palestine | Female | | | < 0.001 | | 1 | | 1 | | 1 | | < 0.001 | | 1 | | | 1 | | 1 | 1 | | 1 | | 0.89 | < 0.001 | 0.823 | 0.935 |
| Palestine | Male | | | < 0.001 | | 1 | | 1 | | 1 | | < 0.001 | | 1 | | | 0.99 | | 1 | 0.978 | | 0.99 | | 0.76 | < 0.001 | 0.58 | 0.54 |
| Panama | Both | | | < 0.001 | | 1 | | 1 | | 1 | | < 0.001 | | 1 | | | 1 | | 1 | 0.979 | | 0.99 | | 0.84 | < 0.001 | 0.917 | 0.58 |
| Panama | Female | | | < 0.001 | | 1 | | 1 | | 1 | | < 0.001 | | 1 | | | 1 | | 1 | 0.997 | | 0.89 | | 0.27 | < 0.001 | 0.876 | 0.712 |
| Panama | Male | | | < 0.001 | | 1 | | 1 | | 1 | | < 0.001 | | 1 | | | 0.99 | | 1 | 0.985 | | 0.96 | | 0.37 | < 0.001 | 0.906 | 0.666 |
| Papua New Guinea | Both | | | < 0.001 | | 1 | | 0.982 | | 1 | | < 0.001 | | 1 | | | 0.93 | | 1 | 0.587 | | 0.33 | | 0.05 | < 0.001 | 0.811 | 0.174 |
| Papua New Guinea | Female | | | < 0.001 | | 1 | | 1 | | 1 | | < 0.001 | | 1 | | | 0.99 | | 1 | 0.958 | | 0.92 | | 0.3 | < 0.001 | 0.831 | 0.52 |
| Papua New Guinea | Male | | | < 0.001 | | 1 | | 1 | | 1 | | < 0.001 | | 1 | | | 0.97 | | 1 | 0.728 | | 0.68 | | 0.21 | < 0.001 | 0.978 | 0.232 |
| Paraguay | Both | | | < 0.001 | | 1 | | 1 | | 1 | | < 0.001 | | 1 | | | 0.99 | | 1 | 0.647 | | 0.77 | | 0.87 | < 0.001 | 0.895 | 0.149 |
| Paraguay | Female | | | < 0.001 | | 1 | | 1 | | 1 | | < 0.001 | | 1 | | | 1 | | 1 | 0.947 | | 0.98 | | 1 | < 0.001 | 0.949 | 0.437 |
| Paraguay | Male | | | < 0.001 | | 1 | | 1 | | 1 | | < 0.001 | | 1 | | | 0.99 | | 1 | 0.829 | | 0.91 | | 0.85 | < 0.001 | 0.862 | 0.271 |
| Peru | Both | | | < 0.001 | | < 0.001 | | < 0.001 | | < 0.001 | | < 0.001 | | < 0.001 | | | 0.03 | | < 0.001 | < 0.001 | | < 0.001 | | < 0.001 | < 0.001 | < 0.001 | < 0.001 |
| Peru | Female | | | < 0.001 | | < 0.001 | | < 0.001 | | 0 | | < 0.001 | | 0.301 | | | 0.5 | | < 0.001 | 0.113 | | 0 | | < 0.001 | < 0.001 | 0.006 | 0.024 |
| Peru | Male | | | < 0.001 | | < 0.001 | | < 0.001 | | < 0.001 | | < 0.001 | | < 0.001 | | | 0.08 | | < 0.001 | < 0.001 | | < 0.001 | | < 0.001 | < 0.001 | < 0.001 | < 0.001 |
| Philippines | Both | | | < 0.001 | | < 0.001 | | < 0.001 | | < 0.001 | | < 0.001 | | 0.301 | | | < 0.001 | | < 0.001 | < 0.001 | | < 0.001 | | < 0.001 | < 0.001 | < 0.001 | < 0.001 |
| Philippines | Female | | | < 0.001 | | 0 | | < 0.001 | | < 0.001 | | < 0.001 | | 0.01 | | | < 0.001 | | < 0.001 | < 0.001 | | < 0.001 | | < 0.001 | < 0.001 | 0.021 | < 0.001 |
| Philippines | Male | | | < 0.001 | | 0.04 | | < 0.001 | | 0.01 | | < 0.001 | | 0.999 | | | < 0.001 | | 0.015 | < 0.001 | | < 0.001 | | < 0.001 | < 0.001 | 0.008 | < 0.001 |
| Poland | Both | | | < 0.001 | | < 0.001 | | < 0.001 | | < 0.001 | | < 0.001 | | < 0.001 | | | < 0.001 | | < 0.001 | < 0.001 | | < 0.001 | | < 0.001 | < 0.001 | < 0.001 | < 0.001 |
| Poland | Female | | | < 0.001 | | < 0.001 | | < 0.001 | | < 0.001 | | < 0.001 | | < 0.001 | | | < 0.001 | | < 0.001 | < 0.001 | | < 0.001 | | < 0.001 | < 0.001 | < 0.001 | < 0.001 |
| Poland | Male | | | < 0.001 | | < 0.001 | | < 0.001 | | < 0.001 | | < 0.001 | | < 0.001 | | | < 0.001 | | < 0.001 | < 0.001 | | < 0.001 | | < 0.001 | < 0.001 | < 0.001 | < 0.001 |
| Portugal | Both | | | < 0.001 | | < 0.001 | | < 0.001 | | < 0.001 | | < 0.001 | | < 0.001 | | | < 0.001 | | < 0.001 | < 0.001 | | < 0.001 | | < 0.001 | < 0.001 | < 0.001 | 0.183 |
| Portugal | Female | | | < 0.001 | | < 0.001 | | < 0.001 | | < 0.001 | | < 0.001 | | < 0.001 | | | < 0.001 | | < 0.001 | < 0.001 | | < 0.001 | | < 0.001 | 0.51 | < 0.001 | 0.623 |
| Portugal | Male | | | < 0.001 | | < 0.001 | | < 0.001 | | < 0.001 | | < 0.001 | | < 0.001 | | | < 0.001 | | < 0.001 | < 0.001 | | < 0.001 | | < 0.001 | < 0.001 | < 0.001 | 0.208 |
| Puerto Rico | Both | | | < 0.001 | | < 0.001 | | < 0.001 | | < 0.001 | | < 0.001 | | 0.003 | | | 0.85 | | < 0.001 | 0.589 | | 0.19 | | 0.06 | < 0.001 | < 0.001 | 0.15 |
| Puerto Rico | Female | | | < 0.001 | | 0.01 | | < 0.001 | | < 0.001 | | < 0.001 | | 0.182 | | | 0.96 | | 0.004 | 0.891 | | 0.25 | | 0.03 | < 0.001 | 0.068 | 0.347 |
| Puerto Rico | Male | | | < 0.001 | | 0 | | 0.003 | | < 0.001 | | < 0.001 | | 0.603 | | | 0.9 | | 0.001 | 0.705 | | 0.75 | | 0.59 | < 0.001 | 0.009 | 0.204 |
| Qatar | Both | | | < 0.001 | | 1 | | 1 | | 1 | | < 0.001 | | 1 | | | 0.98 | | 1 | 0.996 | | 0.99 | | 0.52 | < 0.001 | 0.799 | 0.953 |
| Qatar | Female | | | < 0.001 | | 1 | | 1 | | 1 | | 0.31 | | 1 | | | 1 | | 1 | 1 | | 0.99 | | 0.47 | 0.007 | 0.808 | 0.942 |
| Qatar | Male | | | < 0.001 | | 1 | | 1 | | 1 | | < 0.001 | | 1 | | | 0.96 | | 1 | 0.98 | | 0.96 | | 0.43 | < 0.001 | 0.925 | 0.981 |
| Republic of Korea | Both | | | < 0.001 | | < 0.001 | | < 0.001 | | < 0.001 | | < 0.001 | | < 0.001 | | | < 0.001 | | < 0.001 | < 0.001 | | < 0.001 | | < 0.001 | < 0.001 | < 0.001 | < 0.001 |
| Republic of Korea | Female | | | < 0.001 | | < 0.001 | | < 0.001 | | < 0.001 | | < 0.001 | | < 0.001 | | | < 0.001 | | < 0.001 | < 0.001 | | < 0.001 | | < 0.001 | < 0.001 | < 0.001 | < 0.001 |
| Republic of Korea | Male | | | < 0.001 | | < 0.001 | | < 0.001 | | < 0.001 | | < 0.001 | | 0.271 | | | < 0.001 | | < 0.001 | < 0.001 | | < 0.001 | | < 0.001 | < 0.001 | < 0.001 | < 0.001 |
| Republic of Moldova | Both | | | < 0.001 | | < 0.001 | | < 0.001 | | < 0.001 | | < 0.001 | | 0.104 | | | 0.73 | | < 0.001 | < 0.001 | | < 0.001 | | 0 | < 0.001 | < 0.001 | < 0.001 |
| Republic of Moldova | Female | | | < 0.001 | | < 0.001 | | < 0.001 | | < 0.001 | | < 0.001 | | 0.012 | | | 0.8 | | < 0.001 | < 0.001 | | < 0.001 | | < 0.001 | < 0.001 | < 0.001 | < 0.001 |
| Republic of Moldova | Male | | | < 0.001 | | < 0.001 | | < 0.001 | | < 0.001 | | < 0.001 | | 0.276 | | | 0.67 | | < 0.001 | < 0.001 | | < 0.001 | | 0 | < 0.001 | < 0.001 | < 0.001 |
| Romania | Both | | | < 0.001 | | < 0.001 | | < 0.001 | | < 0.001 | | < 0.001 | | < 0.001 | | | < 0.001 | | < 0.001 | < 0.001 | | < 0.001 | | < 0.001 | < 0.001 | < 0.001 | < 0.001 |
| Romania | Female | | | < 0.001 | | < 0.001 | | < 0.001 | | < 0.001 | | < 0.001 | | < 0.001 | | | < 0.001 | | < 0.001 | < 0.001 | | < 0.001 | | < 0.001 | < 0.001 | < 0.001 | 0.001 |
| Romania | Male | | | < 0.001 | | < 0.001 | | < 0.001 | | < 0.001 | | < 0.001 | | < 0.001 | | | < 0.001 | | < 0.001 | < 0.001 | | < 0.001 | | < 0.001 | < 0.001 | < 0.001 | < 0.001 |
| Russian Federation | Both | | | < 0.001 | | < 0.001 | | < 0.001 | | < 0.001 | | < 0.001 | | < 0.001 | | | < 0.001 | | < 0.001 | < 0.001 | | < 0.001 | | < 0.001 | < 0.001 | < 0.001 | < 0.001 |
| Russian Federation | Female | | | < 0.001 | | < 0.001 | | < 0.001 | | < 0.001 | | < 0.001 | | < 0.001 | | | < 0.001 | | < 0.001 | < 0.001 | | < 0.001 | | < 0.001 | < 0.001 | < 0.001 | < 0.001 |
| Russian Federation | Male | | | < 0.001 | | < 0.001 | | < 0.001 | | < 0.001 | | < 0.001 | | < 0.001 | | | < 0.001 | | < 0.001 | < 0.001 | | < 0.001 | | < 0.001 | < 0.001 | < 0.001 | < 0.001 |
| Rwanda | Both | | | < 0.001 | | 1 | | 0.612 | | 1 | | < 0.001 | | 1 | | | 0.57 | | 1 | 0.68 | | 0.06 | | 0 | < 0.001 | 0.708 | 0.807 |
| Rwanda | Female | | | < 0.001 | | 1 | | 1 | | 1 | | < 0.001 | | 1 | | | 0.81 | | 1 | 0.912 | | 0.47 | | 0.05 | < 0.001 | 0.958 | 0.966 |
| Rwanda | Male | | | < 0.001 | | 1 | | 0.998 | | 1 | | < 0.001 | | 1 | | | 0.69 | | 1 | 0.834 | | 0.47 | | 0.07 | < 0.001 | 0.876 | 0.86 |
| Saint Kitts and Nevis | Both | | | < 0.001 | | 1 | | 1 | | 1 | | 0.58 | | 1 | | | 1 | | 1 | 1 | | 1 | | 0.89 | < 0.001 | 0.951 | 0.936 |
| Saint Kitts and Nevis | Female | | | 0.335 | | 1 | | 1 | | 1 | | 0.95 | | 1 | | | 1 | | 1 | 1 | | 1 | | 0.93 | 0.032 | 0.982 | 0.975 |
| Saint Kitts and Nevis | Male | | | 0.05 | | 1 | | 1 | | 1 | | 0.95 | | 1 | | | 1 | | 1 | 1 | | 1 | | 0.88 | 0.036 | 0.973 | 0.955 |
| Saint Lucia | Both | | | < 0.001 | | 1 | | 1 | | 1 | | < 0.001 | | 1 | | | 1 | | 1 | 1 | | 1 | | 0.97 | < 0.001 | 0.947 | 0.864 |
| Saint Lucia | Female | | | < 0.001 | | 1 | | 1 | | 1 | | 0.07 | | 1 | | | 1 | | 1 | 1 | | 1 | | 0.73 | < 0.001 | 0.975 | 0.93 |
| Saint Lucia | Male | | | < 0.001 | | 1 | | 1 | | 1 | | 0.08 | | 1 | | | 1 | | 1 | 1 | | 1 | | 0.93 | < 0.001 | 0.952 | 0.913 |
| Saint Vincent and the Grenadines | Both | | | < 0.001 | | 1 | | 1 | | 1 | | 0.02 | | 1 | | | 1 | | 1 | 1 | | 1 | | 0.85 | < 0.001 | 0.986 | 0.92 |
| Saint Vincent and the Grenadines | Female | | | 0.002 | | 1 | | 1 | | 1 | | 0.45 | | 1 | | | 1 | | 1 | 1 | | 1 | | 0.85 | 0.002 | 0.982 | 0.932 |
| Saint Vincent and the Grenadines | Male | | | < 0.001 | | 1 | | 1 | | 1 | | 0.36 | | 1 | | | 1 | | 1 | 1 | | 1 | | 0.86 | < 0.001 | 0.965 | 0.955 |
| Samoa | Both | | | 0.015 | | 1 | | 1 | | 1 | | 0.92 | | 1 | | | 1 | | 1 | 1 | | 1 | | 0.84 | < 0.001 | 0.996 | 0.967 |
| Samoa | Female | | | 0.993 | | 1 | | 1 | | 1 | | 1 | | 1 | | | 1 | | 1 | 1 | | 1 | | 0.91 | 0.077 | 0.992 | 0.977 |
| Samoa | Male | | | 0.047 | | 1 | | 1 | | 1 | | 0.99 | | 1 | | | 1 | | 1 | 1 | | 1 | | 0.85 | < 0.001 | 0.997 | 0.949 |
| San Marino | Both | | | 0.422 | | 1 | | 1 | | 1 | | 1 | | 1 | | | 1 | | 1 | 0.99 | | 0.88 | | 0.2 | 0.048 | 0.878 | 0.652 |
| San Marino | Female | | | 0.968 | | 1 | | 1 | | 1 | | 0.98 | | 1 | | | 1 | | 1 | 0.998 | | 0.98 | | 0.43 | 0.735 | 0.794 | 0.767 |
| San Marino | Male | | | 0.664 | | 1 | | 1 | | 1 | | 1 | | 1 | | | 1 | | 1 | 0.996 | | 0.97 | | 0.37 | 0.067 | 0.94 | 0.719 |
| Sao Tome and Principe | Both | | | 0.059 | | 1 | | 1 | | 1 | | 0.95 | | 1 | | | 1 | | 1 | 1 | | 1 | | 0.55 | < 0.001 | 0.981 | 0.859 |
| Sao Tome and Principe | Female | | | 0.83 | | 1 | | 1 | | 1 | | 1 | | 1 | | | 1 | | 1 | 1 | | 1 | | 0.78 | 0.025 | 0.977 | 0.866 |
| Sao Tome and Principe | Male | | | 0.55 | | 1 | | 1 | | 1 | | 1 | | 1 | | | 1 | | 1 | 1 | | 1 | | 0.7 | 0.01 | 0.982 | 0.915 |
| Saudi Arabia | Both | | | < 0.001 | | < 0.001 | | < 0.001 | | < 0.001 | | < 0.001 | | < 0.001 | | | < 0.001 | | < 0.001 | < 0.001 | | < 0.001 | | < 0.001 | < 0.001 | < 0.001 | < 0.001 |
| Saudi Arabia | Female | | | < 0.001 | | 0.02 | | < 0.001 | | 0.09 | | < 0.001 | | 0.573 | | | 0.81 | | 0.006 | < 0.001 | | < 0.001 | | < 0.001 | < 0.001 | 0.014 | < 0.001 |
| Saudi Arabia | Male | | | < 0.001 | | < 0.001 | | < 0.001 | | < 0.001 | | < 0.001 | | < 0.001 | | | < 0.001 | | < 0.001 | < 0.001 | | < 0.001 | | < 0.001 | < 0.001 | < 0.001 | < 0.001 |
| Senegal | Both | | | < 0.001 | | 1 | | 1 | | 1 | | < 0.001 | | 1 | | | 0.99 | | 0.999 | 0.909 | | 0.93 | | 0.72 | < 0.001 | 0.634 | 0.338 |
| Senegal | Female | | | < 0.001 | | 1 | | 1 | | 1 | | < 0.001 | | 1 | | | 1 | | 1 | 0.97 | | 0.99 | | 0.9 | < 0.001 | 0.803 | 0.485 |
| Senegal | Male | | | < 0.001 | | 1 | | 1 | | 1 | | < 0.001 | | 1 | | | 1 | | 1 | 0.958 | | 0.97 | | 0.76 | < 0.001 | 0.552 | 0.437 |
| Serbia | Both | | | < 0.001 | | < 0.001 | | < 0.001 | | 0.49 | | < 0.001 | | < 0.001 | | | < 0.001 | | < 0.001 | < 0.001 | | < 0.001 | | < 0.001 | < 0.001 | 0.004 | < 0.001 |
| Serbia | Female | | | < 0.001 | | < 0.001 | | < 0.001 | | 0.97 | | < 0.001 | | < 0.001 | | | 0.01 | | < 0.001 | < 0.001 | | < 0.001 | | 0.41 | < 0.001 | 0.119 | 0.002 |
| Serbia | Male | | | < 0.001 | | < 0.001 | | < 0.001 | | < 0.001 | | < 0.001 | | < 0.001 | | | < 0.001 | | < 0.001 | < 0.001 | | < 0.001 | | < 0.001 | < 0.001 | < 0.001 | < 0.001 |
| Seychelles | Both | | | 0.197 | | 1 | | 1 | | 1 | | 1 | | 1 | | | 1 | | 1 | 1 | | 1 | | 0.8 | 0.002 | 0.996 | 0.911 |
| Seychelles | Female | | | 1 | | 1 | | 1 | | 1 | | 1 | | 1 | | | 1 | | 1 | 1 | | 1 | | 0.84 | 0.231 | 0.999 | 0.903 |
| Seychelles | Male | | | 0.34 | | 1 | | 1 | | 1 | | 1 | | 1 | | | 1 | | 1 | 1 | | 1 | | 0.73 | 0.014 | 0.998 | 0.905 |
| Sierra Leone | Both | | | < 0.001 | | 1 | | 1 | | 1 | | < 0.001 | | 1 | | | 1 | | 1 | 0.918 | | 0.91 | | 0.36 | < 0.001 | 0.536 | 0.355 |
| Sierra Leone | Female | | | < 0.001 | | 1 | | 1 | | 1 | | < 0.001 | | 1 | | | 1 | | 1 | 0.906 | | 0.94 | | 0.53 | < 0.001 | 0.908 | 0.319 |
| Sierra Leone | Male | | | < 0.001 | | 1 | | 1 | | 1 | | < 0.001 | | 1 | | | 1 | | 1 | 0.969 | | 0.99 | | 0.64 | < 0.001 | 0.575 | 0.485 |
| Singapore | Both | | | < 0.001 | | 0.13 | | < 0.001 | | 1 | | < 0.001 | | 0.245 | | | < 0.001 | | 0.076 | < 0.001 | | < 0.001 | | < 0.001 | < 0.001 | 0.678 | 0.343 |
| Singapore | Female | | | < 0.001 | | 0.91 | | < 0.001 | | 0.1 | | < 0.001 | | 0.907 | | | 0 | | 0.823 | 0.002 | | < 0.001 | | < 0.001 | < 0.001 | 0.816 | 0.219 |
| Singapore | Male | | | < 0.001 | | 0.8 | | < 0.001 | | 1 | | < 0.001 | | 0.855 | | | 0.03 | | 0.67 | 0.024 | | < 0.001 | | < 0.001 | < 0.001 | 0.76 | 0.044 |
| Slovakia | Both | | | < 0.001 | | < 0.001 | | < 0.001 | | 0.34 | | < 0.001 | | < 0.001 | | | 0.42 | | < 0.001 | < 0.001 | | < 0.001 | | < 0.001 | < 0.001 | 0.572 | < 0.001 |
| Slovakia | Female | | | < 0.001 | | < 0.001 | | < 0.001 | | 0.06 | | < 0.001 | | < 0.001 | | | 0.31 | | < 0.001 | < 0.001 | | < 0.001 | | < 0.001 | < 0.001 | 0.973 | < 0.001 |
| Slovakia | Male | | | < 0.001 | | < 0.001 | | < 0.001 | | 0.82 | | < 0.001 | | < 0.001 | | | 0.23 | | < 0.001 | 0.165 | | < 0.001 | | < 0.001 | < 0.001 | 0.049 | 0.136 |
| Slovenia | Both | | | < 0.001 | | 0.23 | | < 0.001 | | 0.08 | | < 0.001 | | 0.731 | | | < 0.001 | | 0.133 | < 0.001 | | < 0.001 | | < 0.001 | < 0.001 | 0.305 | < 0.001 |
| Slovenia | Female | | | < 0.001 | | 0.99 | | < 0.001 | | 0.55 | | < 0.001 | | 1 | | | < 0.001 | | 0.953 | < 0.001 | | < 0.001 | | < 0.001 | < 0.001 | 0.506 | < 0.001 |
| Slovenia | Male | | | < 0.001 | | 0.92 | | < 0.001 | | 0.33 | | < 0.001 | | 1 | | | < 0.001 | | 0.818 | < 0.001 | | < 0.001 | | < 0.001 | < 0.001 | 0.165 | 0.011 |
| Solomon Islands | Both | | | < 0.001 | | 1 | | 1 | | 1 | | 0.4 | | 1 | | | 1 | | 1 | 1 | | 1 | | 0.85 | < 0.001 | 0.977 | 0.964 |
| Solomon Islands | Female | | | 0.729 | | 1 | | 1 | | 1 | | 1 | | 1 | | | 1 | | 1 | 1 | | 1 | | 0.97 | 0.133 | 0.99 | 0.993 |
| Solomon Islands | Male | | | < 0.001 | | 1 | | 1 | | 1 | | 0.67 | | 1 | | | 1 | | 1 | 1 | | 1 | | 0.99 | 0.004 | 0.994 | 0.953 |
| Somalia | Both | | | < 0.001 | | 1 | | 0.992 | | 1 | | < 0.001 | | 1 | | | 1 | | 1 | 0.648 | | 0.33 | | 0.04 | < 0.001 | 0.543 | 0.128 |
| Somalia | Female | | | < 0.001 | | 1 | | 1 | | 1 | | < 0.001 | | 1 | | | 1 | | 1 | 0.888 | | 0.65 | | 0.09 | < 0.001 | 0.912 | 0.297 |
| Somalia | Male | | | < 0.001 | | 1 | | 0.994 | | 1 | | < 0.001 | | 1 | | | 1 | | 1 | 0.765 | | 0.37 | | 0.04 | < 0.001 | 0.896 | 0.194 |
| South Africa | Both | | | < 0.001 | | 0.73 | | < 0.001 | | 0.63 | | < 0.001 | | 0.707 | | | < 0.001 | | 0.561 | < 0.001 | | < 0.001 | | < 0.001 | < 0.001 | 0.863 | 0.002 |
| South Africa | Female | | | < 0.001 | | 0.83 | | < 0.001 | | 0.06 | | < 0.001 | | 0.994 | | | 0 | | 0.666 | < 0.001 | | < 0.001 | | < 0.001 | < 0.001 | 0.011 | < 0.001 |
| South Africa | Male | | | < 0.001 | | 1 | | < 0.001 | | 0.93 | | < 0.001 | | 1 | | | < 0.001 | | 1 | < 0.001 | | < 0.001 | | < 0.001 | < 0.001 | 0.886 | 0.008 |
| South Sudan | Both | | | < 0.001 | | 1 | | 0.752 | | 1 | | < 0.001 | | 1 | | | 1 | | 1 | 0.737 | | 0.03 | | < 0.001 | < 0.001 | 0.695 | 0.159 |
| South Sudan | Female | | | < 0.001 | | 1 | | 1 | | 1 | | < 0.001 | | 1 | | | 1 | | 1 | 0.949 | | 0.38 | | 0.03 | < 0.001 | 0.946 | 0.397 |
| South Sudan | Male | | | < 0.001 | | 1 | | 0.784 | | 1 | | < 0.001 | | 1 | | | 1 | | 1 | 0.909 | | 0.05 | | < 0.001 | < 0.001 | 0.811 | 0.318 |
| Spain | Both | | | < 0.001 | | < 0.001 | | < 0.001 | | < 0.001 | | < 0.001 | | < 0.001 | | | < 0.001 | | < 0.001 | < 0.001 | | < 0.001 | | < 0.001 | < 0.001 | < 0.001 | < 0.001 |
| Spain | Female | | | < 0.001 | | < 0.001 | | < 0.001 | | < 0.001 | | < 0.001 | | < 0.001 | | | 0.1 | | < 0.001 | < 0.001 | | < 0.001 | | < 0.001 | 0.351 | < 0.001 | < 0.001 |
| Spain | Male | | | < 0.001 | | < 0.001 | | < 0.001 | | < 0.001 | | < 0.001 | | < 0.001 | | | < 0.001 | | < 0.001 | < 0.001 | | < 0.001 | | < 0.001 | < 0.001 | < 0.001 | 0.004 |
| Sri Lanka | Both | | | < 0.001 | | < 0.001 | | < 0.001 | | 0.07 | | < 0.001 | | < 0.001 | | | 0.24 | | < 0.001 | 0.002 | | < 0.001 | | < 0.001 | < 0.001 | 0.02 | < 0.001 |
| Sri Lanka | Female | | | < 0.001 | | 0.62 | | 0.002 | | 0.47 | | < 0.001 | | 0.557 | | | 0.32 | | 0.422 | 0.265 | | 0 | | 0 | < 0.001 | 0.696 | 0.319 |
| Sri Lanka | Male | | | < 0.001 | | 0 | | < 0.001 | | 0.33 | | < 0.001 | | 0.708 | | | 0.62 | | < 0.001 | < 0.001 | | < 0.001 | | < 0.001 | < 0.001 | 0.007 | < 0.001 |
| Sudan | Both | | | < 0.001 | | < 0.001 | | < 0.001 | | < 0.001 | | < 0.001 | | < 0.001 | | | 0.01 | | < 0.001 | 0.017 | | < 0.001 | | < 0.001 | < 0.001 | < 0.001 | 0.457 |
| Sudan | Female | | | < 0.001 | | 0.04 | | < 0.001 | | 0.03 | | < 0.001 | | 0.04 | | | 0.32 | | 0.013 | 0.223 | | < 0.001 | | < 0.001 | < 0.001 | 0.142 | 0.198 |
| Sudan | Male | | | < 0.001 | | < 0.001 | | < 0.001 | | < 0.001 | | < 0.001 | | 0.03 | | | 0.01 | | < 0.001 | 0.018 | | < 0.001 | | < 0.001 | < 0.001 | < 0.001 | 0.139 |
| Suriname | Both | | | < 0.001 | | 1 | | 0.999 | | 1 | | < 0.001 | | 1 | | | 1 | | 1 | 0.893 | | 0.39 | | 0.03 | < 0.001 | 0.748 | 0.33 |
| Suriname | Female | | | < 0.001 | | 1 | | 1 | | 1 | | < 0.001 | | 1 | | | 1 | | 1 | 0.982 | | 0.94 | | 0.29 | < 0.001 | 0.892 | 0.56 |
| Suriname | Male | | | < 0.001 | | 1 | | 1 | | 1 | | < 0.001 | | 1 | | | 1 | | 1 | 0.958 | | 0.52 | | 0.04 | < 0.001 | 0.828 | 0.458 |
| Sweden | Both | | | < 0.001 | | < 0.001 | | < 0.001 | | < 0.001 | | < 0.001 | | < 0.001 | | | < 0.001 | | < 0.001 | < 0.001 | | < 0.001 | | < 0.001 | < 0.001 | < 0.001 | 0.156 |
| Sweden | Female | | | < 0.001 | | < 0.001 | | < 0.001 | | < 0.001 | | < 0.001 | | < 0.001 | | | < 0.001 | | < 0.001 | < 0.001 | | < 0.001 | | < 0.001 | 0.28 | < 0.001 | 0.014 |
| Sweden | Male | | | < 0.001 | | < 0.001 | | < 0.001 | | < 0.001 | | < 0.001 | | < 0.001 | | | < 0.001 | | < 0.001 | < 0.001 | | < 0.001 | | < 0.001 | < 0.001 | < 0.001 | 0.201 |
| Switzerland | Both | | | < 0.001 | | < 0.001 | | < 0.001 | | < 0.001 | | < 0.001 | | < 0.001 | | | 0.33 | | < 0.001 | < 0.001 | | < 0.001 | | < 0.001 | < 0.001 | < 0.001 | < 0.001 |
| Switzerland | Female | | | < 0.001 | | < 0.001 | | < 0.001 | | < 0.001 | | < 0.001 | | < 0.001 | | | 0.13 | | < 0.001 | < 0.001 | | < 0.001 | | < 0.001 | 0.036 | < 0.001 | < 0.001 |
| Switzerland | Male | | | < 0.001 | | < 0.001 | | < 0.001 | | < 0.001 | | < 0.001 | | < 0.001 | | | 0.27 | | < 0.001 | < 0.001 | | < 0.001 | | < 0.001 | < 0.001 | < 0.001 | < 0.001 |
| Syrian Arab Republic | Both | | | < 0.001 | | < 0.001 | | < 0.001 | | < 0.001 | | < 0.001 | | < 0.001 | | | < 0.001 | | < 0.001 | < 0.001 | | < 0.001 | | 0.19 | < 0.001 | 0.302 | 0.005 |
| Syrian Arab Republic | Female | | | < 0.001 | | < 0.001 | | < 0.001 | | < 0.001 | | < 0.001 | | < 0.001 | | | 0.04 | | < 0.001 | 0.045 | | 0.04 | | 0.11 | < 0.001 | 0.07 | 0.091 |
| Syrian Arab Republic | Male | | | < 0.001 | | < 0.001 | | < 0.001 | | < 0.001 | | < 0.001 | | < 0.001 | | | < 0.001 | | < 0.001 | < 0.001 | | < 0.001 | | 0.17 | < 0.001 | 0.69 | 0.081 |
| Taiwan (Province of China) | Both | | | < 0.001 | | < 0.001 | | < 0.001 | | < 0.001 | | < 0.001 | | < 0.001 | | | 0.09 | | < 0.001 | < 0.001 | | < 0.001 | | < 0.001 | < 0.001 | < 0.001 | < 0.001 |
| Taiwan (Province of China) | Female | | | < 0.001 | | < 0.001 | | < 0.001 | | < 0.001 | | < 0.001 | | 0.012 | | | 0.41 | | < 0.001 | 0.14 | | 0.07 | | 0.17 | < 0.001 | 0.002 | 0.128 |
| Taiwan (Province of China) | Male | | | < 0.001 | | < 0.001 | | < 0.001 | | < 0.001 | | < 0.001 | | < 0.001 | | | 0.22 | | < 0.001 | < 0.001 | | < 0.001 | | < 0.001 | < 0.001 | < 0.001 | < 0.001 |
| Tajikistan | Both | | | < 0.001 | | < 0.001 | | < 0.001 | | < 0.001 | | < 0.001 | | < 0.001 | | | 0.93 | | < 0.001 | 0.061 | | < 0.001 | | < 0.001 | < 0.001 | < 0.001 | 0.005 |
| Tajikistan | Female | | | < 0.001 | | < 0.001 | | < 0.001 | | < 0.001 | | < 0.001 | | 0.086 | | | 0.71 | | < 0.001 | 0.656 | | < 0.001 | | < 0.001 | < 0.001 | < 0.001 | 0.343 |
| Tajikistan | Male | | | < 0.001 | | < 0.001 | | < 0.001 | | < 0.001 | | < 0.001 | | < 0.001 | | | 0.84 | | < 0.001 | 0.002 | | < 0.001 | | < 0.001 | < 0.001 | < 0.001 | < 0.001 |
| Thailand | Both | | | < 0.001 | | < 0.001 | | < 0.001 | | < 0.001 | | < 0.001 | | < 0.001 | | | 0 | | < 0.001 | < 0.001 | | < 0.001 | | < 0.001 | < 0.001 | < 0.001 | < 0.001 |
| Thailand | Female | | | < 0.001 | | < 0.001 | | < 0.001 | | < 0.001 | | < 0.001 | | < 0.001 | | | 0.05 | | < 0.001 | < 0.001 | | < 0.001 | | < 0.001 | < 0.001 | < 0.001 | < 0.001 |
| Thailand | Male | | | < 0.001 | | < 0.001 | | < 0.001 | | < 0.001 | | < 0.001 | | < 0.001 | | | < 0.001 | | < 0.001 | < 0.001 | | < 0.001 | | < 0.001 | < 0.001 | < 0.001 | 0.01 |
| Timor-Leste | Both | | | < 0.001 | | 1 | | 1 | | 1 | | 0.43 | | 1 | | | 1 | | 1 | 0.999 | | 1 | | 0.87 | < 0.001 | 0.919 | 0.762 |
| Timor-Leste | Female | | | 0.449 | | 1 | | 1 | | 1 | | 1 | | 1 | | | 1 | | 1 | 1 | | 1 | | 0.92 | 0.004 | 0.929 | 0.911 |
| Timor-Leste | Male | | | < 0.001 | | 1 | | 1 | | 1 | | 0.75 | | 1 | | | 1 | | 1 | 0.999 | | 1 | | 0.88 | < 0.001 | 0.919 | 0.753 |
| Togo | Both | | | < 0.001 | | 1 | | 0.998 | | 1 | | < 0.001 | | 1 | | | 1 | | 1 | 0.778 | | 0.5 | | 0.06 | < 0.001 | 0.507 | 0.212 |
| Togo | Female | | | < 0.001 | | 1 | | 1 | | 1 | | < 0.001 | | 1 | | | 1 | | 1 | 0.939 | | 0.8 | | 0.16 | < 0.001 | 0.945 | 0.39 |
| Togo | Male | | | < 0.001 | | 1 | | 1 | | 1 | | < 0.001 | | 1 | | | 0.99 | | 1 | 0.912 | | 0.57 | | 0.07 | < 0.001 | 0.821 | 0.387 |
| Tokelau | Both | | | 1 | | 1 | | 1 | | 1 | | 1 | | 1 | | | 1 | | 1 | 1 | | 1 | | 0.99 | 0.705 | 0.997 | 0.997 |
| Tokelau | Female | | | 1 | | 1 | | 1 | | 1 | | 1 | | 1 | | | 1 | | 1 | 1 | | 1 | | 1 | 0.866 | 0.999 | 0.996 |
| Tokelau | Male | | | 1 | | 1 | | 1 | | 1 | | 1 | | 1 | | | 1 | | 1 | 1 | | 1 | | 1 | 0.722 | 1 | 0.996 |
| Tonga | Both | | | 0.332 | | 1 | | 1 | | 1 | | 1 | | 1 | | | 1 | | 1 | 1 | | 1 | | 0.94 | 0.001 | 0.976 | 0.987 |
| Tonga | Female | | | 0.999 | | 1 | | 1 | | 1 | | 1 | | 1 | | | 1 | | 1 | 1 | | 1 | | 0.98 | 0.15 | 0.998 | 0.991 |
| Tonga | Male | | | 0.57 | | 1 | | 1 | | 1 | | 1 | | 1 | | | 1 | | 1 | 1 | | 1 | | 0.95 | 0.01 | 0.997 | 0.982 |
| Trinidad and Tobago | Both | | | < 0.001 | | 1 | | 0.979 | | 0.98 | | < 0.001 | | 1 | | | 0.93 | | 1 | 0.922 | | 0.41 | | 0.06 | < 0.001 | 0.53 | 0.442 |
| Trinidad and Tobago | Female | | | < 0.001 | | 1 | | 0.995 | | 1 | | < 0.001 | | 1 | | | 0.97 | | 1 | 0.987 | | 0.45 | | 0.04 | < 0.001 | 0.77 | 0.714 |
| Trinidad and Tobago | Male | | | < 0.001 | | 1 | | 1 | | 0.99 | | < 0.001 | | 1 | | | 0.97 | | 1 | 0.967 | | 0.9 | | 0.35 | < 0.001 | 0.577 | 0.531 |
| Tunisia | Both | | | < 0.001 | | 0 | | < 0.001 | | 0.91 | | < 0.001 | | 0.162 | | | 0.16 | | < 0.001 | 0.008 | | 0 | | 0.17 | < 0.001 | 0.514 | < 0.001 |
| Tunisia | Female | | | < 0.001 | | 1 | | 0.83 | | 0.99 | | < 0.001 | | 0.998 | | | 0.76 | | 0.983 | 0.632 | | 0.63 | | 0.5 | < 0.001 | 0.939 | 0.167 |
| Tunisia | Male | | | < 0.001 | | 0.11 | | < 0.001 | | 0.98 | | < 0.001 | | 0.883 | | | 0.21 | | 0.046 | 0.004 | | 0.01 | | 0.54 | < 0.001 | 0.471 | < 0.001 |
| Turkey | Both | | | < 0.001 | | < 0.001 | | < 0.001 | | < 0.001 | | < 0.001 | | < 0.001 | | | 0.04 | | < 0.001 | < 0.001 | | < 0.001 | | < 0.001 | < 0.001 | 0.585 | 0.002 |
| Turkey | Female | | | < 0.001 | | < 0.001 | | < 0.001 | | 0 | | < 0.001 | | < 0.001 | | | 0.43 | | < 0.001 | 0.065 | | < 0.001 | | < 0.001 | < 0.001 | 0.632 | 0.026 |
| Turkey | Male | | | < 0.001 | | < 0.001 | | < 0.001 | | < 0.001 | | < 0.001 | | < 0.001 | | | 0 | | < 0.001 | < 0.001 | | < 0.001 | | < 0.001 | < 0.001 | 0.965 | 0.001 |
| Turkmenistan | Both | | | < 0.001 | | 0.06 | | < 0.001 | | 0.02 | | < 0.001 | | 0.998 | | | < 0.001 | | 0.025 | < 0.001 | | < 0.001 | | 0 | < 0.001 | < 0.001 | < 0.001 |
| Turkmenistan | Female | | | < 0.001 | | 0.61 | | < 0.001 | | 0.12 | | < 0.001 | | 1 | | | 0.02 | | 0.412 | < 0.001 | | < 0.001 | | 0.1 | < 0.001 | 0.02 | < 0.001 |
| Turkmenistan | Male | | | < 0.001 | | 0.26 | | < 0.001 | | 0.69 | | < 0.001 | | 0.997 | | | < 0.001 | | 0.137 | < 0.001 | | < 0.001 | | 0 | < 0.001 | 0.027 | < 0.001 |
| Tuvalu | Both | | | 1 | | 1 | | 1 | | 1 | | 1 | | 1 | | | 1 | | 1 | 1 | | 1 | | 0.94 | 0.407 | 0.985 | 0.982 |
| Tuvalu | Female | | | 1 | | 1 | | 1 | | 1 | | 1 | | 1 | | | 1 | | 1 | 1 | | 1 | | 0.99 | 0.688 | 0.997 | 0.991 |
| Tuvalu | Male | | | 1 | | 1 | | 1 | | 1 | | 1 | | 1 | | | 1 | | 1 | 1 | | 1 | | 1 | 0.503 | 0.999 | 0.989 |
| Uganda | Both | | | < 0.001 | | 1 | | 0.919 | | 0.99 | | < 0.001 | | 1 | | | 0.61 | | 1 | 0.022 | | 0 | | 0.03 | < 0.001 | 0.464 | 0.001 |
| Uganda | Female | | | < 0.001 | | 1 | | 1 | | 1 | | < 0.001 | | 1 | | | 0.88 | | 1 | 0.437 | | 0.41 | | 0.45 | < 0.001 | 0.799 | 0.061 |
| Uganda | Male | | | < 0.001 | | 1 | | 1 | | 1 | | < 0.001 | | 1 | | | 0.73 | | 1 | 0.041 | | 0.05 | | 0.54 | < 0.001 | 0.637 | 0.002 |
| Ukraine | Both | | | < 0.001 | | < 0.001 | | < 0.001 | | 0.03 | | < 0.001 | | < 0.001 | | | < 0.001 | | < 0.001 | < 0.001 | | < 0.001 | | 0 | < 0.001 | 0.47 | < 0.001 |
| Ukraine | Female | | | < 0.001 | | < 0.001 | | < 0.001 | | 0.86 | | < 0.001 | | < 0.001 | | | < 0.001 | | < 0.001 | < 0.001 | | < 0.001 | | 0.55 | < 0.001 | 0.282 | < 0.001 |
| Ukraine | Male | | | < 0.001 | | < 0.001 | | < 0.001 | | < 0.001 | | < 0.001 | | < 0.001 | | | < 0.001 | | < 0.001 | < 0.001 | | < 0.001 | | 0 | < 0.001 | 0.54 | < 0.001 |
| United Arab Emirates | Both | | | < 0.001 | | 1 | | 0.933 | | 1 | | < 0.001 | | 1 | | | 0.45 | | 1 | 0.241 | | 0.16 | | 0.19 | < 0.001 | 0.897 | 0.122 |
| United Arab Emirates | Female | | | < 0.001 | | 1 | | 1 | | 1 | | < 0.001 | | 1 | | | 0.93 | | 1 | 0.954 | | 0.94 | | 0.53 | < 0.001 | 0.886 | 0.552 |
| United Arab Emirates | Male | | | < 0.001 | | 1 | | 1 | | 1 | | < 0.001 | | 1 | | | 0.6 | | 1 | 0.512 | | 0.55 | | 0.51 | < 0.001 | 0.935 | 0.255 |
| United Kingdom | Both | | | < 0.001 | | < 0.001 | | < 0.001 | | < 0.001 | | < 0.001 | | < 0.001 | | | < 0.001 | | < 0.001 | < 0.001 | | < 0.001 | | < 0.001 | < 0.001 | < 0.001 | < 0.001 |
| United Kingdom | Female | | | < 0.001 | | < 0.001 | | < 0.001 | | < 0.001 | | < 0.001 | | < 0.001 | | | < 0.001 | | < 0.001 | < 0.001 | | < 0.001 | | < 0.001 | < 0.001 | < 0.001 | < 0.001 |
| United Kingdom | Male | | | < 0.001 | | < 0.001 | | < 0.001 | | 0.03 | | < 0.001 | | < 0.001 | | | 0 | | < 0.001 | < 0.001 | | < 0.001 | | < 0.001 | < 0.001 | < 0.001 | < 0.001 |
| United Republic of Tanzania | Both | | | < 0.001 | | 0.96 | | < 0.001 | | 0.95 | | < 0.001 | | 0.999 | | | 0.95 | | 0.893 | 0.101 | | < 0.001 | | < 0.001 | < 0.001 | 0.604 | 0.011 |
| United Republic of Tanzania | Female | | | < 0.001 | | 1 | | < 0.001 | | 1 | | < 0.001 | | 1 | | | 0.96 | | 1 | 0.519 | | < 0.001 | | < 0.001 | < 0.001 | 0.951 | 0.114 |
| United Republic of Tanzania | Male | | | < 0.001 | | 1 | | < 0.001 | | 0.92 | | < 0.001 | | 1 | | | 0.99 | | 0.999 | 0.146 | | < 0.001 | | < 0.001 | < 0.001 | 0.695 | 0.013 |
| United States of America | Both | | | < 0.001 | | < 0.001 | | < 0.001 | | < 0.001 | | < 0.001 | | < 0.001 | | | < 0.001 | | < 0.001 | < 0.001 | | < 0.001 | | < 0.001 | < 0.001 | < 0.001 | 0.078 |
| United States of America | Female | | | < 0.001 | | < 0.001 | | < 0.001 | | < 0.001 | | < 0.001 | | < 0.001 | | | < 0.001 | | < 0.001 | < 0.001 | | < 0.001 | | < 0.001 | < 0.001 | < 0.001 | < 0.001 |
| United States of America | Male | | | < 0.001 | | < 0.001 | | < 0.001 | | < 0.001 | | < 0.001 | | < 0.001 | | | < 0.001 | | < 0.001 | < 0.001 | | < 0.001 | | < 0.001 | < 0.001 | < 0.001 | 0.442 |
| United States Virgin Islands | Both | | | < 0.001 | | 1 | | 1 | | 1 | | 0.01 | | 1 | | | 1 | | 1 | 1 | | 1 | | 0.87 | < 0.001 | 0.956 | 0.977 |
| United States Virgin Islands | Female | | | < 0.001 | | 1 | | 1 | | 1 | | 0.38 | | 1 | | | 1 | | 1 | 1 | | 1 | | 0.93 | 0.004 | 0.988 | 0.946 |
| United States Virgin Islands | Male | | | < 0.001 | | 1 | | 1 | | 1 | | 0.44 | | 1 | | | 1 | | 1 | 1 | | 1 | | 0.78 | < 0.001 | 0.975 | 0.96 |
| Uruguay | Both | | | < 0.001 | | 0.11 | | < 0.001 | | 0.01 | | < 0.001 | | 1 | | | 0.28 | | 0.046 | 0.064 | | < 0.001 | | < 0.001 | < 0.001 | 0.007 | 0.026 |
| Uruguay | Female | | | < 0.001 | | 1 | | < 0.001 | | 0.76 | | < 0.001 | | 1 | | | 0.32 | | 0.999 | 0.059 | | < 0.001 | | < 0.001 | < 0.001 | 0.097 | 0.021 |
| Uruguay | Male | | | < 0.001 | | 0.83 | | < 0.001 | | 0.26 | | < 0.001 | | 1 | | | 0.6 | | 0.669 | 0.492 | | < 0.001 | | < 0.001 | < 0.001 | 0.084 | 0.216 |
| Uzbekistan | Both | | | < 0.001 | | < 0.001 | | < 0.001 | | < 0.001 | | < 0.001 | | < 0.001 | | | 0.48 | | < 0.001 | < 0.001 | | < 0.001 | | < 0.001 | < 0.001 | < 0.001 | < 0.001 |
| Uzbekistan | Female | | | < 0.001 | | < 0.001 | | < 0.001 | | < 0.001 | | < 0.001 | | < 0.001 | | | 0.2 | | < 0.001 | < 0.001 | | < 0.001 | | < 0.001 | 0.14 | < 0.001 | < 0.001 |
| Uzbekistan | Male | | | < 0.001 | | < 0.001 | | < 0.001 | | 0.02 | | < 0.001 | | 0.171 | | | 0.78 | | < 0.001 | < 0.001 | | < 0.001 | | < 0.001 | < 0.001 | < 0.001 | < 0.001 |
| Vanuatu | Both | | | 0.009 | | 1 | | 1 | | 1 | | 0.92 | | 1 | | | 1 | | 1 | 1 | | 1 | | 0.81 | 0.002 | 0.992 | 0.918 |
| Vanuatu | Female | | | 0.991 | | 1 | | 1 | | 1 | | 1 | | 1 | | | 1 | | 1 | 1 | | 1 | | 0.94 | 0.162 | 0.988 | 0.972 |
| Vanuatu | Male | | | 0.03 | | 1 | | 1 | | 1 | | 0.98 | | 1 | | | 1 | | 1 | 1 | | 1 | | 0.92 | 0.01 | 0.999 | 0.98 |
| Venezuela (Bolivarian Republic of) | Both | | | < 0.001 | | 0.4 | | < 0.001 | | 0.81 | | < 0.001 | | 0.538 | | | 0.26 | | 0.248 | < 0.001 | | < 0.001 | | < 0.001 | < 0.001 | 0.446 | < 0.001 |
| Venezuela (Bolivarian Republic of) | Female | | | < 0.001 | | 0.42 | | < 0.001 | | 0.14 | | < 0.001 | | 0.446 | | | 0.2 | | 0.254 | < 0.001 | | < 0.001 | | < 0.001 | < 0.001 | 0.419 | < 0.001 |
| Venezuela (Bolivarian Republic of) | Male | | | < 0.001 | | 0.19 | | < 0.001 | | 0.38 | | < 0.001 | | 0.754 | | | 0.39 | | 0.113 | 0.511 | | < 0.001 | | < 0.001 | < 0.001 | 0.151 | 0.687 |
| Viet Nam | Both | | | < 0.001 | | < 0.001 | | < 0.001 | | < 0.001 | | < 0.001 | | < 0.001 | | | 0.6 | | < 0.001 | < 0.001 | | < 0.001 | | < 0.001 | < 0.001 | 0.064 | < 0.001 |
| Viet Nam | Female | | | < 0.001 | | < 0.001 | | < 0.001 | | < 0.001 | | < 0.001 | | < 0.001 | | | 0.57 | | < 0.001 | < 0.001 | | < 0.001 | | < 0.001 | < 0.001 | 0.654 | < 0.001 |
| Viet Nam | Male | | | < 0.001 | | 0 | | < 0.001 | | < 0.001 | | < 0.001 | | 0.039 | | | 0.81 | | < 0.001 | < 0.001 | | < 0.001 | | < 0.001 | < 0.001 | < 0.001 | < 0.001 |
| Yemen | Both | | | < 0.001 | | < 0.001 | | < 0.001 | | < 0.001 | | < 0.001 | | < 0.001 | | | 0.01 | | < 0.001 | < 0.001 | | < 0.001 | | < 0.001 | < 0.001 | 0.772 | < 0.001 |
| Yemen | Female | | | < 0.001 | | 0.89 | | 0.21 | | 0.61 | | < 0.001 | | 0.853 | | | 0.55 | | 0.752 | 0.029 | | 0.05 | | 0.19 | < 0.001 | 0.972 | 0.009 |
| Yemen | Male | | | < 0.001 | | 0.05 | | < 0.001 | | 0.07 | | < 0.001 | | 0.641 | | | 0.03 | | 0.02 | < 0.001 | | < 0.001 | | < 0.001 | < 0.001 | 0.273 | 0.002 |
| Zambia | Both | | | < 0.001 | | 1 | | 0.799 | | 0.99 | | < 0.001 | | 1 | | | 0.61 | | 0.994 | 0.203 | | 0.05 | | 0.06 | < 0.001 | 0.428 | 0.026 |
| Zambia | Female | | | < 0.001 | | 1 | | 1 | | 1 | | < 0.001 | | 1 | | | 0.92 | | 1 | 0.81 | | 0.62 | | 0.24 | < 0.001 | 0.925 | 0.244 |
| Zambia | Male | | | < 0.001 | | 1 | | 1 | | 1 | | < 0.001 | | 1 | | | 0.82 | | 1 | 0.507 | | 0.54 | | 0.5 | < 0.001 | 0.879 | 0.094 |
| Zimbabwe | Both | | | < 0.001 | | 1 | | < 0.001 | | 1 | | < 0.001 | | 1 | | | 0.84 | | 1 | < 0.001 | | < 0.001 | | < 0.001 | < 0.001 | 0.807 | < 0.001 |
| Zimbabwe | Female | | | < 0.001 | | 1 | | < 0.001 | | 0.99 | | < 0.001 | | 1 | | | 0.57 | | 1 | < 0.001 | | < 0.001 | | < 0.001 | < 0.001 | 0.634 | < 0.001 |
| Zimbabwe | | Male | < 0.001 | | 1 | | 0.004 | 1 | < 0.001 | | 1 | | 0.92 | | 1 | 0.049 | | < 0.001 | < 0.001 | < 0.001 | 0.695 | 0.002 |  |  |  |  |  |
